# Myocardial adaption to HI(R)T in previously untrained men with a randomized, longitudinal cardiac MR imaging study (Physical adaptions in Untrained on Strength and Heart trial, PUSH-trial)

**DOI:** 10.1371/journal.pone.0189204

**Published:** 2017-12-07

**Authors:** Michael Scharf, Derya Oezdemir, Axel Schmid, Wolfgang Kemmler, Simon von Stengel, Matthias S. May, Michael Uder, Michael M. Lell

**Affiliations:** 1 Department of Radiology, Friedrich-Alexander-University Erlangen-Nuremberg, Erlangen, Bavaria, Germany; 2 Department of Medical Physics, Friedrich-Alexander-University Erlangen-Nuremberg, Erlangen, Bavaria, Germany; 3 Department of Radiology and Nuclear Medicine, Hospital of Nuremberg, Paracelsus Medical University, Nuremberg, Bavaria, Germany; Freeman Hospital, UNITED KINGDOM

## Abstract

**Objective:**

Although musculoskeletal effects in resistance training are well described, little is known about structural and functional cardiac adaption in formerly untrained subjects. We prospectively evaluated whether short term high intensity (resistance) training (HI(R)T) induces detectable morphologic cardiac changes in previously untrained men in a randomized controlled magnetic resonance imaging (MRI) study.

**Materials and methods:**

80 untrained middle-aged men were randomly assigned to a HI(R)T-group (n = 40; 43.5±5.9 years) or an inactive control group (n = 40; 42.0±6.3 years). HI(R)T comprised 22 weeks of training focusing on a single-set to failure protocol in 2–3 sessions/week, each with 10–13 exercises addressing main muscle groups. Repetitions were decreased from 8–10 to 3–5 during study period. Before and after HI(R)T all subjects underwent physiologic examination and cardiac MRI (cine imaging, tagging).

**Results:**

Indexed left (LV) and right ventricular (RV) volume (LV: 76.8±15.6 to 78.7±14.8 ml/m^2^; RV: 77.0±15.5 to 78.7±15.1 ml/m^2^) and mass (LV: 55.5±9.7 to 57.0±8.8 g/m^2^; RV: 14.6±3.0 to 15.0±2.9 g/m^2^) significantly increased with HI(R)T (all p<0.001). Mean LV and RV remodeling indices of HI(R)T-group did not alter with training (0.73g/mL and 0.19g/mL, respectively [p = 0.96 and p = 0.87]), indicating balanced cardiac adaption. Indexed LV (48.4±11.1 to 50.8±11.0 ml/m^2^) and RV (48.5±11.0 to 50.6±10.7 ml/m^2^) stroke volume significantly increased with HI(R)T (p<0.001). Myocardial strain and strain rates did not change following resistance exercise. Left atrial volume at end systole slightly increased after HI(R)T (36.2±7.9 to 37.0±8.4 ml/m^2^, p = 0.411), the ratio to end-diastolic LV volume at baseline and post-training was unchanged (0.47 vs. 0.47, p = 0.79).

**Conclusion:**

22 weeks of HI(R)T lead to measurable, physiological changes in cardiac atrial and ventricular morphologic characteristics and function in previously untrained men.

**Trial regristration:**

The PUSH-trial is registered at the US National Institutes of Health (ClinicalTrials.gov), NCT01766791.

## Introduction

Resistance training (RT) is an increasingly popular method of conditioning for recreational purposes and in competitive athletes. In 2011, about 24% of middle-aged and older adults in the US performed strength-training activities at least twice a week [1]. Besides recreation, RT is increasingly used in prevention and rehabilitation [2].

Although the benefits of RT on the skeletal muscle are well established [3], the effect on cardiac morphology and function remains equivocal. Based on predominantly cross-sectional studies of elite athletes using echocardiography, two types of ventricular remodeling have been postulated: ‘Concentric’ cardiac hypertrophy in response to strength training due to pressure load and ‘eccentric’ hypertrophy in endurance trained athletes as a result of volume load [4–7]. In concentric remodeling myocardial mass and wall thickness are increased without substantial change in cavity size. Eccentric cardiac adaption is characterized by cavity dilation and thickening of the ventricular wall [8]. Data from cardiovascular magnetic resonance imaging (MRI), allowing evaluation of smaller changes in left (LV) and right ventricular (RV) cardiac volume and mass than other imaging modalities, are limited [9]. A cross-sectional cardiovascular MRI study by Fleck et al. [10] showed concentric LV myocardial adaption in RT whereas others did not [11,12]. To our knowledge there are no longitudinal cardiovascular MRI studies, especially in previously sedentary individuals, to evaluate specific cardiac adaption in RT. In addition, most previous cardiovascular MRI studies focused only on specific aspects of cardiac adaption, for example, LV hypertrophy without assessment of the RV [13]. Besides beneficial cardiovascular adaption [14] there is still controversy whether high-intensity RT (HI(R)T) may also induce pathologic structural remodeling of the heart [15]. A comprehensive evaluation of the myocardial structural changes associated with RT is necessary to differentiate physiologic changes from cardiac maladaption. The Physical adaptions in Untrained on Strength and Heart (PUSH)-trial was designed to address these issues.

## Materials and methods

### Trial design

The Physical adaptions in Untrained on Strength and Heart (PUSH) study was conducted in collaboration of the Department of Radiology and the Institute of Medical Physics (IMP), Friedrich-Alexander University Erlangen-Nuremberg (FAU), Germany. Study period was from April 2012 to July 2013. We conducted a 22-week randomized controlled exercise trial with a parallel group design, focusing on the effect of high intensity, single set resistance exercise protocols (HI(R)T) on functional and morphologic cardiac parameters in middle-aged (30–50 years old) untrained males compared to sedentary controls. Details of the whole PUSH trial have been described before [16].

The study was approved by the institutional review board of the Faculty of Medicine of the Friedrich-Alexander-University (FAU) of Erlangen-Nuremberg (ethics application number 53_12 B) and written informed consent was obtained from all subjects. PUSH complied with the Declaration of Helsinki “Ethical Principles for Medical Research Involving Human Subjects”. The study was registered at www.clinicaltrials.gov (NCT01766791).

### Outcomes

#### Primary study outcome

Myocardial mass (MM) changes as determined by cardiac MRI from baseline to the end of the intervention after 22 weeks of exercise.

#### Secondary study outcome

Changes in end-diastolic volume (EDV) as determined by cardiac MRI from baseline to the end of the intervention after 22 weeks of exercise.

### Changes of trial outcomes after trial commencement

Originally, we planned to assess the effects of detraining on changes in myocardial mass and end-diastolic volume induced by 22 weeks of HI(R)T. Therefore, we intended to perform follow-up cardiac MRI examinations six months after the end of the training period. However, because most of the participants (n = 31) continued RT after the intervention period, reversibility of myocardial adaption was not tested.

### Study population

Sample size was determined (G*power, version 3.1.3; 2009, Faul et al., Dusseldorf, Germany) [17]. We estimated that a sample size of 32 individuals in each group would have a power of 90% to detect a between-group difference of 5±3g in myocardial mass (MM) with α = 0.05. Assuming a drop-out rate of 20% our goal was to recruit 40 subjects per group.

**Fig 1:**
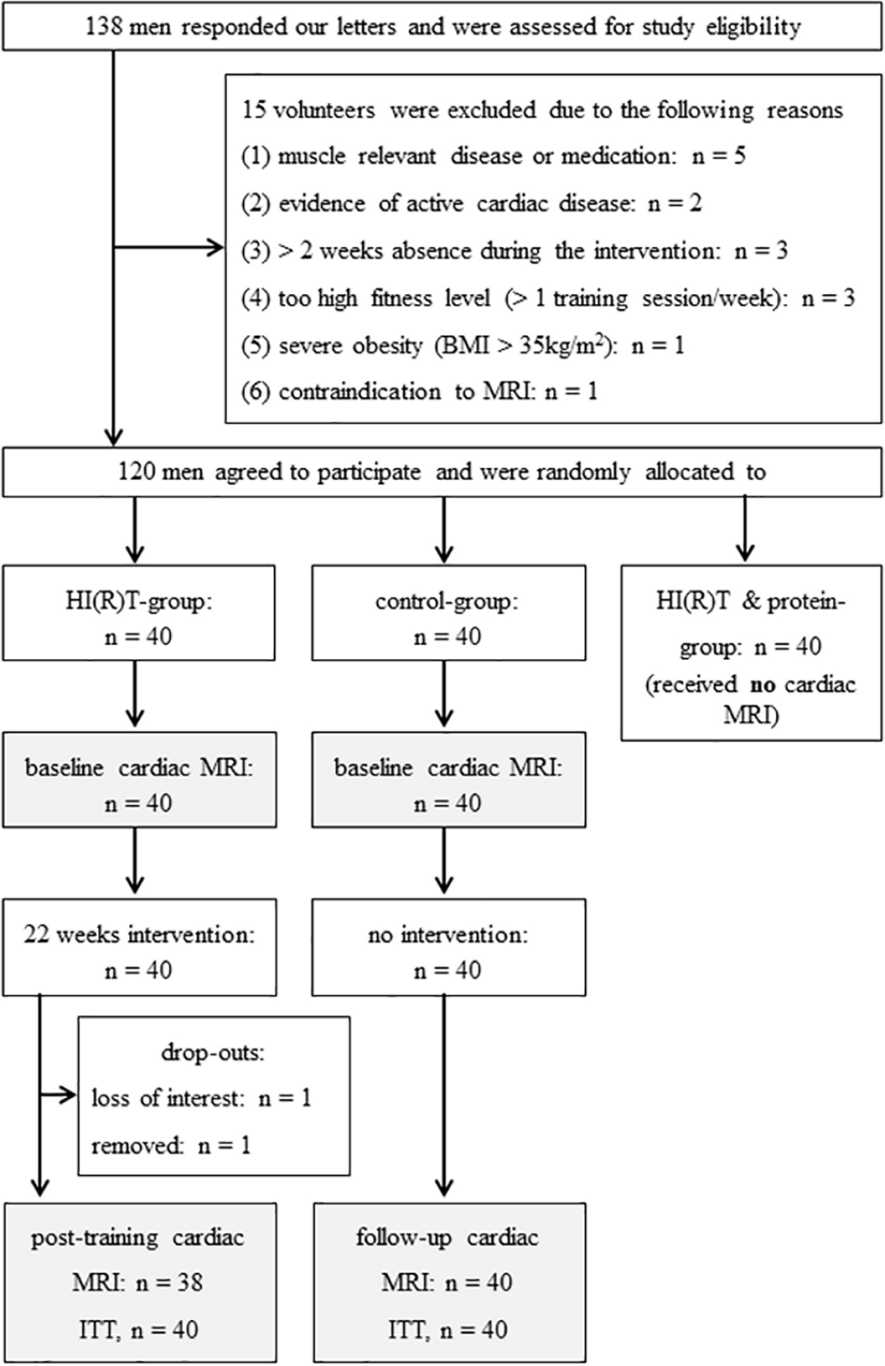
Flow-chart of the PUSH-study: 138 male volunteers were enrolled in this study. After clinical evaluations fifteen subjects were excluded from the study due to medical reasons. Three subjects refused random allocation to one study group. Finally, 120 participants were randomly assigned to a HI(R)T- (n = 40), control- (n = 40), and HI(R)T & protein supplementation-group (n = 40). The latter one was investigated by the collaborative institute and did not receive cardiac MRI [16]. All subjects of HI(R)T- and control-group received cardiac MRI before and after the intervention period. BMI, body mass index; HI(R)T, high-intensity (resistance) training; and ITT, intention-to-treat analysis.

Eligible study members were untrained (i.e., ≤ 1 resistance exercise session/week; ≤ 2 total exercise sessions/week during the last 2 years) men between 30 and 50 years of age. Initially, two-thousand randomly selected men in the area of Erlangen (Germany), aged 30–50 years, were contacted using the citizen’s register of the municipality. Of those who responded 138 men were assessed for eligibility (Fig 1). All participants completed a medical history and physical activity questionnaire. All subjects denied the use of illicit substances and underwent physical examination, 12-lead rest electrocardiography, and echocardiographic examinations. Hypertension was defined as a systolic blood pressure ≥ 140 mmHg, a diastolic blood pressure ≥ 90 mmHg, or use of antihypertensive medications. Prevalent diabetes mellitus was defined as fasting glucose ≥ 126 mg/dL, haemoglobin A1c ≥ 6.5%, or use of antidiabetic medications. After clinical evaluations fifteen subjects were excluded from the study because of a too high fitness level ([resistance] training > 1 session/week, n = 3), contraindication to MRI (n = 1), diseases/medications that affect muscle metabolism (n = 5), evidence of active cardiac/cardiovascular disease (n = 2), predictable absence during intervention > 2 weeks (n = 3), and severe obesity (body mass index > 35 kg/m^2^, n = 1). Of the 123 men remaining, three were unwilling to be randomly allocated to a group and quit the study. Physical characteristics at baseline and post-training, profile of risk factors, and past medical history anamnesis are shown in Table 1.

**Table 1:** Baseline and post-training physical characteristics of HI(R)T and control group.

	controls		HI(R)T		*P* Value
	baseline	follow-up	baseline	post-training	baseline
	(n = 40)	(n = 40)	(n = 40)	(n = 38)	
Parameter				(ITT, n = 40)	
Age, y	42.0±6.3	na	43.5±5.9	na	0.27
Height, m	1.80±7.8	na	1.81±7.0	na	0.77
Body weight, kg	86.3±15.2	86.7±14.8	88.9±14.9	88.5±14.6	0.45
Body mass index, kg/m^2^	26.5±4.2	26.6±4.0	27.2±4.0	27.1±4.0	0.48
BSA, m^2^	2.07±0.2	2.08±0.2	2.11±0.2	2.10±0.2	0.44
Resting heart rate, bpm	74.7±12.4	74.3±9.8	77.4±13.0	75.2±11.8	0.35
Systolic BP, mmHg	131±13.1	132±12.9	131±12.8	131±13.5	0.52
Diastolic BP, mmHg	87±8.4	88±9.2	88±7.9	87±8.5	0.61
Risk factors:					
• *Hypertension*, n (%)	6 (15)	na	5 (13)	na	0.80
• *Smoking*, n (%)	2 (5)	na	1 (2.5)	na	0.73
• *Diabetes mellitus*, n (%)	3 (8)	na	2 (5)	na	0.65
Past medical history:					
• *β-blockers*, n (%)	5 (13)	na	4 (10)	na	0.81
• *Furosemide*, n (%)	3 (8)	na	3 (8)	na	1
• *ACEi/ARBs*, n (%)	3 (8)	na	4 (10)	na	0.79

Data for physical characteristics are given as mean ± SD. Values for risk factors and past medical history are indicated as numbers and percentages. *P* values refer to intergroup differences at baseline. Distribution of cardiac risk factors and medications between the two groups was similar. BP indicates blood pressure; ACEi, angiotensin-converting enzyme inhibitor; ARB, angiotensin II inhibitor; BSA, body surface area; HI(R)T, high-intensity (resistance) training; ITT, intention-to-treat analyis; and na, not applicable.

### Randomization procedures

Stratified for age (4 stratas of 5 years), 120 participants were randomly assigned to three study arms by a uniform allocation rate (1 : 1 : 1): HI(R)T- (n = 40), control- (n = 40), and HI(R)T & protein supplementation-group (n = 40) (Fig 1). The latter one was investigated by the collaborative institute and did not receive cardiac MRI [16]. For the allocation, lots in opaque plastic shells were drawn by the participants themselves. Neither participants nor researchers knew the allocation beforehand. Finally, group status of the study subjects was listed and participants were assigned to the different study arms by the principle investigators.

### Training procedure

Before the actual training period of HI(R)T-group there was a pre-phase consisting of two weeks initiation and four weeks of conditioning (applying 1–2 sessions/week, 10 exercises, each with two sets of 10–15 repetitions). Members of HI(R)T-group were then trained for 16 weeks with a linearly periodized resistance exercise protocol subdivided in 4-week phases with each 4^th^ week as a rest week. The exercise program consisted of two to three supervised sessions per week addressing the main muscle groups by 10–13 exercises out of a pool of 17 exercises conducted on resistance devices (MedX, Ocala, FL, USA). We used a single-set-to-failure protocol with prescribed exercise intensity as a range of repetitions (e.g., 6–8) that had to be accomplished under the premise of work to momentary muscle failure. The number of repetitions was consistently decreased from 8–10 to 3–5 repetitions during the training period. Daily data were recorded on duration and type of training activities performed throughout the study period. During the whole study period, the control group was requested to maintain their lifestyle and physical activity. Detailed exercise program and procedure has been described previously [16].

### Cardiac MRI protocol

**Fig 2:**
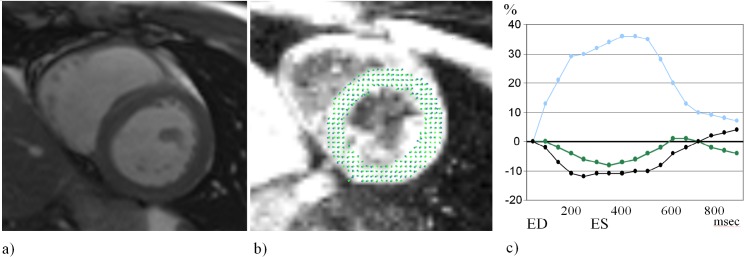
**Pre- and post-training cardiac examination:** (a) End-diastolic short-axis cine MR images acquired with steady-state free-precession sequences with electrocardiographic gating and (b) evaluation of myocardial strain using InTag. (c) Curves show percentage changes of circumferential (black line), longitudinal (green line) and radial (blue line) strain throughout the cardiac cycle. ED = end-diastole; ES = end-systole.

Cardiac MRI at baseline (before the introductory training) and post-training MRI was performed on a 1.5-T unit (Magnetom Avanto, VB 17A; Siemens Healthcare, Erlangen, Germany) using a six-channel phased-array surface and spine matrix receiver coil. Four-, three-, and two-chamber long- and short-axis cine images (Fig 2) were acquired by using breath-hold balanced steady-state free-precession sequences with retrospective electrocardiographic gating with the following scan parameters: field of view, 215 to 265×300 to 340 mm^2^; slice thickness, 6 mm; intersection-gap, 1.5 mm; repetition/echo time, 41.25 to 50.7/1.12 to 1.38 ms; flip angle, 61° to 75°; matrix, 105 to 156×192 to 256; pixel size, 1.5 to 2.8×1.2 to 2.0 mm^2^; number of reconstructed phases, 25; parallel acquisition acceleration factor of 2. Myocardial strain was assessed using a prototype balanced steady-state free-precession-based tagging sequence with the following image acquisition parameters: complementary spatial modulation of magnetization, field of view, 340×340 mm^2^; slice thickness, 6 mm; tag-spacing, 6 mm; repetition/echo time, 32.23/1.23 ms; flip angle, 20°; matrix, 77×256; PAT acceleration factor of 2.

### Blinding

Outcome assessors were blinded to study subjects’ group assignment. All personal data was removed from cardiac MRI exams by the study nurse. Readers evaluated studies independently and in random order.

### Image analysis

Quantitative image analysis was performed using dedicated software (Argus 4.01; Siemens Healthcare, Erlangen, Germany). LV and RV functional analysis was performed in consensus by two observers with more than 5 (—) and more than 14 (—) years of experience in MRI. Tracing of the endo- and epicardial borders from base to apex was performed manually at end diastole and end systole. Papillary muscles and epicardial adipose tissue were excluded. All results were divided by BSA to minimize differences of myocardium parameters related to weight and height. LV wall thickness was defined as the average of six segment thickness measurements (anterior, anterolateral, anteroseptal, inferior, inferolateral, and inferoseptal). We calculated the LV and RV remodeling index (myocardial mass/end-diastolic volume). An increased remodeling index is consistent with concentric hypertrophy, whereas a reduced remodeling index is indicative of isolated cavitary dilation [18]. In addition, the maximum left atrial (LA) volume was calculated according to the biplane area-length method and indexed for BSA [19]. An enlarged LA volume index was defined as greater than 55mL/m^2^. Three-dimensional left ventricular analysis of myocardial strain was performed using semi-automatic software (InTag, CREATIS, Lyon, France).

### Statistical analysis

Statistical analysis was performed using software (SPSS, 21.0; SPSS, Chicago, Illinois, USA and R: A Language and Environment for Statistical Computing 2016, Vienna, Austria). The primary analysis was intention-to-treat and involved all patients who were randomly assigned, independently of compliance or lost to follow-up. Normal distribution of variables was checked graphically and statistically (Shapiro-Wilks-Test). Change from baseline was analyzed and compared in HI(R)T- versus control-group in a linear mixed effects model with random intercept where measurement occasions are nested in patients. Patients who were lost to follow-up are considered to be missing at random conditional on the previous measurement occasions (MAR) and were included in the model via estimation of the covariance structure within maximum likelihood estimation. Mean changes from baseline ± standard errors are shown together with baseline values ± standard deviations. Differences in mean changes of HI(R)T-group versus control-group are presented with 95% confidence interval and error probability (p value). Each shown result is inherently adjusted for baseline differences in considered outcome but not for other baseline values. Analyses adjusted for age, BMI, BSA and heart rate are not shown here, but yielded no substantially different results. A two-sided P-value of less than .05 was considered statistically significant.

## Results

During the intervention period there were two drop-outs in the HI(R)T-group (removed, n = 1; loss of interest, n = 1). No significant intra- or intergroup differences for baseline and post-training physical characteristics, risk factors, and past medical history were determined for HI(R)T and controls (Table 1). In HI(R)T resting heart rate marginally but not significantly decreased from 77 bpm at baseline to 75 bpm post-training (p = 0.081). Electrocardiographic patterns in exercise and control group remained unchanged during the study period.

MRI-derived baseline and post-training LV and RV functional and morphologic parameters are detailed in Table 2. Image quality was diagnostic in all the cardiac MRI examinations. None of the participants, except one control subject, had a decreased LV ejection fraction (EF) below 50%, neither before nor after the intervention period. All RV EF values were within the normal range for healthy adults (47%-74%).

**Table 2:** LV and RV morphologic and functional parameters of HI(R)T- and control group at baseline and post-training.

	controls		HI(R)T		HI(R)T vs. controls	
Parameter	baseline (n = 40)	change from baseline	baseline (n = 40)	change from baseline	difference of mean change	*p-*value (mixed model)
	(mean±SD)	(mean±SD)	(mean±SD)	(mean±SD)	[95%CI]	
LV						
End-diastolic volume index (mL/m^2^)	78.5±14.8	-0.2±0.3	76.8±15.6	1.9±0.3	2.1 [1.4; 2.9]	<0.001*
End-systolic volume index (mL/m^2^)	30.0±8.4	0.1±0.2	28.4±8.8	-0.5±0.3	-0.6 [-1.3; 0.1]	0.114
Stroke volume index (mL/m^2^)	48.6±10.6	0.3±0.3	48.4±11.1	2.4±0.3	2.7 [1.9; 3.5]	<0.001*
Mass index at end-diastole (g/m^2^)	55.4±9.2	-0.2±0.2	55.5±9.7	1.5±0.2	1.7 [1.1; 2.3]	<0.001*
Wall thickness (mm)	7.9±1.5	0.2±0.1	8.3±1.6	0.1±0.1	-0.1 [-0.4; 0.3]	0.786
Ejection fraction (%)	61.8±7.7	-0.2±0.3	63.2±2.7	1.5±0.3	1.7 [0.8; 2.5]	<0.001*
RV						
End-diastolic volume index (mL/m^2^)	79.0±14.7	-0.7±0.3	77.0±15.5	1.7±0.3	2.4 [1.5; 3.4]	<0.001*
End-systolic volume index (mL/m^2)^	30.4±8.5	-0.4±0.4	29.0±9.0	-0.9±0.4	-0.5 [-1.6; 0.5]	0.335
Stroke volume index (mL/m^2^)	48.6±10.4	-0.4±0.3	48.5±11.0	2.2±0.3	2.5[1.7; 3.4]	<0.001*
Mass index at end-diastole (g/m^2^)	15.0±2.8	-0.1±0.1	14.6±3.0	0.3±0.1	0.5 [0.3; 0.6]	<0.001*
Ejection fraction (%)	61.6±7.8	0.1±0.3	63.0±8.2	1.3±0.3	1.2 [0.3; 2.1]	0.010*
Cardiac index (L/min/m^2^)	3.6±1.0	-0.2±0.1	3.7±1.1	0.0±0.1	0.2 [-0.0; 0.3]	0.068

Data for baseline values and changes after the intervention period are given as mean ± standard deviations (SD). *P* values of mixed model analysis are indicated for differences of mean changes [95% confidence interval, CI] from baseline to follow-up between control subjects and HI(R)T-group. Ejection fraction = (stroke volume/end-diastolic volume) x 100. HI(R)T indicates high-intensity (resistance) training; LV = left ventricle; and RV = right ventricle.

*Statistically significant values.

**Fig 3:**
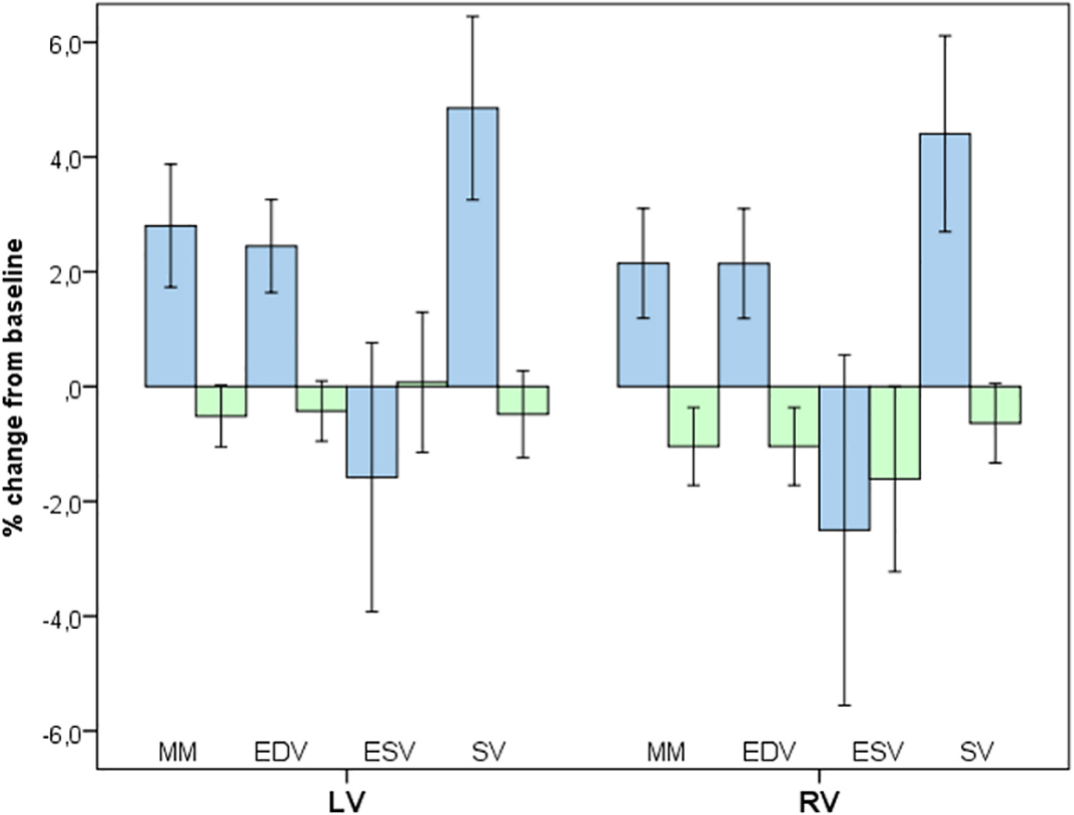
Cardiac morphologic changes after 22 weeks of HI(R)T compared to inactive controls: Effect of 22 weeks of HI(R)T on MRI derived measures of indexed LV and RV myocardial mass (MM), end-diastolic volume (EDV), end-systolic volume (ESV), and stroke volume (SV). Parameters of the HI(R)T-group significantly increased with training whereas values for controls remained unchanged or slightly declined. LV = left ventricle; and RV = right ventricle.

Compared to controls, indexed LV and RV myocardial mass had significantly increased in the HI(R)T-group after 22 weeks training by 2.8% and 2.2% (Fig 3). Mean values and ranges for LV wall thickness in both study arms remained unchanged (HI(R)T: baseline, 6.7–10.4 mm and post-training 6.6–11.0 mm; controls: 5.7–11.0 mm and 5.7–10.9 mm; p = 0.766).

Parallel to enhanced myocardial mass in HI(R)T subjects, values for indexed LV and RV end-diastolic volumes were significantly higher (2.5% and 2.2%) after the intervention period compared to controls. Higher end-diastolic volumes were associated with greater LV and RV stroke volumes (4.9%, 4.4%) and slightly smaller end-systolic volumes (-1.6%, -2.5%). Only one subject in the HI(R)T-group (LV: 99 ml) and three controls (LV: 95, 102, 103 ml) were beyond normal LV ranges for healthy, male, non-athletic subjects (LV: 47–92 mL/m^2^). No subject exceeded normal limits for RV end-diastolic volumes (RV: 55–105 mL/m^2^).

**Fig 4:**
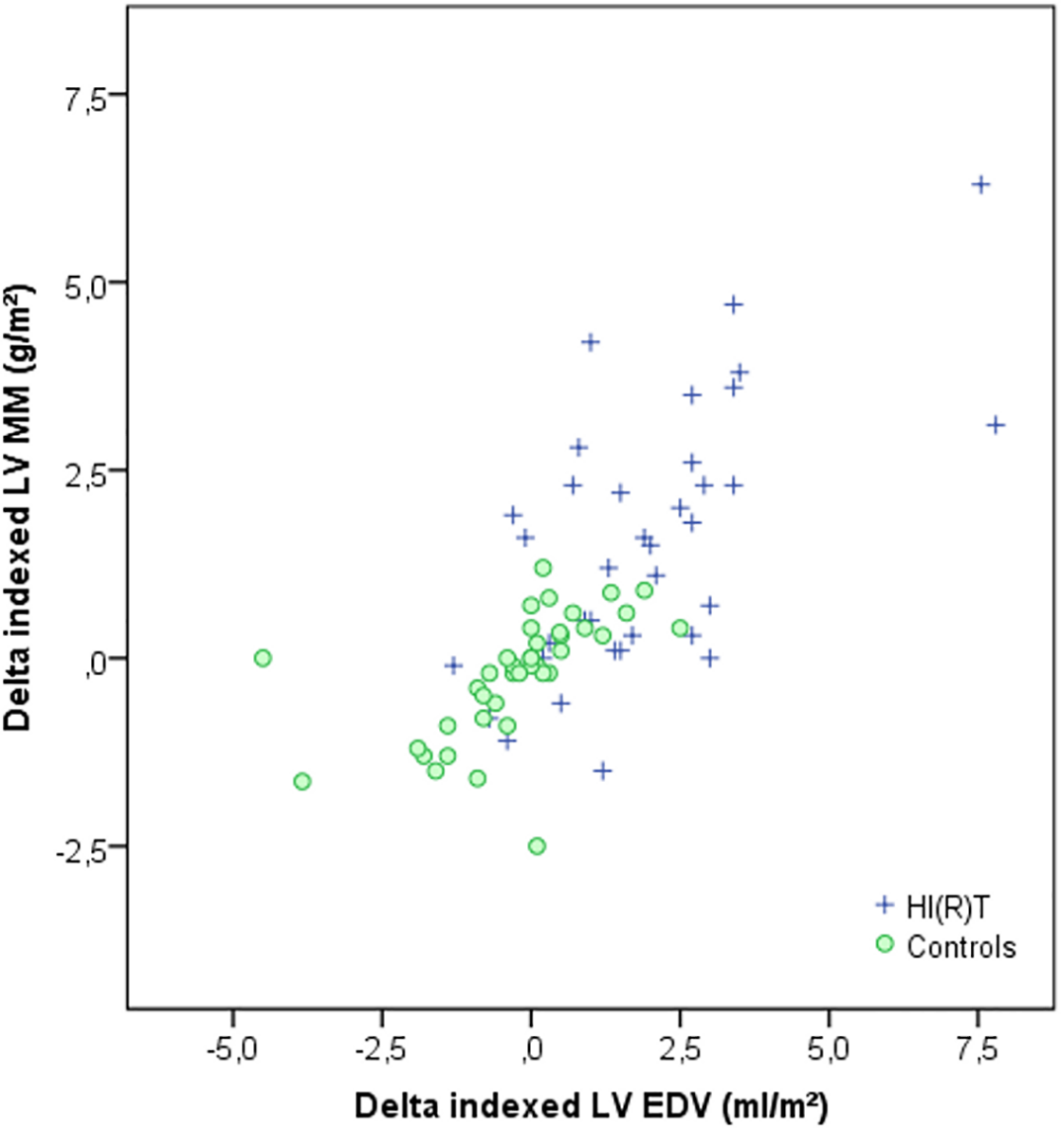
Changes in LV remodeling indices after HI(R)T compared to inactive controls: Graph shows the LV remodeling indices, expressed as the ratio of changes in indexed myocardial mass (MM) to indexed end-diastolic volume (EDV) between baseline and follow-up MRI. Even though the indexed MM and EDV were significantly greater after HI(R)T, the mean value for the LV remodeling index was similar to that of the control subjects. LV = left ventricular.

In the HI(R)T-group changes in LV and RV myocardial mass were highly correlated with increments in end-diastolic volume (Pearson correlations: LV = 0.649 and RV = 0.690; both p<0.001). LV and RV remodeling indices in the exercise-group remained unchanged after 22 weeks of HI(R)T (LV, 0.74g/mL ± 0.01; RV, 0.19g/mL ± 0.08; p = 0.96 and p = 0.87) compared to baseline values (LV, 0.73g/mL ± 0.10; RV, 0.19g/mL ± 0.02). This is indicative of balanced cardiac adaption (Fig 4). In addition, in HI(R)T LV-to-RV ratios for indexed end-diastolic volume and myocardial mass did not alter with training (EDV: 1.00 vs. 1.00, p = 0.025; MM: 3.84 vs. 3.86, p = 0.981).

Mean value for indexed maximum LA volume at end systole increased by 2.2% from 36.2 mL/m^2^ ± 7.9 to 37.0 mL/m^2^ ± 8.4 (p = 0.411) with HI(R)T. In control- and HI(R)T-group ratios of indexed end-systolic LA volume to end-diastolic LV volume at baseline (0.47 vs. 0.47, p = 0.890) and after the intervention (0.47 vs. 0.46, p = 0.488) were comparable.

Peak systolic circumferential strain before and after the intervention period ranged between -10% to -25% without significant intergroup differences for HI(R)T- and control-group subjects (Table 3). Intragroup analysis of HI(R)T-members showed no significant differences between baseline and post-training within the three LV levels for circumferential (Ecc), radial (Err), and for longitudinal strain (Ell).

**Table 3:** Mean peak systolic LV circumferential strain of HI(R)T- and control group at baseline and follow-up.

	Baseline			Follow-up		
	controls	HI(R)T		controls	HI(R)T	
Parameter	(n = 40)	(n = 40)	*P*-Value	(n = 40)	(n = 38) (ITT, n = 40)	*P* Value
LVES Ecc basal	-14.2±5.7	-14.4±4.1	0.755	-13.9±4.2	-14.2±3.3	0.679
LVES Ecc medial	-21.4±3.6	-21.9±2.9	0.669	-21.9±2.6	-21.5±2.7	0.429
LVES Ecc apikal	-19.5±4.1	-19.1±3.5	0.612	-19.4±3.8	-18.9±3.4	0.623

Data are given as mean in percentage shortening of circumferential strain (Ecc) at LVES ± SD. There were no statistical significant intra- or intergroup differences at the three short axis levels at baseline and follow-up. HI(R)T indicates high-intensity (resistance) training; ITT, intention-to-treat analysis; and LVES, left ventricular end-systole.

## Discussion

This longitudinal cardiac MRI study shows that a relatively short period (22 weeks) of HI(R)T in previously untrained male subjects induces significant morphologic changes of the LV and RV.

Previous cross-sectional echocardiographic [20–22] and MRI [10] studies postulated concentric cardiac hypertrophy in resistance training. In contrast, increase in LV and RV myocardial masses in our investigation was associated with an equivalent increment in end-diastolic ventricular volumes. These findings are supported by comparable remodeling indices of HI(R)T subjects at baseline and post-training, indicating balanced cardiac adaption without preponderance of one specific remodeling mechanism.

The discrepancy between results found in our investigation and previous cross-sectional studies might in part be explained by differences in imaging modalities. Compared to echocardiography, cardiac MRI provides high quality images and is intrinsically three dimensional (i.e., does not rely on geometric assumptions). Especially, evaluation of the RV myocardial mass and volume is limited using echocardiography [23,24]. Cardiac MRI is therefore considered the current reference standard for LV and RV volume and mass assessment [25]. Conflicting study findings might also be related to effects of type, intensity, and duration of training. In contrast to previous studies [10,20–22] our population consisted of sportive inexperienced subjects with presumably higher potential for cardiac remodeling than active sportsmen.

In the present study, there were no differences in the LV/RV ratios for volume and mass indices, indicating balanced cardiac adaption of both chambers. This is in contrast to a previous animal study in rats [26] postulating isolated LV hypertrophy without changes in the RV and agrees with a longitudinal cardiac MRI study by Spence et al. [24], showing mild morphological RV adaption after six months of intense resistance training.

Indexed LV and RV myocardial masses at baseline and after the training period were below previously reported mean values for healthy male subjects (LV, 69–112 g/m^2^ and RV, 16–36 g/m^2^, respectively) [27,28]. We suppose that this is related to a larger body surface area of our study subjects as compared to previous investigations. Indexed LV and RV myocardial mass above upper limits is supposed to be associated with an increase in cardiovascular mortality [29]. None of our study participants had exaggerated LV or RV myocardial mass values. This is due to participants’ low baseline fitness level and the relatively short training period within this study.

Mean LV wall thickness of all study subjects at baseline (8.1 mm) is lower than that of previously reported reference values (8.8–9.0 mm) for healthy, male, non-athletic subjects [5,30]. This discrepancy might be due to the selection of older individuals in our study (30–50 years). Compared to previous cross-sectional studies assessing elite strength trained athletes (range, 9–16 mm) [31,32] and Olympic weight lifters (mean, 9.8 mm) [33], LV ventricular septum thickness in our HI(R)T-members after the intervention period was much lower. This reflects the different fitness level.

Ventricular remodeling of HI(R)T-members in our investigation is accompanied by a balanced increase in LA dilation. However, LA enlargement in our study (2.2%) was lower than that observed in previously sedentary individuals after a comparable period of high-intensity interval training (13%) [34] and in long-time elite endurance athletes (up to 62%) [35].

Besides the beneficial cardiovascular effects of regular resistance exercise training there is still uncertainty whether high-intensity RT is associated with cardiac maladaption. Post mortem investigations in athletes suffering from sudden cardiac death detected myocardial interstitial fibrosis [36] and animal studies in rats have demonstrated that intensive resistance exercise leads to overexpression of LV proteome which similar changes found in the initial phase of heart failure [15]. It has been shown that strain-technologies can be used to evaluate the function of the ventricular fiber architecture [37] and to measure dynamic changes in the geometry and fibrous structure of the heart [38]. In our study, we found no association of HI(R)T in previously untrained men and myocardial damage. To assess myocardial deformation we focused on circumferential strain analysis as radial strain has been shown to be relatively imprecise, because only a small number of tags span the myocardial wall [39]. Circumferential strain values at systole in our study ranged from -10% to -25%. This corresponds to results of previous investigations in healthy subjects [40]. Global longitudinal strain is a strong predictor for major adverse cardiac events [41] and a meta-analysis by Huttin et al. [42] defined a cut-off level from -12.8% to -10.2% for peak systolic longitudinal strain to predict adverse remodeling after ST-segment elevation myocardial infarction. Unchanged longitudinal strain values in our study contrast with an echocardiographic longitudinal study by Schmidt et al., who showed an increase (by 6%) in systolic longitudinal strain after 12 months of RT [43]. Discrepancies probably reflect differences in imaging modality, training duration, and younger age of subjects in our study.

Physiologic morphologic adaption in our study is supported by unremarkable electrocardio-graphic patterns before and post-training. Therefore, we hypothesize that the mentioned changes in protein expression are related to a physiological process with transient myocardial adaption leading to enhanced cardiac structure and function. This is supported by the findings of a concomitant increased expression of protective proteins described in the study by Dantas et al. [15] and an improvement of collagen deposition and inflammatory profile after RT in chronic heart failure rats in a study by Alves et al. [44].

LV and RV ejection fraction in our study slightly improved after HI(R)T. This finding is attributable to the larger EDV of hypertrophied ventricles.

As our study comprises a relatively broad age range with a relevant distribution of physical characteristics, risk factor profile, and fitness level we hypothesize that physiologic cardiac adaptions to HI(R)T found in our investigation are generalizable to a large part of the population.

Our study has several limitations. Because our study involved only volunteers, not clinically suffering from cardiac disease, intravenous contrast agent was not applied. Therefore, we could not measure delayed gadolinium-enhancement, indicating potential cardiac fibrosis. Risk factors (e.g. hypertension) and impact of medication in a small number of participants might influence study results although subgroup study analysis showed no statistically relevant effect. We performed MRI only at the beginning and end of the training or waiting period, so we do not have information about time specific effects on cardiac adaption in the course of the study. This is due to the high training effort for participants and methodical limitations. Because most of the study subjects continued RT after the intervention period, reversibility of myocardial adaption could not be tested. The long-term dynamics of myocardial adaption in HI(R)T need to be addressed in further studies. We did not perform MRI with exercise or pharmacological stress, which in part may explain the relatively wide range in end-systolic volume. We also did not perform a direct comparison to the effects of other types of training (e.g., endurance training), known cardiac diseases (e.g. hypertrophic cardiomyopathy) or the abuse of anabolic substances to describe differences in cardiac adaption. We did not test participants for anabolic steroid abuse but all study subjects denied the use of illicit substances.

## Conclusion

This longitudinal cardiovascular MRI study suggests that a relatively short period of HI(R)T in previously untrained men is associated with physiological, significant changes in cardiac atrial and ventricular morphologic characteristics and function.

## Supporting information

S1 File Pre- and post-interventional LV and RV morphologic and functional data. (SAV)

S2 File CONSORT checklist. (DOC)

S3 File Trial study protocol (German). (PDF)

S4 File Trial study protocol (English translation). (DOCX)

S5 File Histograms of LV and RV residuals. (DOCX)

S6 File LV and RV mixed model analysis. (TXT)

S7 File LV and RV adjusted analysis. (TXT)
