# Supplementary material for: Myocardial adaption to HI(R)T in previously untrained men with a randomized, longitudinal cardiac MR imaging study (Physical adaptions in Untrained on Strength and Heart trial, PUSH-trial)
Source: PLoS One. 2017 Dec 7;12(12):e0189204. doi: 10.1371/journal.pone.0189204 (PMC5720775; doi:10.1371/journal.pone.0189204)
Supplement: S3 File — (PDF) [file pone.0189204.s003.pdf]

An die Geschäftsstelle der
Ethik-Kommission
der Medizinischen Fakultät
der FAU Erlangen-Nürnberg
Krankenhausstr. 12
91054 Erlangen

Antrag
an die Ethik-Kommission
der Medizinischen Fakultät

*Bitte **in deutscher Sprache** ausfüllen,*
Zutreffendes bitte ankreuzen.
Für multizentrische Studien mit Vorvotum einer nach Landesrecht gebildeten zuständigen Ethik-Kommission können Sie das verkürzte Antragsformular verwenden, abzurufen unter: http://www.ethik.med.uni-erlangen.de *(Anschlussvotum)*

Antrag auf Beurteilung eines Forschungsprojektes (keine Arzneimittelprüfung)

bitte 9-fach einschließlich Anlagen einreichen sowie 1-mal in elektronischer Fassung

Titel des Projektes:
Einfluss eines 16-wöchigen Kraft-Trainingsprogramms auf leistungsphysiologische und gesundheitsrelevante muskuläre und kardiale Größen bei untrainierten Männern im mittleren Lebensalter. Eine randomisierte kontrollierte Interventionsstudie mit modernen, bildgebenden Verfahren.

I. Projektleitung

1. Name der/des verantwortlichen Projektleiterin/s an der FAU:
Professor Dr. Wolfgang Kemmler[1], Osteoporose-Forschungszentrum, Institut für Medizinische Physik, FAU (Direktor: Professor Dr. Dr. Willi A. Kalender)
App.-Nr. 23999; E-Mail: wolfgang.kemmler@imp.uni-erlangen.de

Angaben über die Qualifikation der/des Versuchsleiterin/s:
Promotion, Habilitation (liegen bereits vor)

2. a) Weitere Teilnehmer/innen vor Ort (alphabetische Reihenfolge):
Prof. Dr. Klaus Engelke, IMP, FAU
PD. Dr. Michael Lell, Radiologisches Institut, FAU
Prof. Dr. Harald Quick, IMP, FAU
PD. Dr. Axel Schmid, Radiologisches Institut, FAU
Dr. Michael Scharf, Radiologisches Institut, FAU
Dr. Simon von Stengel, Osteoporoseforschungszentrum, FAU
Andreas Wittke, IMP, FAU

b) Weitere Prüfzentren (bei multizentrischen Studien): keine

3. Handelt es sich bei diesem Antrag um ein bereits von der Ethik-Kommission der Medizinischen Fakultät der Friedrich-Alexander-Universität Erlangen-Nürnberg begutachtetes Projekt?

ja (bitte lfd. Nr. angeben) ☒ nein

II. Forschungsvorhaben

1. Geplanter Beginn der Studie: September 2012 // voraussichtliches Ende: August 2013
Dauer der Studienteilnahme für den einzelnen Probanden:

Intervention (s.u.): 4 Monate // 8 Monate (Kontrollgruppen-Wartelisten-Design)
Untersuchung (s.u.): ca. 100 min jeweils zu Beginn und Studienende

2. Kurzer Abriss des Projektes *(maximal 1,5 Seiten)*:

Einführung und Fragestellung

Krafttraining gilt auch bei intensiver Durchführung als gesundheitsfördernde Intervention, die sich bei einem zunehmenden Anteil der Bevölkerung immer größerer Beliebtheit erfreut. Neben Körperformung und Attraktivität steht für viele Menschen der präventive Aspekt des Muskeltrainings im Vordergrund. Tatsächlich weisen eine Vielzahl von Untersuchungen (1-5) positive Effekte eines regelmäßig durchgeführten, „überschwelligen" Trainings nach, so auf gesundheitsrelevante muskuläre, physiologische, metabolische und kardiovaskuläre Parameter wie beispielsweise die Körperzusammensetzung/funktionelle Kapazität, Blutfette/Lipoproteine oder Glucoseintoleranz/ Insulinsensitivität. Zudem ist anzunehmen, dass auch relativ rasche funktionelle und morphologische Anpassungserscheinungen des Herzens nachweisbar sind (6, 7). Allerdings liegen keine kernspintomographischen Längsschnittuntersuchungen vor, welche die physiologischen Adaptationserscheinungen des Herzens nach einem mehrmonatigen intensiven Krafttraining bei inaktiven Personen evaluieren. Parallel dazu sind die vorliegenden Methoden, mit denen die Körperzusammensetzung und die muskuläre Massenentwicklung bislang evaluiert wurden, als suboptimal einzuschätzen. Um eine valide und reliable Erfassung der Veränderung muskulärer, physiologischer und kardiologischer Größen als Reaktion auf ein gesundheitsorientiertes Muskeltraining zu gewährleisten, ist der messmethodische Schwerpunkt der vorliegenden Untersuchung auf den Einsatz moderner bildgebender Verfahren (Kernspintomographie (MRT), Computertomographie (CT), Dual Energy X-Ray Absorptiometry (DXA)) und moderner Segmentierungs- und Quantifizierungssoftware gerichtet.
Ziel der Untersuchung ist somit die Evaluierung der Effekte eines intensiven 16-wöchigen Krafttrainingsprogramms auf leistungsphysiologische und gesundheitsrelevante muskuläre und kardiale Größen bei untrainierten Männern im mittleren Lebensalter unter besonderer Berücksichtigung bildgebender Verfahren.

Design:

Randomisierte, kontrollierte, teilverblindete Studie mit Wartegruppe (cross-over)

Endpunkte

Primäre Endpunkte:

- Muskelquerschnitt der Oberschenkelmuskulatur (MRT, CT)
- Intraabdominale Fettmasse (MRT)

Sekundäre Endpunkte:

- Kraftfähigkeiten (u.a. abhängige Variable) [1]
- Gesamtkörperfett und Muskelmasse sowie regionale Verteilung (DXA, MRT)
- Metabolisches Syndrom-Score (8), und 10 Jahres-CHD-Risiko (9)
- Kardiale Masse und enddiastolisches Volumen (MRT)
- Hormonelle Regulation (u.a. freies Testosteron, hGH, Cortisol)
- Schmerzintensität, Quality of Life

[1] Ein Ziel der Studie ist die Identifikation von Variablen, welche die Varianz des Kraft-/Leistungszuwachs am höchsten aufklären.

Stichprobe

Zwei-Gruppen Design, randomisiert, z.T. cross-over (mit Wartegruppe)[2]

- Gruppe 1, n = 40: High Intensity Resistance Training (HIT)[3]
- Gruppe 2, n = 40: Kontrollgruppe (Wartegruppe; im Anschluss „Power-Training[4]“)

Interventionsprogramm

Gruppe 1: HIT mit konventioneller Bewegungsgeschwindigkeit (TUT[5] 2s-1s-2s)

Periodisiertes, progressives Krafttraining über 16 Wochen, basierend auf individuellen Trainingsplänen auf der Basis von 1 RM/x RM-Tests (1, 4, 10 Wochen), zunehmende Intensivierung der Reizhöhe (bis Woche 8), anschließend periodisiertes HIT (70-92,5%, 1RM) mit 2-3 Trainingseinheiten je Woche (1-2 gemeinsame, überwachte Trainingseinheiten; 1-2 Trainingseinheiten in Eigenregie (Vorgaben über Trainingsplan)).

Gruppe 2 (ehemalige Kontrollgruppe): Power-Training (TUT: ⚡-1s-2s)

s.o. aber explosive Ausführung im konzentrischen Bewegungsbereich nach initialer Konditionierung über 6 Wochen. Vergleichbares Trainingsprotokoll, allerdings im Intensitätsbereich von 40-70%, 1RM.

3. Studienbezogene Maßnahmen:

Bitte beschreiben Sie hier alle Maßnahmen, die studienbedingt durchgeführt werden sowie alle erforderlichen Abweichungen von der üblichen Routine-Behandlung:

Interventionsprogramm s.o.

Messungen (jeweils basal und nach 16 Wochen)

Bildgebende Verfahren:

Ganzkörper-MRT und Ganzkörper-DXA zur Erfassung der gesamten und regionalen Körperzusammensetzung.

Lokale MRT und CT am Oberschenkel (über „Muskelbauch“)

Kardiale MRT-Untersuchung mit Kontrastmittel i.v. (Analyse fibrotischer Herzmuskelveränderungen, Strain der Herzmuskulatur, enddiastolisches Volumen (EDV), endsystolisches Volumen (ESV), Schlagvolumen (SV), Ejektionsfraktion (EF) und myokardiale Masse (MM), Herzmuskeldicke).

Blutdruck und Herzfrequenzverhalten in Ruhe

Körperliche Fitness:

Erfassung unterschiedlicher Kraftfähigkeiten (u.a. 1 RM, Schnellkraft) mittels isokinetischem Dynamometer.

Psychosoziale Parameter

Befindlichkeit und Schmerzparameter, QoL (Fragebogen)

Labor

Blutfette/Lipoproteine, Glukose, Insulin, HbAC1, Entzündungsmarker

Testosteron, freies Testosteron, hGH, Cortisol; (evt. noch Parameter des Immunsystems)

Ernährungsanalyse

Analyse über 4 Tage (standardisierte Protokolle)

Anamnese und Risikofaktorenprofil über Fragebogen

---

[2] Die Wartegruppe dient zunächst als parallele Kontrollgruppe zur Interventionsgruppe. Nach Abschluss dieses Untersuchungsabschnittes wird mit dieser Gruppe ebenfalls eine 16-wöchige Intervention durchgeführt, sodass ein eingeschränktes 3-Gruppendesign generiert wird.

[3] Training mit relativ hoher Reizintensität (≥70% des Einwiederholungsmaximums: 1RM)

[4] Training mit explosiver Bewegungsausführung im konzentrischen Bereich (bei vglw. geringer Reizintensität im Bereich 40-60% 1RM)

[5] **T**ime **U**nder **T**ension: Dauer der jeweiligen Bewegungsabschnitte, konzentrisch – isometrisch – exzentrisch in Sec.

4. Wird die Studie gemäß der von der 48. Generalversammlung des Weltärztebundes in Somerset West revidierten Deklaration von Helsinki aus dem Jahre 1996 durchgeführt? Bitte angeben, ob alle anderen Erprobungsmöglichkeiten ausgeschöpft wurden.

Ja, die Intervention wurde in vorhergehenden Studien bereits validiert und optimiert. Die Messtechnologie ist ebenfalls etabliert, wurde aber bislang nur suboptimal zur Validierung der hier genannten Fragestellung eingesetzt.

5. Art des Forschungsvorhabens:
Handelt es sich um
eine diagnostische Prüfung?
eine therapeutische Prüfung?
eine Verträglichkeitsprüfung?
☒ einen ausschließlich wissenschaftlichen Versuch?

6. Gesetzliche Grundlagen

a) Handelt es sich um eine Untersuchung, die dazu bestimmt ist, klinische oder pharmakologische Wirkungen von Arzneimitteln zu erforschen oder nachzuweisen oder Nebenwirkungen festzustellen oder die Resorption, die Verteilung, den Stoffwechsel oder die Ausscheidung zu untersuchen, **mit dem Ziel, sich von der Unbedenklichkeit oder Wirksamkeit des Arzneimittels zu überzeugen** (klinische Prüfung eines Arzneimittels nach §§ 40 Arzneimittelgesetz)?

nein

Bitte begründen. Erläuterungen zum Antrag auf Bewertung einer klinischen Arzneimittelprüfung nach § 40 AMG finden Sie unter http://www.ethik.med.uni-erlangen.de/

b) Handelt es sich um eine klinische Prüfung nach § 20 Medizinproduktegesetz (MPG)?

ja ☒ nein

Bitte begründen. Liegt eine CE-Zertifizierung für das Medizinprodukt vor? Werden zusätzlich invasive oder andere belastende Untersuchungen durchgeführt?
Sämtliche Messverfahren sind CE-zertifiziert und entsprechend MPG geprüft und zugelassen. Invasive Untersuchungen werden nicht durchgeführt, mit Ausnahme von Blutentnahmen und einer venösen Kontrastmittelapplikation.

c) Handelt es sich um ein Vorhaben nach § 8 des Gesetzes zur Regelung des Transfusionswesens (TFG)?

ja ☒ nein

7. Handelt es sich um einen Versuch nach

§ 23 Strahlenschutzverordnung? ☒ ja nein

§ 28 Röntgenverordnung? ☒ ja nein

8. Typ der Studie:

- offen
- ☒ blind
- doppelblind
- ☒ vergleichend
- ☒ randomisiert
- multizentrisch
- ☒ Feldstudie
- Pilotstudie

9. Wissenschaftliche Begründung des Projekts, insbesondere:

a. Erläuterung des Versuchsziels

Das Ziel der Untersuchung ist die Evaluierung eines intensiven körperlichen Krafttrainings auf leistungsphysiologische und gesundheitsrelevante muskuläre und kardiale Größen bei untrainierten Männern im mittleren Lebensalter unter besonderer Berücksichtigung moderner bildgebender Verfahren. Aus radiologischer Sicht ist das Ziele der Studie die Identifikation typischer Kenngrößen der physiologischen Adaption der Körperzusammensetzung und des Herz-Kreislauf-Systems nach unterschiedlichen Typen von Krafttraining (s.o.) sowie der Methodenvergleich kernspintomographischer mit computertomographischen Muskelquerschnitten des Oberschenkels sowie der gesamten und der regionalen Gesamtkörperzusammensetzung mittels MRT vs. dem Goldstandard DXA-Methode.

b. Darstellung des bisherigen Wissensstandes

Eine Vielzahl von wissenschaftlichen Untersuchungen berichten den positiven Effekt eines „Krafttrainings“ auf funktionelle und gesundheitsrelevante physiologische und metabolische Größen (Übersicht in (1, 3, 10, 11)) bei Menschen in mittlerem Lebensalter. Obgleich viele dieser Daten bereits in frühen Studien mit suboptimaler Messmethodik/-technik und schlechter Reproduzierbarkeit evaluiert wurden, gelten sie in der wissenschaftlichen Literatur als absolut verlässlich und werden vielfach zitiert. Moderne bildgebende Verfahren wie die Kernspintomographie (MRT) oder die Computertomographie (CT) im Verbund mit valider Segmentierungstechnologie und quantitativer Analyse haben die in der Vergangenheit eingesetzten Verfahren mit suboptimaler Auflösung und ausschließlich qualitativer Auswerteprozedur bereits in vielen Bereichen ersetzt. So können Risikofaktoren wie die Körperfettverteilung derzeit schon quantifiziert und der Einfluss einer Intervention validiert werden (12-14). Auch die muskuläre Komponente der Körperzusammensetzung und insbesondere der Muskelquerschnitt wurde von einigen neueren Untersuchungen mittels moderner bildgebender Verfahren wie MRT oder CT untersucht (15, 16), die allerdings auf keine validierte Segmentierungs- und Quantifizierungssoftware zurückgreifen konnten.

Dies trifft ebenfalls für die kardiale Volumetrie mittels MRT zu, die derzeit als Goldstandard für die links- und rechtsventrikuläre Volumen- und Massenbestimmung gilt (17). Im Gegensatz zu echokardiographischen Untersuchungen handelt es sich bei der kardialen MRT um ein dreidimensionales Verfahren, welches dadurch eine wesentlich genauere morphologische Darstellung des Herzens ermöglicht (18) und somit u.a.

interventionsbedingte Veränderungen früher zu identifizieren vermag. Darüber hinaus werden sportliche Belastungen immer wieder mit einem plötzlichen Herztod, insbesondere bei männlichen Athleten, assoziiert (23-24). Ob hierfür pathologische morphologische Veränderungen des Myokards durch körperliches Training oder angeborene Organerkrankungen zu Grunde liegen, ist unklar.

10. Angaben zur Nutzen-Risiko-Relation

a. Welcher Nutzen ist von den Ergebnissen der Studie zu erwarten

aa) für die Versuchsteilnehmer?

Steigerung der körperlichen Fitness und physischen Attraktivität durch das Trainingsprogramm. Zudem Verminderung des metabolischen und kardiovaskulären Gesundheitsrisikos und Verbesserung der funktionellen Kapazität. Ggf. Detektion relevanter kardiologischer Befunde (Herzklappendefekte, Kardiomyopathie, Fehlbildungen).

ab) für die Heilkunde?

Beschreibung physiologischer muskulärer, metabolischer, physiologischer und kardialer Adaptation nach intensivem Krafttraining u.a. als Voraussetzung zur Abgrenzung pathologischer Veränderungen und Identifizierung geeigneter Verfahren zur Kontrolle von Trainingseffekten. Sekundäres Ziel ist es, Risikopatienten zu identifizieren, die vor Aufnahme eines spezifischen Trainings ein ausführliches Untersuchungsprogramm durchführen sollten, um Sekundärschäden zur vermeiden.

ac) für die Wissenschaft (z.B. Ergebnisse, die nicht unmittelbar therapeutischen Zwecken dienen)?

Verbesserter Einblick in die Grundlagen trainingsinduzierter muskulärer und kardiovaskulärer Adaptation als Basis optimaler sportwissenschaftlicher Trainings-empfehlungen. Evaluierung und Weiterentwicklung von bildgebenden Untersuchungs-verfahren und computerunterstützter Evaluationssoftware zur Diagnostik und zum Monitoring von Interventionseffekten.

b. Mit welchem Risiko ist die Studie für die Versuchsteilnehmer verbunden?

ba) Welcher Art sind die Risiken? Risikoeinschätzung, vorhersehbare Risiken der Behandlung und sonstiger studienbedingter Verfahren, die eingesetzt werden sollen (einschließlich Schmerz, Unannehmlichkeiten, Beschwerden, Verletzung der persönlichen Integrität und Maßnahmen zur Vermeidung und/oder zur Behandlung von unvorhersehbaren/ unerwünschten Ereignissen)

Die Risiken der Intervention (konsequent angeleitetes Krafttrainingsprogramm) sind sehr gering und bleiben nicht zuletzt aufgrund der intensiven Betreuung deutlich hinten denen eines selbständig durchgeführten Muskeltrainings zurück. Selbstverständlich sind in den ersten Wochen der Intervention leichte trainingsbedingte Beschwerden wie bspw. DOMS (Muskelkater) zu erwarten.

Die radiologischen Verfahren Dual Energy X-Ray Absorptiometrie (Gesamtkörper-DXA) bzw. Computertomographie (medialer Anteil Oberschenkel) sind mit niedrigen Strahlendosen (< 10 µSv pro DXA-Messung) bzw. geringen relativen Dosen (<0.8 mSv/CT-Messung[6]) verbunden. Eine Genehmigung dieser Verfahren

---

[6] Dieser Wert wurde ohne Berücksichtigung entsprechender Schutzmaßnahmen (Abdecken der Gonaden mit Bleischutz errechnet.

wird beim Bundesamt für Strahlenschutz nach Vorliegen der Genehmigung der Ethikkommission selbstverständlich beantragt.

Bei der Blutentnahme und durch periphere Verweilkatheter für die Kontrastmittel-(KM)-Applikation sind Blutergüsse und Infektionen nie komplett ausgeschlossen. In der Kernspintomographie werden nur geringe KM-Mengen eingesetzt. Daher sind die Risiken bei einem Paravasat des KMs in das Weichteilgewebe sowie Nierenbelastungen und mögliche allergische KM-Reaktionen minimiert. Probanden mit relativen oder absoluten Kontraindikationen für eine MRT werden von der Studie ausgeschlossen (s.u.). Insbesondere Probanden mit Nierenfunktionsstörungen oder bekannten KM-Reaktionen. Die KM-Gabe ist notwendig, um relevante kardiale Vorerkrankungen auszuschließen (siehe Ausschlusskriterien), die ein Risiko für die Intervention darstellen können.

bb) Mit welcher Wahrscheinlichkeit ist zu erwarten, daß sich die Risiken realisieren? Wie sicher ist die Wahrscheinlichkeit abschätzbar?

Insgesamt besteht nur eine geringe Wahrscheinlichkeit, dass sich die Risiken realisieren. Die Messungen stellen Standardmessungen der klinischen Routine dar, die von geschultem Fachpersonal sachkundig ausgeführt werden. Die Belastungsvorgaben erfolgen individuell, basierend auf einer Leistungsdiagnostik, sodass eine Überforderung der Teilnehmer kaum zu erwarten ist.

c. Warum ist das mögliche Risiko im Verhältnis zu dem zu erwartenden Nutzen Ihrer Ansicht nach vertretbar?

Es besteht keine wesentlich über das Alltagsrisiko hinausgehende Gefährdung, jedoch ein hoher zu erwartender Nutzen für die Teilnehmer hinsichtlich der Steigerung der körperlichen Fitness, gesundheitsrelevanter Größen, Wohlbefinden, Attraktivität und Selbstwirksamkeit/Kontrollüberzeugung.

d. Werden Zwischenergebnisse ausgewertet, um einen Trend zu erkennen?
ja ☒ nein, kurzer Interventionszeitraum macht Zwischenanalyse inadäquat.

e. Sind Kriterien festgelegt worden, bei deren Eintreten der Versuch geändert oder abgebrochen werden soll? ja, welche? ☒ nein

(allerdings Abbruch des jeweiligen Testverfahrens bei Unwohlsein, oder generell auf Wunsch des Patienten)

11. Bei klinischen Prüfungen nach MPG:

a. Welches Medizinprodukt soll geprüft werden? **entfällt**

b. Wird die klinische Prüfung von einer entsprechend qualifizierten und befugten Person geleitet, die mindestens eine zweijährige Erfahrung in der klinischen Prüfung von Medizinprodukten nachweisen kann? ja nein

c. Wurde (soweit erforderlich) eine dem jeweiligen Stand der wissenschaftlichen Erkenntnisse entsprechende biologische Sicherheitsprüfung oder sonstige für die vorgesehene Zweckbestimmung des Medizinproduktes erforderliche Prüfung durchgeführt? ja nein

d. Wurde (soweit erforderlich) die sicherheitstechnische Unbedenklichkeit für die Anwendung des Medizinproduktes unter Berücksichtigung des Standes der Technik sowie der Arbeitsschutz- und Unfallverhütungsvorschriften nachgewiesen?
ja nein

e. Ist der Leiter der klinischen Prüfung über die Ergebnisse der biologischen Sicherheitsprüfung und die voraussichtlich mit der klinischen Prüfung verbundenen Risiken informiert worden? ja nein

12. a) Ist die Mitarbeit eines Statistikers vorgesehen? ☒ ja nein
b) Welche statistischen Methoden sollen benutzt werden?

Komplettes statistisches Instrumentarium zur Erfassung von Effekten (bspw. Varianzanalysen/nicht parametrische Tests zur Identifikation von Zwischengruppenunterschieden). Zudem regressionsanalytische Modelle zur Aufklärung von Varianzen.

13. a) Handelt es sich um eine multizentrische Studie (d.h. eine nach einem *einzigen* Prüfplan durchgeführte Studie, die in mehr als einer Prüfstelle erfolgt und daher von mehr als einem Prüfer vorgenommen wird)? ja ☒ nein

b) Wurden/Werden an anderer Stelle Studien mit demselben oder einem ähnlichen Ziel durchgeführt? ja, wo? ☒ nein

Es wurden in der Vergangenheit bereits mehrere Untersuchungen mit dem Ziel der Evaluierung eines gesundheitssportlichen Trainings auf muskuläre, physiologische, metabolische und kardiale Parameter (meist isoliert) durchgeführt (s.o.). Diese Studie ist jedoch die erste, die u.a. den Effekt eines intensiven Kraft-/Powertrainings auf muskuläre Parameter, Körperzusammensetzung, metabolische und kardiovaskuläre Größen bei untrainierten Männern in mittlerem Lebensalter auch mittels moderner bildgebender Verfahren und Auswertesoftware evaluiert.

14. Wer hat die Studie initiiert? **Institut für Medizinische Physik**

15. Wer finanziert sie? (*Bitte geben Sie an, ob Drittmittel von nichtöffentlicher Seite beantragt werden. Falls ja, in welcher Höhe?*)

Derzeit sind noch keine Mittel beantragt, es werden jedoch nach positivem Votum der Ethikkommission versucht über unterschiedliche Ebenen Drittmittel zu generieren.

16. Die Aufwandsentschädigung wird übernommen von (*bitte Ansprechpartner benennen*):

Institut für Medizinische Physik (Direktor: Prof. Dr. Dr. W.A. Kalender)

Ansprechpartner: Prof. Dr. Wolfgang Kemmler

III. Angaben zu den Versuchsteilnehmern

1. Anzahl (*bei vergleichenden Studien bitte Aufteilung auf Gruppen angeben*)

80 Personen gesamt:
- Gruppe 1, n = 40: High Intensity Resistance Training (HIT)[7]
- Gruppe 2, n = 40: Kontrollgruppe (Wartegruppe; im Anschluss „Power-Training“)

Bei Nullhypothesen-basierten Studien:
Wurde eine formale Fallzahlschätzung vorgenommen?

☒ ja, Basis CSA-Oberschenkelmuskulatur nein

2. Alter und Geschlecht (*bitte geben Sie das Alter der Versuchsteilnehmer sowie die als Ausschlusskriterien vorgesehenen Ober- und Untergrenzen an*)

[7] Training mit relativ hoher Reizintensität (≥70% des Einwiederholungsmaximums: 1RM)

Männer, 30. - 50. Lebensjahr

3. Status: Handelt es sich bei den Versuchsteilnehmern um
   ☒ gesunde Personen
   schwangere oder stillende Frauen
   Kinder oder Jugendliche
   einschlägig Erkrankte (*bitte geben Sie die Krankheit und das Stadium an*)
   Personen, die an anderen Krankheiten leiden? (Insbesondere: psychische Krankheiten, die Zweifel an der Geschäfts- oder Einsichtsfähigkeit begründen)

4. Welche sonstigen **Einschlusskriterien** (z.B. erlaubte Begleitmedikation) sind vorgesehen?

   - initial Untrainierte (während der vergangenen 2 Jahre: ≤1 h/Woche Sport mit positivem Effekt auf die Muskulatur; ≤2 h/Woche Sport gesamt)

5. Welche sonstigen **Ausschlusskriterien** (z.B. fortgeschrittene Nieren- oder Leber-insuffizienz, verbotene Begleitmedikation etc.) sind vorgesehen?
   - Geschichte leistungssportlicher Ausübung von Disziplinen mit erheblicher Relevanz für Körperzusammensetzung und Kraftfähigkeiten
   - pathologische muskuläre, metabolische und kardiale Veränderungen oder Entzündungen; deutlich eingeschränkte Gelenksbeweglichkeit in Knie und Hüfte.
   - Medikamente/Erkrankungen mit relevantem Einfluss auf Körperzusammensetzung und Herz-Kreislauf-System
   - sehr geringe körperliche Leistungsfähigkeit (< 100 Watt auf dem Fahrradergometer)
   - schwere Adipositas (BMI > 35 kg/m^2)
   - Abwesenheit ≥ 2 Wochen während des Interventionszeitraums
   - Geplante Aufnahme einer relevanten parallelen Trainingsmaßnahme
   - Kontraindikationen gegen MRT (Klaustrophobie, Herzschrittmacher, magnetisier-bare intracorporale Fremdkörper); Körpermaße die eine MRT-Messung verhindern
   - Drogenmissbrauch

6. Sollen auch Personen teilnehmen, die auf gerichtliche oder behördliche Anordnung in einer Anstalt verwahrt werden?
   ja ☒ nein

7. Sollen auch Personen teilnehmen, die sich schon für andere Forschungsvorhaben zur Verfügung gestellt haben?
   ja ☒ nein
   wie lange muss die letzte Teilnahme zurückliegen?

8. Bei Studien an Minderjährigen (oder sonst nicht geschäftsfähigen Personen) **entfällt**
   a. Warum kann die Studie nicht an Erwachsenen (voll Geschäftsfähigen) durchgeführt werden?

   b. Sind Aufklärung und Einwilligung der (des) gesetzlichen Vertreter(s) gewährleistet? (*bitte vorformulierte Erklärung beifügen*)
   ja nein, weil

c. Sind zusätzliche Aufklärung und Einwilligung der minderjährigen (nicht voll geschäftsfähigen) Versuchsteilnehmer gewährleistet, die selbst in der Lage sind, Wesen, Bedeutung und Tragweite des Versuchs einzusehen und ihren Willen danach zu bestimmen?

ja nein

9. Probandenversicherung

Wird zugunsten der Versuchsteilnehmer eine Versicherung abgeschlossen?

ja (*bitte Police beifügen, aus der die Versicherungsgesellschaft und die Höhe der Versicherungsleistung hervorgeht)*

☒ nein

10. Schweigepflicht/Datenschutz

Werden die ärztlichen Schweigepflicht- und die Datenschutzbestimmungen beachtet?

ja

11. Entgelt für Probanden

Soll den Versuchsteilnehmern ein Entgelt (Aufwandsentschädigung o.ä.) gezahlt werden?

ja, in Höhe von EUR ☒ nein

12. Wie sollen die Versuchsteilnehmer über Wesen, Bedeutung und Tragweite der Studie **aufgeklärt** werden?

Bitte in deutscher Sprache beifügen:

Dokumentation des Inhalts der Patientenaufklärung durch die/den versuchsdurchführende/n Ärztin/Arzt (Merkblatt), insbesondere mit Hinweisen über:

- **Ziele und Methoden** der Studie;
- **Nutzen und Risiko** der Studie;
- bekannte und möglicherweise zu erwartende **Wirkungen und Nebenwirkungen** von Medikamenten;
- Eingriffe, die nur aus wissenschaftlichen Gründen erfolgen;
- ein angebrachtes Verhalten des Patienten während und nach dem Versuch;
- die **Widerruflichkeit** einer Einwilligung;
- das Bestehen und den Umfang der gesetzlichen **Probandenversicherung** (Name/Anschrift/Telefon/Fax der Versicherungsgesellschaft, Nummer der Versicherungspolice) sowie die danach von der Versuchsperson zu beachtenden Obliegenheiten;
- **Ausschlusskriterien** (z.B. Schwangerschaft/Stillzeit);
- Name und Telefon des **Ansprechpartner**s vor Ort.
- **Besondere Aufklärung** über die Situation
  a. bei der randomisierten Studie
  f. beim Blind- und Doppelblindversuch.

13. Wie sollen die Versuchsteilnehmer ihre **Einwilligung** in die Teilnahme an der Studie erklären? (*bitte formulierte deutschsprachige Erklärung mit datenschutzrechtlicher Einwilligungserklärung beifügen*)

Schriftliche Einverständniserklärung nach umfangreicher mündlicher und schriftlicher Aufklärung über Ziele, Nutzen und Risiken der Untersuchung.

Ich weiß, daß auch bei einer positiven Beurteilung des Vorhabens durch die Ethik-Kommission der Medizinischen Fakultät der FAU Erlangen-Nürnberg die ärztliche und juristische Verantwortung für die Durchführung des Projektes uneingeschränkt bei der Leiterin/dem Leiter verbleibt.

Erlangen/Nürnberg
Datum ....

Unterschrift des/der Antragstellers/in

____

(Name in Druckbuchstaben)

Unterschrift der/des Leiterin/Leiters der Einrichtung, in der das Vorhaben durchgeführt werden soll.
Mit der Durchführung des Forschungsvorhabens einverstanden:

Datum ....

Unterschrift des/der Leiters/Leiterin der Einrichtung

____

(Name in Druckbuchstaben)

EK_May09/mit Unterschriftsblatt

Literatur

1. Benson AC, Torode ME, Fiatarone Singh MA. Effects of resistance training on metabolic fitness in children and adolescents: a systematic review. *Obes Rev*. 2008;9(1):43-66.
2. Kelley GA, Kelley KS. Impact of progressive resistance training on lipids and lipoproteins in adults: a meta-analysis of randomized controlled trials. *Prev Med*. 2009;48(1):9-19.
3. Latham NK, Bennett DA, Stretton CM, Anderson CS. Systematic review of progressive resistance strength training in older adults. *J Gerontol A Biol Sci Med Sci*. 2004;59(1):48-61.
4. Macaluso A, De Vito G. Muscle strength, power and adaptations to resistance training in older people. *Eur J Appl Physiol*. 2004;91:450-472.
5. Snowling NJ, Hopkins WG. Effects of different modes of exercise training on glucose control and risk factors for complications in type 2 diabetic patients: a meta-analysis. *Diabetes Care*. 2006;29(11):2518-27.
6. Weineck J. *Optimales Training* Erlangen: Spitta-Verlag; 2007.
7. Weineck J. *Sportbiologie*. Vol. 10 Balingen: Spitta Verlag; 2009.
8. Wijndaele K, Beunen G, Duvigneaud N, et al. A continuous metabolic syndrome risk score: utility for epidemiological analyses. *Diabetes Care*. 2006;29(10):2329.
9. Wilson PW, D'Agostino RB, Levy D, Belanger AM, Silbershatz H, Kannel WB. Prediction of coronary heart disease using risk factor categories. *Circulation*. 1998;97(18):1837-47.
10. Asikainen TM, Kukkonen-Harjula K, Miilunpalo S. Exercise for health for early postmenopausal women: a systematic review of randomised controlled trials. *Sports Med*. 2004;34(11):753-78.
11. Lagally KM, Cordero J, Good J, Brown DD, McCaw ST. Physiologic and metabolic responses to a continuous functional resistance exercise workout. *J Strength Cond Res*. 2009;23(2):373-9.
12. Kay SJ, Fiatarone Singh MA. The influence of physical activity on abdominal fat: a systematic review of the literature. *Obes Rev*. 2006;7(2):183-200.
13. Kemmler W, von Stengel S, Engelke K, Haberle L, Mayhew JL, Kalender WA. Exercise, body composition, and functional ability: a randomized controlled trial. *Am J Prev Med*. 2010;38(3):279-87.
14. Lamb HJ. Total body fat distribution as part of multiorgan MR imaging: new tool for risk assessment in the metabolic syndrome? *Radiology*. 2010;257(2):307-8.
15. Weiss EP, Racette SB, Villareal DT, et al. Lower extremity muscle size and strength and aerobic capacity decrease with caloric restriction but not with exercise-induced weight loss. *J Appl Physiol*. 2007;102(2):634-40.
16. Valtonen A, Poyhonen T, Sipila S, Heinonen A. Effects of aquatic resistance training on mobility limitation and lower-limb impairments after knee replacement. *Arch Phys Med Rehabil*;91(6):833-9.
17. Petersen SE, Hudsmith LE, Robson MD, et al. Sex-specific characteristics of cardiac function, geometry, and mass in young adult elite athletes. *J Magn Reson Imaging*. 2006;24(2):297-303.
18. Grothues F, Smith GC, Moon JC, et al. Comparison of interstudy reproducibility of cardiovascular magnetic resonance with two-dimensional echocardiography in normal subjects and in patients with heart failure or left ventricular hypertrophy. *Am J Cardiol*. 2002;90(1):29-34.

An die Geschäftsstelle der
Ethik-Kommission
der Medizinischen Fakultät
der FAU Erlangen-Nürnberg
Krankenhausstr. 12
91054 Erlangen

Antrag
an die Ethik-Kommission
der Medizinischen Fakultät

*Bitte **in deutscher Sprache** ausfüllen,*
Zutreffendes bitte ankreuzen.
Für multizentrische Studien mit Vorvotum einer nach Landesrecht gebildeten zuständigen Ethik-Kommission können Sie das verkürzte Antragsformular verwenden, abzurufen unter: http://www.ethik.med.uni-erlangen.de *(Anschlussvotum)*

Antrag auf Beurteilung eines Forschungsprojektes (keine Arzneimittelprüfung)

bitte 9-fach einschließlich Anlagen einreichen sowie 1-mal in elektronischer Fassung

**Titel des Projektes:**<br>Einfluss eines 16-wöchigen Kraft-Trainingsprogramms auf leistungsphysiologische und gesundheitsrelevante muskuläre und kardiale Größen bei untrainierten Männern im mittleren Lebensalter. Eine randomisierte kontrollierte Interventionsstudie mit modernen, bildgebenden Verfahren.

I. Projektleitung

1. Name der/des verantwortlichen Projektleiterin/s an der FAU:
Professor Dr. Wolfgang Kemmler[1], Osteoporose-Forschungszentrum, Institut für Medizinische Physik, FAU (Direktor: Professor Dr. Dr. Willi A. Kalender)
App.-Nr. 23999; E-Mail: wolfgang.kemmler@imp.uni-erlangen.de

Angaben über die Qualifikation der/des Versuchsleiterin/s:
Promotion, Habilitation (liegen bereits vor)

2. a) Weitere Teilnehmer/innen vor Ort (alphabetische Reihenfolge):
Prof. Dr. Klaus Engelke, IMP, FAU
PD. Dr. Michael Lell, Radiologisches Institut, FAU
Prof. Dr. Harald Quick, IMP, FAU
PD. Dr. Axel Schmid, Radiologisches Institut, FAU
Dr. Michael Scharf, Radiologisches Institut, FAU
Dr. Simon von Stengel, Osteoporoseforschungszentrum, FAU
Andreas Wittke, IMP, FAU

b) Weitere Prüfzentren (bei multizentrischen Studien): keine

3. Handelt es sich bei diesem Antrag um ein bereits von der Ethik-Kommission der Medizinischen Fakultät der Friedrich-Alexander-Universität Erlangen-Nürnberg begutachtetes Projekt?

ja (bitte lfd. Nr. angeben) ☒ nein

II. Forschungsvorhaben

1. Geplanter Beginn der Studie: September 2012 // voraussichtliches Ende: August 2013
Dauer der Studienteilnahme für den einzelnen Probanden:

Intervention (s.u.): 4 Monate // 8 Monate (Kontrollgruppen-Wartelisten-Design)
Untersuchung (s.u.): ca. 100 min jeweils zu Beginn und Studienende

2. Kurzer Abriss des Projektes *(maximal 1,5 Seiten)*:

Einführung und Fragestellung

Krafttraining gilt auch bei intensiver Durchführung als gesundheitsfördernde Intervention, die sich bei einem zunehmenden Anteil der Bevölkerung immer größerer Beliebtheit erfreut. Neben Körperformung und Attraktivität steht für viele Menschen der präventive Aspekt des Muskeltrainings im Vordergrund. Tatsächlich weisen eine Vielzahl von Untersuchungen (1-5) positive Effekte eines regelmäßig durchgeführten, „überschwelligen“ Trainings nach, so auf gesundheitsrelevante muskuläre, physiologische, metabolische und kardiovaskuläre Parameter wie beispielsweise die Körperzusammensetzung/funktionelle Kapazität, Blutfette/Lipoproteine oder Glucoseintoleranz/ Insulinsensitivität. Zudem ist anzunehmen, dass auch relativ rasche funktionelle und morphologische Anpassungserscheinungen des Herzens nachweisbar sind (6, 7). Allerdings liegen keine kernspintomographischen Längsschnittuntersuchungen vor, welche die physiologischen Adaptationserscheinungen des Herzens nach einem mehrmonatigen intensiven Krafttraining bei inaktiven Personen evaluieren. Parallel dazu sind die vorliegenden Methoden, mit denen die Körperzusammensetzung und die muskuläre Massenentwicklung bislang evaluiert wurden, als suboptimal einzuschätzen. Um eine valide und reliable Erfassung der Veränderung muskulärer, physiologischer und kardiologischer Größen als Reaktion auf ein gesundheitsorientiertes Muskeltraining zu gewährleisten, ist der messmethodische Schwerpunkt der vorliegenden Untersuchung auf den Einsatz moderner bildgebender Verfahren (Kernspintomographie (MRT), Computertomographie (CT), Dual Energy X-Ray Absorptiometry (DXA)) und moderner Segmentierungs- und Quantifizierungssoftware gerichtet.
Ziel der Untersuchung ist somit die Evaluierung der Effekte eines intensiven 16-wöchigen Krafttrainingsprogramms auf leistungsphysiologische und gesundheitsrelevante muskuläre und kardiale Größen bei untrainierten Männern im mittleren Lebensalter unter besonderer Berücksichtigung bildgebender Verfahren.

Design:

Randomisierte, kontrollierte, teilverblindete Studie mit Wartegruppe (cross-over)

Endpunkte

Primäre Endpunkte:

- Muskelquerschnitt der Oberschenkelmuskulatur (MRT, CT)
- Intraabdominale Fettmasse (MRT)

Sekundäre Endpunkte:

- Kraftfähigkeiten (u.a. abhängige Variable) [1]
- Gesamtkörperfett und Muskelmasse sowie regionale Verteilung (DXA, MRT)
- Metabolisches Syndrom-Score (8), und 10 Jahres-CHD-Risiko (9)
- Kardiale Masse und enddiastolisches Volumen (MRT)
- Hormonelle Regulation (u.a. freies Testosteron, hGH, Cortisol)
- Schmerzintensität, Quality of Life

[1] Ein Ziel der Studie ist die Identifikation von Variablen, welche die Varianz des Kraft-/Leistungszuwachs am höchsten aufklären.

Stichprobe

Zwei-Gruppen Design, randomisiert, z.T. cross-over (mit Wartegruppe)[2]

- Gruppe 1, n = 40: High Intensity Resistance Training (HIT)[3]
- Gruppe 2, n = 40: Kontrollgruppe (Wartegruppe; im Anschluss „Power-Training[4]“)

Interventionsprogramm

Gruppe 1: HIT mit konventioneller Bewegungsgeschwindigkeit (TUT[5] 2s-1s-2s)

Periodisiertes, progressives Krafttraining über 16 Wochen, basierend auf individuellen Trainingsplänen auf der Basis von 1 RM/x RM-Tests (1, 4, 10 Wochen), zunehmende Intensivierung der Reizhöhe (bis Woche 8), anschließend periodisiertes HIT (70-92,5%, 1RM) mit 2-3 Trainingseinheiten je Woche (1-2 gemeinsame, überwachte Trainingseinheiten; 1-2 Trainingseinheiten in Eigenregie (Vorgaben über Trainingsplan)).

Gruppe 2 (ehemalige Kontrollgruppe): Power-Training (TUT: ϟ-1s-2s)

s.o. aber explosive Ausführung im konzentrischen Bewegungsbereich nach initialer Konditionierung über 6 Wochen. Vergleichbares Trainingsprotokoll, allerdings im Intensitätsbereich von 40-70%, 1RM.

3. Studienbezogene Maßnahmen:

Bitte beschreiben Sie hier alle Maßnahmen, die studienbedingt durchgeführt werden sowie alle erforderlichen Abweichungen von der üblichen Routine-Behandlung:

Interventionsprogramm s.o.

Messungen (jeweils basal und nach 16 Wochen)

Bildgebende Verfahren:

Ganzkörper-MRT und Ganzkörper-DXA zur Erfassung der gesamten und regionalen Körperzusammensetzung.

Lokale MRT und CT am Oberschenkel (über „Muskelbauch“)

Kardiale MRT-Untersuchung mit Kontrastmittel i.v. (Analyse fibrotischer Herzmuskelveränderungen, Strain der Herzmuskulatur, enddiastolisches Volumen (EDV), endsystolisches Volumen (ESV), Schlagvolumen (SV), Ejektionsfraktion (EF) und myokardiale Masse (MM), Herzmuskeldicke).

Blutdruck und Herzfrequenzverhalten in Ruhe

Körperliche Fitness:

Erfassung unterschiedlicher Kraftfähigkeiten (u.a. 1 RM, Schnellkraft) mittels isokinetischem Dynamometer.

Psychosoziale Parameter

Befindlichkeit und Schmerzparameter, QoL (Fragebogen)

Labor

Blutfette/Lipoproteine, Glukose, Insulin, HbAC1, Entzündungsmarker

Testosteron, freies Testosteron, hGH, Cortisol; (evt. noch Parameter des Immunsystems)

Ernährungsanalyse

Analyse über 4 Tage (standardisierte Protokolle)

Anamnese und Risikofaktorenprofil über Fragebogen

---

[2] Die Wartegruppe dient zunächst als parallele Kontrollgruppe zur Interventionsgruppe. Nach Abschluss dieses Untersuchungsabschnittes wird mit dieser Gruppe ebenfalls eine 16-wöchige Intervention durchgeführt, sodass ein eingeschränktes 3-Gruppendesign generiert wird.

[3] Training mit relativ hoher Reizintensität (≥70% des Einwiederholungsmaximums: 1RM)

[4] Training mit explosiver Bewegungsausführung im konzentrischen Bereich (bei vglw. geringer Reizintensität im Bereich 40-60% 1RM)

[5] **T**ime **U**nder **T**ension: Dauer der jeweiligen Bewegungsabschnitte, konzentrisch – isometrisch – exzentrisch in Sec.

4. Wird die Studie gemäß der von der 48. Generalversammlung des Weltärztebundes in Somerset West revidierten Deklaration von Helsinki aus dem Jahre 1996 durchgeführt? Bitte angeben, ob alle anderen Erprobungsmöglichkeiten ausgeschöpft wurden.

Ja, die Intervention wurde in vorhergehenden Studien bereits validiert und optimiert. Die Messtechnologie ist ebenfalls etabliert, wurde aber bislang nur suboptimal zur Validierung der hier genannten Fragestellung eingesetzt.

5. Art des Forschungsvorhabens:
Handelt es sich um
- eine diagnostische Prüfung?
- eine therapeutische Prüfung?
- eine Verträglichkeitsprüfung?
- ☒ einen ausschließlich wissenschaftlichen Versuch?

6. Gesetzliche Grundlagen

a) Handelt es sich um eine Untersuchung, die dazu bestimmt ist, klinische oder pharmakologische Wirkungen von Arzneimitteln zu erforschen oder nachzuweisen oder Nebenwirkungen festzustellen oder die Resorption, die Verteilung, den Stoffwechsel oder die Ausscheidung zu untersuchen, **mit dem Ziel, sich von der Unbedenklichkeit oder Wirksamkeit des Arzneimittels zu überzeugen** (klinische Prüfung eines Arzneimittels nach §§ 40 Arzneimittelgesetz)?

nein

Bitte begründen. Erläuterungen zum Antrag auf Bewertung einer klinischen Arzneimittelprüfung nach § 40 AMG finden Sie unter http://www.ethik.med.uni-erlangen.de/

b) Handelt es sich um eine klinische Prüfung nach § 20 Medizinproduktegesetz (MPG)?

ja ☒ nein

Bitte begründen. Liegt eine CE-Zertifizierung für das Medizinprodukt vor? Werden zusätzlich invasive oder andere belastende Untersuchungen durchgeführt?
Sämtliche Messverfahren sind CE-zertifiziert und entsprechend MPG geprüft und zugelassen. Invasive Untersuchungen werden nicht durchgeführt, mit Ausnahme von Blutentnahmen und einer venösen Kontrastmittelapplikation.

c) Handelt es sich um ein Vorhaben nach § 8 des Gesetzes zur Regelung des Transfusionswesens (TFG)?

ja ☒ nein

7. Handelt es sich um einen Versuch nach

§ 23 Strahlenschutzverordnung? ☒ ja nein

§ 28 Röntgenverordnung? ☒ ja nein

8. Typ der Studie:

- offen
- ☒ blind
- doppelblind
- ☒ vergleichend
- ☒ randomisiert
- multizentrisch
- ☒ Feldstudie
- Pilotstudie

9. Wissenschaftliche Begründung des Projekts, insbesondere:

a. Erläuterung des Versuchsziels

Das Ziel der Untersuchung ist die Evaluierung eines intensiven körperlichen Krafttrainings auf leistungsphysiologische und gesundheitsrelevante muskuläre und kardiale Größen bei untrainierten Männern im mittleren Lebensalter unter besonderer Berücksichtigung moderner bildgebender Verfahren. Aus radiologischer Sicht ist das Ziele der Studie die Identifikation typischer Kenngrößen der physiologischen Adaption der Körperzusammensetzung und des Herz-Kreislauf-Systems nach unterschiedlichen Typen von Krafttraining (s.o.) sowie der Methodenvergleich kernspintomographischer mit computertomographischen Muskelquerschnitten des Oberschenkels sowie der gesamten und der regionalen Gesamtkörperzusammensetzung mittels MRT vs. dem Goldstandard DXA-Methode.

b. Darstellung des bisherigen Wissensstandes

Eine Vielzahl von wissenschaftlichen Untersuchungen berichten den positiven Effekt eines „Krafttrainings“ auf funktionelle und gesundheitsrelevante physiologische und metabolische Größen (Übersicht in (1, 3, 10, 11)) bei Menschen in mittlerem Lebensalter. Obgleich viele dieser Daten bereits in frühen Studien mit suboptimaler Messmethodik/-technik und schlechter Reproduzierbarkeit evaluiert wurden, gelten sie in der wissenschaftlichen Literatur als absolut verlässlich und werden vielfach zitiert. Moderne bildgebende Verfahren wie die Kernspintomographie (MRT) oder die Computertomographie (CT) im Verbund mit valider Segmentierungstechnologie und quantitativer Analyse haben die in der Vergangenheit eingesetzten Verfahren mit suboptimaler Auflösung und ausschließlich qualitativer Auswerteprozedur bereits in vielen Bereichen ersetzt. So können Risikofaktoren wie die Körperfettverteilung derzeit schon quantifiziert und der Einfluss einer Intervention validiert werden (12-14). Auch die muskuläre Komponente der Körperzusammensetzung und insbesondere der Muskelquerschnitt wurde von einigen neueren Untersuchungen mittels moderner bildgebender Verfahren wie MRT oder CT untersucht (15, 16), die allerdings auf keine validierte Segmentierungs- und Quantifizierungssoftware zurückgreifen konnten.

Dies trifft ebenfalls für die kardiale Volumetrie mittels MRT zu, die derzeit als Goldstandard für die links- und rechtsventrikuläre Volumen- und Massenbestimmung gilt (17). Im Gegensatz zu echokardiographischen Untersuchungen handelt es sich bei der kardialen MRT um ein dreidimensionales Verfahren, welches dadurch eine wesentlich genauere morphologische Darstellung des Herzens ermöglicht (18) und somit u.a.

interventionsbedingte Veränderungen früher zu identifizieren vermag. Darüber hinaus werden sportliche Belastungen immer wieder mit einem plötzlichen Herztod, insbesondere bei männlichen Athleten, assoziiert (23-24). Ob hierfür pathologische morphologische Veränderungen des Myokards durch körperliches Training oder angeborene Organerkrankungen zu Grunde liegen, ist unklar.

10. Angaben zur Nutzen-Risiko-Relation

a. Welcher Nutzen ist von den Ergebnissen der Studie zu erwarten

aa) für die Versuchsteilnehmer?

Steigerung der körperlichen Fitness und physischen Attraktivität durch das Trainingsprogramm. Zudem Verminderung des metabolischen und kardiovaskulären Gesundheitsrisikos und Verbesserung der funktionellen Kapazität. Ggf. Detektion relevanter kardiologischer Befunde (Herzklappendefekte, Kardiomyopathie, Fehlbildungen).

ab) für die Heilkunde?

Beschreibung physiologischer muskulärer, metabolischer, physiologischer und kardialer Adaptation nach intensivem Krafttraining u.a. als Voraussetzung zur Abgrenzung pathologischer Veränderungen und Identifizierung geeigneter Verfahren zur Kontrolle von Trainingseffekten. Sekundäres Ziel ist es, Risikopatienten zu identifizieren, die vor Aufnahme eines spezifischen Trainings ein ausführliches Untersuchungsprogramm durchführen sollten, um Sekundärschäden zur vermeiden.

ac) für die Wissenschaft (z.B. Ergebnisse, die nicht unmittelbar therapeutischen Zwecken dienen)?

Verbesserter Einblick in die Grundlagen trainingsinduzierter muskulärer und kardiovaskulärer Adaptation als Basis optimaler sportwissenschaftlicher Trainingsempfehlungen. Evaluierung und Weiterentwicklung von bildgebenden Untersuchungsverfahren und computerunterstützter Evaluationssoftware zur Diagnostik und zum Monitoring von Interventionseffekten.

b. Mit welchem Risiko ist die Studie für die Versuchsteilnehmer verbunden?

ba) Welcher Art sind die Risiken? Risikoeinschätzung, vorhersehbare Risiken der Behandlung und sonstiger studienbedingter Verfahren, die eingesetzt werden sollen (einschließlich Schmerz, Unannehmlichkeiten, Beschwerden, Verletzung der persönlichen Integrität und Maßnahmen zur Vermeidung und/oder zur Behandlung von unvorhersehbaren/ unerwünschten Ereignissen)

Die Risiken der Intervention (konsequent angeleitetes Krafttrainingsprogramm) sind sehr gering und bleiben nicht zuletzt aufgrund der intensiven Betreuung deutlich hinten denen eines selbständig durchgeführten Muskeltrainings zurück. Selbstverständlich sind in den ersten Wochen der Intervention leichte trainingsbedingte Beschwerden wie bspw. DOMS (Muskelkater) zu erwarten.

Die radiologischen Verfahren Dual Energy X-Ray Absorptiometrie (Gesamtkörper-DXA) bzw. Computertomographie (medialer Anteil Oberschenkel) sind mit niedrigen Strahlendosen (< 10 µSv pro DXA-Messung) bzw. geringen relativen Dosen (<0.8 mSv/CT-Messung[6]) verbunden. Eine Genehmigung dieser Verfahren

[6] Dieser Wert wurde ohne Berücksichtigung entsprechender Schutzmaßnahmen (Abdecken der Gonaden mit Bleischutz errechnet.

wird beim Bundesamt für Strahlenschutz nach Vorliegen der Genehmigung der Ethikkommission selbstverständlich beantragt.

Bei der Blutentnahme und durch periphere Verweilkatheter für die Kontrastmittel-(KM)-Applikation sind Blutergüsse und Infektionen nie komplett ausgeschlossen. In der Kernspintomographie werden nur geringe KM-Mengen eingesetzt. Daher sind die Risiken bei einem Paravasat des KMs in das Weichteilgewebe sowie Nierenbelastungen und mögliche allergische KM-Reaktionen minimiert. Probanden mit relativen oder absoluten Kontraindikationen für eine MRT werden von der Studie ausgeschlossen (s.u.). Insbesondere Probanden mit Nierenfunktionsstörungen oder bekannten KM-Reaktionen. Die KM-Gabe ist notwendig, um relevante kardiale Vorerkrankungen auszuschließen (siehe Ausschlusskriterien), die ein Risiko für die Intervention darstellen können.

bb) Mit welcher Wahrscheinlichkeit ist zu erwarten, daß sich die Risiken realisieren? Wie sicher ist die Wahrscheinlichkeit abschätzbar?

Insgesamt besteht nur eine geringe Wahrscheinlichkeit, dass sich die Risiken realisieren. Die Messungen stellen Standardmessungen der klinischen Routine dar, die von geschultem Fachpersonal sachkundig ausgeführt werden. Die Belastungsvorgaben erfolgen individuell, basierend auf einer Leistungsdiagnostik, sodass eine Überforderung der Teilnehmer kaum zu erwarten ist.

c. Warum ist das mögliche Risiko im Verhältnis zu dem zu erwartenden Nutzen Ihrer Ansicht nach vertretbar?

Es besteht keine wesentlich über das Alltagsrisiko hinausgehende Gefährdung, jedoch ein hoher zu erwartender Nutzen für die Teilnehmer hinsichtlich der Steigerung der körperlichen Fitness, gesundheitsrelevanter Größen, Wohlbefinden, Attraktivität und Selbstwirksamkeit/Kontrollüberzeugung.

d. Werden Zwischenergebnisse ausgewertet, um einen Trend zu erkennen?
ja ☒ nein, kurzer Interventionszeitraum macht Zwischenanalyse inadäquat.

e. Sind Kriterien festgelegt worden, bei deren Eintreten der Versuch geändert oder abgebrochen werden soll? ja, welche? ☒ nein

(allerdings Abbruch des jeweiligen Testverfahrens bei Unwohlsein, oder generell auf Wunsch des Patienten)

11. Bei klinischen Prüfungen nach MPG:

a. Welches Medizinprodukt soll geprüft werden? **entfällt**

b. Wird die klinische Prüfung von einer entsprechend qualifizierten und befugten Person geleitet, die mindestens eine zweijährige Erfahrung in der klinischen Prüfung von Medizinprodukten nachweisen kann? ja nein

c. Wurde (soweit erforderlich) eine dem jeweiligen Stand der wissenschaftlichen Erkenntnisse entsprechende biologische Sicherheitsprüfung oder sonstige für die vorgesehene Zweckbestimmung des Medizinproduktes erforderliche Prüfung durchgeführt? ja nein

d. Wurde (soweit erforderlich) die sicherheitstechnische Unbedenklichkeit für die Anwendung des Medizinproduktes unter Berücksichtigung des Standes der Technik sowie der Arbeitsschutz- und Unfallverhütungsvorschriften nachgewiesen?
ja nein

e. Ist der Leiter der klinischen Prüfung über die Ergebnisse der biologischen Sicherheitsprüfung und die voraussichtlich mit der klinischen Prüfung verbundenen Risiken informiert worden? ja nein

12. a) Ist die Mitarbeit eines Statistikers vorgesehen? ☒ ja nein
b) Welche statistischen Methoden sollen benutzt werden?

Komplettes statistisches Instrumentarium zur Erfassung von Effekten (bspw. Varianzanalysen/nicht parametrische Tests zur Identifikation von Zwischengruppenunterschieden). Zudem regressionsanalytische Modelle zur Aufklärung von Varianzen.

13. a) Handelt es sich um eine multizentrische Studie (d.h. eine nach einem *einzigen* Prüfplan durchgeführte Studie, die in mehr als einer Prüfstelle erfolgt und daher von mehr als einem Prüfer vorgenommen wird)? ja ☒ nein

b) Wurden/Werden an anderer Stelle Studien mit demselben oder einem ähnlichen Ziel durchgeführt? ja, wo? ☒ nein

Es wurden in der Vergangenheit bereits mehrere Untersuchungen mit dem Ziel der Evaluierung eines gesundheitssportlichen Trainings auf muskuläre, physiologische, metabolische und kardiale Parameter (meist isoliert) durchgeführt (s.o.). Diese Studie ist jedoch die erste, die u.a. den Effekt eines intensiven Kraft-/Powertrainings auf muskuläre Parameter, Körperzusammensetzung, metabolische und kardiovaskuläre Größen bei untrainierten Männern in mittlerem Lebensalter auch mittels moderner bildgebender Verfahren und Auswertesoftware evaluiert.

14. Wer hat die Studie initiiert? **Institut für Medizinische Physik**

15. Wer finanziert sie? (*Bitte geben Sie an, ob Drittmittel von nichtöffentlicher Seite beantragt werden. Falls ja, in welcher Höhe?*)

Derzeit sind noch keine Mittel beantragt, es werden jedoch nach positivem Votum der Ethikkommission versucht über unterschiedliche Ebenen Drittmittel zu generieren.

16. Die Aufwandsentschädigung wird übernommen von (*bitte Ansprechpartner benennen*):

Institut für Medizinische Physik (Direktor: Prof. Dr. Dr. W.A. Kalender)

Ansprechpartner: Prof. Dr. Wolfgang Kemmler

III. Angaben zu den Versuchsteilnehmern

1. Anzahl (*bei vergleichenden Studien bitte Aufteilung auf Gruppen angeben*)

80 Personen gesamt:

- Gruppe 1, n = 40: High Intensity Resistance Training (HIT)[7]
- Gruppe 2, n = 40: Kontrollgruppe (Wartegruppe; im Anschluss „Power-Training“)

Bei Nullhypothesen-basierten Studien:
Wurde eine formale Fallzahlschätzung vorgenommen?

☒ ja, Basis CSA-Oberschenkelmuskulatur nein

2. Alter und Geschlecht (*bitte geben Sie das Alter der Versuchsteilnehmer sowie die als Ausschlusskriterien vorgesehenen Ober- und Untergrenzen an*)

[7] Training mit relativ hoher Reizintensität (≥70% des Einwiederholungsmaximums: 1RM)

Männer, 30. - 50. Lebensjahr

3. Status: Handelt es sich bei den Versuchsteilnehmern um
   ☒ gesunde Personen
   schwangere oder stillende Frauen
   Kinder oder Jugendliche
   einschlägig Erkrankte (*bitte geben Sie die Krankheit und das Stadium an*)
   Personen, die an anderen Krankheiten leiden? (Insbesondere: psychische Krankheiten, die Zweifel an der Geschäfts- oder Einsichtsfähigkeit begründen)

4. Welche sonstigen **Einschlusskriterien** (z.B. erlaubte Begleitmedikation) sind vorgesehen?
   - initial Untrainierte (während der vergangenen 2 Jahre: ≤1 h/Woche Sport mit positivem Effekt auf die Muskulatur; ≤2 h/Woche Sport gesamt)

5. Welche sonstigen **Ausschlusskriterien** (z.B. fortgeschrittene Nieren- oder Leber-insuffizienz, verbotene Begleitmedikation etc.) sind vorgesehen?
   - Geschichte leistungssportlicher Ausübung von Disziplinen mit erheblicher Relevanz für Körperzusammensetzung und Kraftfähigkeiten
   - pathologische muskuläre, metabolische und kardiale Veränderungen oder Entzündungen; deutlich eingeschränkte Gelenksbeweglichkeit in Knie und Hüfte.
   - Medikamente/Erkrankungen mit relevantem Einfluss auf Körperzusammensetzung und Herz-Kreislauf-System
   - sehr geringe körperliche Leistungsfähigkeit (< 100 Watt auf dem Fahrradergometer)
   - schwere Adipositas (BMI > 35 kg/m^2)
   - Abwesenheit ≥ 2 Wochen während des Interventionszeitraums
   - Geplante Aufnahme einer relevanten parallelen Trainingsmaßnahme
   - Kontraindikationen gegen MRT (Klaustrophobie, Herzschrittmacher, magnetisier-bare intracorporale Fremdkörper); Körpermaße die eine MRT-Messung verhindern
   - Drogenmissbrauch

6. Sollen auch Personen teilnehmen, die auf gerichtliche oder behördliche Anordnung in einer Anstalt verwahrt werden?
   ja ☒ nein

7. Sollen auch Personen teilnehmen, die sich schon für andere Forschungsvorhaben zur Verfügung gestellt haben?
   ja ☒ nein
   wie lange muss die letzte Teilnahme zurückliegen?

8. Bei Studien an Minderjährigen (oder sonst nicht geschäftsfähigen Personen) **entfällt**
   a. Warum kann die Studie nicht an Erwachsenen (voll Geschäftsfähigen) durchgeführt werden?

   b. Sind Aufklärung und Einwilligung der (des) gesetzlichen Vertreter(s) gewährleistet? (*bitte vorformulierte Erklärung beifügen*)
   ja nein, weil

c. Sind zusätzliche Aufklärung und Einwilligung der minderjährigen (nicht voll geschäftsfähigen) Versuchsteilnehmer gewährleistet, die selbst in der Lage sind, Wesen, Bedeutung und Tragweite des Versuchs einzusehen und ihren Willen danach zu bestimmen?

ja nein

9. Probandenversicherung

Wird zugunsten der Versuchsteilnehmer eine Versicherung abgeschlossen?

ja (*bitte Police beifügen, aus der die Versicherungsgesellschaft und die Höhe der Versicherungsleistung hervorgeht)*

☒ nein

10. Schweigepflicht/Datenschutz

Werden die ärztlichen Schweigepflicht- und die Datenschutzbestimmungen beachtet?

ja

11. Entgelt für Probanden

Soll den Versuchsteilnehmern ein Entgelt (Aufwandsentschädigung o.ä.) gezahlt werden?

ja, in Höhe von EUR ☒ nein

12. Wie sollen die Versuchsteilnehmer über Wesen, Bedeutung und Tragweite der Studie **aufgeklärt** werden?

Bitte in deutscher Sprache beifügen:

Dokumentation des Inhalts der Patientenaufklärung durch die/den versuchsdurchführende/n Ärztin/Arzt (Merkblatt), insbesondere mit Hinweisen über:

- **Ziele und Methoden** der Studie;
- **Nutzen und Risiko** der Studie;
- bekannte und möglicherweise zu erwartende **Wirkungen und Nebenwirkungen** von Medikamenten;
- Eingriffe, die nur aus wissenschaftlichen Gründen erfolgen;
- ein angebrachtes Verhalten des Patienten während und nach dem Versuch;
- die **Widerruflichkeit** einer Einwilligung;
- das Bestehen und den Umfang der gesetzlichen **Probandenversicherung** (Name/Anschrift/Telefon/Fax der Versicherungsgesellschaft, Nummer der Versicherungspolice) sowie die danach von der Versuchsperson zu beachtenden Obliegenheiten;
- **Ausschlusskriterien** (z.B. Schwangerschaft/Stillzeit);
- Name und Telefon des **Ansprechpartner**s vor Ort.
- **Besondere Aufklärung** über die Situation
  a. bei der randomisierten Studie
  f. beim Blind- und Doppelblindversuch.

13. Wie sollen die Versuchsteilnehmer ihre **Einwilligung** in die Teilnahme an der Studie erklären? (*bitte formulierte deutschsprachige Erklärung mit datenschutzrechtlicher Einwilligungserklärung beifügen*)

Schriftliche Einverständniserklärung nach umfangreicher mündlicher und schriftlicher Aufklärung über Ziele, Nutzen und Risiken der Untersuchung.

Ich weiß, daß auch bei einer positiven Beurteilung des Vorhabens durch die Ethik-Kommission der Medizinischen Fakultät der FAU Erlangen-Nürnberg die ärztliche und juristische Verantwortung für die Durchführung des Projektes uneingeschränkt bei der Leiterin/dem Leiter verbleibt.

Erlangen/Nürnberg
Datum .... Unterschrift des/der Antragstellers/in

____
(Name in Druckbuchstaben)

Unterschrift der/des Leiterin/Leiters der Einrichtung, in der das Vorhaben durchgeführt werden soll.
Mit der Durchführung des Forschungsvorhabens einverstanden:

Datum .... Unterschrift des/der Leiters/Leiterin der Einrichtung

____
(Name in Druckbuchstaben)

EK_May09/mit Unterschriftsblatt

Literatur

1. Benson AC, Torode ME, Fiatarone Singh MA. Effects of resistance training on metabolic fitness in children and adolescents: a systematic review. *Obes Rev*. 2008;9(1):43-66.
2. Kelley GA, Kelley KS. Impact of progressive resistance training on lipids and lipoproteins in adults: a meta-analysis of randomized controlled trials. *Prev Med*. 2009;48(1):9-19.
3. Latham NK, Bennett DA, Stretton CM, Anderson CS. Systematic review of progressive resistance strength training in older adults. *J Gerontol A Biol Sci Med Sci*. 2004;59(1):48-61.
4. Macaluso A, De Vito G. Muscle strength, power and adaptations to resistance training in older people. *Eur J Appl Physiol*. 2004;91:450-472.
5. Snowling NJ, Hopkins WG. Effects of different modes of exercise training on glucose control and risk factors for complications in type 2 diabetic patients: a meta-analysis. *Diabetes Care*. 2006;29(11):2518-27.
6. Weineck J. *Optimales Training* Erlangen: Spitta-Verlag; 2007.
7. Weineck J. *Sportbiologie*. Vol. 10 Balingen: Spitta Verlag; 2009.
8. Wijndaele K, Beunen G, Duvigneaud N, et al. A continuous metabolic syndrome risk score: utility for epidemiological analyses. *Diabetes Care*. 2006;29(10):2329.
9. Wilson PW, D'Agostino RB, Levy D, Belanger AM, Silbershatz H, Kannel WB. Prediction of coronary heart disease using risk factor categories. *Circulation*. 1998;97(18):1837-47.
10. Asikainen TM, Kukkonen-Harjula K, Miilunpalo S. Exercise for health for early postmenopausal women: a systematic review of randomised controlled trials. *Sports Med*. 2004;34(11):753-78.
11. Lagally KM, Cordero J, Good J, Brown DD, McCaw ST. Physiologic and metabolic responses to a continuous functional resistance exercise workout. *J Strength Cond Res*. 2009;23(2):373-9.
12. Kay SJ, Fiatarone Singh MA. The influence of physical activity on abdominal fat: a systematic review of the literature. *Obes Rev*. 2006;7(2):183-200.
13. Kemmler W, von Stengel S, Engelke K, Haberle L, Mayhew JL, Kalender WA. Exercise, body composition, and functional ability: a randomized controlled trial. *Am J Prev Med*. 2010;38(3):279-87.
14. Lamb HJ. Total body fat distribution as part of multiorgan MR imaging: new tool for risk assessment in the metabolic syndrome? *Radiology*. 2010;257(2):307-8.
15. Weiss EP, Racette SB, Villareal DT, et al. Lower extremity muscle size and strength and aerobic capacity decrease with caloric restriction but not with exercise-induced weight loss. *J Appl Physiol*. 2007;102(2):634-40.
16. Valtonen A, Poyhonen T, Sipila S, Heinonen A. Effects of aquatic resistance training on mobility limitation and lower-limb impairments after knee replacement. *Arch Phys Med Rehabil*;91(6):833-9.
17. Petersen SE, Hudsmith LE, Robson MD, et al. Sex-specific characteristics of cardiac function, geometry, and mass in young adult elite athletes. *J Magn Reson Imaging*. 2006;24(2):297-303.
18. Grothues F, Smith GC, Moon JC, et al. Comparison of interstudy reproducibility of cardiovascular magnetic resonance with two-dimensional echocardiography in normal subjects and in patients with heart failure or left ventricular hypertrophy. *Am J Cardiol*. 2002;90(1):29-34.

An die Geschäftsstelle der
Ethik-Kommission
der Medizinischen Fakultät
der FAU Erlangen-Nürnberg
Krankenhausstr. 12
91054 Erlangen

Antrag
an die Ethik-Kommission
der Medizinischen Fakultät

Bitte ***in deutscher Sprache*** *ausfüllen,*
Zutreffendes bitte ankreuzen.
Für multizentrische Studien mit Vorvotum einer nach Landesrecht gebildeten zuständigen Ethik-Kommission können Sie das verkürzte Antragsformular verwenden, abzurufen unter: http://www.ethik.med.uni-erlangen.de *(Anschlussvotum)*

Antrag auf Beurteilung eines Forschungsprojektes (keine Arzneimittelprüfung)

bitte 9-fach einschließlich Anlagen einreichen sowie 1-mal in elektronischer Fassung

Titel des Projektes:
Einfluss eines 16-wöchigen Kraft-Trainingsprogramms auf leistungsphysiologische und gesundheitsrelevante muskuläre und kardiale Größen bei untrainierten Männern im mittleren Lebensalter. Eine randomisierte kontrollierte Interventionsstudie mit modernen, bildgebenden Verfahren.

I. Projektleitung

1. Name der/des verantwortlichen Projektleiterin/s an der FAU:
Professor Dr. Wolfgang Kemmler[1], Osteoporose-Forschungszentrum, Institut für Medizinische Physik, FAU (Direktor: Professor Dr. Dr. Willi A. Kalender)
App.-Nr. 23999; E-Mail: wolfgang.kemmler@imp.uni-erlangen.de

Angaben über die Qualifikation der/des Versuchsleiterin/s:
Promotion, Habilitation (liegen bereits vor)

2. a) Weitere Teilnehmer/innen vor Ort (alphabetische Reihenfolge):
Prof. Dr. Klaus Engelke, IMP, FAU
PD. Dr. Michael Lell, Radiologisches Institut, FAU
Prof. Dr. Harald Quick, IMP, FAU
PD. Dr. Axel Schmid, Radiologisches Institut, FAU
Dr. Michael Scharf, Radiologisches Institut, FAU
Dr. Simon von Stengel, Osteoporoseforschungszentrum, FAU
Andreas Wittke, IMP, FAU

b) Weitere Prüfzentren (bei multizentrischen Studien): keine

3. Handelt es sich bei diesem Antrag um ein bereits von der Ethik-Kommission der Medizinischen Fakultät der Friedrich-Alexander-Universität Erlangen-Nürnberg begutachtetes Projekt?

ja (bitte lfd. Nr. angeben) ☒ nein

II. Forschungsvorhaben

1. Geplanter Beginn der Studie: September 2012 // voraussichtliches Ende: August 2013
Dauer der Studienteilnahme für den einzelnen Probanden:

Intervention (s.u.): 4 Monate // 8 Monate (Kontrollgruppen-Wartelisten-Design)
Untersuchung (s.u.): ca. 100 min jeweils zu Beginn und Studienende

2. Kurzer Abriss des Projektes *(maximal 1,5 Seiten)*:

Einführung und Fragestellung
Krafttraining gilt auch bei intensiver Durchführung als gesundheitsfördernde Intervention, die sich bei einem zunehmenden Anteil der Bevölkerung immer größerer Beliebtheit erfreut. Neben Körperformung und Attraktivität steht für viele Menschen der präventive Aspekt des Muskeltrainings im Vordergrund. Tatsächlich weisen eine Vielzahl von Untersuchungen (1-5) positive Effekte eines regelmäßig durchgeführten, „überschwelligen“ Trainings nach, so auf gesundheitsrelevante muskuläre, physiologische, metabolische und kardiovaskuläre Parameter wie beispielsweise die Körperzusammensetzung/funktionelle Kapazität, Blutfette/Lipoproteine oder Glucoseintoleranz/ Insulinsensitivität. Zudem ist anzunehmen, dass auch relativ rasche funktionelle und morphologische Anpassungserscheinungen des Herzens nachweisbar sind (6, 7). Allerdings liegen keine kernspintomographischen Längsschnittuntersuchungen vor, welche die physiologischen Adaptationserscheinungen des Herzens nach einem mehrmonatigen intensiven Krafttraining bei inaktiven Personen evaluieren. Parallel dazu sind die vorliegenden Methoden, mit denen die Körperzusammensetzung und die muskuläre Massenentwicklung bislang evaluiert wurden, als suboptimal einzuschätzen. Um eine valide und reliable Erfassung der Veränderung muskulärer, physiologischer und kardiologischer Größen als Reaktion auf ein gesundheitsorientiertes Muskeltraining zu gewährleisten, ist der messmethodische Schwerpunkt der vorliegenden Untersuchung auf den Einsatz moderner bildgebender Verfahren (Kernspintomographie (MRT), Computertomographie (CT), Dual Energy X-Ray Absorptiometry (DXA)) und moderner Segmentierungs- und Quantifizierungssoftware gerichtet.
Ziel der Untersuchung ist somit die Evaluierung der Effekte eines intensiven 16-wöchigen Krafttrainingsprogramms auf leistungsphysiologische und gesundheitsrelevante muskuläre und kardiale Größen bei untrainierten Männern im mittleren Lebensalter unter besonderer Berücksichtigung bildgebender Verfahren.

Design:
Randomisierte, kontrollierte, teilverblindete Studie mit Wartegruppe (cross-over)

Endpunkte
Primäre Endpunkte:

- Muskelquerschnitt der Oberschenkelmuskulatur (MRT, CT)
- Intraabdominale Fettmasse (MRT)

Sekundäre Endpunkte:

- Kraftfähigkeiten (u.a. abhängige Variable) [1]
- Gesamtkörperfett und Muskelmasse sowie regionale Verteilung (DXA, MRT)
- Metabolisches Syndrom-Score (8), und 10 Jahres-CHD-Risiko (9)
- Kardiale Masse und enddiastolisches Volumen (MRT)
- Hormonelle Regulation (u.a. freies Testosteron, hGH, Cortisol)
- Schmerzintensität, Quality of Life

[1] Ein Ziel der Studie ist die Identifikation von Variablen, welche die Varianz des Kraft-/Leistungszuwachs am höchsten aufklären.

Stichprobe

Zwei-Gruppen Design, randomisiert, z.T. cross-over (mit Wartegruppe)[2]

- Gruppe 1, n = 40: High Intensity Resistance Training (HIT)[3]
- Gruppe 2, n = 40: Kontrollgruppe (Wartegruppe; im Anschluss „Power-Training[4]“)

Interventionsprogramm

Gruppe 1: HIT mit konventioneller Bewegungsgeschwindigkeit (TUT[5] 2s-1s-2s)

Periodisiertes, progressives Krafttraining über 16 Wochen, basierend auf individuellen Trainingsplänen auf der Basis von 1 RM/x RM-Tests (1, 4, 10 Wochen), zunehmende Intensivierung der Reizhöhe (bis Woche 8), anschließend periodisiertes HIT (70-92,5%, 1RM) mit 2-3 Trainingseinheiten je Woche (1-2 gemeinsame, überwachte Trainingseinheiten; 1-2 Trainingseinheiten in Eigenregie (Vorgaben über Trainingsplan)).

Gruppe 2 (ehemalige Kontrollgruppe): Power-Training (TUT: ϟ-1s-2s)

s.o. aber explosive Ausführung im konzentrischen Bewegungsbereich nach initialer Konditionierung über 6 Wochen. Vergleichbares Trainingsprotokoll, allerdings im Intensitätsbereich von 40-70%, 1RM.

3. Studienbezogene Maßnahmen:

Bitte beschreiben Sie hier alle Maßnahmen, die studienbedingt durchgeführt werden sowie alle erforderlichen Abweichungen von der üblichen Routine-Behandlung:

Interventionsprogramm s.o.

Messungen (jeweils basal und nach 16 Wochen)

Bildgebende Verfahren:

Ganzkörper-MRT und Ganzkörper-DXA zur Erfassung der gesamten und regionalen Körperzusammensetzung.

Lokale MRT und CT am Oberschenkel (über „Muskelbauch“)

Kardiale MRT-Untersuchung mit Kontrastmittel i.v. (Analyse fibrotischer Herzmuskelveränderungen, Strain der Herzmuskulatur, enddiastolisches Volumen (EDV), endsystolisches Volumen (ESV), Schlagvolumen (SV), Ejektionsfraktion (EF) und myokardiale Masse (MM), Herzmuskeldicke).

Blutdruck und Herzfrequenzverhalten in Ruhe

Körperliche Fitness:

Erfassung unterschiedlicher Kraftfähigkeiten (u.a. 1 RM, Schnellkraft) mittels isokinetischem Dynamometer.

Psychosoziale Parameter

Befindlichkeit und Schmerzparameter, QoL (Fragebogen)

Labor

Blutfette/Lipoproteine, Glukose, Insulin, HbAC1, Entzündungsmarker

Testosteron, freies Testosteron, hGH, Cortisol; (evt. noch Parameter des Immunsystems)

Ernährungsanalyse

Analyse über 4 Tage (standardisierte Protokolle)

Anamnese und Risikofaktorenprofil über Fragebogen

---

[2] Die Wartegruppe dient zunächst als parallele Kontrollgruppe zur Interventionsgruppe. Nach Abschluss dieses Untersuchungsabschnittes wird mit dieser Gruppe ebenfalls eine 16-wöchige Intervention durchgeführt, sodass ein eingeschränktes 3-Gruppendesign generiert wird.

[3] Training mit relativ hoher Reizintensität (≥70% des Einwiederholungsmaximums: 1RM)

[4] Training mit explosiver Bewegungsausführung im konzentrischen Bereich (bei vglw. geringer Reizintensität im Bereich 40-60% 1RM)

[5] **T**ime **U**nder **T**ension: Dauer der jeweiligen Bewegungsabschnitte, konzentrisch – isometrisch – exzentrisch in Sec.

4. Wird die Studie gemäß der von der 48. Generalversammlung des Weltärztebundes in Somerset West revidierten Deklaration von Helsinki aus dem Jahre 1996 durchgeführt? Bitte angeben, ob alle anderen Erprobungsmöglichkeiten ausgeschöpft wurden.

Ja, die Intervention wurde in vorhergehenden Studien bereits validiert und optimiert. Die Messtechnologie ist ebenfalls etabliert, wurde aber bislang nur suboptimal zur Validierung der hier genannten Fragestellung eingesetzt.

5. Art des Forschungsvorhabens:
Handelt es sich um
eine diagnostische Prüfung?
eine therapeutische Prüfung?
eine Verträglichkeitsprüfung?
☒ einen ausschließlich wissenschaftlichen Versuch?

6. Gesetzliche Grundlagen

a) Handelt es sich um eine Untersuchung, die dazu bestimmt ist, klinische oder pharmakologische Wirkungen von Arzneimitteln zu erforschen oder nachzuweisen oder Nebenwirkungen festzustellen oder die Resorption, die Verteilung, den Stoffwechsel oder die Ausscheidung zu untersuchen, **mit dem Ziel, sich von der Unbedenklichkeit oder Wirksamkeit des Arzneimittels zu überzeugen** (klinische Prüfung eines Arzneimittels nach §§ 40 Arzneimittelgesetz)?

nein

Bitte begründen. Erläuterungen zum Antrag auf Bewertung einer klinischen Arzneimittelprüfung nach § 40 AMG finden Sie unter http://www.ethik.med.uni-erlangen.de/

b) Handelt es sich um eine klinische Prüfung nach § 20 Medizinproduktegesetz (MPG)?
ja ☒ nein
Bitte begründen. Liegt eine CE-Zertifizierung für das Medizinprodukt vor? Werden zusätzlich invasive oder andere belastende Untersuchungen durchgeführt?
Sämtliche Messverfahren sind CE-zertifiziert und entsprechend MPG geprüft und zugelassen. Invasive Untersuchungen werden nicht durchgeführt, mit Ausnahme von Blutentnahmen und einer venösen Kontrastmittelapplikation.

c) Handelt es sich um ein Vorhaben nach § 8 des Gesetzes zur Regelung des Transfusionswesens (TFG)?
ja ☒ nein

7. Handelt es sich um einen Versuch nach

§ 23 Strahlenschutzverordnung? ☒ ja nein

§ 28 Röntgenverordnung? ☒ ja nein

8. Typ der Studie:

- offen
- ☒ blind
- doppelblind
- ☒ vergleichend
- ☒ randomisiert
- multizentrisch
- ☒ Feldstudie
- Pilotstudie

9. Wissenschaftliche Begründung des Projekts, insbesondere:

a. Erläuterung des Versuchsziels

Das Ziel der Untersuchung ist die Evaluierung eines intensiven körperlichen Krafttrainings auf leistungsphysiologische und gesundheitsrelevante muskuläre und kardiale Größen bei untrainierten Männern im mittleren Lebensalter unter besonderer Berücksichtigung moderner bildgebender Verfahren. Aus radiologischer Sicht ist das Ziele der Studie die Identifikation typischer Kenngrößen der physiologischen Adaption der Körperzusammensetzung und des Herz-Kreislauf-Systems nach unterschiedlichen Typen von Krafttraining (s.o.) sowie der Methodenvergleich kernspintomographischer mit computertomographischen Muskelquerschnitten des Oberschenkels sowie der gesamten und der regionalen Gesamtkörperzusammensetzung mittels MRT vs. dem Goldstandard DXA-Methode.

b. Darstellung des bisherigen Wissensstandes

Eine Vielzahl von wissenschaftlichen Untersuchungen berichten den positiven Effekt eines „Krafttrainings“ auf funktionelle und gesundheitsrelevante physiologische und metabolische Größen (Übersicht in (1, 3, 10, 11)) bei Menschen in mittlerem Lebensalter. Obgleich viele dieser Daten bereits in frühen Studien mit suboptimaler Messmethodik/-technik und schlechter Reproduzierbarkeit evaluiert wurden, gelten sie in der wissenschaftlichen Literatur als absolut verlässlich und werden vielfach zitiert. Moderne bildgebende Verfahren wie die Kernspintomographie (MRT) oder die Computertomographie (CT) im Verbund mit valider Segmentierungstechnologie und quantitativer Analyse haben die in der Vergangenheit eingesetzten Verfahren mit suboptimaler Auflösung und ausschließlich qualitativer Auswerteprozedur bereits in vielen Bereichen ersetzt. So können Risikofaktoren wie die Körperfettverteilung derzeit schon quantifiziert und der Einfluss einer Intervention validiert werden (12-14). Auch die muskuläre Komponente der Körperzusammensetzung und insbesondere der Muskelquerschnitt wurde von einigen neueren Untersuchungen mittels moderner bildgebender Verfahren wie MRT oder CT untersucht (15, 16), die allerdings auf keine validierte Segmentierungs- und Quantifizierungssoftware zurückgreifen konnten.

Dies trifft ebenfalls für die kardiale Volumetrie mittels MRT zu, die derzeit als Goldstandard für die links- und rechtsventrikuläre Volumen- und Massenbestimmung gilt (17). Im Gegensatz zu echokardiographischen Untersuchungen handelt es sich bei der kardialen MRT um ein dreidimensionales Verfahren, welches dadurch eine wesentlich genauere morphologische Darstellung des Herzens ermöglicht (18) und somit u.a.

interventionsbedingte Veränderungen früher zu identifizieren vermag. Darüber hinaus werden sportliche Belastungen immer wieder mit einem plötzlichen Herztod, insbesondere bei männlichen Athleten, assoziiert (23-24). Ob hierfür pathologische morphologische Veränderungen des Myokards durch körperliches Training oder angeborene Organerkrankungen zu Grunde liegen, ist unklar.

10. Angaben zur Nutzen-Risiko-Relation

a. Welcher Nutzen ist von den Ergebnissen der Studie zu erwarten

aa) für die Versuchsteilnehmer?

Steigerung der körperlichen Fitness und physischen Attraktivität durch das Trainingsprogramm. Zudem Verminderung des metabolischen und kardiovaskulären Gesundheitsrisikos und Verbesserung der funktionellen Kapazität. Ggf. Detektion relevanter kardiologischer Befunde (Herzklappendefekte, Kardiomyopathie, Fehlbildungen).

ab) für die Heilkunde?

Beschreibung physiologischer muskulärer, metabolischer, physiologischer und kardialer Adaptation nach intensivem Krafttraining u.a. als Voraussetzung zur Abgrenzung pathologischer Veränderungen und Identifizierung geeigneter Verfahren zur Kontrolle von Trainingseffekten. Sekundäres Ziel ist es, Risikopatienten zu identifizieren, die vor Aufnahme eines spezifischen Trainings ein ausführliches Untersuchungsprogramm durchführen sollten, um Sekundärschäden zur vermeiden.

ac) für die Wissenschaft (z.B. Ergebnisse, die nicht unmittelbar therapeutischen Zwecken dienen)?

Verbesserter Einblick in die Grundlagen trainingsinduzierter muskulärer und kardiovaskulärer Adaptation als Basis optimaler sportwissenschaftlicher Trainings-empfehlungen. Evaluierung und Weiterentwicklung von bildgebenden Untersuchungs-verfahren und computerunterstützter Evaluationssoftware zur Diagnostik und zum Monitoring von Interventionseffekten.

b. Mit welchem Risiko ist die Studie für die Versuchsteilnehmer verbunden?

ba) Welcher Art sind die Risiken? Risikoeinschätzung, vorhersehbare Risiken der Behandlung und sonstiger studienbedingter Verfahren, die eingesetzt werden sollen (einschließlich Schmerz, Unannehmlichkeiten, Beschwerden, Verletzung der persönlichen Integrität und Maßnahmen zur Vermeidung und/oder zur Behandlung von unvorhersehbaren/ unerwünschten Ereignissen)

Die Risiken der Intervention (konsequent angeleitetes Krafttrainingsprogramm) sind sehr gering und bleiben nicht zuletzt aufgrund der intensiven Betreuung deutlich hinten denen eines selbständig durchgeführten Muskeltrainings zurück. Selbstverständlich sind in den ersten Wochen der Intervention leichte trainingsbedingte Beschwerden wie bspw. DOMS (Muskelkater) zu erwarten.

Die radiologischen Verfahren Dual Energy X-Ray Absorptiometrie (Gesamtkörper-DXA) bzw. Computertomographie (medialer Anteil Oberschenkel) sind mit niedrigen Strahlendosen (< 10 µSv pro DXA-Messung) bzw. geringen relativen Dosen (<0.8 mSv/CT-Messung[6]) verbunden. Eine Genehmigung dieser Verfahren

[6] Dieser Wert wurde ohne Berücksichtigung entsprechender Schutzmaßnahmen (Abdecken der Gonaden mit Bleischutz errechnet.

wird beim Bundesamt für Strahlenschutz nach Vorliegen der Genehmigung der Ethikkommission selbstverständlich beantragt.

Bei der Blutentnahme und durch periphere Verweilkatheter für die Kontrastmittel-(KM)-Applikation sind Blutergüsse und Infektionen nie komplett ausgeschlossen. In der Kernspintomographie werden nur geringe KM-Mengen eingesetzt. Daher sind die Risiken bei einem Paravasat des KMs in das Weichteilgewebe sowie Nierenbelastungen und mögliche allergische KM-Reaktionen minimiert. Probanden mit relativen oder absoluten Kontraindikationen für eine MRT werden von der Studie ausgeschlossen (s.u.). Insbesondere Probanden mit Nierenfunktionsstörungen oder bekannten KM-Reaktionen. Die KM-Gabe ist notwendig, um relevante kardiale Vorerkrankungen auszuschließen (siehe Ausschlusskriterien), die ein Risiko für die Intervention darstellen können.

bb) Mit welcher Wahrscheinlichkeit ist zu erwarten, daß sich die Risiken realisieren? Wie sicher ist die Wahrscheinlichkeit abschätzbar?

Insgesamt besteht nur eine geringe Wahrscheinlichkeit, dass sich die Risiken realisieren. Die Messungen stellen Standardmessungen der klinischen Routine dar, die von geschultem Fachpersonal sachkundig ausgeführt werden. Die Belastungsvorgaben erfolgen individuell, basierend auf einer Leistungsdiagnostik, sodass eine Überforderung der Teilnehmer kaum zu erwarten ist.

c. Warum ist das mögliche Risiko im Verhältnis zu dem zu erwartenden Nutzen Ihrer Ansicht nach vertretbar?

Es besteht keine wesentlich über das Alltagsrisiko hinausgehende Gefährdung, jedoch ein hoher zu erwartender Nutzen für die Teilnehmer hinsichtlich der Steigerung der körperlichen Fitness, gesundheitsrelevanter Größen, Wohlbefinden, Attraktivität und Selbstwirksamkeit/Kontrollüberzeugung.

d. Werden Zwischenergebnisse ausgewertet, um einen Trend zu erkennen?
ja ☒ nein, kurzer Interventionszeitraum macht Zwischenanalyse inadäquat.

e. Sind Kriterien festgelegt worden, bei deren Eintreten der Versuch geändert oder abgebrochen werden soll? ja, welche? ☒ nein

(allerdings Abbruch des jeweiligen Testverfahrens bei Unwohlsein, oder generell auf Wunsch des Patienten)

11. Bei klinischen Prüfungen nach MPG:

a. Welches Medizinprodukt soll geprüft werden? **entfällt**

b. Wird die klinische Prüfung von einer entsprechend qualifizierten und befugten Person geleitet, die mindestens eine zweijährige Erfahrung in der klinischen Prüfung von Medizinprodukten nachweisen kann? ja nein

c. Wurde (soweit erforderlich) eine dem jeweiligen Stand der wissenschaftlichen Erkenntnisse entsprechende biologische Sicherheitsprüfung oder sonstige für die vorgesehene Zweckbestimmung des Medizinproduktes erforderliche Prüfung durchgeführt? ja nein

d. Wurde (soweit erforderlich) die sicherheitstechnische Unbedenklichkeit für die Anwendung des Medizinproduktes unter Berücksichtigung des Standes der Technik sowie der Arbeitsschutz- und Unfallverhütungsvorschriften nachgewiesen?
ja nein

e. Ist der Leiter der klinischen Prüfung über die Ergebnisse der biologischen Sicherheitsprüfung und die voraussichtlich mit der klinischen Prüfung verbundenen Risiken informiert worden? ja nein

12. a) Ist die Mitarbeit eines Statistikers vorgesehen? ☒ ja nein
b) Welche statistischen Methoden sollen benutzt werden?

Komplettes statistisches Instrumentarium zur Erfassung von Effekten (bspw. Varianzanalysen/nicht parametrische Tests zur Identifikation von Zwischengruppenunterschieden). Zudem regressionsanalytische Modelle zur Aufklärung von Varianzen.

13. a) Handelt es sich um eine multizentrische Studie (d.h. eine nach einem *einzigen* Prüfplan durchgeführte Studie, die in mehr als einer Prüfstelle erfolgt und daher von mehr als einem Prüfer vorgenommen wird)? ja ☒ nein

b) Wurden/Werden an anderer Stelle Studien mit demselben oder einem ähnlichen Ziel durchgeführt? ja, wo? ☒ nein

Es wurden in der Vergangenheit bereits mehrere Untersuchungen mit dem Ziel der Evaluierung eines gesundheitssportlichen Trainings auf muskuläre, physiologische, metabolische und kardiale Parameter (meist isoliert) durchgeführt (s.o.). Diese Studie ist jedoch die erste, die u.a. den Effekt eines intensiven Kraft-/Powertrainings auf muskuläre Parameter, Körperzusammensetzung, metabolische und kardiovaskuläre Größen bei untrainierten Männern in mittlerem Lebensalter auch mittels moderner bildgebender Verfahren und Auswertesoftware evaluiert.

14. Wer hat die Studie initiiert? **Institut für Medizinische Physik**

15. Wer finanziert sie? (*Bitte geben Sie an, ob Drittmittel von nichtöffentlicher Seite beantragt werden. Falls ja, in welcher Höhe?*)

Derzeit sind noch keine Mittel beantragt, es werden jedoch nach positivem Votum der Ethikkommission versucht über unterschiedliche Ebenen Drittmittel zu generieren.

16. Die Aufwandsentschädigung wird übernommen von (*bitte Ansprechpartner benennen*):

Institut für Medizinische Physik (Direktor: Prof. Dr. Dr. W.A. Kalender)

Ansprechpartner: Prof. Dr. Wolfgang Kemmler

III. Angaben zu den Versuchsteilnehmern

1. Anzahl (*bei vergleichenden Studien bitte Aufteilung auf Gruppen angeben*)

80 Personen gesamt:

- Gruppe 1, n = 40: High Intensity Resistance Training (HIT)[7]
- Gruppe 2, n = 40: Kontrollgruppe (Wartegruppe; im Anschluss „Power-Training“)

Bei Nullhypothesen-basierten Studien:
Wurde eine formale Fallzahlschätzung vorgenommen?

☒ ja, Basis CSA-Oberschenkelmuskulatur nein

2. Alter und Geschlecht (*bitte geben Sie das Alter der Versuchsteilnehmer sowie die als Ausschlusskriterien vorgesehenen Ober- und Untergrenzen an*)

---

[7] Training mit relativ hoher Reizintensität (≥70% des Einwiederholungsmaximums: 1RM)

Männer, 30. - 50. Lebensjahr

3. Status: Handelt es sich bei den Versuchsteilnehmern um
   ☒ gesunde Personen
   schwangere oder stillende Frauen
   Kinder oder Jugendliche
   einschlägig Erkrankte (*bitte geben Sie die Krankheit und das Stadium an*)
   Personen, die an anderen Krankheiten leiden? (Insbesondere: psychische Krankheiten, die Zweifel an der Geschäfts- oder Einsichtsfähigkeit begründen)

4. Welche sonstigen **Einschlusskriterien** (z.B. erlaubte Begleitmedikation) sind vorgesehen?

- initial Untrainierte (während der vergangenen 2 Jahre: ≤1 h/Woche Sport mit positivem Effekt auf die Muskulatur; ≤2 h/Woche Sport gesamt)

5. Welche sonstigen **Ausschlusskriterien** (z.B. fortgeschrittene Nieren- oder Leber-insuffizienz, verbotene Begleitmedikation etc.) sind vorgesehen?

- Geschichte leistungssportlicher Ausübung von Disziplinen mit erheblicher Relevanz für Körperzusammensetzung und Kraftfähigkeiten
- pathologische muskuläre, metabolische und kardiale Veränderungen oder Entzündungen; deutlich eingeschränkte Gelenksbeweglichkeit in Knie und Hüfte.
- Medikamente/Erkrankungen mit relevantem Einfluss auf Körperzusammensetzung und Herz-Kreislauf-System
- sehr geringe körperliche Leistungsfähigkeit (< 100 Watt auf dem Fahrradergometer)
- schwere Adipositas (BMI > 35 kg/m^2)
- Abwesenheit ≥ 2 Wochen während des Interventionszeitraums
- Geplante Aufnahme einer relevanten parallelen Trainingsmaßnahme
- Kontraindikationen gegen MRT (Klaustrophobie, Herzschrittmacher, magnetisier-bare intracorporale Fremdkörper); Körpermaße die eine MRT-Messung verhindern
- Drogenmissbrauch

6. Sollen auch Personen teilnehmen, die auf gerichtliche oder behördliche Anordnung in einer Anstalt verwahrt werden?
   ja ☒ nein

7. Sollen auch Personen teilnehmen, die sich schon für andere Forschungsvorhaben zur Verfügung gestellt haben?
   ja ☒ nein
   wie lange muss die letzte Teilnahme zurückliegen?

8. Bei Studien an Minderjährigen (oder sonst nicht geschäftsfähigen Personen) **entfällt**
   a. Warum kann die Studie nicht an Erwachsenen (voll Geschäftsfähigen) durchgeführt werden?

   b. Sind Aufklärung und Einwilligung der (des) gesetzlichen Vertreter(s) gewährleistet? (*bitte vorformulierte Erklärung beifügen*)
   ja nein, weil

c. Sind zusätzliche Aufklärung und Einwilligung der minderjährigen (nicht voll geschäftsfähigen) Versuchsteilnehmer gewährleistet, die selbst in der Lage sind, Wesen, Bedeutung und Tragweite des Versuchs einzusehen und ihren Willen danach zu bestimmen?

ja nein

9. Probandenversicherung

Wird zugunsten der Versuchsteilnehmer eine Versicherung abgeschlossen?

ja (*bitte Police beifügen, aus der die Versicherungsgesellschaft und die Höhe der Versicherungsleistung hervorgeht)*

☒ nein

10. Schweigepflicht/Datenschutz

Werden die ärztlichen Schweigepflicht- und die Datenschutzbestimmungen beachtet?

ja

11. Entgelt für Probanden

Soll den Versuchsteilnehmern ein Entgelt (Aufwandsentschädigung o.ä.) gezahlt werden?

ja, in Höhe von EUR ☒ nein

12. Wie sollen die Versuchsteilnehmer über Wesen, Bedeutung und Tragweite der Studie **aufgeklärt** werden?

Bitte in deutscher Sprache beifügen:

Dokumentation des Inhalts der Patientenaufklärung durch die/den versuchsdurchführende/n Ärztin/Arzt (Merkblatt), insbesondere mit Hinweisen über:

- **Ziele und Methoden** der Studie;
- **Nutzen und Risiko** der Studie;
- bekannte und möglicherweise zu erwartende **Wirkungen und Nebenwirkungen** von Medikamenten;
- Eingriffe, die nur aus wissenschaftlichen Gründen erfolgen;
- ein angebrachtes Verhalten des Patienten während und nach dem Versuch;
- die **Widerruflichkeit** einer Einwilligung;
- das Bestehen und den Umfang der gesetzlichen **Probandenversicherung** (Name/Anschrift/Telefon/Fax der Versicherungsgesellschaft, Nummer der Versicherungspolice) sowie die danach von der Versuchsperson zu beachtenden Obliegenheiten;
- **Ausschlusskriterien** (z.B. Schwangerschaft/Stillzeit);
- Name und Telefon des **Ansprechpartner**s vor Ort.
- **Besondere Aufklärung** über die Situation
  - a. bei der randomisierten Studie
  - f. beim Blind- und Doppelblindversuch.

13. Wie sollen die Versuchsteilnehmer ihre **Einwilligung** in die Teilnahme an der Studie erklären? (*bitte formulierte deutschsprachige Erklärung mit datenschutzrechtlicher Einwilligungserklärung beifügen*)

Schriftliche Einverständniserklärung nach umfangreicher mündlicher und schriftlicher Aufklärung über Ziele, Nutzen und Risiken der Untersuchung.

Ich weiß, daß auch bei einer positiven Beurteilung des Vorhabens durch die Ethik-Kommission der Medizinischen Fakultät der FAU Erlangen-Nürnberg die ärztliche und juristische Verantwortung für die Durchführung des Projektes uneingeschränkt bei der Leiterin/dem Leiter verbleibt.

Erlangen/Nürnberg
Datum .... Unterschrift des/der Antragstellers/in

____
(Name in Druckbuchstaben)

Unterschrift der/des Leiterin/Leiters der Einrichtung, in der das Vorhaben durchgeführt werden soll.
Mit der Durchführung des Forschungsvorhabens einverstanden:

Datum .... Unterschrift des/der Leiters/Leiterin der Einrichtung

____
(Name in Druckbuchstaben)

EK_May09/mit Unterschriftsblatt

Literatur

1. Benson AC, Torode ME, Fiatarone Singh MA. Effects of resistance training on metabolic fitness in children and adolescents: a systematic review. *Obes Rev*. 2008;9(1):43-66.
2. Kelley GA, Kelley KS. Impact of progressive resistance training on lipids and lipoproteins in adults: a meta-analysis of randomized controlled trials. *Prev Med*. 2009;48(1):9-19.
3. Latham NK, Bennett DA, Stretton CM, Anderson CS. Systematic review of progressive resistance strength training in older adults. *J Gerontol A Biol Sci Med Sci*. 2004;59(1):48-61.
4. Macaluso A, De Vito G. Muscle strength, power and adaptations to resistance training in older people. *Eur J Appl Physiol*. 2004;91:450-472.
5. Snowling NJ, Hopkins WG. Effects of different modes of exercise training on glucose control and risk factors for complications in type 2 diabetic patients: a meta-analysis. *Diabetes Care*. 2006;29(11):2518-27.
6. Weineck J. *Optimales Training* Erlangen: Spitta-Verlag; 2007.
7. Weineck J. *Sportbiologie*. Vol. 10 Balingen: Spitta Verlag; 2009.
8. Wijndaele K, Beunen G, Duvigneaud N, et al. A continuous metabolic syndrome risk score: utility for epidemiological analyses. *Diabetes Care*. 2006;29(10):2329.
9. Wilson PW, D'Agostino RB, Levy D, Belanger AM, Silbershatz H, Kannel WB. Prediction of coronary heart disease using risk factor categories. *Circulation*. 1998;97(18):1837-47.
10. Asikainen TM, Kukkonen-Harjula K, Miilunpalo S. Exercise for health for early postmenopausal women: a systematic review of randomised controlled trials. *Sports Med*. 2004;34(11):753-78.
11. Lagally KM, Cordero J, Good J, Brown DD, McCaw ST. Physiologic and metabolic responses to a continuous functional resistance exercise workout. *J Strength Cond Res*. 2009;23(2):373-9.
12. Kay SJ, Fiatarone Singh MA. The influence of physical activity on abdominal fat: a systematic review of the literature. *Obes Rev*. 2006;7(2):183-200.
13. Kemmler W, von Stengel S, Engelke K, Haberle L, Mayhew JL, Kalender WA. Exercise, body composition, and functional ability: a randomized controlled trial. *Am J Prev Med*. 2010;38(3):279-87.
14. Lamb HJ. Total body fat distribution as part of multiorgan MR imaging: new tool for risk assessment in the metabolic syndrome? *Radiology*. 2010;257(2):307-8.
15. Weiss EP, Racette SB, Villareal DT, et al. Lower extremity muscle size and strength and aerobic capacity decrease with caloric restriction but not with exercise-induced weight loss. *J Appl Physiol*. 2007;102(2):634-40.
16. Valtonen A, Poyhonen T, Sipila S, Heinonen A. Effects of aquatic resistance training on mobility limitation and lower-limb impairments after knee replacement. *Arch Phys Med Rehabil*;91(6):833-9.
17. Petersen SE, Hudsmith LE, Robson MD, et al. Sex-specific characteristics of cardiac function, geometry, and mass in young adult elite athletes. *J Magn Reson Imaging*. 2006;24(2):297-303.
18. Grothues F, Smith GC, Moon JC, et al. Comparison of interstudy reproducibility of cardiovascular magnetic resonance with two-dimensional echocardiography in normal subjects and in patients with heart failure or left ventricular hypertrophy. *Am J Cardiol*. 2002;90(1):29-34.

An die Geschäftsstelle der
Ethik-Kommission
der Medizinischen Fakultät
der FAU Erlangen-Nürnberg
Krankenhausstr. 12
91054 Erlangen

Antrag
an die Ethik-Kommission
der Medizinischen Fakultät

*Bitte **in deutscher Sprache** ausfüllen,*
Zutreffendes bitte ankreuzen.
Für multizentrische Studien mit Vorvotum einer nach Landesrecht gebildeten zuständigen Ethik-Kommission können Sie das verkürzte Antragsformular verwenden, abzurufen unter: http://www.ethik.med.uni-erlangen.de *(Anschlussvotum)*

Antrag auf Beurteilung eines Forschungsprojektes (keine Arzneimittelprüfung)

bitte 9-fach einschließlich Anlagen einreichen sowie 1-mal in elektronischer Fassung

Titel des Projektes:
Einfluss eines 16-wöchigen Kraft-Trainingsprogramms auf leistungsphysiologische und gesundheitsrelevante muskuläre und kardiale Größen bei untrainierten Männern im mittleren Lebensalter. Eine randomisierte kontrollierte Interventionsstudie mit modernen, bildgebenden Verfahren.

I. Projektleitung

1. Name der/des verantwortlichen Projektleiterin/s an der FAU:
Professor Dr. Wolfgang Kemmler[1], Osteoporose-Forschungszentrum, Institut für Medizinische Physik, FAU (Direktor: Professor Dr. Dr. Willi A. Kalender)
App.-Nr. 23999; E-Mail: wolfgang.kemmler@imp.uni-erlangen.de

Angaben über die Qualifikation der/des Versuchsleiterin/s:
Promotion, Habilitation (liegen bereits vor)

2. a) Weitere Teilnehmer/innen vor Ort (alphabetische Reihenfolge):
Prof. Dr. Klaus Engelke, IMP, FAU
PD. Dr. Michael Lell, Radiologisches Institut, FAU
Prof. Dr. Harald Quick, IMP, FAU
PD. Dr. Axel Schmid, Radiologisches Institut, FAU
Dr. Michael Scharf, Radiologisches Institut, FAU
Dr. Simon von Stengel, Osteoporoseforschungszentrum, FAU
Andreas Wittke, IMP, FAU

b) Weitere Prüfzentren (bei multizentrischen Studien): keine

3. Handelt es sich bei diesem Antrag um ein bereits von der Ethik-Kommission der Medizinischen Fakultät der Friedrich-Alexander-Universität Erlangen-Nürnberg begutachtetes Projekt?

ja (bitte lfd. Nr. angeben) ☒ nein

II. Forschungsvorhaben

1. Geplanter Beginn der Studie: September 2012 // voraussichtliches Ende: August 2013
Dauer der Studienteilnahme für den einzelnen Probanden:

Intervention (s.u.): 4 Monate // 8 Monate (Kontrollgruppen-Wartelisten-Design)
Untersuchung (s.u.): ca. 100 min jeweils zu Beginn und Studienende

2. Kurzer Abriss des Projektes *(maximal 1,5 Seiten)*:

Einführung und Fragestellung

Krafttraining gilt auch bei intensiver Durchführung als gesundheitsfördernde Intervention, die sich bei einem zunehmenden Anteil der Bevölkerung immer größerer Beliebtheit erfreut. Neben Körperformung und Attraktivität steht für viele Menschen der präventive Aspekt des Muskeltrainings im Vordergrund. Tatsächlich weisen eine Vielzahl von Untersuchungen (1-5) positive Effekte eines regelmäßig durchgeführten, „überschwelligen" Trainings nach, so auf gesundheitsrelevante muskuläre, physiologische, metabolische und kardiovaskuläre Parameter wie beispielsweise die Körperzusammensetzung/funktionelle Kapazität, Blutfette/Lipoproteine oder Glucoseintoleranz/ Insulinsensitivität. Zudem ist anzunehmen, dass auch relativ rasche funktionelle und morphologische Anpassungserscheinungen des Herzens nachweisbar sind (6, 7). Allerdings liegen keine kernspintomographischen Längsschnittuntersuchungen vor, welche die physiologischen Adaptationserscheinungen des Herzens nach einem mehrmonatigen intensiven Krafttraining bei inaktiven Personen evaluieren. Parallel dazu sind die vorliegenden Methoden, mit denen die Körperzusammensetzung und die muskuläre Massenentwicklung bislang evaluiert wurden, als suboptimal einzuschätzen. Um eine valide und reliable Erfassung der Veränderung muskulärer, physiologischer und kardiologischer Größen als Reaktion auf ein gesundheitsorientiertes Muskeltraining zu gewährleisten, ist der messmethodische Schwerpunkt der vorliegenden Untersuchung auf den Einsatz moderner bildgebender Verfahren (Kernspintomographie (MRT), Computertomographie (CT), Dual Energy X-Ray Absorptiometry (DXA)) und moderner Segmentierungs- und Quantifizierungssoftware gerichtet.
Ziel der Untersuchung ist somit die Evaluierung der Effekte eines intensiven 16-wöchigen Krafttrainingsprogramms auf leistungsphysiologische und gesundheitsrelevante muskuläre und kardiale Größen bei untrainierten Männern im mittleren Lebensalter unter besonderer Berücksichtigung bildgebender Verfahren.

Design:

Randomisierte, kontrollierte, teilverblindete Studie mit Wartegruppe (cross-over)

Endpunkte

Primäre Endpunkte:

- Muskelquerschnitt der Oberschenkelmuskulatur (MRT, CT)
- Intraabdominale Fettmasse (MRT)

Sekundäre Endpunkte:

- Kraftfähigkeiten (u.a. abhängige Variable) [1]
- Gesamtkörperfett und Muskelmasse sowie regionale Verteilung (DXA, MRT)
- Metabolisches Syndrom-Score (8), und 10 Jahres-CHD-Risiko (9)
- Kardiale Masse und enddiastolisches Volumen (MRT)
- Hormonelle Regulation (u.a. freies Testosteron, hGH, Cortisol)
- Schmerzintensität, Quality of Life

[1] Ein Ziel der Studie ist die Identifikation von Variablen, welche die Varianz des Kraft-/Leistungszuwachs am höchsten aufklären.

Stichprobe

Zwei-Gruppen Design, randomisiert, z.T. cross-over (mit Wartegruppe)[2]

- Gruppe 1, n = 40: High Intensity Resistance Training (HIT)[3]
- Gruppe 2, n = 40: Kontrollgruppe (Wartegruppe; im Anschluss „Power-Training[4]“)

Interventionsprogramm

Gruppe 1: HIT mit konventioneller Bewegungsgeschwindigkeit (TUT[5] 2s-1s-2s)

Periodisiertes, progressives Krafttraining über 16 Wochen, basierend auf individuellen Trainingsplänen auf der Basis von 1 RM/x RM-Tests (1, 4, 10 Wochen), zunehmende Intensivierung der Reizhöhe (bis Woche 8), anschließend periodisiertes HIT (70-92,5%, 1RM) mit 2-3 Trainingseinheiten je Woche (1-2 gemeinsame, überwachte Trainingseinheiten; 1-2 Trainingseinheiten in Eigenregie (Vorgaben über Trainingsplan)).

Gruppe 2 (ehemalige Kontrollgruppe): Power-Training (TUT: ϟ-1s-2s)

s.o. aber explosive Ausführung im konzentrischen Bewegungsbereich nach initialer Konditionierung über 6 Wochen. Vergleichbares Trainingsprotokoll, allerdings im Intensitätsbereich von 40-70%, 1RM.

3. Studienbezogene Maßnahmen:

Bitte beschreiben Sie hier alle Maßnahmen, die studienbedingt durchgeführt werden sowie alle erforderlichen Abweichungen von der üblichen Routine-Behandlung:

Interventionsprogramm s.o.

Messungen (jeweils basal und nach 16 Wochen)

Bildgebende Verfahren:

Ganzkörper-MRT und Ganzkörper-DXA zur Erfassung der gesamten und regionalen Körperzusammensetzung.

Lokale MRT und CT am Oberschenkel (über „Muskelbauch“)

Kardiale MRT-Untersuchung mit Kontrastmittel i.v. (Analyse fibrotischer Herzmuskelveränderungen, Strain der Herzmuskulatur, enddiastolisches Volumen (EDV), endsystolisches Volumen (ESV), Schlagvolumen (SV), Ejektionsfraktion (EF) und myokardiale Masse (MM), Herzmuskeldicke).

Blutdruck und Herzfrequenzverhalten in Ruhe

Körperliche Fitness:

Erfassung unterschiedlicher Kraftfähigkeiten (u.a. 1 RM, Schnellkraft) mittels isokinetischem Dynamometer.

Psychosoziale Parameter

Befindlichkeit und Schmerzparameter, QoL (Fragebogen)

Labor

Blutfette/Lipoproteine, Glukose, Insulin, HbAC1, Entzündungsmarker

Testosteron, freies Testosteron, hGH, Cortisol; (evt. noch Parameter des Immunsystems)

Ernährungsanalyse

Analyse über 4 Tage (standardisierte Protokolle)

Anamnese und Risikofaktorenprofil über Fragebogen

---

[2] Die Wartegruppe dient zunächst als parallele Kontrollgruppe zur Interventionsgruppe. Nach Abschluss dieses Untersuchungsabschnittes wird mit dieser Gruppe ebenfalls eine 16-wöchige Intervention durchgeführt, sodass ein eingeschränktes 3-Gruppendesign generiert wird.

[3] Training mit relativ hoher Reizintensität (≥70% des Einwiederholungsmaximums: 1RM)

[4] Training mit explosiver Bewegungsausführung im konzentrischen Bereich (bei vglw. geringer Reizintensität im Bereich 40-60% 1RM)

[5] **T**ime **U**nder **T**ension: Dauer der jeweiligen Bewegungsabschnitte, konzentrisch – isometrisch – exzentrisch in Sec.

4. Wird die Studie gemäß der von der 48. Generalversammlung des Weltärztebundes in Somerset West revidierten Deklaration von Helsinki aus dem Jahre 1996 durchgeführt? Bitte angeben, ob alle anderen Erprobungsmöglichkeiten ausgeschöpft wurden.

Ja, die Intervention wurde in vorhergehenden Studien bereits validiert und optimiert. Die Messtechnologie ist ebenfalls etabliert, wurde aber bislang nur suboptimal zur Validierung der hier genannten Fragestellung eingesetzt.

5. Art des Forschungsvorhabens:
Handelt es sich um
eine diagnostische Prüfung?
eine therapeutische Prüfung?
eine Verträglichkeitsprüfung?
☒ einen ausschließlich wissenschaftlichen Versuch?

6. Gesetzliche Grundlagen

a) Handelt es sich um eine Untersuchung, die dazu bestimmt ist, klinische oder pharmakologische Wirkungen von Arzneimitteln zu erforschen oder nachzuweisen oder Nebenwirkungen festzustellen oder die Resorption, die Verteilung, den Stoffwechsel oder die Ausscheidung zu untersuchen, **mit dem Ziel, sich von der Unbedenklichkeit oder Wirksamkeit des Arzneimittels zu überzeugen** (klinische Prüfung eines Arzneimittels nach §§ 40 Arzneimittelgesetz)?

nein

Bitte begründen. Erläuterungen zum Antrag auf Bewertung einer klinischen Arzneimittelprüfung nach § 40 AMG finden Sie unter http://www.ethik.med.uni-erlangen.de/

b) Handelt es sich um eine klinische Prüfung nach § 20 Medizinproduktegesetz (MPG)?
ja ☒ nein
Bitte begründen. Liegt eine CE-Zertifizierung für das Medizinprodukt vor? Werden zusätzlich invasive oder andere belastende Untersuchungen durchgeführt?
Sämtliche Messverfahren sind CE-zertifiziert und entsprechend MPG geprüft und zugelassen. Invasive Untersuchungen werden nicht durchgeführt, mit Ausnahme von Blutentnahmen und einer venösen Kontrastmittelapplikation.

c) Handelt es sich um ein Vorhaben nach § 8 des Gesetzes zur Regelung des Transfusionswesens (TFG)?
ja ☒ nein

7. Handelt es sich um einen Versuch nach

§ 23 Strahlenschutzverordnung? ☒ ja nein

§ 28 Röntgenverordnung? ☒ ja nein

8. Typ der Studie:

- offen
- ☒ blind
- doppelblind
- ☒ vergleichend
- ☒ randomisiert
- multizentrisch
- ☒ Feldstudie
- Pilotstudie

9. Wissenschaftliche Begründung des Projekts, insbesondere:

a. Erläuterung des Versuchsziels

Das Ziel der Untersuchung ist die Evaluierung eines intensiven körperlichen Krafttrainings auf leistungsphysiologische und gesundheitsrelevante muskuläre und kardiale Größen bei untrainierten Männern im mittleren Lebensalter unter besonderer Berücksichtigung moderner bildgebender Verfahren. Aus radiologischer Sicht ist das Ziele der Studie die Identifikation typischer Kenngrößen der physiologischen Adaption der Körperzusammensetzung und des Herz-Kreislauf-Systems nach unterschiedlichen Typen von Krafttraining (s.o.) sowie der Methodenvergleich kernspintomographischer mit computertomographischen Muskelquerschnitten des Oberschenkels sowie der gesamten und der regionalen Gesamtkörperzusammensetzung mittels MRT vs. dem Goldstandard DXA-Methode.

b. Darstellung des bisherigen Wissensstandes

Eine Vielzahl von wissenschaftlichen Untersuchungen berichten den positiven Effekt eines „Krafttrainings“ auf funktionelle und gesundheitsrelevante physiologische und metabolische Größen (Übersicht in (1, 3, 10, 11)) bei Menschen in mittlerem Lebensalter. Obgleich viele dieser Daten bereits in frühen Studien mit suboptimaler Messmethodik/-technik und schlechter Reproduzierbarkeit evaluiert wurden, gelten sie in der wissenschaftlichen Literatur als absolut verlässlich und werden vielfach zitiert. Moderne bildgebende Verfahren wie die Kernspintomographie (MRT) oder die Computertomographie (CT) im Verbund mit valider Segmentierungstechnologie und quantitativer Analyse haben die in der Vergangenheit eingesetzten Verfahren mit suboptimaler Auflösung und ausschließlich qualitativer Auswerteprozedur bereits in vielen Bereichen ersetzt. So können Risikofaktoren wie die Körperfettverteilung derzeit schon quantifiziert und der Einfluss einer Intervention validiert werden (12-14). Auch die muskuläre Komponente der Körperzusammensetzung und insbesondere der Muskelquerschnitt wurde von einigen neueren Untersuchungen mittels moderner bildgebender Verfahren wie MRT oder CT untersucht (15, 16), die allerdings auf keine validierte Segmentierungs- und Quantifizierungssoftware zurückgreifen konnten.

Dies trifft ebenfalls für die kardiale Volumetrie mittels MRT zu, die derzeit als Goldstandard für die links- und rechtsventrikuläre Volumen- und Massenbestimmung gilt (17). Im Gegensatz zu echokardiographischen Untersuchungen handelt es sich bei der kardialen MRT um ein dreidimensionales Verfahren, welches dadurch eine wesentlich genauere morphologische Darstellung des Herzens ermöglicht (18) und somit u.a.

interventionsbedingte Veränderungen früher zu identifizieren vermag. Darüber hinaus werden sportliche Belastungen immer wieder mit einem plötzlichen Herztod, insbesondere bei männlichen Athleten, assoziiert (23-24). Ob hierfür pathologische morphologische Veränderungen des Myokards durch körperliches Training oder angeborene Organerkrankungen zu Grunde liegen, ist unklar.

10. Angaben zur Nutzen-Risiko-Relation

a. Welcher Nutzen ist von den Ergebnissen der Studie zu erwarten

aa) für die Versuchsteilnehmer?

Steigerung der körperlichen Fitness und physischen Attraktivität durch das Trainingsprogramm. Zudem Verminderung des metabolischen und kardiovaskulären Gesundheitsrisikos und Verbesserung der funktionellen Kapazität. Ggf. Detektion relevanter kardiologischer Befunde (Herzklappendefekte, Kardiomyopathie, Fehlbildungen).

ab) für die Heilkunde?

Beschreibung physiologischer muskulärer, metabolischer, physiologischer und kardialer Adaptation nach intensivem Krafttraining u.a. als Voraussetzung zur Abgrenzung pathologischer Veränderungen und Identifizierung geeigneter Verfahren zur Kontrolle von Trainingseffekten. Sekundäres Ziel ist es, Risikopatienten zu identifizieren, die vor Aufnahme eines spezifischen Trainings ein ausführliches Untersuchungsprogramm durchführen sollten, um Sekundärschäden zur vermeiden.

ac) für die Wissenschaft (z.B. Ergebnisse, die nicht unmittelbar therapeutischen Zwecken dienen)?

Verbesserter Einblick in die Grundlagen trainingsinduzierter muskulärer und kardiovaskulärer Adaptation als Basis optimaler sportwissenschaftlicher Trainingsempfehlungen. Evaluierung und Weiterentwicklung von bildgebenden Untersuchungsverfahren und computerunterstützter Evaluationssoftware zur Diagnostik und zum Monitoring von Interventionseffekten.

b. Mit welchem Risiko ist die Studie für die Versuchsteilnehmer verbunden?

ba) Welcher Art sind die Risiken? Risikoeinschätzung, vorhersehbare Risiken der Behandlung und sonstiger studienbedingter Verfahren, die eingesetzt werden sollen (einschließlich Schmerz, Unannehmlichkeiten, Beschwerden, Verletzung der persönlichen Integrität und Maßnahmen zur Vermeidung und/oder zur Behandlung von unvorhersehbaren/ unerwünschten Ereignissen)

Die Risiken der Intervention (konsequent angeleitetes Krafttrainingsprogramm) sind sehr gering und bleiben nicht zuletzt aufgrund der intensiven Betreuung deutlich hinten denen eines selbständig durchgeführten Muskeltrainings zurück. Selbstverständlich sind in den ersten Wochen der Intervention leichte trainingsbedingte Beschwerden wie bspw. DOMS (Muskelkater) zu erwarten.

Die radiologischen Verfahren Dual Energy X-Ray Absorptiometrie (Gesamtkörper-DXA) bzw. Computertomographie (medialer Anteil Oberschenkel) sind mit niedrigen Strahlendosen (< 10 µSv pro DXA-Messung) bzw. geringen relativen Dosen (<0.8 mSv/CT-Messung[6]) verbunden. Eine Genehmigung dieser Verfahren

[6] Dieser Wert wurde ohne Berücksichtigung entsprechender Schutzmaßnahmen (Abdecken der Gonaden mit Bleischutz errechnet.

wird beim Bundesamt für Strahlenschutz nach Vorliegen der Genehmigung der Ethikkommission selbstverständlich beantragt.

Bei der Blutentnahme und durch periphere Verweilkatheter für die Kontrastmittel-(KM)-Applikation sind Blutergüsse und Infektionen nie komplett ausgeschlossen. In der Kernspintomographie werden nur geringe KM-Mengen eingesetzt. Daher sind die Risiken bei einem Paravasat des KMs in das Weichteilgewebe sowie Nierenbelastungen und mögliche allergische KM-Reaktionen minimiert. Probanden mit relativen oder absoluten Kontraindikationen für eine MRT werden von der Studie ausgeschlossen (s.u.). Insbesondere Probanden mit Nierenfunktionsstörungen oder bekannten KM-Reaktionen. Die KM-Gabe ist notwendig, um relevante kardiale Vorerkrankungen auszuschließen (siehe Ausschlusskriterien), die ein Risiko für die Intervention darstellen können.

bb) Mit welcher Wahrscheinlichkeit ist zu erwarten, daß sich die Risiken realisieren? Wie sicher ist die Wahrscheinlichkeit abschätzbar?

Insgesamt besteht nur eine geringe Wahrscheinlichkeit, dass sich die Risiken realisieren. Die Messungen stellen Standardmessungen der klinischen Routine dar, die von geschultem Fachpersonal sachkundig ausgeführt werden. Die Belastungsvorgaben erfolgen individuell, basierend auf einer Leistungsdiagnostik, sodass eine Überforderung der Teilnehmer kaum zu erwarten ist.

c. Warum ist das mögliche Risiko im Verhältnis zu dem zu erwartenden Nutzen Ihrer Ansicht nach vertretbar?

Es besteht keine wesentlich über das Alltagsrisiko hinausgehende Gefährdung, jedoch ein hoher zu erwartender Nutzen für die Teilnehmer hinsichtlich der Steigerung der körperlichen Fitness, gesundheitsrelevanter Größen, Wohlbefinden, Attraktivität und Selbstwirksamkeit/Kontrollüberzeugung.

d. Werden Zwischenergebnisse ausgewertet, um einen Trend zu erkennen?
ja ☒ nein, kurzer Interventionszeitraum macht Zwischenanalyse inadäquat.

e. Sind Kriterien festgelegt worden, bei deren Eintreten der Versuch geändert oder abgebrochen werden soll? ja, welche? ☒ nein

(allerdings Abbruch des jeweiligen Testverfahrens bei Unwohlsein, oder generell auf Wunsch des Patienten)

11. Bei klinischen Prüfungen nach MPG:

a. Welches Medizinprodukt soll geprüft werden? **entfällt**

b. Wird die klinische Prüfung von einer entsprechend qualifizierten und befugten Person geleitet, die mindestens eine zweijährige Erfahrung in der klinischen Prüfung von Medizinprodukten nachweisen kann? ja nein

c. Wurde (soweit erforderlich) eine dem jeweiligen Stand der wissenschaftlichen Erkenntnisse entsprechende biologische Sicherheitsprüfung oder sonstige für die vorgesehene Zweckbestimmung des Medizinproduktes erforderliche Prüfung durchgeführt? ja nein

d. Wurde (soweit erforderlich) die sicherheitstechnische Unbedenklichkeit für die Anwendung des Medizinproduktes unter Berücksichtigung des Standes der Technik sowie der Arbeitsschutz- und Unfallverhütungsvorschriften nachgewiesen?
ja nein

e. Ist der Leiter der klinischen Prüfung über die Ergebnisse der biologischen Sicherheitsprüfung und die voraussichtlich mit der klinischen Prüfung verbundenen Risiken informiert worden? ja nein

12. a) Ist die Mitarbeit eines Statistikers vorgesehen? ☒ ja nein
b) Welche statistischen Methoden sollen benutzt werden?

Komplettes statistisches Instrumentarium zur Erfassung von Effekten (bspw. Varianzanalysen/nicht parametrische Tests zur Identifikation von Zwischengruppenunterschieden). Zudem regressionsanalytische Modelle zur Aufklärung von Varianzen.

13. a) Handelt es sich um eine multizentrische Studie (d.h. eine nach einem *einzigen* Prüfplan durchgeführte Studie, die in mehr als einer Prüfstelle erfolgt und daher von mehr als einem Prüfer vorgenommen wird)? ja ☒ nein

b) Wurden/Werden an anderer Stelle Studien mit demselben oder einem ähnlichen Ziel durchgeführt? ja, wo? ☒ nein

Es wurden in der Vergangenheit bereits mehrere Untersuchungen mit dem Ziel der Evaluierung eines gesundheitssportlichen Trainings auf muskuläre, physiologische, metabolische und kardiale Parameter (meist isoliert) durchgeführt (s.o.). Diese Studie ist jedoch die erste, die u.a. den Effekt eines intensiven Kraft-/Powertrainings auf muskuläre Parameter, Körperzusammensetzung, metabolische und kardiovaskuläre Größen bei untrainierten Männern in mittlerem Lebensalter auch mittels moderner bildgebender Verfahren und Auswertesoftware evaluiert.

14. Wer hat die Studie initiiert? **Institut für Medizinische Physik**
15. Wer finanziert sie? (*Bitte geben Sie an, ob Drittmittel von nichtöffentlicher Seite beantragt werden. Falls ja, in welcher Höhe?*)

Derzeit sind noch keine Mittel beantragt, es werden jedoch nach positivem Votum der Ethikkommission versucht über unterschiedliche Ebenen Drittmittel zu generieren.

16. Die Aufwandsentschädigung wird übernommen von (*bitte Ansprechpartner benennen*):

Institut für Medizinische Physik (Direktor: Prof. Dr. Dr. W.A. Kalender)

Ansprechpartner: Prof. Dr. Wolfgang Kemmler

III. Angaben zu den Versuchsteilnehmern

1. Anzahl (*bei vergleichenden Studien bitte Aufteilung auf Gruppen angeben*)

80 Personen gesamt:

- Gruppe 1, n = 40: High Intensity Resistance Training (HIT)[7]
- Gruppe 2, n = 40: Kontrollgruppe (Wartegruppe; im Anschluss „Power-Training")

Bei Nullhypothesen-basierten Studien:
Wurde eine formale Fallzahlschätzung vorgenommen?

☒ ja, Basis CSA-Oberschenkelmuskulatur nein

2. Alter und Geschlecht (*bitte geben Sie das Alter der Versuchsteilnehmer sowie die als Ausschlusskriterien vorgesehenen Ober- und Untergrenzen an*)

[7] Training mit relativ hoher Reizintensität (≥70% des Einwiederholungsmaximums: 1RM)

Männer, 30. - 50. Lebensjahr

3. Status: Handelt es sich bei den Versuchsteilnehmern um
   ☒ gesunde Personen
   schwangere oder stillende Frauen
   Kinder oder Jugendliche
   einschlägig Erkrankte (*bitte geben Sie die Krankheit und das Stadium an*)
   Personen, die an anderen Krankheiten leiden? (Insbesondere: psychische Krankheiten, die Zweifel an der Geschäfts- oder Einsichtsfähigkeit begründen)

4. Welche sonstigen **Einschlusskriterien** (z.B. erlaubte Begleitmedikation) sind vorgesehen?

   - initial Untrainierte (während der vergangenen 2 Jahre: ≤1 h/Woche Sport mit positivem Effekt auf die Muskulatur; ≤2 h/Woche Sport gesamt)

5. Welche sonstigen **Ausschlusskriterien** (z.B. fortgeschrittene Nieren- oder Leberinsuffizienz, verbotene Begleitmedikation etc.) sind vorgesehen?
   - Geschichte leistungssportlicher Ausübung von Disziplinen mit erheblicher Relevanz für Körperzusammensetzung und Kraftfähigkeiten
   - pathologische muskuläre, metabolische und kardiale Veränderungen oder Entzündungen; deutlich eingeschränkte Gelenksbeweglichkeit in Knie und Hüfte.
   - Medikamente/Erkrankungen mit relevantem Einfluss auf Körperzusammensetzung und Herz-Kreislauf-System
   - sehr geringe körperliche Leistungsfähigkeit (< 100 Watt auf dem Fahrradergometer)
   - schwere Adipositas (BMI > 35 kg/m^2)
   - Abwesenheit ≥ 2 Wochen während des Interventionszeitraums
   - Geplante Aufnahme einer relevanten parallelen Trainingsmaßnahme
   - Kontraindikationen gegen MRT (Klaustrophobie, Herzschrittmacher, magnetisierbare intracorporale Fremdkörper); Körpermaße die eine MRT-Messung verhindern
   - Drogenmissbrauch

6. Sollen auch Personen teilnehmen, die auf gerichtliche oder behördliche Anordnung in einer Anstalt verwahrt werden?
   ja ☒ nein

7. Sollen auch Personen teilnehmen, die sich schon für andere Forschungsvorhaben zur Verfügung gestellt haben?
   ja ☒ nein
   wie lange muss die letzte Teilnahme zurückliegen?

8. Bei Studien an Minderjährigen (oder sonst nicht geschäftsfähigen Personen) **entfällt**
   a. Warum kann die Studie nicht an Erwachsenen (voll Geschäftsfähigen) durchgeführt werden?

   b. Sind Aufklärung und Einwilligung der (des) gesetzlichen Vertreter(s) gewährleistet? (*bitte vorformulierte Erklärung beifügen*)
   ja nein, weil

c. Sind zusätzliche Aufklärung und Einwilligung der minderjährigen (nicht voll geschäftsfähigen) Versuchsteilnehmer gewährleistet, die selbst in der Lage sind, Wesen, Bedeutung und Tragweite des Versuchs einzusehen und ihren Willen danach zu bestimmen?

ja nein

9. Probandenversicherung

Wird zugunsten der Versuchsteilnehmer eine Versicherung abgeschlossen?

ja (*bitte Police beifügen, aus der die Versicherungsgesellschaft und die Höhe der Versicherungsleistung hervorgeht)*

☒ nein

10. Schweigepflicht/Datenschutz

Werden die ärztlichen Schweigepflicht- und die Datenschutzbestimmungen beachtet?

ja

11. Entgelt für Probanden

Soll den Versuchsteilnehmern ein Entgelt (Aufwandsentschädigung o.ä.) gezahlt werden?

ja, in Höhe von EUR ☒ nein

12. Wie sollen die Versuchsteilnehmer über Wesen, Bedeutung und Tragweite der Studie **aufgeklärt** werden?

Bitte in deutscher Sprache beifügen:

Dokumentation des Inhalts der Patientenaufklärung durch die/den versuchsdurchführende/n Ärztin/Arzt (Merkblatt), insbesondere mit Hinweisen über:

- **Ziele und Methoden** der Studie;
- **Nutzen und Risiko** der Studie;
- bekannte und möglicherweise zu erwartende **Wirkungen und Nebenwirkungen** von Medikamenten;
- Eingriffe, die nur aus wissenschaftlichen Gründen erfolgen;
- ein angebrachtes Verhalten des Patienten während und nach dem Versuch;
- die **Widerruflichkeit** einer Einwilligung;
- das Bestehen und den Umfang der gesetzlichen **Probandenversicherung** (Name/Anschrift/Telefon/Fax der Versicherungsgesellschaft, Nummer der Versicherungspolice) sowie die danach von der Versuchsperson zu beachtenden Obliegenheiten;
- **Ausschlusskriterien** (z.B. Schwangerschaft/Stillzeit);
- Name und Telefon des **Ansprechpartner**s vor Ort.
- **Besondere Aufklärung** über die Situation
  a. bei der randomisierten Studie
  f. beim Blind- und Doppelblindversuch.

13. Wie sollen die Versuchsteilnehmer ihre **Einwilligung** in die Teilnahme an der Studie erklären? (*bitte formulierte deutschsprachige Erklärung mit datenschutzrechtlicher Einwilligungserklärung beifügen*)

Schriftliche Einverständniserklärung nach umfangreicher mündlicher und schriftlicher Aufklärung über Ziele, Nutzen und Risiken der Untersuchung.

Ich weiß, daß auch bei einer positiven Beurteilung des Vorhabens durch die Ethik-Kommission der Medizinischen Fakultät der FAU Erlangen-Nürnberg die ärztliche und juristische Verantwortung für die Durchführung des Projektes uneingeschränkt bei der Leiterin/dem Leiter verbleibt.

Erlangen/Nürnberg
Datum ....

Unterschrift des/der Antragstellers/in

____
(Name in Druckbuchstaben)

Unterschrift der/des Leiterin/Leiters der Einrichtung, in der das Vorhaben durchgeführt werden soll.
Mit der Durchführung des Forschungsvorhabens einverstanden:

Datum ....

Unterschrift des/der Leiters/Leiterin der Einrichtung

____
(Name in Druckbuchstaben)

EK_May09/mit Unterschriftsblatt

Literatur

1. Benson AC, Torode ME, Fiatarone Singh MA. Effects of resistance training on metabolic fitness in children and adolescents: a systematic review. *Obes Rev*. 2008;9(1):43-66.
2. Kelley GA, Kelley KS. Impact of progressive resistance training on lipids and lipoproteins in adults: a meta-analysis of randomized controlled trials. *Prev Med*. 2009;48(1):9-19.
3. Latham NK, Bennett DA, Stretton CM, Anderson CS. Systematic review of progressive resistance strength training in older adults. *J Gerontol A Biol Sci Med Sci*. 2004;59(1):48-61.
4. Macaluso A, De Vito G. Muscle strength, power and adaptations to resistance training in older people. *Eur J Appl Physiol*. 2004;91:450-472.
5. Snowling NJ, Hopkins WG. Effects of different modes of exercise training on glucose control and risk factors for complications in type 2 diabetic patients: a meta-analysis. *Diabetes Care*. 2006;29(11):2518-27.
6. Weineck J. *Optimales Training* Erlangen: Spitta-Verlag; 2007.
7. Weineck J. *Sportbiologie*. Vol. 10 Balingen: Spitta Verlag; 2009.
8. Wijndaele K, Beunen G, Duvigneaud N, et al. A continuous metabolic syndrome risk score: utility for epidemiological analyses. *Diabetes Care*. 2006;29(10):2329.
9. Wilson PW, D'Agostino RB, Levy D, Belanger AM, Silbershatz H, Kannel WB. Prediction of coronary heart disease using risk factor categories. *Circulation*. 1998;97(18):1837-47.
10. Asikainen TM, Kukkonen-Harjula K, Miilunpalo S. Exercise for health for early postmenopausal women: a systematic review of randomised controlled trials. *Sports Med*. 2004;34(11):753-78.
11. Lagally KM, Cordero J, Good J, Brown DD, McCaw ST. Physiologic and metabolic responses to a continuous functional resistance exercise workout. *J Strength Cond Res*. 2009;23(2):373-9.
12. Kay SJ, Fiatarone Singh MA. The influence of physical activity on abdominal fat: a systematic review of the literature. *Obes Rev*. 2006;7(2):183-200.
13. Kemmler W, von Stengel S, Engelke K, Haberle L, Mayhew JL, Kalender WA. Exercise, body composition, and functional ability: a randomized controlled trial. *Am J Prev Med*. 2010;38(3):279-87.
14. Lamb HJ. Total body fat distribution as part of multiorgan MR imaging: new tool for risk assessment in the metabolic syndrome? *Radiology*. 2010;257(2):307-8.
15. Weiss EP, Racette SB, Villareal DT, et al. Lower extremity muscle size and strength and aerobic capacity decrease with caloric restriction but not with exercise-induced weight loss. *J Appl Physiol*. 2007;102(2):634-40.
16. Valtonen A, Poyhonen T, Sipila S, Heinonen A. Effects of aquatic resistance training on mobility limitation and lower-limb impairments after knee replacement. *Arch Phys Med Rehabil*;91(6):833-9.
17. Petersen SE, Hudsmith LE, Robson MD, et al. Sex-specific characteristics of cardiac function, geometry, and mass in young adult elite athletes. *J Magn Reson Imaging*. 2006;24(2):297-303.
18. Grothues F, Smith GC, Moon JC, et al. Comparison of interstudy reproducibility of cardiovascular magnetic resonance with two-dimensional echocardiography in normal subjects and in patients with heart failure or left ventricular hypertrophy. *Am J Cardiol*. 2002;90(1):29-34.

An die Geschäftsstelle der
Ethik-Kommission
der Medizinischen Fakultät
der FAU Erlangen-Nürnberg
Krankenhausstr. 12
91054 Erlangen

Antrag
an die Ethik-Kommission
der Medizinischen Fakultät

*Bitte **in deutscher Sprache** ausfüllen,*
Zutreffendes bitte ankreuzen.
Für multizentrische Studien mit Vorvotum einer nach Landesrecht gebildeten zuständigen Ethik-Kommission können Sie das verkürzte Antragsformular verwenden, abzurufen unter: http://www.ethik.med.uni-erlangen.de *(Anschlussvotum)*

Antrag auf Beurteilung eines Forschungsprojektes (keine Arzneimittelprüfung)

bitte 9-fach einschließlich Anlagen einreichen sowie 1-mal in elektronischer Fassung

**Titel des Projektes:**
Einfluss eines 16-wöchigen Kraft-Trainingsprogramms auf leistungsphysiologische und gesundheitsrelevante muskuläre und kardiale Größen bei untrainierten Männern im mittleren Lebensalter. Eine randomisierte kontrollierte Interventionsstudie mit modernen, bildgebenden Verfahren.

I. Projektleitung

1. Name der/des verantwortlichen Projektleiterin/s an der FAU:
Professor Dr. Wolfgang Kemmler[1], Osteoporose-Forschungszentrum, Institut für Medizinische Physik, FAU (Direktor: Professor Dr. Dr. Willi A. Kalender)
App.-Nr. 23999; E-Mail: wolfgang.kemmler@imp.uni-erlangen.de

Angaben über die Qualifikation der/des Versuchsleiterin/s:
Promotion, Habilitation (liegen bereits vor)

2. a) Weitere Teilnehmer/innen vor Ort (alphabetische Reihenfolge):
Prof. Dr. Klaus Engelke, IMP, FAU
PD. Dr. Michael Lell, Radiologisches Institut, FAU
Prof. Dr. Harald Quick, IMP, FAU
PD. Dr. Axel Schmid, Radiologisches Institut, FAU
Dr. Michael Scharf, Radiologisches Institut, FAU
Dr. Simon von Stengel, Osteoporoseforschungszentrum, FAU
Andreas Wittke, IMP, FAU

b) Weitere Prüfzentren (bei multizentrischen Studien): keine

3. Handelt es sich bei diesem Antrag um ein bereits von der Ethik-Kommission der Medizinischen Fakultät der Friedrich-Alexander-Universität Erlangen-Nürnberg begutachtetes Projekt?

ja (bitte lfd. Nr. angeben) ☒ nein

II. Forschungsvorhaben

1. Geplanter Beginn der Studie: September 2012 // voraussichtliches Ende: August 2013
Dauer der Studienteilnahme für den einzelnen Probanden:

Intervention (s.u.): 4 Monate // 8 Monate (Kontrollgruppen-Wartelisten-Design)
Untersuchung (s.u.): ca. 100 min jeweils zu Beginn und Studienende

2. Kurzer Abriss des Projektes *(maximal 1,5 Seiten)*:

Einführung und Fragestellung
Krafttraining gilt auch bei intensiver Durchführung als gesundheitsfördernde Intervention, die sich bei einem zunehmenden Anteil der Bevölkerung immer größerer Beliebtheit erfreut. Neben Körperformung und Attraktivität steht für viele Menschen der präventive Aspekt des Muskeltrainings im Vordergrund. Tatsächlich weisen eine Vielzahl von Untersuchungen (1-5) positive Effekte eines regelmäßig durchgeführten, „überschwelligen" Trainings nach, so auf gesundheitsrelevante muskuläre, physiologische, metabolische und kardiovaskuläre Parameter wie beispielsweise die Körperzusammensetzung/funktionelle Kapazität, Blutfette/Lipoproteine oder Glucoseintoleranz/ Insulinsensitivität. Zudem ist anzunehmen, dass auch relativ rasche funktionelle und morphologische Anpassungserscheinungen des Herzens nachweisbar sind (6, 7). Allerdings liegen keine kernspintomographischen Längsschnittuntersuchungen vor, welche die physiologischen Adaptationserscheinungen des Herzens nach einem mehrmonatigen intensiven Krafttraining bei inaktiven Personen evaluieren. Parallel dazu sind die vorliegenden Methoden, mit denen die Körperzusammensetzung und die muskuläre Massenentwicklung bislang evaluiert wurden, als suboptimal einzuschätzen. Um eine valide und reliable Erfassung der Veränderung muskulärer, physiologischer und kardiologischer Größen als Reaktion auf ein gesundheitsorientiertes Muskeltraining zu gewährleisten, ist der messmethodische Schwerpunkt der vorliegenden Untersuchung auf den Einsatz moderner bildgebender Verfahren (Kernspintomographie (MRT), Computertomographie (CT), Dual Energy X-Ray Absorptiometry (DXA)) und moderner Segmentierungs- und Quantifizierungssoftware gerichtet.
Ziel der Untersuchung ist somit die Evaluierung der Effekte eines intensiven 16-wöchigen Krafttrainingsprogramms auf leistungsphysiologische und gesundheitsrelevante muskuläre und kardiale Größen bei untrainierten Männern im mittleren Lebensalter unter besonderer Berücksichtigung bildgebender Verfahren.

Design:
Randomisierte, kontrollierte, teilverblindete Studie mit Wartegruppe (cross-over)

Endpunkte
Primäre Endpunkte:

- Muskelquerschnitt der Oberschenkelmuskulatur (MRT, CT)
- Intraabdominale Fettmasse (MRT)

Sekundäre Endpunkte:

- Kraftfähigkeiten (u.a. abhängige Variable) [1]
- Gesamtkörperfett und Muskelmasse sowie regionale Verteilung (DXA, MRT)
- Metabolisches Syndrom-Score (8), und 10 Jahres-CHD-Risiko (9)
- Kardiale Masse und enddiastolisches Volumen (MRT)
- Hormonelle Regulation (u.a. freies Testosteron, hGH, Cortisol)
- Schmerzintensität, Quality of Life

[1] Ein Ziel der Studie ist die Identifikation von Variablen, welche die Varianz des Kraft-/Leistungszuwachs am höchsten aufklären.

Stichprobe

Zwei-Gruppen Design, randomisiert, z.T. cross-over (mit Wartegruppe)[2]

- Gruppe 1, n = 40: High Intensity Resistance Training (HIT)[3]
- Gruppe 2, n = 40: Kontrollgruppe (Wartegruppe; im Anschluss „Power-Training[4]“)

Interventionsprogramm

Gruppe 1: HIT mit konventioneller Bewegungsgeschwindigkeit (TUT[5] 2s-1s-2s)

Periodisiertes, progressives Krafttraining über 16 Wochen, basierend auf individuellen Trainingsplänen auf der Basis von 1 RM/x RM-Tests (1, 4, 10 Wochen), zunehmende Intensivierung der Reizhöhe (bis Woche 8), anschließend periodisiertes HIT (70-92,5%, 1RM) mit 2-3 Trainingseinheiten je Woche (1-2 gemeinsame, überwachte Trainingseinheiten; 1-2 Trainingseinheiten in Eigenregie (Vorgaben über Trainingsplan)).

Gruppe 2 (ehemalige Kontrollgruppe): Power-Training (TUT: ϟ-1s-2s)

s.o. aber explosive Ausführung im konzentrischen Bewegungsbereich nach initialer Konditionierung über 6 Wochen. Vergleichbares Trainingsprotokoll, allerdings im Intensitätsbereich von 40-70%, 1RM.

3. Studienbezogene Maßnahmen:

Bitte beschreiben Sie hier alle Maßnahmen, die studienbedingt durchgeführt werden sowie alle erforderlichen Abweichungen von der üblichen Routine-Behandlung:

Interventionsprogramm s.o.

Messungen (jeweils basal und nach 16 Wochen)

Bildgebende Verfahren:

Ganzkörper-MRT und Ganzkörper-DXA zur Erfassung der gesamten und regionalen Körperzusammensetzung.

Lokale MRT und CT am Oberschenkel (über „Muskelbauch“)

Kardiale MRT-Untersuchung mit Kontrastmittel i.v. (Analyse fibrotischer Herzmuskelveränderungen, Strain der Herzmuskulatur, enddiastolisches Volumen (EDV), endsystolisches Volumen (ESV), Schlagvolumen (SV), Ejektionsfraktion (EF) und myokardiale Masse (MM), Herzmuskeldicke).

Blutdruck und Herzfrequenzverhalten in Ruhe

Körperliche Fitness:

Erfassung unterschiedlicher Kraftfähigkeiten (u.a. 1 RM, Schnellkraft) mittels isokinetischem Dynamometer.

Psychosoziale Parameter

Befindlichkeit und Schmerzparameter, QoL (Fragebogen)

Labor

Blutfette/Lipoproteine, Glukose, Insulin, HbAC1, Entzündungsmarker

Testosteron, freies Testosteron, hGH, Cortisol; (evt. noch Parameter des Immunsystems)

Ernährungsanalyse

Analyse über 4 Tage (standardisierte Protokolle)

Anamnese und Risikofaktorenprofil über Fragebogen

---

[2] Die Wartegruppe dient zunächst als parallele Kontrollgruppe zur Interventionsgruppe. Nach Abschluss dieses Untersuchungsabschnittes wird mit dieser Gruppe ebenfalls eine 16-wöchige Intervention durchgeführt, sodass ein eingeschränktes 3-Gruppendesign generiert wird.

[3] Training mit relativ hoher Reizintensität (≥70% des Einwiederholungsmaximums: 1RM)

[4] Training mit explosiver Bewegungsausführung im konzentrischen Bereich (bei vglw. geringer Reizintensität im Bereich 40-60% 1RM)

[5] **T**ime **U**nder **T**ension: Dauer der jeweiligen Bewegungsabschnitte, konzentrisch – isometrisch – exzentrisch in Sec.

4. Wird die Studie gemäß der von der 48. Generalversammlung des Weltärztebundes in Somerset West revidierten Deklaration von Helsinki aus dem Jahre 1996 durchgeführt? Bitte angeben, ob alle anderen Erprobungsmöglichkeiten ausgeschöpft wurden.

Ja, die Intervention wurde in vorhergehenden Studien bereits validiert und optimiert. Die Messtechnologie ist ebenfalls etabliert, wurde aber bislang nur suboptimal zur Validierung der hier genannten Fragestellung eingesetzt.

5. Art des Forschungsvorhabens:
Handelt es sich um
eine diagnostische Prüfung?
eine therapeutische Prüfung?
eine Verträglichkeitsprüfung?
☒ einen ausschließlich wissenschaftlichen Versuch?

6. Gesetzliche Grundlagen

a) Handelt es sich um eine Untersuchung, die dazu bestimmt ist, klinische oder pharmakologische Wirkungen von Arzneimitteln zu erforschen oder nachzuweisen oder Nebenwirkungen festzustellen oder die Resorption, die Verteilung, den Stoffwechsel oder die Ausscheidung zu untersuchen, **mit dem Ziel, sich von der Unbedenklichkeit oder Wirksamkeit des Arzneimittels zu überzeugen** (klinische Prüfung eines Arzneimittels nach §§ 40 Arzneimittelgesetz)?

nein

Bitte begründen. Erläuterungen zum Antrag auf Bewertung einer klinischen Arzneimittelprüfung nach § 40 AMG finden Sie unter http://www.ethik.med.uni-erlangen.de/

b) Handelt es sich um eine klinische Prüfung nach § 20 Medizinproduktegesetz (MPG)?
ja ☒ nein
Bitte begründen. Liegt eine CE-Zertifizierung für das Medizinprodukt vor? Werden zusätzlich invasive oder andere belastende Untersuchungen durchgeführt?
Sämtliche Messverfahren sind CE-zertifiziert und entsprechend MPG geprüft und zugelassen. Invasive Untersuchungen werden nicht durchgeführt, mit Ausnahme von Blutentnahmen und einer venösen Kontrastmittelapplikation.

c) Handelt es sich um ein Vorhaben nach § 8 des Gesetzes zur Regelung des Transfusionswesens (TFG)?
ja ☒ nein

7. Handelt es sich um einen Versuch nach
§ 23 Strahlenschutzverordnung? ☒ ja nein
§ 28 Röntgenverordnung? ☒ ja nein

8. Typ der Studie:
- offen
- ☒ blind
- doppelblind
- ☒ vergleichend
- ☒ randomisiert
- multizentrisch
- ☒ Feldstudie
- Pilotstudie

9. Wissenschaftliche Begründung des Projekts, insbesondere:
a. Erläuterung des Versuchsziels

Das Ziel der Untersuchung ist die Evaluierung eines intensiven körperlichen Krafttrainings auf leistungsphysiologische und gesundheitsrelevante muskuläre und kardiale Größen bei untrainierten Männern im mittleren Lebensalter unter besonderer Berücksichtigung moderner bildgebender Verfahren. Aus radiologischer Sicht ist das Ziele der Studie die Identifikation typischer Kenngrößen der physiologischen Adaption der Körperzusammensetzung und des Herz-Kreislauf-Systems nach unterschiedlichen Typen von Krafttraining (s.o.) sowie der Methodenvergleich kernspintomographischer mit computertomographischen Muskelquerschnitten des Oberschenkels sowie der gesamten und der regionalen Gesamtkörperzusammensetzung mittels MRT vs. dem Goldstandard DXA-Methode.

b. Darstellung des bisherigen Wissensstandes

Eine Vielzahl von wissenschaftlichen Untersuchungen berichten den positiven Effekt eines „Krafttrainings“ auf funktionelle und gesundheitsrelevante physiologische und metabolische Größen (Übersicht in (1, 3, 10, 11)) bei Menschen in mittlerem Lebensalter. Obgleich viele dieser Daten bereits in frühen Studien mit suboptimaler Messmethodik/-technik und schlechter Reproduzierbarkeit evaluiert wurden, gelten sie in der wissenschaftlichen Literatur als absolut verlässlich und werden vielfach zitiert. Moderne bildgebende Verfahren wie die Kernspintomographie (MRT) oder die Computertomographie (CT) im Verbund mit valider Segmentierungstechnologie und quantitativer Analyse haben die in der Vergangenheit eingesetzten Verfahren mit suboptimaler Auflösung und ausschließlich qualitativer Auswerteprozedur bereits in vielen Bereichen ersetzt. So können Risikofaktoren wie die Körperfettverteilung derzeit schon quantifiziert und der Einfluss einer Intervention validiert werden (12-14). Auch die muskuläre Komponente der Körperzusammensetzung und insbesondere der Muskelquerschnitt wurde von einigen neueren Untersuchungen mittels moderner bildgebender Verfahren wie MRT oder CT untersucht (15, 16), die allerdings auf keine validierte Segmentierungs- und Quantifizierungssoftware zurückgreifen konnten.

Dies trifft ebenfalls für die kardiale Volumetrie mittels MRT zu, die derzeit als Goldstandard für die links- und rechtsventrikuläre Volumen- und Massenbestimmung gilt (17). Im Gegensatz zu echokardiographischen Untersuchungen handelt es sich bei der kardialen MRT um ein dreidimensionales Verfahren, welches dadurch eine wesentlich genauere morphologische Darstellung des Herzens ermöglicht (18) und somit u.a.

interventionsbedingte Veränderungen früher zu identifizieren vermag. Darüber hinaus werden sportliche Belastungen immer wieder mit einem plötzlichen Herztod, insbesondere bei männlichen Athleten, assoziiert (23-24). Ob hierfür pathologische morphologische Veränderungen des Myokards durch körperliches Training oder angeborene Organerkrankungen zu Grunde liegen, ist unklar.

10. Angaben zur Nutzen-Risiko-Relation

a. Welcher Nutzen ist von den Ergebnissen der Studie zu erwarten

aa) für die Versuchsteilnehmer?

Steigerung der körperlichen Fitness und physischen Attraktivität durch das Trainingsprogramm. Zudem Verminderung des metabolischen und kardiovaskulären Gesundheitsrisikos und Verbesserung der funktionellen Kapazität. Ggf. Detektion relevanter kardiologischer Befunde (Herzklappendefekte, Kardiomyopathie, Fehlbildungen).

ab) für die Heilkunde?

Beschreibung physiologischer muskulärer, metabolischer, physiologischer und kardialer Adaptation nach intensivem Krafttraining u.a. als Voraussetzung zur Abgrenzung pathologischer Veränderungen und Identifizierung geeigneter Verfahren zur Kontrolle von Trainingseffekten. Sekundäres Ziel ist es, Risikopatienten zu identifizieren, die vor Aufnahme eines spezifischen Trainings ein ausführliches Untersuchungsprogramm durchführen sollten, um Sekundärschäden zur vermeiden.

ac) für die Wissenschaft (z.B. Ergebnisse, die nicht unmittelbar therapeutischen Zwecken dienen)?

Verbesserter Einblick in die Grundlagen trainingsinduzierter muskulärer und kardiovaskulärer Adaptation als Basis optimaler sportwissenschaftlicher Trainings-empfehlungen. Evaluierung und Weiterentwicklung von bildgebenden Untersuchungs-verfahren und computerunterstützter Evaluationssoftware zur Diagnostik und zum Monitoring von Interventionseffekten.

b. Mit welchem Risiko ist die Studie für die Versuchsteilnehmer verbunden?

ba) Welcher Art sind die Risiken? Risikoeinschätzung, vorhersehbare Risiken der Behandlung und sonstiger studienbedingter Verfahren, die eingesetzt werden sollen (einschließlich Schmerz, Unannehmlichkeiten, Beschwerden, Verletzung der persönlichen Integrität und Maßnahmen zur Vermeidung und/oder zur Behandlung von unvorhersehbaren/ unerwünschten Ereignissen)

Die Risiken der Intervention (konsequent angeleitetes Krafttrainingsprogramm) sind sehr gering und bleiben nicht zuletzt aufgrund der intensiven Betreuung deutlich hinten denen eines selbständig durchgeführten Muskeltrainings zurück. Selbstverständlich sind in den ersten Wochen der Intervention leichte trainingsbedingte Beschwerden wie bspw. DOMS (Muskelkater) zu erwarten.

Die radiologischen Verfahren Dual Energy X-Ray Absorptiometrie (Gesamtkörper-DXA) bzw. Computertomographie (medialer Anteil Oberschenkel) sind mit niedrigen Strahlendosen (< 10 µSv pro DXA-Messung) bzw. geringen relativen Dosen (<0.8 mSv/CT-Messung[6]) verbunden. Eine Genehmigung dieser Verfahren

[6] Dieser Wert wurde ohne Berücksichtigung entsprechender Schutzmaßnahmen (Abdecken der Gonaden mit Bleischutz errechnet.

wird beim Bundesamt für Strahlenschutz nach Vorliegen der Genehmigung der Ethikkommission selbstverständlich beantragt.

Bei der Blutentnahme und durch periphere Verweilkatheter für die Kontrastmittel-(KM)-Applikation sind Blutergüsse und Infektionen nie komplett ausgeschlossen. In der Kernspintomographie werden nur geringe KM-Mengen eingesetzt. Daher sind die Risiken bei einem Paravasat des KMs in das Weichteilgewebe sowie Nierenbelastungen und mögliche allergische KM-Reaktionen minimiert. Probanden mit relativen oder absoluten Kontraindikationen für eine MRT werden von der Studie ausgeschlossen (s.u.). Insbesondere Probanden mit Nierenfunktionsstörungen oder bekannten KM-Reaktionen. Die KM-Gabe ist notwendig, um relevante kardiale Vorerkrankungen auszuschließen (siehe Ausschlusskriterien), die ein Risiko für die Intervention darstellen können.

bb) Mit welcher Wahrscheinlichkeit ist zu erwarten, daß sich die Risiken realisieren? Wie sicher ist die Wahrscheinlichkeit abschätzbar?

Insgesamt besteht nur eine geringe Wahrscheinlichkeit, dass sich die Risiken realisieren. Die Messungen stellen Standardmessungen der klinischen Routine dar, die von geschultem Fachpersonal sachkundig ausgeführt werden. Die Belastungsvorgaben erfolgen individuell, basierend auf einer Leistungsdiagnostik, sodass eine Überforderung der Teilnehmer kaum zu erwarten ist.

c. Warum ist das mögliche Risiko im Verhältnis zu dem zu erwartenden Nutzen Ihrer Ansicht nach vertretbar?

Es besteht keine wesentlich über das Alltagsrisiko hinausgehende Gefährdung, jedoch ein hoher zu erwartender Nutzen für die Teilnehmer hinsichtlich der Steigerung der körperlichen Fitness, gesundheitsrelevanter Größen, Wohlbefinden, Attraktivität und Selbstwirksamkeit/Kontrollüberzeugung.

d. Werden Zwischenergebnisse ausgewertet, um einen Trend zu erkennen?
ja ☒ nein, kurzer Interventionszeitraum macht Zwischenanalyse inadäquat.

e. Sind Kriterien festgelegt worden, bei deren Eintreten der Versuch geändert oder abgebrochen werden soll? ja, welche? ☒ nein

(allerdings Abbruch des jeweiligen Testverfahrens bei Unwohlsein, oder generell auf Wunsch des Patienten)

11. Bei klinischen Prüfungen nach MPG:

a. Welches Medizinprodukt soll geprüft werden? **entfällt**

b. Wird die klinische Prüfung von einer entsprechend qualifizierten und befugten Person geleitet, die mindestens eine zweijährige Erfahrung in der klinischen Prüfung von Medizinprodukten nachweisen kann? ja nein

c. Wurde (soweit erforderlich) eine dem jeweiligen Stand der wissenschaftlichen Erkenntnisse entsprechende biologische Sicherheitsprüfung oder sonstige für die vorgesehene Zweckbestimmung des Medizinproduktes erforderliche Prüfung durchgeführt? ja nein

d. Wurde (soweit erforderlich) die sicherheitstechnische Unbedenklichkeit für die Anwendung des Medizinproduktes unter Berücksichtigung des Standes der Technik sowie der Arbeitsschutz- und Unfallverhütungsvorschriften nachgewiesen?
ja nein

e. Ist der Leiter der klinischen Prüfung über die Ergebnisse der biologischen Sicherheitsprüfung und die voraussichtlich mit der klinischen Prüfung verbundenen Risiken informiert worden? ja nein

12. a) Ist die Mitarbeit eines Statistikers vorgesehen? ☒ ja nein
b) Welche statistischen Methoden sollen benutzt werden?

Komplettes statistisches Instrumentarium zur Erfassung von Effekten (bspw. Varianzanalysen/nicht parametrische Tests zur Identifikation von Zwischengruppenunterschieden). Zudem regressionsanalytische Modelle zur Aufklärung von Varianzen.

13. a) Handelt es sich um eine multizentrische Studie (d.h. eine nach einem *einzigen* Prüfplan durchgeführte Studie, die in mehr als einer Prüfstelle erfolgt und daher von mehr als einem Prüfer vorgenommen wird)? ja ☒ nein

b) Wurden/Werden an anderer Stelle Studien mit demselben oder einem ähnlichen Ziel durchgeführt? ja, wo? ☒ nein

Es wurden in der Vergangenheit bereits mehrere Untersuchungen mit dem Ziel der Evaluierung eines gesundheitssportlichen Trainings auf muskuläre, physiologische, metabolische und kardiale Parameter (meist isoliert) durchgeführt (s.o.). Diese Studie ist jedoch die erste, die u.a. den Effekt eines intensiven Kraft-/Powertrainings auf muskuläre Parameter, Körperzusammensetzung, metabolische und kardiovaskuläre Größen bei untrainierten Männern in mittlerem Lebensalter auch mittels moderner bildgebender Verfahren und Auswertesoftware evaluiert.

14. Wer hat die Studie initiiert? **Institut für Medizinische Physik**

15. Wer finanziert sie? (*Bitte geben Sie an, ob Drittmittel von nichtöffentlicher Seite beantragt werden. Falls ja, in welcher Höhe?*)

Derzeit sind noch keine Mittel beantragt, es werden jedoch nach positivem Votum der Ethikkommission versucht über unterschiedliche Ebenen Drittmittel zu generieren.

16. Die Aufwandsentschädigung wird übernommen von (*bitte Ansprechpartner benennen*):

Institut für Medizinische Physik (Direktor: Prof. Dr. Dr. W.A. Kalender)

Ansprechpartner: Prof. Dr. Wolfgang Kemmler

III. Angaben zu den Versuchsteilnehmern

1. Anzahl (*bei vergleichenden Studien bitte Aufteilung auf Gruppen angeben*)

80 Personen gesamt:

- Gruppe 1, n = 40: High Intensity Resistance Training (HIT)[7]
- Gruppe 2, n = 40: Kontrollgruppe (Wartegruppe; im Anschluss „Power-Training")

Bei Nullhypothesen-basierten Studien:
Wurde eine formale Fallzahlschätzung vorgenommen?

☒ ja, Basis CSA-Oberschenkelmuskulatur nein

2. Alter und Geschlecht (*bitte geben Sie das Alter der Versuchsteilnehmer sowie die als Ausschlusskriterien vorgesehenen Ober- und Untergrenzen an*)

[7] Training mit relativ hoher Reizintensität (≥70% des Einwiederholungsmaximums: 1RM)

Männer, 30. - 50. Lebensjahr

3. Status: Handelt es sich bei den Versuchsteilnehmern um

   ☒ gesunde Personen

   schwangere oder stillende Frauen

   Kinder oder Jugendliche

   einschlägig Erkrankte (*bitte geben Sie die Krankheit und das Stadium an*)

   Personen, die an anderen Krankheiten leiden? (Insbesondere: psychische Krankheiten, die Zweifel an der Geschäfts- oder Einsichtsfähigkeit begründen)

4. Welche sonstigen **Einschlusskriterien** (z.B. erlaubte Begleitmedikation) sind vorgesehen?

   - initial Untrainierte (während der vergangenen 2 Jahre: ≤1 h/Woche Sport mit positivem Effekt auf die Muskulatur; ≤2 h/Woche Sport gesamt)

5. Welche sonstigen **Ausschlusskriterien** (z.B. fortgeschrittene Nieren- oder Leber-insuffizienz, verbotene Begleitmedikation etc.) sind vorgesehen?

   - Geschichte leistungssportlicher Ausübung von Disziplinen mit erheblicher Relevanz für Körperzusammensetzung und Kraftfähigkeiten
   - pathologische muskuläre, metabolische und kardiale Veränderungen oder Entzündungen; deutlich eingeschränkte Gelenksbeweglichkeit in Knie und Hüfte.
   - Medikamente/Erkrankungen mit relevantem Einfluss auf Körperzusammensetzung und Herz-Kreislauf-System
   - sehr geringe körperliche Leistungsfähigkeit (< 100 Watt auf dem Fahrradergometer)
   - schwere Adipositas (BMI > 35 kg/m^2)
   - Abwesenheit ≥ 2 Wochen während des Interventionszeitraums
   - Geplante Aufnahme einer relevanten parallelen Trainingsmaßnahme
   - Kontraindikationen gegen MRT (Klaustrophobie, Herzschrittmacher, magnetisier-bare intracorporale Fremdkörper); Körpermaße die eine MRT-Messung verhindern
   - Drogenmissbrauch

6. Sollen auch Personen teilnehmen, die auf gerichtliche oder behördliche Anordnung in einer Anstalt verwahrt werden?

   ja ☒ nein

7. Sollen auch Personen teilnehmen, die sich schon für andere Forschungsvorhaben zur Verfügung gestellt haben?

   ja ☒ nein

   wie lange muss die letzte Teilnahme zurückliegen?

8. Bei Studien an Minderjährigen (oder sonst nicht geschäftsfähigen Personen) **entfällt**

   a. Warum kann die Studie nicht an Erwachsenen (voll Geschäftsfähigen) durchgeführt werden?

   b. Sind Aufklärung und Einwilligung der (des) gesetzlichen Vertreter(s) gewährleistet? (*bitte vorformulierte Erklärung beifügen*)

   ja nein, weil

c. Sind zusätzliche Aufklärung und Einwilligung der minderjährigen (nicht voll geschäftsfähigen) Versuchsteilnehmer gewährleistet, die selbst in der Lage sind, Wesen, Bedeutung und Tragweite des Versuchs einzusehen und ihren Willen danach zu bestimmen?

ja nein

9. Probandenversicherung

Wird zugunsten der Versuchsteilnehmer eine Versicherung abgeschlossen?

ja (*bitte Police beifügen, aus der die Versicherungsgesellschaft und die Höhe der Versicherungsleistung hervorgeht)*

☒ nein

10. Schweigepflicht/Datenschutz

Werden die ärztlichen Schweigepflicht- und die Datenschutzbestimmungen beachtet?

ja

11. Entgelt für Probanden

Soll den Versuchsteilnehmern ein Entgelt (Aufwandsentschädigung o.ä.) gezahlt werden?

ja, in Höhe von EUR ☒ nein

12. Wie sollen die Versuchsteilnehmer über Wesen, Bedeutung und Tragweite der Studie **aufgeklärt** werden?

Bitte in deutscher Sprache beifügen:

Dokumentation des Inhalts der Patientenaufklärung durch die/den versuchsdurchführende/n Ärztin/Arzt (Merkblatt), insbesondere mit Hinweisen über:

- **Ziele und Methoden** der Studie;
- **Nutzen und Risiko** der Studie;
- bekannte und möglicherweise zu erwartende **Wirkungen und Nebenwirkungen** von Medikamenten;
- Eingriffe, die nur aus wissenschaftlichen Gründen erfolgen;
- ein angebrachtes Verhalten des Patienten während und nach dem Versuch;
- die **Widerruflichkeit** einer Einwilligung;
- das Bestehen und den Umfang der gesetzlichen **Probandenversicherung** (Name/Anschrift/Telefon/Fax der Versicherungsgesellschaft, Nummer der Versicherungspolice) sowie die danach von der Versuchsperson zu beachtenden Obliegenheiten;
- **Ausschlusskriterien** (z.B. Schwangerschaft/Stillzeit);
- Name und Telefon des **Ansprechpartner**s vor Ort.
- **Besondere Aufklärung** über die Situation
  a. bei der randomisierten Studie
  f. beim Blind- und Doppelblindversuch.

13. Wie sollen die Versuchsteilnehmer ihre **Einwilligung** in die Teilnahme an der Studie erklären? (*bitte formulierte deutschsprachige Erklärung mit datenschutzrechtlicher Einwilligungserklärung beifügen*)

Schriftliche Einverständniserklärung nach umfangreicher mündlicher und schriftlicher Aufklärung über Ziele, Nutzen und Risiken der Untersuchung.

Ich weiß, daß auch bei einer positiven Beurteilung des Vorhabens durch die Ethik-Kommission der Medizinischen Fakultät der FAU Erlangen-Nürnberg die ärztliche und juristische Verantwortung für die Durchführung des Projektes uneingeschränkt bei der Leiterin/dem Leiter verbleibt.

Erlangen/Nürnberg
Datum ....

Unterschrift des/der Antragstellers/in

____

(Name in Druckbuchstaben)

Unterschrift der/des Leiterin/Leiters der Einrichtung, in der das Vorhaben durchgeführt werden soll.
Mit der Durchführung des Forschungsvorhabens einverstanden:

Datum ....

Unterschrift des/der Leiters/Leiterin der Einrichtung

____

(Name in Druckbuchstaben)

EK_May09/mit Unterschriftsblatt

Literatur

1. Benson AC, Torode ME, Fiatarone Singh MA. Effects of resistance training on metabolic fitness in children and adolescents: a systematic review. *Obes Rev*. 2008;9(1):43-66.
2. Kelley GA, Kelley KS. Impact of progressive resistance training on lipids and lipoproteins in adults: a meta-analysis of randomized controlled trials. *Prev Med*. 2009;48(1):9-19.
3. Latham NK, Bennett DA, Stretton CM, Anderson CS. Systematic review of progressive resistance strength training in older adults. *J Gerontol A Biol Sci Med Sci*. 2004;59(1):48-61.
4. Macaluso A, De Vito G. Muscle strength, power and adaptations to resistance training in older people. *Eur J Appl Physiol*. 2004;91:450-472.
5. Snowling NJ, Hopkins WG. Effects of different modes of exercise training on glucose control and risk factors for complications in type 2 diabetic patients: a meta-analysis. *Diabetes Care*. 2006;29(11):2518-27.
6. Weineck J. *Optimales Training* Erlangen: Spitta-Verlag; 2007.
7. Weineck J. *Sportbiologie*. Vol. 10 Balingen: Spitta Verlag; 2009.
8. Wijndaele K, Beunen G, Duvigneaud N, et al. A continuous metabolic syndrome risk score: utility for epidemiological analyses. *Diabetes Care*. 2006;29(10):2329.
9. Wilson PW, D'Agostino RB, Levy D, Belanger AM, Silbershatz H, Kannel WB. Prediction of coronary heart disease using risk factor categories. *Circulation*. 1998;97(18):1837-47.
10. Asikainen TM, Kukkonen-Harjula K, Miilunpalo S. Exercise for health for early postmenopausal women: a systematic review of randomised controlled trials. *Sports Med*. 2004;34(11):753-78.
11. Lagally KM, Cordero J, Good J, Brown DD, McCaw ST. Physiologic and metabolic responses to a continuous functional resistance exercise workout. *J Strength Cond Res*. 2009;23(2):373-9.
12. Kay SJ, Fiatarone Singh MA. The influence of physical activity on abdominal fat: a systematic review of the literature. *Obes Rev*. 2006;7(2):183-200.
13. Kemmler W, von Stengel S, Engelke K, Haberle L, Mayhew JL, Kalender WA. Exercise, body composition, and functional ability: a randomized controlled trial. *Am J Prev Med*. 2010;38(3):279-87.
14. Lamb HJ. Total body fat distribution as part of multiorgan MR imaging: new tool for risk assessment in the metabolic syndrome? *Radiology*. 2010;257(2):307-8.
15. Weiss EP, Racette SB, Villareal DT, et al. Lower extremity muscle size and strength and aerobic capacity decrease with caloric restriction but not with exercise-induced weight loss. *J Appl Physiol*. 2007;102(2):634-40.
16. Valtonen A, Poyhonen T, Sipila S, Heinonen A. Effects of aquatic resistance training on mobility limitation and lower-limb impairments after knee replacement. *Arch Phys Med Rehabil*;91(6):833-9.
17. Petersen SE, Hudsmith LE, Robson MD, et al. Sex-specific characteristics of cardiac function, geometry, and mass in young adult elite athletes. *J Magn Reson Imaging*. 2006;24(2):297-303.
18. Grothues F, Smith GC, Moon JC, et al. Comparison of interstudy reproducibility of cardiovascular magnetic resonance with two-dimensional echocardiography in normal subjects and in patients with heart failure or left ventricular hypertrophy. *Am J Cardiol*. 2002;90(1):29-34.

An die Geschäftsstelle der
Ethik-Kommission
der Medizinischen Fakultät
der FAU Erlangen-Nürnberg
Krankenhausstr. 12
91054 Erlangen

Antrag
an die Ethik-Kommission
der Medizinischen Fakultät

*Bitte **in deutscher Sprache** ausfüllen,*
Zutreffendes bitte ankreuzen.
Für multizentrische Studien mit Vorvotum einer nach Landesrecht gebildeten zuständigen Ethik-Kommission können Sie das verkürzte Antragsformular verwenden, abzurufen unter: http://www.ethik.med.uni-erlangen.de *(Anschlussvotum)*

Antrag auf Beurteilung eines Forschungsprojektes
(keine Arzneimittelprüfung)

bitte 9-fach einschließlich Anlagen einreichen sowie 1-mal in elektronischer Fassung

Titel des Projektes:
Einfluss eines 16-wöchigen Kraft-Trainingsprogramms auf leistungsphysiologische und gesundheitsrelevante muskuläre und kardiale Größen bei untrainierten Männern im mittleren Lebensalter. Eine randomisierte kontrollierte Interventionsstudie mit modernen, bildgebenden Verfahren.

I. Projektleitung

1. Name der/des verantwortlichen Projektleiterin/s an der FAU:
Professor Dr. Wolfgang Kemmler[1], Osteoporose-Forschungszentrum, Institut für Medizinische Physik, FAU (Direktor: Professor Dr. Dr. Willi A. Kalender)
App.-Nr. 23999; E-Mail: wolfgang.kemmler@imp.uni-erlangen.de

Angaben über die Qualifikation der/des Versuchsleiterin/s:
Promotion, Habilitation (liegen bereits vor)

2. a) Weitere Teilnehmer/innen vor Ort (alphabetische Reihenfolge):
Prof. Dr. Klaus Engelke, IMP, FAU
PD. Dr. Michael Lell, Radiologisches Institut, FAU
Prof. Dr. Harald Quick, IMP, FAU
PD. Dr. Axel Schmid, Radiologisches Institut, FAU
Dr. Michael Scharf, Radiologisches Institut, FAU
Dr. Simon von Stengel, Osteoporoseforschungszentrum, FAU
Andreas Wittke, IMP, FAU

b) Weitere Prüfzentren (bei multizentrischen Studien): keine

3. Handelt es sich bei diesem Antrag um ein bereits von der Ethik-Kommission der Medizinischen Fakultät der Friedrich-Alexander-Universität Erlangen-Nürnberg begutachtetes Projekt?

ja (bitte lfd. Nr. angeben) ☒ nein

II. Forschungsvorhaben

1. Geplanter Beginn der Studie: September 2012 // voraussichtliches Ende: August 2013
Dauer der Studienteilnahme für den einzelnen Probanden:

Intervention (s.u.): 4 Monate // 8 Monate (Kontrollgruppen-Wartelisten-Design)
Untersuchung (s.u.): ca. 100 min jeweils zu Beginn und Studienende

2. Kurzer Abriss des Projektes *(maximal 1,5 Seiten)*:

Einführung und Fragestellung

Krafttraining gilt auch bei intensiver Durchführung als gesundheitsfördernde Intervention, die sich bei einem zunehmenden Anteil der Bevölkerung immer größerer Beliebtheit erfreut. Neben Körperformung und Attraktivität steht für viele Menschen der präventive Aspekt des Muskeltrainings im Vordergrund. Tatsächlich weisen eine Vielzahl von Untersuchungen (1-5) positive Effekte eines regelmäßig durchgeführten, „überschwelligen" Trainings nach, so auf gesundheitsrelevante muskuläre, physiologische, metabolische und kardiovaskuläre Parameter wie beispielsweise die Körperzusammensetzung/funktionelle Kapazität, Blutfette/Lipoproteine oder Glucoseintoleranz/ Insulinsensitivität. Zudem ist anzunehmen, dass auch relativ rasche funktionelle und morphologische Anpassungserscheinungen des Herzens nachweisbar sind (6, 7). Allerdings liegen keine kernspintomographischen Längsschnittuntersuchungen vor, welche die physiologischen Adaptationserscheinungen des Herzens nach einem mehrmonatigen intensiven Krafttraining bei inaktiven Personen evaluieren. Parallel dazu sind die vorliegenden Methoden, mit denen die Körperzusammensetzung und die muskuläre Massenentwicklung bislang evaluiert wurden, als suboptimal einzuschätzen. Um eine valide und reliable Erfassung der Veränderung muskulärer, physiologischer und kardiologischer Größen als Reaktion auf ein gesundheitsorientiertes Muskeltraining zu gewährleisten, ist der messmethodische Schwerpunkt der vorliegenden Untersuchung auf den Einsatz moderner bildgebender Verfahren (Kernspintomographie (MRT), Computertomographie (CT), Dual Energy X-Ray Absorptiometry (DXA)) und moderner Segmentierungs- und Quantifizierungssoftware gerichtet.
Ziel der Untersuchung ist somit die Evaluierung der Effekte eines intensiven 16-wöchigen Krafttrainingsprogramms auf leistungsphysiologische und gesundheitsrelevante muskuläre und kardiale Größen bei untrainierten Männern im mittleren Lebensalter unter besonderer Berücksichtigung bildgebender Verfahren.

Design:

Randomisierte, kontrollierte, teilverblindete Studie mit Wartegruppe (cross-over)

Endpunkte

Primäre Endpunkte:

- Muskelquerschnitt der Oberschenkelmuskulatur (MRT, CT)
- Intraabdominale Fettmasse (MRT)

Sekundäre Endpunkte:

- Kraftfähigkeiten (u.a. abhängige Variable) [1]
- Gesamtkörperfett und Muskelmasse sowie regionale Verteilung (DXA, MRT)
- Metabolisches Syndrom-Score (8), und 10 Jahres-CHD-Risiko (9)
- Kardiale Masse und enddiastolisches Volumen (MRT)
- Hormonelle Regulation (u.a. freies Testosteron, hGH, Cortisol)
- Schmerzintensität, Quality of Life

[1] Ein Ziel der Studie ist die Identifikation von Variablen, welche die Varianz des Kraft-/Leistungszuwachs am höchsten aufklären.

Stichprobe

Zwei-Gruppen Design, randomisiert, z.T. cross-over (mit Wartegruppe)[2]

- Gruppe 1, n = 40: High Intensity Resistance Training (HIT)[3]
- Gruppe 2, n = 40: Kontrollgruppe (Wartegruppe; im Anschluss „Power-Training[4]“)

Interventionsprogramm

Gruppe 1: HIT mit konventioneller Bewegungsgeschwindigkeit (TUT[5] 2s-1s-2s)

Periodisiertes, progressives Krafttraining über 16 Wochen, basierend auf individuellen Trainingsplänen auf der Basis von 1 RM/x RM-Tests (1, 4, 10 Wochen), zunehmende Intensivierung der Reizhöhe (bis Woche 8), anschließend periodisiertes HIT (70-92,5%, 1RM) mit 2-3 Trainingseinheiten je Woche (1-2 gemeinsame, überwachte Trainingseinheiten; 1-2 Trainingseinheiten in Eigenregie (Vorgaben über Trainingsplan)).

Gruppe 2 (ehemalige Kontrollgruppe): Power-Training (TUT: ⚡-1s-2s)

s.o. aber explosive Ausführung im konzentrischen Bewegungsbereich nach initialer Konditionierung über 6 Wochen. Vergleichbares Trainingsprotokoll, allerdings im Intensitätsbereich von 40-70%, 1RM.

3. Studienbezogene Maßnahmen:

Bitte beschreiben Sie hier alle Maßnahmen, die studienbedingt durchgeführt werden sowie alle erforderlichen Abweichungen von der üblichen Routine-Behandlung:

Interventionsprogramm s.o.

Messungen (jeweils basal und nach 16 Wochen)

Bildgebende Verfahren:

Ganzkörper-MRT und Ganzkörper-DXA zur Erfassung der gesamten und regionalen Körperzusammensetzung.

Lokale MRT und CT am Oberschenkel (über „Muskelbauch“)

Kardiale MRT-Untersuchung mit Kontrastmittel i.v. (Analyse fibrotischer Herzmuskelveränderungen, Strain der Herzmuskulatur, enddiastolisches Volumen (EDV), endsystolisches Volumen (ESV), Schlagvolumen (SV), Ejektionsfraktion (EF) und myokardiale Masse (MM), Herzmuskeldicke).

Blutdruck und Herzfrequenzverhalten in Ruhe

Körperliche Fitness:

Erfassung unterschiedlicher Kraftfähigkeiten (u.a. 1 RM, Schnellkraft) mittels isokinetischem Dynamometer.

Psychosoziale Parameter

Befindlichkeit und Schmerzparameter, QoL (Fragebogen)

Labor

Blutfette/Lipoproteine, Glukose, Insulin, HbAC1, Entzündungsmarker

Testosteron, freies Testosteron, hGH, Cortisol; (evt. noch Parameter des Immunsystems)

Ernährungsanalyse

Analyse über 4 Tage (standardisierte Protokolle)

Anamnese und Risikofaktorenprofil über Fragebogen

---

[2] Die Wartegruppe dient zunächst als parallele Kontrollgruppe zur Interventionsgruppe. Nach Abschluss dieses Untersuchungsabschnittes wird mit dieser Gruppe ebenfalls eine 16-wöchige Intervention durchgeführt, sodass ein eingeschränktes 3-Gruppendesign generiert wird.

[3] Training mit relativ hoher Reizintensität (≥70% des Einwiederholungsmaximums: 1RM)

[4] Training mit explosiver Bewegungsausführung im konzentrischen Bereich (bei vglw. geringer Reizintensität im Bereich 40-60% 1RM)

[5] **T**ime **U**nder **T**ension: Dauer der jeweiligen Bewegungsabschnitte, konzentrisch – isometrisch – exzentrisch in Sec.

4. Wird die Studie gemäß der von der 48. Generalversammlung des Weltärztebundes in Somerset West revidierten Deklaration von Helsinki aus dem Jahre 1996 durchgeführt? Bitte angeben, ob alle anderen Erprobungsmöglichkeiten ausgeschöpft wurden.

Ja, die Intervention wurde in vorhergehenden Studien bereits validiert und optimiert. Die Messtechnologie ist ebenfalls etabliert, wurde aber bislang nur suboptimal zur Validierung der hier genannten Fragestellung eingesetzt.

5. Art des Forschungsvorhabens:
Handelt es sich um
eine diagnostische Prüfung?
eine therapeutische Prüfung?
eine Verträglichkeitsprüfung?
☒ einen ausschließlich wissenschaftlichen Versuch?

6. Gesetzliche Grundlagen

a) Handelt es sich um eine Untersuchung, die dazu bestimmt ist, klinische oder pharmakologische Wirkungen von Arzneimitteln zu erforschen oder nachzuweisen oder Nebenwirkungen festzustellen oder die Resorption, die Verteilung, den Stoffwechsel oder die Ausscheidung zu untersuchen, **mit dem Ziel, sich von der Unbedenklichkeit oder Wirksamkeit des Arzneimittels zu überzeugen** (klinische Prüfung eines Arzneimittels nach §§ 40 Arzneimittelgesetz)?

nein

Bitte begründen. Erläuterungen zum Antrag auf Bewertung einer klinischen Arzneimittelprüfung nach § 40 AMG finden Sie unter http://www.ethik.med.uni-erlangen.de/

b) Handelt es sich um eine klinische Prüfung nach § 20 Medizinproduktegesetz (MPG)?
ja ☒ nein
Bitte begründen. Liegt eine CE-Zertifizierung für das Medizinprodukt vor? Werden zusätzlich invasive oder andere belastende Untersuchungen durchgeführt?
Sämtliche Messverfahren sind CE-zertifiziert und entsprechend MPG geprüft und zugelassen. Invasive Untersuchungen werden nicht durchgeführt, mit Ausnahme von Blutentnahmen und einer venösen Kontrastmittelapplikation.

c) Handelt es sich um ein Vorhaben nach § 8 des Gesetzes zur Regelung des Transfusionswesens (TFG)?
ja ☒ nein

7. Handelt es sich um einen Versuch nach

§ 23 Strahlenschutzverordnung? ☒ ja nein

§ 28 Röntgenverordnung? ☒ ja nein

8. Typ der Studie:

- offen
- ☒ blind
- doppelblind
- ☒ vergleichend
- ☒ randomisiert
- multizentrisch
- ☒ Feldstudie
- Pilotstudie

9. Wissenschaftliche Begründung des Projekts, insbesondere:

a. Erläuterung des Versuchsziels

Das Ziel der Untersuchung ist die Evaluierung eines intensiven körperlichen Krafttrainings auf leistungsphysiologische und gesundheitsrelevante muskuläre und kardiale Größen bei untrainierten Männern im mittleren Lebensalter unter besonderer Berücksichtigung moderner bildgebender Verfahren. Aus radiologischer Sicht ist das Ziele der Studie die Identifikation typischer Kenngrößen der physiologischen Adaption der Körperzusammensetzung und des Herz-Kreislauf-Systems nach unterschiedlichen Typen von Krafttraining (s.o.) sowie der Methodenvergleich kernspintomographischer mit computertomographischen Muskelquerschnitten des Oberschenkels sowie der gesamten und der regionalen Gesamtkörperzusammensetzung mittels MRT vs. dem Goldstandard DXA-Methode.

b. Darstellung des bisherigen Wissensstandes

Eine Vielzahl von wissenschaftlichen Untersuchungen berichten den positiven Effekt eines „Krafttrainings“ auf funktionelle und gesundheitsrelevante physiologische und metabolische Größen (Übersicht in (1, 3, 10, 11)) bei Menschen in mittlerem Lebensalter. Obgleich viele dieser Daten bereits in frühen Studien mit suboptimaler Messmethodik/-technik und schlechter Reproduzierbarkeit evaluiert wurden, gelten sie in der wissenschaftlichen Literatur als absolut verlässlich und werden vielfach zitiert. Moderne bildgebende Verfahren wie die Kernspintomographie (MRT) oder die Computertomographie (CT) im Verbund mit valider Segmentierungstechnologie und quantitativer Analyse haben die in der Vergangenheit eingesetzten Verfahren mit suboptimaler Auflösung und ausschließlich qualitativer Auswerteprozedur bereits in vielen Bereichen ersetzt. So können Risikofaktoren wie die Körperfettverteilung derzeit schon quantifiziert und der Einfluss einer Intervention validiert werden (12-14). Auch die muskuläre Komponente der Körperzusammensetzung und insbesondere der Muskelquerschnitt wurde von einigen neueren Untersuchungen mittels moderner bildgebender Verfahren wie MRT oder CT untersucht (15, 16), die allerdings auf keine validierte Segmentierungs- und Quantifizierungssoftware zurückgreifen konnten.

Dies trifft ebenfalls für die kardiale Volumetrie mittels MRT zu, die derzeit als Goldstandard für die links- und rechtsventrikuläre Volumen- und Massenbestimmung gilt (17). Im Gegensatz zu echokardiographischen Untersuchungen handelt es sich bei der kardialen MRT um ein dreidimensionales Verfahren, welches dadurch eine wesentlich genauere morphologische Darstellung des Herzens ermöglicht (18) und somit u.a.

interventionsbedingte Veränderungen früher zu identifizieren vermag. Darüber hinaus werden sportliche Belastungen immer wieder mit einem plötzlichen Herztod, insbesondere bei männlichen Athleten, assoziiert (23-24). Ob hierfür pathologische morphologische Veränderungen des Myokards durch körperliches Training oder angeborene Organerkrankungen zu Grunde liegen, ist unklar.

10. Angaben zur Nutzen-Risiko-Relation

a. Welcher Nutzen ist von den Ergebnissen der Studie zu erwarten

aa) für die Versuchsteilnehmer?

Steigerung der körperlichen Fitness und physischen Attraktivität durch das Trainingsprogramm. Zudem Verminderung des metabolischen und kardiovaskulären Gesundheitsrisikos und Verbesserung der funktionellen Kapazität. Ggf. Detektion relevanter kardiologischer Befunde (Herzklappendefekte, Kardiomyopathie, Fehlbildungen).

ab) für die Heilkunde?

Beschreibung physiologischer muskulärer, metabolischer, physiologischer und kardialer Adaptation nach intensivem Krafttraining u.a. als Voraussetzung zur Abgrenzung pathologischer Veränderungen und Identifizierung geeigneter Verfahren zur Kontrolle von Trainingseffekten. Sekundäres Ziel ist es, Risikopatienten zu identifizieren, die vor Aufnahme eines spezifischen Trainings ein ausführliches Untersuchungsprogramm durchführen sollten, um Sekundärschäden zur vermeiden.

ac) für die Wissenschaft (z.B. Ergebnisse, die nicht unmittelbar therapeutischen Zwecken dienen)?

Verbesserter Einblick in die Grundlagen trainingsinduzierter muskulärer und kardiovaskulärer Adaptation als Basis optimaler sportwissenschaftlicher Trainingsempfehlungen. Evaluierung und Weiterentwicklung von bildgebenden Untersuchungsverfahren und computerunterstützter Evaluationssoftware zur Diagnostik und zum Monitoring von Interventionseffekten.

b. Mit welchem Risiko ist die Studie für die Versuchsteilnehmer verbunden?

ba) Welcher Art sind die Risiken? Risikoeinschätzung, vorhersehbare Risiken der Behandlung und sonstiger studienbedingter Verfahren, die eingesetzt werden sollen (einschließlich Schmerz, Unannehmlichkeiten, Beschwerden, Verletzung der persönlichen Integrität und Maßnahmen zur Vermeidung und/oder zur Behandlung von unvorhersehbaren/ unerwünschten Ereignissen)

Die Risiken der Intervention (konsequent angeleitetes Krafttrainingsprogramm) sind sehr gering und bleiben nicht zuletzt aufgrund der intensiven Betreuung deutlich hinten denen eines selbständig durchgeführten Muskeltrainings zurück. Selbstverständlich sind in den ersten Wochen der Intervention leichte trainingsbedingte Beschwerden wie bspw. DOMS (Muskelkater) zu erwarten.

Die radiologischen Verfahren Dual Energy X-Ray Absorptiometrie (Gesamtkörper-DXA) bzw. Computertomographie (medialer Anteil Oberschenkel) sind mit niedrigen Strahlendosen (< 10 µSv pro DXA-Messung) bzw. geringen relativen Dosen (<0.8 mSv/CT-Messung[6]) verbunden. Eine Genehmigung dieser Verfahren

[6] Dieser Wert wurde ohne Berücksichtigung entsprechender Schutzmaßnahmen (Abdecken der Gonaden mit Bleischutz errechnet.

wird beim Bundesamt für Strahlenschutz nach Vorliegen der Genehmigung der Ethikkommission selbstverständlich beantragt.

Bei der Blutentnahme und durch periphere Verweilkatheter für die Kontrastmittel-(KM)-Applikation sind Blutergüsse und Infektionen nie komplett ausgeschlossen. In der Kernspintomographie werden nur geringe KM-Mengen eingesetzt. Daher sind die Risiken bei einem Paravasat des KMs in das Weichteilgewebe sowie Nierenbelastungen und mögliche allergische KM-Reaktionen minimiert. Probanden mit relativen oder absoluten Kontraindikationen für eine MRT werden von der Studie ausgeschlossen (s.u.). Insbesondere Probanden mit Nierenfunktionsstörungen oder bekannten KM-Reaktionen. Die KM-Gabe ist notwendig, um relevante kardiale Vorerkrankungen auszuschließen (siehe Ausschlusskriterien), die ein Risiko für die Intervention darstellen können.

bb) Mit welcher Wahrscheinlichkeit ist zu erwarten, daß sich die Risiken realisieren? Wie sicher ist die Wahrscheinlichkeit abschätzbar?

Insgesamt besteht nur eine geringe Wahrscheinlichkeit, dass sich die Risiken realisieren. Die Messungen stellen Standardmessungen der klinischen Routine dar, die von geschultem Fachpersonal sachkundig ausgeführt werden. Die Belastungsvorgaben erfolgen individuell, basierend auf einer Leistungsdiagnostik, sodass eine Überforderung der Teilnehmer kaum zu erwarten ist.

c. Warum ist das mögliche Risiko im Verhältnis zu dem zu erwartenden Nutzen Ihrer Ansicht nach vertretbar?

Es besteht keine wesentlich über das Alltagsrisiko hinausgehende Gefährdung, jedoch ein hoher zu erwartender Nutzen für die Teilnehmer hinsichtlich der Steigerung der körperlichen Fitness, gesundheitsrelevanter Größen, Wohlbefinden, Attraktivität und Selbstwirksamkeit/Kontrollüberzeugung.

d. Werden Zwischenergebnisse ausgewertet, um einen Trend zu erkennen?
ja ☒ nein, kurzer Interventionszeitraum macht Zwischenanalyse inadäquat.

e. Sind Kriterien festgelegt worden, bei deren Eintreten der Versuch geändert oder abgebrochen werden soll? ja, welche? ☒ nein

(allerdings Abbruch des jeweiligen Testverfahrens bei Unwohlsein, oder generell auf Wunsch des Patienten)

11. Bei klinischen Prüfungen nach MPG:

a. Welches Medizinprodukt soll geprüft werden? **entfällt**

b. Wird die klinische Prüfung von einer entsprechend qualifizierten und befugten Person geleitet, die mindestens eine zweijährige Erfahrung in der klinischen Prüfung von Medizinprodukten nachweisen kann? ja nein

c. Wurde (soweit erforderlich) eine dem jeweiligen Stand der wissenschaftlichen Erkenntnisse entsprechende biologische Sicherheitsprüfung oder sonstige für die vorgesehene Zweckbestimmung des Medizinproduktes erforderliche Prüfung durchgeführt? ja nein

d. Wurde (soweit erforderlich) die sicherheitstechnische Unbedenklichkeit für die Anwendung des Medizinproduktes unter Berücksichtigung des Standes der Technik sowie der Arbeitsschutz- und Unfallverhütungsvorschriften nachgewiesen?
ja nein

e. Ist der Leiter der klinischen Prüfung über die Ergebnisse der biologischen Sicherheitsprüfung und die voraussichtlich mit der klinischen Prüfung verbundenen Risiken informiert worden? ja nein

12. a) Ist die Mitarbeit eines Statistikers vorgesehen? ☒ ja nein
b) Welche statistischen Methoden sollen benutzt werden?

Komplettes statistisches Instrumentarium zur Erfassung von Effekten (bspw. Varianzanalysen/nicht parametrische Tests zur Identifikation von Zwischengruppenunterschieden). Zudem regressionsanalytische Modelle zur Aufklärung von Varianzen.

13. a) Handelt es sich um eine multizentrische Studie (d.h. eine nach einem *einzigen* Prüfplan durchgeführte Studie, die in mehr als einer Prüfstelle erfolgt und daher von mehr als einem Prüfer vorgenommen wird)? ja ☒ nein

b) Wurden/Werden an anderer Stelle Studien mit demselben oder einem ähnlichen Ziel durchgeführt? ja, wo? ☒ nein

Es wurden in der Vergangenheit bereits mehrere Untersuchungen mit dem Ziel der Evaluierung eines gesundheitssportlichen Trainings auf muskuläre, physiologische, metabolische und kardiale Parameter (meist isoliert) durchgeführt (s.o.). Diese Studie ist jedoch die erste, die u.a. den Effekt eines intensiven Kraft-/Powertrainings auf muskuläre Parameter, Körperzusammensetzung, metabolische und kardiovaskuläre Größen bei untrainierten Männern in mittlerem Lebensalter auch mittels moderner bildgebender Verfahren und Auswertesoftware evaluiert.

14. Wer hat die Studie initiiert? **Institut für Medizinische Physik**

15. Wer finanziert sie? (*Bitte geben Sie an, ob Drittmittel von nichtöffentlicher Seite beantragt werden. Falls ja, in welcher Höhe?*)

Derzeit sind noch keine Mittel beantragt, es werden jedoch nach positivem Votum der Ethikkommission versucht über unterschiedliche Ebenen Drittmittel zu generieren.

16. Die Aufwandsentschädigung wird übernommen von (*bitte Ansprechpartner benennen*):

Institut für Medizinische Physik (Direktor: Prof. Dr. Dr. W.A. Kalender)

Ansprechpartner: Prof. Dr. Wolfgang Kemmler

III. Angaben zu den Versuchsteilnehmern

1. Anzahl (*bei vergleichenden Studien bitte Aufteilung auf Gruppen angeben*)

80 Personen gesamt:

- Gruppe 1, n = 40: High Intensity Resistance Training (HIT)[7]
- Gruppe 2, n = 40: Kontrollgruppe (Wartegruppe; im Anschluss „Power-Training“)

Bei Nullhypothesen-basierten Studien:
Wurde eine formale Fallzahlschätzung vorgenommen?

☒ ja, Basis CSA-Oberschenkelmuskulatur nein

2. Alter und Geschlecht (*bitte geben Sie das Alter der Versuchsteilnehmer sowie die als Ausschlusskriterien vorgesehenen Ober- und Untergrenzen an*)

[7] Training mit relativ hoher Reizintensität (≥70% des Einwiederholungsmaximums: 1RM)

Männer, 30. - 50. Lebensjahr

3. Status: Handelt es sich bei den Versuchsteilnehmern um
   ☒ gesunde Personen
   schwangere oder stillende Frauen
   Kinder oder Jugendliche
   einschlägig Erkrankte (*bitte geben Sie die Krankheit und das Stadium an*)
   Personen, die an anderen Krankheiten leiden? (Insbesondere: psychische Krankheiten, die Zweifel an der Geschäfts- oder Einsichtsfähigkeit begründen)

4. Welche sonstigen **Einschlusskriterien** (z.B. erlaubte Begleitmedikation) sind vorgesehen?

- initial Untrainierte (während der vergangenen 2 Jahre: ≤1 h/Woche Sport mit positivem Effekt auf die Muskulatur; ≤2 h/Woche Sport gesamt)

5. Welche sonstigen **Ausschlusskriterien** (z.B. fortgeschrittene Nieren- oder Leber-insuffizienz, verbotene Begleitmedikation etc.) sind vorgesehen?

- Geschichte leistungssportlicher Ausübung von Disziplinen mit erheblicher Relevanz für Körperzusammensetzung und Kraftfähigkeiten
- pathologische muskuläre, metabolische und kardiale Veränderungen oder Entzündungen; deutlich eingeschränkte Gelenksbeweglichkeit in Knie und Hüfte.
- Medikamente/Erkrankungen mit relevantem Einfluss auf Körperzusammensetzung und Herz-Kreislauf-System
- sehr geringe körperliche Leistungsfähigkeit (< 100 Watt auf dem Fahrradergometer)
- schwere Adipositas (BMI > 35 kg/m^2)
- Abwesenheit ≥ 2 Wochen während des Interventionszeitraums
- Geplante Aufnahme einer relevanten parallelen Trainingsmaßnahme
- Kontraindikationen gegen MRT (Klaustrophobie, Herzschrittmacher, magnetisier-bare intracorporale Fremdkörper); Körpermaße die eine MRT-Messung verhindern
- Drogenmissbrauch

6. Sollen auch Personen teilnehmen, die auf gerichtliche oder behördliche Anordnung in einer Anstalt verwahrt werden?
   ja ☒ nein

7. Sollen auch Personen teilnehmen, die sich schon für andere Forschungsvorhaben zur Verfügung gestellt haben?
   ja ☒ nein
   wie lange muss die letzte Teilnahme zurückliegen?

8. Bei Studien an Minderjährigen (oder sonst nicht geschäftsfähigen Personen) **entfällt**
   a. Warum kann die Studie nicht an Erwachsenen (voll Geschäftsfähigen) durchgeführt werden?

   b. Sind Aufklärung und Einwilligung der (des) gesetzlichen Vertreter(s) gewährleistet?
   (*bitte vorformulierte Erklärung beifügen*)
   ja nein, weil

c. Sind zusätzliche Aufklärung und Einwilligung der minderjährigen (nicht voll geschäftsfähigen) Versuchsteilnehmer gewährleistet, die selbst in der Lage sind, Wesen, Bedeutung und Tragweite des Versuchs einzusehen und ihren Willen danach zu bestimmen?

ja nein

9. Probandenversicherung

Wird zugunsten der Versuchsteilnehmer eine Versicherung abgeschlossen?

ja (*bitte Police beifügen, aus der die Versicherungsgesellschaft und die Höhe der Versicherungsleistung hervorgeht)*

☒ nein

10. Schweigepflicht/Datenschutz

Werden die ärztlichen Schweigepflicht- und die Datenschutzbestimmungen beachtet?

ja

11. Entgelt für Probanden

Soll den Versuchsteilnehmern ein Entgelt (Aufwandsentschädigung o.ä.) gezahlt werden?

ja, in Höhe von EUR ☒ nein

12. Wie sollen die Versuchsteilnehmer über Wesen, Bedeutung und Tragweite der Studie **aufgeklärt** werden?

Bitte in deutscher Sprache beifügen:

Dokumentation des Inhalts der Patientenaufklärung durch die/den versuchsdurchführende/n Ärztin/Arzt (Merkblatt), insbesondere mit Hinweisen über:

- **Ziele und Methoden** der Studie;
- **Nutzen und Risiko** der Studie;
- bekannte und möglicherweise zu erwartende **Wirkungen und Nebenwirkungen** von Medikamenten;
- Eingriffe, die nur aus wissenschaftlichen Gründen erfolgen;
- ein angebrachtes Verhalten des Patienten während und nach dem Versuch;
- die **Widerruflichkeit** einer Einwilligung;
- das Bestehen und den Umfang der gesetzlichen **Probandenversicherung** (Name/Anschrift/Telefon/Fax der Versicherungsgesellschaft, Nummer der Versicherungspolice) sowie die danach von der Versuchsperson zu beachtenden Obliegenheiten;
- **Ausschlusskriterien** (z.B. Schwangerschaft/Stillzeit);
- Name und Telefon des **Ansprechpartner**s vor Ort.
- **Besondere Aufklärung** über die Situation
  a. bei der randomisierten Studie
  f. beim Blind- und Doppelblindversuch.

13. Wie sollen die Versuchsteilnehmer ihre **Einwilligung** in die Teilnahme an der Studie erklären? (*bitte formulierte deutschsprachige Erklärung mit datenschutzrechtlicher Einwilligungserklärung beifügen*)

Schriftliche Einverständniserklärung nach umfangreicher mündlicher und schriftlicher Aufklärung über Ziele, Nutzen und Risiken der Untersuchung.

Ich weiß, daß auch bei einer positiven Beurteilung des Vorhabens durch die Ethik-Kommission der Medizinischen Fakultät der FAU Erlangen-Nürnberg die ärztliche und juristische Verantwortung für die Durchführung des Projektes uneingeschränkt bei der Leiterin/dem Leiter verbleibt.

Erlangen/Nürnberg
Datum ....

Unterschrift des/der Antragstellers/in

____
(Name in Druckbuchstaben)

Unterschrift der/des Leiterin/Leiters der Einrichtung, in der das Vorhaben durchgeführt werden soll.
Mit der Durchführung des Forschungsvorhabens einverstanden:

Datum ....

Unterschrift des/der Leiters/Leiterin der Einrichtung

____
(Name in Druckbuchstaben)

EK_May09/mit Unterschriftsblatt

Literatur

1. Benson AC, Torode ME, Fiatarone Singh MA. Effects of resistance training on metabolic fitness in children and adolescents: a systematic review. *Obes Rev*. 2008;9(1):43-66.
2. Kelley GA, Kelley KS. Impact of progressive resistance training on lipids and lipoproteins in adults: a meta-analysis of randomized controlled trials. *Prev Med*. 2009;48(1):9-19.
3. Latham NK, Bennett DA, Stretton CM, Anderson CS. Systematic review of progressive resistance strength training in older adults. *J Gerontol A Biol Sci Med Sci*. 2004;59(1):48-61.
4. Macaluso A, De Vito G. Muscle strength, power and adaptations to resistance training in older people. *Eur J Appl Physiol*. 2004;91:450-472.
5. Snowling NJ, Hopkins WG. Effects of different modes of exercise training on glucose control and risk factors for complications in type 2 diabetic patients: a meta-analysis. *Diabetes Care*. 2006;29(11):2518-27.
6. Weineck J. *Optimales Training* Erlangen: Spitta-Verlag; 2007.
7. Weineck J. *Sportbiologie*. Vol. 10 Balingen: Spitta Verlag; 2009.
8. Wijndaele K, Beunen G, Duvigneaud N, et al. A continuous metabolic syndrome risk score: utility for epidemiological analyses. *Diabetes Care*. 2006;29(10):2329.
9. Wilson PW, D'Agostino RB, Levy D, Belanger AM, Silbershatz H, Kannel WB. Prediction of coronary heart disease using risk factor categories. *Circulation*. 1998;97(18):1837-47.
10. Asikainen TM, Kukkonen-Harjula K, Miilunpalo S. Exercise for health for early postmenopausal women: a systematic review of randomised controlled trials. *Sports Med*. 2004;34(11):753-78.
11. Lagally KM, Cordero J, Good J, Brown DD, McCaw ST. Physiologic and metabolic responses to a continuous functional resistance exercise workout. *J Strength Cond Res*. 2009;23(2):373-9.
12. Kay SJ, Fiatarone Singh MA. The influence of physical activity on abdominal fat: a systematic review of the literature. *Obes Rev*. 2006;7(2):183-200.
13. Kemmler W, von Stengel S, Engelke K, Haberle L, Mayhew JL, Kalender WA. Exercise, body composition, and functional ability: a randomized controlled trial. *Am J Prev Med*. 2010;38(3):279-87.
14. Lamb HJ. Total body fat distribution as part of multiorgan MR imaging: new tool for risk assessment in the metabolic syndrome? *Radiology*. 2010;257(2):307-8.
15. Weiss EP, Racette SB, Villareal DT, et al. Lower extremity muscle size and strength and aerobic capacity decrease with caloric restriction but not with exercise-induced weight loss. *J Appl Physiol*. 2007;102(2):634-40.
16. Valtonen A, Poyhonen T, Sipila S, Heinonen A. Effects of aquatic resistance training on mobility limitation and lower-limb impairments after knee replacement. *Arch Phys Med Rehabil*;91(6):833-9.
17. Petersen SE, Hudsmith LE, Robson MD, et al. Sex-specific characteristics of cardiac function, geometry, and mass in young adult elite athletes. *J Magn Reson Imaging*. 2006;24(2):297-303.
18. Grothues F, Smith GC, Moon JC, et al. Comparison of interstudy reproducibility of cardiovascular magnetic resonance with two-dimensional echocardiography in normal subjects and in patients with heart failure or left ventricular hypertrophy. *Am J Cardiol*. 2002;90(1):29-34.

An die Geschäftsstelle der
Ethik-Kommission
der Medizinischen Fakultät
der FAU Erlangen-Nürnberg
Krankenhausstr. 12
91054 Erlangen

Antrag
an die Ethik-Kommission
der Medizinischen Fakultät

*Bitte **in deutscher Sprache** ausfüllen,*
Zutreffendes bitte ankreuzen.
Für multizentrische Studien mit Vorvotum einer nach Landesrecht gebildeten zuständigen Ethik-Kommission können Sie das verkürzte Antragsformular verwenden, abzurufen unter: http://www.ethik.med.uni-erlangen.de *(Anschlussvotum)*

Antrag auf Beurteilung eines Forschungsprojektes (keine Arzneimittelprüfung)

bitte 9-fach einschließlich Anlagen einreichen sowie 1-mal in elektronischer Fassung

Titel des Projektes:
Einfluss eines 16-wöchigen Kraft-Trainingsprogramms auf leistungsphysiologische und gesundheitsrelevante muskuläre und kardiale Größen bei untrainierten Männern im mittleren Lebensalter. Eine randomisierte kontrollierte Interventionsstudie mit modernen, bildgebenden Verfahren.

I. Projektleitung

1. Name der/des verantwortlichen Projektleiterin/s an der FAU:
Professor Dr. Wolfgang Kemmler[1], Osteoporose-Forschungszentrum, Institut für Medizinische Physik, FAU (Direktor: Professor Dr. Dr. Willi A. Kalender)
App.-Nr. 23999; E-Mail: wolfgang.kemmler@imp.uni-erlangen.de

Angaben über die Qualifikation der/des Versuchsleiterin/s:
Promotion, Habilitation (liegen bereits vor)

2. a) Weitere Teilnehmer/innen vor Ort (alphabetische Reihenfolge):
Prof. Dr. Klaus Engelke, IMP, FAU
PD. Dr. Michael Lell, Radiologisches Institut, FAU
Prof. Dr. Harald Quick, IMP, FAU
PD. Dr. Axel Schmid, Radiologisches Institut, FAU
Dr. Michael Scharf, Radiologisches Institut, FAU
Dr. Simon von Stengel, Osteoporoseforschungszentrum, FAU
Andreas Wittke, IMP, FAU

b) Weitere Prüfzentren (bei multizentrischen Studien): keine

3. Handelt es sich bei diesem Antrag um ein bereits von der Ethik-Kommission der Medizinischen Fakultät der Friedrich-Alexander-Universität Erlangen-Nürnberg begutachtetes Projekt?

ja (bitte lfd. Nr. angeben) ☒ nein

II. Forschungsvorhaben

1. Geplanter Beginn der Studie: September 2012 // voraussichtliches Ende: August 2013
Dauer der Studienteilnahme für den einzelnen Probanden:

Intervention (s.u.): 4 Monate // 8 Monate (Kontrollgruppen-Wartelisten-Design)
Untersuchung (s.u.): ca. 100 min jeweils zu Beginn und Studienende

2. Kurzer Abriss des Projektes *(maximal 1,5 Seiten)*:

Einführung und Fragestellung

Krafttraining gilt auch bei intensiver Durchführung als gesundheitsfördernde Intervention, die sich bei einem zunehmenden Anteil der Bevölkerung immer größerer Beliebtheit erfreut. Neben Körperformung und Attraktivität steht für viele Menschen der präventive Aspekt des Muskeltrainings im Vordergrund. Tatsächlich weisen eine Vielzahl von Untersuchungen (1-5) positive Effekte eines regelmäßig durchgeführten, „überschwelligen" Trainings nach, so auf gesundheitsrelevante muskuläre, physiologische, metabolische und kardiovaskuläre Parameter wie beispielsweise die Körperzusammensetzung/funktionelle Kapazität, Blutfette/Lipoproteine oder Glucoseintoleranz/ Insulinsensitivität. Zudem ist anzunehmen, dass auch relativ rasche funktionelle und morphologische Anpassungserscheinungen des Herzens nachweisbar sind (6, 7). Allerdings liegen keine kernspintomographischen Längsschnittuntersuchungen vor, welche die physiologischen Adaptationserscheinungen des Herzens nach einem mehrmonatigen intensiven Krafttraining bei inaktiven Personen evaluieren. Parallel dazu sind die vorliegenden Methoden, mit denen die Körperzusammensetzung und die muskuläre Massenentwicklung bislang evaluiert wurden, als suboptimal einzuschätzen. Um eine valide und reliable Erfassung der Veränderung muskulärer, physiologischer und kardiologischer Größen als Reaktion auf ein gesundheitsorientiertes Muskeltraining zu gewährleisten, ist der messmethodische Schwerpunkt der vorliegenden Untersuchung auf den Einsatz moderner bildgebender Verfahren (Kernspintomographie (MRT), Computertomographie (CT), Dual Energy X-Ray Absorptiometry (DXA)) und moderner Segmentierungs- und Quantifizierungssoftware gerichtet.
Ziel der Untersuchung ist somit die Evaluierung der Effekte eines intensiven 16-wöchigen Krafttrainingsprogramms auf leistungsphysiologische und gesundheitsrelevante muskuläre und kardiale Größen bei untrainierten Männern im mittleren Lebensalter unter besonderer Berücksichtigung bildgebender Verfahren.

Design:

Randomisierte, kontrollierte, teilverblindete Studie mit Wartegruppe (cross-over)

Endpunkte

Primäre Endpunkte:

- Muskelquerschnitt der Oberschenkelmuskulatur (MRT, CT)
- Intraabdominale Fettmasse (MRT)

Sekundäre Endpunkte:

- Kraftfähigkeiten (u.a. abhängige Variable) [1]
- Gesamtkörperfett und Muskelmasse sowie regionale Verteilung (DXA, MRT)
- Metabolisches Syndrom-Score (8), und 10 Jahres-CHD-Risiko (9)
- Kardiale Masse und enddiastolisches Volumen (MRT)
- Hormonelle Regulation (u.a. freies Testosteron, hGH, Cortisol)
- Schmerzintensität, Quality of Life

---

[1] Ein Ziel der Studie ist die Identifikation von Variablen, welche die Varianz des Kraft-/Leistungszuwachs am höchsten aufklären.

Stichprobe

Zwei-Gruppen Design, randomisiert, z.T. cross-over (mit Wartegruppe)[2]

- Gruppe 1, n = 40: High Intensity Resistance Training (HIT)[3]
- Gruppe 2, n = 40: Kontrollgruppe (Wartegruppe; im Anschluss „Power-Training[4]“)

Interventionsprogramm

Gruppe 1: HIT mit konventioneller Bewegungsgeschwindigkeit (TUT[5] 2s-1s-2s)

Periodisiertes, progressives Krafttraining über 16 Wochen, basierend auf individuellen Trainingsplänen auf der Basis von 1 RM/x RM-Tests (1, 4, 10 Wochen), zunehmende Intensivierung der Reizhöhe (bis Woche 8), anschließend periodisiertes HIT (70-92,5%, 1RM) mit 2-3 Trainingseinheiten je Woche (1-2 gemeinsame, überwachte Trainingseinheiten; 1-2 Trainingseinheiten in Eigenregie (Vorgaben über Trainingsplan)).

Gruppe 2 (ehemalige Kontrollgruppe): Power-Training (TUT: ϟ-1s-2s)

s.o. aber explosive Ausführung im konzentrischen Bewegungsbereich nach initialer Konditionierung über 6 Wochen. Vergleichbares Trainingsprotokoll, allerdings im Intensitätsbereich von 40-70%, 1RM.

3. Studienbezogene Maßnahmen:

Bitte beschreiben Sie hier alle Maßnahmen, die studienbedingt durchgeführt werden sowie alle erforderlichen Abweichungen von der üblichen Routine-Behandlung:

Interventionsprogramm s.o.

Messungen (jeweils basal und nach 16 Wochen)

Bildgebende Verfahren:

Ganzkörper-MRT und Ganzkörper-DXA zur Erfassung der gesamten und regionalen Körperzusammensetzung.

Lokale MRT und CT am Oberschenkel (über „Muskelbauch“)

Kardiale MRT-Untersuchung mit Kontrastmittel i.v. (Analyse fibrotischer Herzmuskelveränderungen, Strain der Herzmuskulatur, enddiastolisches Volumen (EDV), endsystolisches Volumen (ESV), Schlagvolumen (SV), Ejektionsfraktion (EF) und myokardiale Masse (MM), Herzmuskeldicke).

Blutdruck und Herzfrequenzverhalten in Ruhe

Körperliche Fitness:

Erfassung unterschiedlicher Kraftfähigkeiten (u.a. 1 RM, Schnellkraft) mittels isokinetischem Dynamometer.

Psychosoziale Parameter

Befindlichkeit und Schmerzparameter, QoL (Fragebogen)

Labor

Blutfette/Lipoproteine, Glukose, Insulin, HbAC1, Entzündungsmarker

Testosteron, freies Testosteron, hGH, Cortisol; (evt. noch Parameter des Immunsystems)

Ernährungsanalyse

Analyse über 4 Tage (standardisierte Protokolle)

Anamnese und Risikofaktorenprofil über Fragebogen

---

[2] Die Wartegruppe dient zunächst als parallele Kontrollgruppe zur Interventionsgruppe. Nach Abschluss dieses Untersuchungsabschnittes wird mit dieser Gruppe ebenfalls eine 16-wöchige Intervention durchgeführt, sodass ein eingeschränktes 3-Gruppendesign generiert wird.

[3] Training mit relativ hoher Reizintensität (≥70% des Einwiederholungsmaximums: 1RM)

[4] Training mit explosiver Bewegungsausführung im konzentrischen Bereich (bei vglw. geringer Reizintensität im Bereich 40-60% 1RM)

[5] **T**ime **U**nder **T**ension: Dauer der jeweiligen Bewegungsabschnitte, konzentrisch – isometrisch – exzentrisch in Sec.

4. Wird die Studie gemäß der von der 48. Generalversammlung des Weltärztebundes in Somerset West revidierten Deklaration von Helsinki aus dem Jahre 1996 durchgeführt? Bitte angeben, ob alle anderen Erprobungsmöglichkeiten ausgeschöpft wurden.

Ja, die Intervention wurde in vorhergehenden Studien bereits validiert und optimiert. Die Messtechnologie ist ebenfalls etabliert, wurde aber bislang nur suboptimal zur Validierung der hier genannten Fragestellung eingesetzt.

5. Art des Forschungsvorhabens:
Handelt es sich um
eine diagnostische Prüfung?
eine therapeutische Prüfung?
eine Verträglichkeitsprüfung?
☒ einen ausschließlich wissenschaftlichen Versuch?

6. Gesetzliche Grundlagen

a) Handelt es sich um eine Untersuchung, die dazu bestimmt ist, klinische oder pharmakologische Wirkungen von Arzneimitteln zu erforschen oder nachzuweisen oder Nebenwirkungen festzustellen oder die Resorption, die Verteilung, den Stoffwechsel oder die Ausscheidung zu untersuchen, **mit dem Ziel, sich von der Unbedenklichkeit oder Wirksamkeit des Arzneimittels zu überzeugen** (klinische Prüfung eines Arzneimittels nach §§ 40 Arzneimittelgesetz)?

nein

Bitte begründen. Erläuterungen zum Antrag auf Bewertung einer klinischen Arzneimittelprüfung nach § 40 AMG finden Sie unter http://www.ethik.med.uni-erlangen.de/

b) Handelt es sich um eine klinische Prüfung nach § 20 Medizinproduktegesetz (MPG)?
ja ☒ nein
Bitte begründen. Liegt eine CE-Zertifizierung für das Medizinprodukt vor? Werden zusätzlich invasive oder andere belastende Untersuchungen durchgeführt?
Sämtliche Messverfahren sind CE-zertifiziert und entsprechend MPG geprüft und zugelassen. Invasive Untersuchungen werden nicht durchgeführt, mit Ausnahme von Blutentnahmen und einer venösen Kontrastmittelapplikation.

c) Handelt es sich um ein Vorhaben nach § 8 des Gesetzes zur Regelung des Transfusionswesens (TFG)?
ja ☒ nein

7. Handelt es sich um einen Versuch nach

§ 23 Strahlenschutzverordnung? ☒ ja nein

§ 28 Röntgenverordnung? ☒ ja nein

8. Typ der Studie:

- offen
- ☒ blind
- doppelblind
- ☒ vergleichend
- ☒ randomisiert
- multizentrisch
- ☒ Feldstudie
- Pilotstudie

9. Wissenschaftliche Begründung des Projekts, insbesondere:

a. Erläuterung des Versuchsziels

Das Ziel der Untersuchung ist die Evaluierung eines intensiven körperlichen Krafttrainings auf leistungsphysiologische und gesundheitsrelevante muskuläre und kardiale Größen bei untrainierten Männern im mittleren Lebensalter unter besonderer Berücksichtigung moderner bildgebender Verfahren. Aus radiologischer Sicht ist das Ziele der Studie die Identifikation typischer Kenngrößen der physiologischen Adaption der Körperzusammensetzung und des Herz-Kreislauf-Systems nach unterschiedlichen Typen von Krafttraining (s.o.) sowie der Methodenvergleich kernspintomographischer mit computertomographischen Muskelquerschnitten des Oberschenkels sowie der gesamten und der regionalen Gesamtkörperzusammensetzung mittels MRT vs. dem Goldstandard DXA-Methode.

b. Darstellung des bisherigen Wissensstandes

Eine Vielzahl von wissenschaftlichen Untersuchungen berichten den positiven Effekt eines „Krafttrainings“ auf funktionelle und gesundheitsrelevante physiologische und metabolische Größen (Übersicht in (1, 3, 10, 11)) bei Menschen in mittlerem Lebensalter. Obgleich viele dieser Daten bereits in frühen Studien mit suboptimaler Messmethodik/-technik und schlechter Reproduzierbarkeit evaluiert wurden, gelten sie in der wissenschaftlichen Literatur als absolut verlässlich und werden vielfach zitiert. Moderne bildgebende Verfahren wie die Kernspintomographie (MRT) oder die Computertomographie (CT) im Verbund mit valider Segmentierungstechnologie und quantitativer Analyse haben die in der Vergangenheit eingesetzten Verfahren mit suboptimaler Auflösung und ausschließlich qualitativer Auswerteprozedur bereits in vielen Bereichen ersetzt. So können Risikofaktoren wie die Körperfettverteilung derzeit schon quantifiziert und der Einfluss einer Intervention validiert werden (12-14). Auch die muskuläre Komponente der Körperzusammensetzung und insbesondere der Muskelquerschnitt wurde von einigen neueren Untersuchungen mittels moderner bildgebender Verfahren wie MRT oder CT untersucht (15, 16), die allerdings auf keine validierte Segmentierungs- und Quantifizierungssoftware zurückgreifen konnten.

Dies trifft ebenfalls für die kardiale Volumetrie mittels MRT zu, die derzeit als Goldstandard für die links- und rechtsventrikuläre Volumen- und Massenbestimmung gilt (17). Im Gegensatz zu echokardiographischen Untersuchungen handelt es sich bei der kardialen MRT um ein dreidimensionales Verfahren, welches dadurch eine wesentlich genauere morphologische Darstellung des Herzens ermöglicht (18) und somit u.a.

interventionsbedingte Veränderungen früher zu identifizieren vermag. Darüber hinaus werden sportliche Belastungen immer wieder mit einem plötzlichen Herztod, insbesondere bei männlichen Athleten, assoziiert (23-24). Ob hierfür pathologische morphologische Veränderungen des Myokards durch körperliches Training oder angeborene Organerkrankungen zu Grunde liegen, ist unklar.

10. Angaben zur Nutzen-Risiko-Relation

a. Welcher Nutzen ist von den Ergebnissen der Studie zu erwarten

aa) für die Versuchsteilnehmer?

Steigerung der körperlichen Fitness und physischen Attraktivität durch das Trainingsprogramm. Zudem Verminderung des metabolischen und kardiovaskulären Gesundheitsrisikos und Verbesserung der funktionellen Kapazität. Ggf. Detektion relevanter kardiologischer Befunde (Herzklappendefekte, Kardiomyopathie, Fehlbildungen).

ab) für die Heilkunde?

Beschreibung physiologischer muskulärer, metabolischer, physiologischer und kardialer Adaptation nach intensivem Krafttraining u.a. als Voraussetzung zur Abgrenzung pathologischer Veränderungen und Identifizierung geeigneter Verfahren zur Kontrolle von Trainingseffekten. Sekundäres Ziel ist es, Risikopatienten zu identifizieren, die vor Aufnahme eines spezifischen Trainings ein ausführliches Untersuchungsprogramm durchführen sollten, um Sekundärschäden zur vermeiden.

ac) für die Wissenschaft (z.B. Ergebnisse, die nicht unmittelbar therapeutischen Zwecken dienen)?

Verbesserter Einblick in die Grundlagen trainingsinduzierter muskulärer und kardiovaskulärer Adaptation als Basis optimaler sportwissenschaftlicher Trainings-empfehlungen. Evaluierung und Weiterentwicklung von bildgebenden Untersuchungs-verfahren und computerunterstützter Evaluationssoftware zur Diagnostik und zum Monitoring von Interventionseffekten.

b. Mit welchem Risiko ist die Studie für die Versuchsteilnehmer verbunden?

ba) Welcher Art sind die Risiken? Risikoeinschätzung, vorhersehbare Risiken der Behandlung und sonstiger studienbedingter Verfahren, die eingesetzt werden sollen (einschließlich Schmerz, Unannehmlichkeiten, Beschwerden, Verletzung der persönlichen Integrität und Maßnahmen zur Vermeidung und/oder zur Behandlung von unvorhersehbaren/ unerwünschten Ereignissen)

Die Risiken der Intervention (konsequent angeleitetes Krafttrainingsprogramm) sind sehr gering und bleiben nicht zuletzt aufgrund der intensiven Betreuung deutlich hinten denen eines selbständig durchgeführten Muskeltrainings zurück. Selbstverständlich sind in den ersten Wochen der Intervention leichte trainingsbedingte Beschwerden wie bspw. DOMS (Muskelkater) zu erwarten.

Die radiologischen Verfahren Dual Energy X-Ray Absorptiometrie (Gesamtkörper-DXA) bzw. Computertomographie (medialer Anteil Oberschenkel) sind mit niedrigen Strahlendosen (< 10 µSv pro DXA-Messung) bzw. geringen relativen Dosen (<0.8 mSv/CT-Messung[6]) verbunden. Eine Genehmigung dieser Verfahren

---

[6] Dieser Wert wurde ohne Berücksichtigung entsprechender Schutzmaßnahmen (Abdecken der Gonaden mit Bleischutz errechnet.

wird beim Bundesamt für Strahlenschutz nach Vorliegen der Genehmigung der Ethikkommission selbstverständlich beantragt.

Bei der Blutentnahme und durch periphere Verweilkatheter für die Kontrastmittel-(KM)-Applikation sind Blutergüsse und Infektionen nie komplett ausgeschlossen. In der Kernspintomographie werden nur geringe KM-Mengen eingesetzt. Daher sind die Risiken bei einem Paravasat des KMs in das Weichteilgewebe sowie Nierenbelastungen und mögliche allergische KM-Reaktionen minimiert. Probanden mit relativen oder absoluten Kontraindikationen für eine MRT werden von der Studie ausgeschlossen (s.u.). Insbesondere Probanden mit Nierenfunktionsstörungen oder bekannten KM-Reaktionen. Die KM-Gabe ist notwendig, um relevante kardiale Vorerkrankungen auszuschließen (siehe Ausschlusskriterien), die ein Risiko für die Intervention darstellen können.

bb) Mit welcher Wahrscheinlichkeit ist zu erwarten, daß sich die Risiken realisieren? Wie sicher ist die Wahrscheinlichkeit abschätzbar?

Insgesamt besteht nur eine geringe Wahrscheinlichkeit, dass sich die Risiken realisieren. Die Messungen stellen Standardmessungen der klinischen Routine dar, die von geschultem Fachpersonal sachkundig ausgeführt werden. Die Belastungsvorgaben erfolgen individuell, basierend auf einer Leistungsdiagnostik, sodass eine Überforderung der Teilnehmer kaum zu erwarten ist.

c. Warum ist das mögliche Risiko im Verhältnis zu dem zu erwartenden Nutzen Ihrer Ansicht nach vertretbar?

Es besteht keine wesentlich über das Alltagsrisiko hinausgehende Gefährdung, jedoch ein hoher zu erwartender Nutzen für die Teilnehmer hinsichtlich der Steigerung der körperlichen Fitness, gesundheitsrelevanter Größen, Wohlbefinden, Attraktivität und Selbstwirksamkeit/Kontrollüberzeugung.

d. Werden Zwischenergebnisse ausgewertet, um einen Trend zu erkennen?
ja ☒ nein, kurzer Interventionszeitraum macht Zwischenanalyse inadäquat.

e. Sind Kriterien festgelegt worden, bei deren Eintreten der Versuch geändert oder abgebrochen werden soll? ja, welche? ☒ nein

(allerdings Abbruch des jeweiligen Testverfahrens bei Unwohlsein, oder generell auf Wunsch des Patienten)

11. Bei klinischen Prüfungen nach MPG:

a. Welches Medizinprodukt soll geprüft werden? **entfällt**

b. Wird die klinische Prüfung von einer entsprechend qualifizierten und befugten Person geleitet, die mindestens eine zweijährige Erfahrung in der klinischen Prüfung von Medizinprodukten nachweisen kann? ja nein

c. Wurde (soweit erforderlich) eine dem jeweiligen Stand der wissenschaftlichen Erkenntnisse entsprechende biologische Sicherheitsprüfung oder sonstige für die vorgesehene Zweckbestimmung des Medizinproduktes erforderliche Prüfung durchgeführt? ja nein

d. Wurde (soweit erforderlich) die sicherheitstechnische Unbedenklichkeit für die Anwendung des Medizinproduktes unter Berücksichtigung des Standes der Technik sowie der Arbeitsschutz- und Unfallverhütungsvorschriften nachgewiesen?
ja nein

e. Ist der Leiter der klinischen Prüfung über die Ergebnisse der biologischen Sicherheitsprüfung und die voraussichtlich mit der klinischen Prüfung verbundenen Risiken informiert worden? ja nein

12. a) Ist die Mitarbeit eines Statistikers vorgesehen? ☒ ja nein
b) Welche statistischen Methoden sollen benutzt werden?

Komplettes statistisches Instrumentarium zur Erfassung von Effekten (bspw. Varianzanalysen/nicht parametrische Tests zur Identifikation von Zwischengruppenunterschieden). Zudem regressionsanalytische Modelle zur Aufklärung von Varianzen.

13. a) Handelt es sich um eine multizentrische Studie (d.h. eine nach einem *einzigen* Prüfplan durchgeführte Studie, die in mehr als einer Prüfstelle erfolgt und daher von mehr als einem Prüfer vorgenommen wird)? ja ☒ nein

b) Wurden/Werden an anderer Stelle Studien mit demselben oder einem ähnlichen Ziel durchgeführt? ja, wo? ☒ nein

Es wurden in der Vergangenheit bereits mehrere Untersuchungen mit dem Ziel der Evaluierung eines gesundheitssportlichen Trainings auf muskuläre, physiologische, metabolische und kardiale Parameter (meist isoliert) durchgeführt (s.o.). Diese Studie ist jedoch die erste, die u.a. den Effekt eines intensiven Kraft-/Powertrainings auf muskuläre Parameter, Körperzusammensetzung, metabolische und kardiovaskuläre Größen bei untrainierten Männern in mittlerem Lebensalter auch mittels moderner bildgebender Verfahren und Auswertesoftware evaluiert.

14. Wer hat die Studie initiiert? **Institut für Medizinische Physik**
15. Wer finanziert sie? (*Bitte geben Sie an, ob Drittmittel von nichtöffentlicher Seite beantragt werden. Falls ja, in welcher Höhe?*)

Derzeit sind noch keine Mittel beantragt, es werden jedoch nach positivem Votum der Ethikkommission versucht über unterschiedliche Ebenen Drittmittel zu generieren.

16. Die Aufwandsentschädigung wird übernommen von (*bitte Ansprechpartner benennen*):

Institut für Medizinische Physik (Direktor: Prof. Dr. Dr. W.A. Kalender)

Ansprechpartner: Prof. Dr. Wolfgang Kemmler

III. Angaben zu den Versuchsteilnehmern

1. Anzahl (*bei vergleichenden Studien bitte Aufteilung auf Gruppen angeben*)

80 Personen gesamt:
- Gruppe 1, n = 40: High Intensity Resistance Training (HIT)[7]
- Gruppe 2, n = 40: Kontrollgruppe (Wartegruppe; im Anschluss „Power-Training")

Bei Nullhypothesen-basierten Studien:
Wurde eine formale Fallzahlschätzung vorgenommen?

☒ ja, Basis CSA-Oberschenkelmuskulatur nein

2. Alter und Geschlecht (*bitte geben Sie das Alter der Versuchsteilnehmer sowie die als Ausschlusskriterien vorgesehenen Ober- und Untergrenzen an*)

[7] Training mit relativ hoher Reizintensität (≥70% des Einwiederholungsmaximums: 1RM)

Männer, 30. - 50. Lebensjahr

3. Status: Handelt es sich bei den Versuchsteilnehmern um
   ☒ gesunde Personen
   schwangere oder stillende Frauen
   Kinder oder Jugendliche
   einschlägig Erkrankte (*bitte geben Sie die Krankheit und das Stadium an*)
   Personen, die an anderen Krankheiten leiden? (Insbesondere: psychische Krankheiten, die Zweifel an der Geschäfts- oder Einsichtsfähigkeit begründen)

4. Welche sonstigen **Einschlusskriterien** (z.B. erlaubte Begleitmedikation) sind vorgesehen?

   - initial Untrainierte (während der vergangenen 2 Jahre: ≤1 h/Woche Sport mit positivem Effekt auf die Muskulatur; ≤2 h/Woche Sport gesamt)

5. Welche sonstigen **Ausschlusskriterien** (z.B. fortgeschrittene Nieren- oder Leber-insuffizienz, verbotene Begleitmedikation etc.) sind vorgesehen?

   - Geschichte leistungssportlicher Ausübung von Disziplinen mit erheblicher Relevanz für Körperzusammensetzung und Kraftfähigkeiten
   - pathologische muskuläre, metabolische und kardiale Veränderungen oder Entzündungen; deutlich eingeschränkte Gelenksbeweglichkeit in Knie und Hüfte.
   - Medikamente/Erkrankungen mit relevantem Einfluss auf Körperzusammensetzung und Herz-Kreislauf-System
   - sehr geringe körperliche Leistungsfähigkeit (< 100 Watt auf dem Fahrradergometer)
   - schwere Adipositas (BMI > 35 kg/m^2)
   - Abwesenheit ≥ 2 Wochen während des Interventionszeitraums
   - Geplante Aufnahme einer relevanten parallelen Trainingsmaßnahme
   - Kontraindikationen gegen MRT (Klaustrophobie, Herzschrittmacher, magnetisier-bare intracorporale Fremdkörper); Körpermaße die eine MRT-Messung verhindern
   - Drogenmissbrauch

6. Sollen auch Personen teilnehmen, die auf gerichtliche oder behördliche Anordnung in einer Anstalt verwahrt werden?
   ja ☒ nein

7. Sollen auch Personen teilnehmen, die sich schon für andere Forschungsvorhaben zur Verfügung gestellt haben?
   ja ☒ nein
   wie lange muss die letzte Teilnahme zurückliegen?

8. Bei Studien an Minderjährigen (oder sonst nicht geschäftsfähigen Personen) **entfällt**
   a. Warum kann die Studie nicht an Erwachsenen (voll Geschäftsfähigen) durchgeführt werden?

   b. Sind Aufklärung und Einwilligung der (des) gesetzlichen Vertreter(s) gewährleistet? (*bitte vorformulierte Erklärung beifügen*)
   ja nein, weil

c. Sind zusätzliche Aufklärung und Einwilligung der minderjährigen (nicht voll geschäftsfähigen) Versuchsteilnehmer gewährleistet, die selbst in der Lage sind, Wesen, Bedeutung und Tragweite des Versuchs einzusehen und ihren Willen danach zu bestimmen?

ja nein

9. Probandenversicherung

Wird zugunsten der Versuchsteilnehmer eine Versicherung abgeschlossen?

ja (*bitte Police beifügen, aus der die Versicherungsgesellschaft und die Höhe der Versicherungsleistung hervorgeht)*

☒ nein

10. Schweigepflicht/Datenschutz

Werden die ärztlichen Schweigepflicht- und die Datenschutzbestimmungen beachtet?

ja

11. Entgelt für Probanden

Soll den Versuchsteilnehmern ein Entgelt (Aufwandsentschädigung o.ä.) gezahlt werden?

ja, in Höhe von EUR ☒ nein

12. Wie sollen die Versuchsteilnehmer über Wesen, Bedeutung und Tragweite der Studie **aufgeklärt** werden?

Bitte in deutscher Sprache beifügen:

Dokumentation des Inhalts der Patientenaufklärung durch die/den versuchsdurchführende/n Ärztin/Arzt (Merkblatt), insbesondere mit Hinweisen über:

- **Ziele und Methoden** der Studie;
- **Nutzen und Risiko** der Studie;
- bekannte und möglicherweise zu erwartende **Wirkungen und Nebenwirkungen** von Medikamenten;
- Eingriffe, die nur aus wissenschaftlichen Gründen erfolgen;
- ein angebrachtes Verhalten des Patienten während und nach dem Versuch;
- die **Widerruflichkeit** einer Einwilligung;
- das Bestehen und den Umfang der gesetzlichen **Probandenversicherung** (Name/Anschrift/Telefon/Fax der Versicherungsgesellschaft, Nummer der Versicherungspolice) sowie die danach von der Versuchsperson zu beachtenden Obliegenheiten;
- **Ausschlusskriterien** (z.B. Schwangerschaft/Stillzeit);
- Name und Telefon des **Ansprechpartner**s vor Ort.
- **Besondere Aufklärung** über die Situation
  a. bei der randomisierten Studie
  f. beim Blind- und Doppelblindversuch.

13. Wie sollen die Versuchsteilnehmer ihre **Einwilligung** in die Teilnahme an der Studie erklären? (*bitte formulierte deutschsprachige Erklärung mit datenschutzrechtlicher Einwilligungserklärung beifügen*)

Schriftliche Einverständniserklärung nach umfangreicher mündlicher und schriftlicher Aufklärung über Ziele, Nutzen und Risiken der Untersuchung.

Ich weiß, daß auch bei einer positiven Beurteilung des Vorhabens durch die Ethik-Kommission der Medizinischen Fakultät der FAU Erlangen-Nürnberg die ärztliche und juristische Verantwortung für die Durchführung des Projektes uneingeschränkt bei der Leiterin/dem Leiter verbleibt.

Erlangen/Nürnberg
Datum ....

Unterschrift des/der Antragstellers/in

____
(Name in Druckbuchstaben)

Unterschrift der/des Leiterin/Leiters der Einrichtung, in der das Vorhaben durchgeführt werden soll.
Mit der Durchführung des Forschungsvorhabens einverstanden:

Datum ....

Unterschrift des/der Leiters/Leiterin der Einrichtung

____
(Name in Druckbuchstaben)

EK_May09/mit Unterschriftsblatt

Literatur

1. Benson AC, Torode ME, Fiatarone Singh MA. Effects of resistance training on metabolic fitness in children and adolescents: a systematic review. *Obes Rev*. 2008;9(1):43-66.
2. Kelley GA, Kelley KS. Impact of progressive resistance training on lipids and lipoproteins in adults: a meta-analysis of randomized controlled trials. *Prev Med*. 2009;48(1):9-19.
3. Latham NK, Bennett DA, Stretton CM, Anderson CS. Systematic review of progressive resistance strength training in older adults. *J Gerontol A Biol Sci Med Sci*. 2004;59(1):48-61.
4. Macaluso A, De Vito G. Muscle strength, power and adaptations to resistance training in older people. *Eur J Appl Physiol*. 2004;91:450-472.
5. Snowling NJ, Hopkins WG. Effects of different modes of exercise training on glucose control and risk factors for complications in type 2 diabetic patients: a meta-analysis. *Diabetes Care*. 2006;29(11):2518-27.
6. Weineck J. *Optimales Training* Erlangen: Spitta-Verlag; 2007.
7. Weineck J. *Sportbiologie*. Vol. 10 Balingen: Spitta Verlag; 2009.
8. Wijndaele K, Beunen G, Duvigneaud N, et al. A continuous metabolic syndrome risk score: utility for epidemiological analyses. *Diabetes Care*. 2006;29(10):2329.
9. Wilson PW, D'Agostino RB, Levy D, Belanger AM, Silbershatz H, Kannel WB. Prediction of coronary heart disease using risk factor categories. *Circulation*. 1998;97(18):1837-47.
10. Asikainen TM, Kukkonen-Harjula K, Miilunpalo S. Exercise for health for early postmenopausal women: a systematic review of randomised controlled trials. *Sports Med*. 2004;34(11):753-78.
11. Lagally KM, Cordero J, Good J, Brown DD, McCaw ST. Physiologic and metabolic responses to a continuous functional resistance exercise workout. *J Strength Cond Res*. 2009;23(2):373-9.
12. Kay SJ, Fiatarone Singh MA. The influence of physical activity on abdominal fat: a systematic review of the literature. *Obes Rev*. 2006;7(2):183-200.
13. Kemmler W, von Stengel S, Engelke K, Haberle L, Mayhew JL, Kalender WA. Exercise, body composition, and functional ability: a randomized controlled trial. *Am J Prev Med*. 2010;38(3):279-87.
14. Lamb HJ. Total body fat distribution as part of multiorgan MR imaging: new tool for risk assessment in the metabolic syndrome? *Radiology*. 2010;257(2):307-8.
15. Weiss EP, Racette SB, Villareal DT, et al. Lower extremity muscle size and strength and aerobic capacity decrease with caloric restriction but not with exercise-induced weight loss. *J Appl Physiol*. 2007;102(2):634-40.
16. Valtonen A, Poyhonen T, Sipila S, Heinonen A. Effects of aquatic resistance training on mobility limitation and lower-limb impairments after knee replacement. *Arch Phys Med Rehabil*;91(6):833-9.
17. Petersen SE, Hudsmith LE, Robson MD, et al. Sex-specific characteristics of cardiac function, geometry, and mass in young adult elite athletes. *J Magn Reson Imaging*. 2006;24(2):297-303.
18. Grothues F, Smith GC, Moon JC, et al. Comparison of interstudy reproducibility of cardiovascular magnetic resonance with two-dimensional echocardiography in normal subjects and in patients with heart failure or left ventricular hypertrophy. *Am J Cardiol*. 2002;90(1):29-34.

An die Geschäftsstelle der
Ethik-Kommission
der Medizinischen Fakultät
der FAU Erlangen-Nürnberg
Krankenhausstr. 12
91054 Erlangen

Antrag
an die Ethik-Kommission
der Medizinischen Fakultät

*Bitte **in deutscher Sprache** ausfüllen,*
Zutreffendes bitte ankreuzen.
Für multizentrische Studien mit Vorvotum einer nach Landesrecht gebildeten zuständigen Ethik-Kommission können Sie das verkürzte Antragsformular verwenden, abzurufen unter: http://www.ethik.med.uni-erlangen.de (Anschlussvotum)

Antrag auf Beurteilung eines Forschungsprojektes (keine Arzneimittelprüfung)

bitte 9-fach einschließlich Anlagen einreichen sowie 1-mal in elektronischer Fassung

Titel des Projektes:
Einfluss eines 16-wöchigen Kraft-Trainingsprogramms auf leistungsphysiologische und gesundheitsrelevante muskuläre und kardiale Größen bei untrainierten Männern im mittleren Lebensalter. Eine randomisierte kontrollierte Interventionsstudie mit modernen, bildgebenden Verfahren.

I. Projektleitung

1. Name der/des verantwortlichen Projektleiterin/s an der FAU:
Professor Dr. Wolfgang Kemmler[1], Osteoporose-Forschungszentrum, Institut für Medizinische Physik, FAU (Direktor: Professor Dr. Dr. Willi A. Kalender)
App.-Nr. 23999; E-Mail: wolfgang.kemmler@imp.uni-erlangen.de

Angaben über die Qualifikation der/des Versuchsleiterin/s:
Promotion, Habilitation (liegen bereits vor)

2. a) Weitere Teilnehmer/innen vor Ort (alphabetische Reihenfolge):
Prof. Dr. Klaus Engelke, IMP, FAU
PD. Dr. Michael Lell, Radiologisches Institut, FAU
Prof. Dr. Harald Quick, IMP, FAU
PD. Dr. Axel Schmid, Radiologisches Institut, FAU
Dr. Michael Scharf, Radiologisches Institut, FAU
Dr. Simon von Stengel, Osteoporoseforschungszentrum, FAU
Andreas Wittke, IMP, FAU

b) Weitere Prüfzentren (bei multizentrischen Studien): keine

3. Handelt es sich bei diesem Antrag um ein bereits von der Ethik-Kommission der Medizinischen Fakultät der Friedrich-Alexander-Universität Erlangen-Nürnberg begutachtetes Projekt?

ja (bitte lfd. Nr. angeben) ☒ nein

II. Forschungsvorhaben

1. Geplanter Beginn der Studie: September 2012 // voraussichtliches Ende: August 2013
Dauer der Studienteilnahme für den einzelnen Probanden:

Intervention (s.u.): 4 Monate // 8 Monate (Kontrollgruppen-Wartelisten-Design)
Untersuchung (s.u.): ca. 100 min jeweils zu Beginn und Studienende

2. Kurzer Abriss des Projektes *(maximal 1,5 Seiten)*:

Einführung und Fragestellung
Krafttraining gilt auch bei intensiver Durchführung als gesundheitsfördernde Intervention, die sich bei einem zunehmenden Anteil der Bevölkerung immer größerer Beliebtheit erfreut. Neben Körperformung und Attraktivität steht für viele Menschen der präventive Aspekt des Muskeltrainings im Vordergrund. Tatsächlich weisen eine Vielzahl von Untersuchungen (1-5) positive Effekte eines regelmäßig durchgeführten, „überschwelligen“ Trainings nach, so auf gesundheitsrelevante muskuläre, physiologische, metabolische und kardiovaskuläre Parameter wie beispielsweise die Körperzusammensetzung/funktionelle Kapazität, Blutfette/Lipoproteine oder Glucoseintoleranz/ Insulinsensitivität. Zudem ist anzunehmen, dass auch relativ rasche funktionelle und morphologische Anpassungserscheinungen des Herzens nachweisbar sind (6, 7). Allerdings liegen keine kernspintomographischen Längsschnittuntersuchungen vor, welche die physiologischen Adaptationserscheinungen des Herzens nach einem mehrmonatigen intensiven Krafttraining bei inaktiven Personen evaluieren. Parallel dazu sind die vorliegenden Methoden, mit denen die Körperzusammensetzung und die muskuläre Massenentwicklung bislang evaluiert wurden, als suboptimal einzuschätzen. Um eine valide und reliable Erfassung der Veränderung muskulärer, physiologischer und kardiologischer Größen als Reaktion auf ein gesundheitsorientiertes Muskeltraining zu gewährleisten, ist der messmethodische Schwerpunkt der vorliegenden Untersuchung auf den Einsatz moderner bildgebender Verfahren (Kernspintomographie (MRT), Computertomographie (CT), Dual Energy X-Ray Absorptiometry (DXA)) und moderner Segmentierungs- und Quantifizierungssoftware gerichtet.
Ziel der Untersuchung ist somit die Evaluierung der Effekte eines intensiven 16-wöchigen Krafttrainingsprogramms auf leistungsphysiologische und gesundheitsrelevante muskuläre und kardiale Größen bei untrainierten Männern im mittleren Lebensalter unter besonderer Berücksichtigung bildgebender Verfahren.

Design:
Randomisierte, kontrollierte, teilverblindete Studie mit Wartegruppe (cross-over)

Endpunkte
Primäre Endpunkte:

- Muskelquerschnitt der Oberschenkelmuskulatur (MRT, CT)
- Intraabdominale Fettmasse (MRT)

Sekundäre Endpunkte:

- Kraftfähigkeiten (u.a. abhängige Variable) [1]
- Gesamtkörperfett und Muskelmasse sowie regionale Verteilung (DXA, MRT)
- Metabolisches Syndrom-Score (8), und 10 Jahres-CHD-Risiko (9)
- Kardiale Masse und enddiastolisches Volumen (MRT)
- Hormonelle Regulation (u.a. freies Testosteron, hGH, Cortisol)
- Schmerzintensität, Quality of Life

[1] Ein Ziel der Studie ist die Identifikation von Variablen, welche die Varianz des Kraft-/Leistungszuwachs am höchsten aufklären.

Stichprobe

Zwei-Gruppen Design, randomisiert, z.T. cross-over (mit Wartegruppe)[2]

- Gruppe 1, n = 40: High Intensity Resistance Training (HIT)[3]
- Gruppe 2, n = 40: Kontrollgruppe (Wartegruppe; im Anschluss „Power-Training[4]“)

Interventionsprogramm

Gruppe 1: HIT mit konventioneller Bewegungsgeschwindigkeit (TUT[5] 2s-1s-2s)

Periodisiertes, progressives Krafttraining über 16 Wochen, basierend auf individuellen Trainingsplänen auf der Basis von 1 RM/x RM-Tests (1, 4, 10 Wochen), zunehmende Intensivierung der Reizhöhe (bis Woche 8), anschließend periodisiertes HIT (70-92,5%, 1RM) mit 2-3 Trainingseinheiten je Woche (1-2 gemeinsame, überwachte Trainingseinheiten; 1-2 Trainingseinheiten in Eigenregie (Vorgaben über Trainingsplan)).

Gruppe 2 (ehemalige Kontrollgruppe): Power-Training (TUT: ϟ-1s-2s)

s.o. aber explosive Ausführung im konzentrischen Bewegungsbereich nach initialer Konditionierung über 6 Wochen. Vergleichbares Trainingsprotokoll, allerdings im Intensitätsbereich von 40-70%, 1RM.

3. Studienbezogene Maßnahmen:

Bitte beschreiben Sie hier alle Maßnahmen, die studienbedingt durchgeführt werden sowie alle erforderlichen Abweichungen von der üblichen Routine-Behandlung:

Interventionsprogramm s.o.

Messungen (jeweils basal und nach 16 Wochen)

Bildgebende Verfahren:

Ganzkörper-MRT und Ganzkörper-DXA zur Erfassung der gesamten und regionalen Körperzusammensetzung.

Lokale MRT und CT am Oberschenkel (über „Muskelbauch“)

Kardiale MRT-Untersuchung mit Kontrastmittel i.v. (Analyse fibrotischer Herzmuskelveränderungen, Strain der Herzmuskulatur, enddiastolisches Volumen (EDV), endsystolisches Volumen (ESV), Schlagvolumen (SV), Ejektionsfraktion (EF) und myokardiale Masse (MM), Herzmuskeldicke).

Blutdruck und Herzfrequenzverhalten in Ruhe

Körperliche Fitness:

Erfassung unterschiedlicher Kraftfähigkeiten (u.a. 1 RM, Schnellkraft) mittels isokinetischem Dynamometer.

Psychosoziale Parameter

Befindlichkeit und Schmerzparameter, QoL (Fragebogen)

Labor

Blutfette/Lipoproteine, Glukose, Insulin, HbAC1, Entzündungsmarker

Testosteron, freies Testosteron, hGH, Cortisol; (evt. noch Parameter des Immunsystems)

Ernährungsanalyse

Analyse über 4 Tage (standardisierte Protokolle)

Anamnese und Risikofaktorenprofil über Fragebogen

---

[2] Die Wartegruppe dient zunächst als parallele Kontrollgruppe zur Interventionsgruppe. Nach Abschluss dieses Untersuchungsabschnittes wird mit dieser Gruppe ebenfalls eine 16-wöchige Intervention durchgeführt, sodass ein eingeschränktes 3-Gruppendesign generiert wird.

[3] Training mit relativ hoher Reizintensität (≥70% des Einwiederholungsmaximums: 1RM)

[4] Training mit explosiver Bewegungsausführung im konzentrischen Bereich (bei vglw. geringer Reizintensität im Bereich 40-60% 1RM)

[5] **T**ime **U**nder **T**ension: Dauer der jeweiligen Bewegungsabschnitte, konzentrisch – isometrisch – exzentrisch in Sec.

4. Wird die Studie gemäß der von der 48. Generalversammlung des Weltärztebundes in Somerset West revidierten Deklaration von Helsinki aus dem Jahre 1996 durchgeführt? Bitte angeben, ob alle anderen Erprobungsmöglichkeiten ausgeschöpft wurden.

Ja, die Intervention wurde in vorhergehenden Studien bereits validiert und optimiert. Die Messtechnologie ist ebenfalls etabliert, wurde aber bislang nur suboptimal zur Validierung der hier genannten Fragestellung eingesetzt.

5. Art des Forschungsvorhabens:
Handelt es sich um
eine diagnostische Prüfung?
eine therapeutische Prüfung?
eine Verträglichkeitsprüfung?
☒ einen ausschließlich wissenschaftlichen Versuch?

6. Gesetzliche Grundlagen

a) Handelt es sich um eine Untersuchung, die dazu bestimmt ist, klinische oder pharmakologische Wirkungen von Arzneimitteln zu erforschen oder nachzuweisen oder Nebenwirkungen festzustellen oder die Resorption, die Verteilung, den Stoffwechsel oder die Ausscheidung zu untersuchen, **mit dem Ziel, sich von der Unbedenklichkeit oder Wirksamkeit des Arzneimittels zu überzeugen** (klinische Prüfung eines Arzneimittels nach §§ 40 Arzneimittelgesetz)?

nein

Bitte begründen. Erläuterungen zum Antrag auf Bewertung einer klinischen Arzneimittelprüfung nach § 40 AMG finden Sie unter http://www.ethik.med.uni-erlangen.de/

b) Handelt es sich um eine klinische Prüfung nach § 20 Medizinproduktegesetz (MPG)?
ja ☒ nein
Bitte begründen. Liegt eine CE-Zertifizierung für das Medizinprodukt vor? Werden zusätzlich invasive oder andere belastende Untersuchungen durchgeführt?
Sämtliche Messverfahren sind CE-zertifiziert und entsprechend MPG geprüft und zugelassen. Invasive Untersuchungen werden nicht durchgeführt, mit Ausnahme von Blutentnahmen und einer venösen Kontrastmittelapplikation.

c) Handelt es sich um ein Vorhaben nach § 8 des Gesetzes zur Regelung des Transfusionswesens (TFG)?
ja ☒ nein

7. Handelt es sich um einen Versuch nach

§ 23 Strahlenschutzverordnung? ☒ ja nein

§ 28 Röntgenverordnung? ☒ ja nein

8. Typ der Studie:

- offen
- ☒ blind
- doppelblind
- ☒ vergleichend
- ☒ randomisiert
- multizentrisch
- ☒ Feldstudie
- Pilotstudie

9. Wissenschaftliche Begründung des Projekts, insbesondere:

a. Erläuterung des Versuchsziels

Das Ziel der Untersuchung ist die Evaluierung eines intensiven körperlichen Krafttrainings auf leistungsphysiologische und gesundheitsrelevante muskuläre und kardiale Größen bei untrainierten Männern im mittleren Lebensalter unter besonderer Berücksichtigung moderner bildgebender Verfahren. Aus radiologischer Sicht ist das Ziele der Studie die Identifikation typischer Kenngrößen der physiologischen Adaption der Körperzusammensetzung und des Herz-Kreislauf-Systems nach unterschiedlichen Typen von Krafttraining (s.o.) sowie der Methodenvergleich kernspintomographischer mit computertomographischen Muskelquerschnitten des Oberschenkels sowie der gesamten und der regionalen Gesamtkörperzusammensetzung mittels MRT vs. dem Goldstandard DXA-Methode.

b. Darstellung des bisherigen Wissensstandes

Eine Vielzahl von wissenschaftlichen Untersuchungen berichten den positiven Effekt eines „Krafttrainings“ auf funktionelle und gesundheitsrelevante physiologische und metabolische Größen (Übersicht in (1, 3, 10, 11)) bei Menschen in mittlerem Lebensalter. Obgleich viele dieser Daten bereits in frühen Studien mit suboptimaler Messmethodik/-technik und schlechter Reproduzierbarkeit evaluiert wurden, gelten sie in der wissenschaftlichen Literatur als absolut verlässlich und werden vielfach zitiert. Moderne bildgebende Verfahren wie die Kernspintomographie (MRT) oder die Computertomographie (CT) im Verbund mit valider Segmentierungstechnologie und quantitativer Analyse haben die in der Vergangenheit eingesetzten Verfahren mit suboptimaler Auflösung und ausschließlich qualitativer Auswerteprozedur bereits in vielen Bereichen ersetzt. So können Risikofaktoren wie die Körperfettverteilung derzeit schon quantifiziert und der Einfluss einer Intervention validiert werden (12-14). Auch die muskuläre Komponente der Körperzusammensetzung und insbesondere der Muskelquerschnitt wurde von einigen neueren Untersuchungen mittels moderner bildgebender Verfahren wie MRT oder CT untersucht (15, 16), die allerdings auf keine validierte Segmentierungs- und Quantifizierungssoftware zurückgreifen konnten.

Dies trifft ebenfalls für die kardiale Volumetrie mittels MRT zu, die derzeit als Goldstandard für die links- und rechtsventrikuläre Volumen- und Massenbestimmung gilt (17). Im Gegensatz zu echokardiographischen Untersuchungen handelt es sich bei der kardialen MRT um ein dreidimensionales Verfahren, welches dadurch eine wesentlich genauere morphologische Darstellung des Herzens ermöglicht (18) und somit u.a.

interventionsbedingte Veränderungen früher zu identifizieren vermag. Darüber hinaus werden sportliche Belastungen immer wieder mit einem plötzlichen Herztod, insbesondere bei männlichen Athleten, assoziiert (23-24). Ob hierfür pathologische morphologische Veränderungen des Myokards durch körperliches Training oder angeborene Organerkrankungen zu Grunde liegen, ist unklar.

10. Angaben zur Nutzen-Risiko-Relation

a. Welcher Nutzen ist von den Ergebnissen der Studie zu erwarten

aa) für die Versuchsteilnehmer?

Steigerung der körperlichen Fitness und physischen Attraktivität durch das Trainingsprogramm. Zudem Verminderung des metabolischen und kardiovaskulären Gesundheitsrisikos und Verbesserung der funktionellen Kapazität. Ggf. Detektion relevanter kardiologischer Befunde (Herzklappendefekte, Kardiomyopathie, Fehlbildungen).

ab) für die Heilkunde?

Beschreibung physiologischer muskulärer, metabolischer, physiologischer und kardialer Adaptation nach intensivem Krafttraining u.a. als Voraussetzung zur Abgrenzung pathologischer Veränderungen und Identifizierung geeigneter Verfahren zur Kontrolle von Trainingseffekten. Sekundäres Ziel ist es, Risikopatienten zu identifizieren, die vor Aufnahme eines spezifischen Trainings ein ausführliches Untersuchungsprogramm durchführen sollten, um Sekundärschäden zur vermeiden.

ac) für die Wissenschaft (z.B. Ergebnisse, die nicht unmittelbar therapeutischen Zwecken dienen)?

Verbesserter Einblick in die Grundlagen trainingsinduzierter muskulärer und kardiovaskulärer Adaptation als Basis optimaler sportwissenschaftlicher Trainingsempfehlungen. Evaluierung und Weiterentwicklung von bildgebenden Untersuchungsverfahren und computerunterstützter Evaluationssoftware zur Diagnostik und zum Monitoring von Interventionseffekten.

b. Mit welchem Risiko ist die Studie für die Versuchsteilnehmer verbunden?

ba) Welcher Art sind die Risiken? Risikoeinschätzung, vorhersehbare Risiken der Behandlung und sonstiger studienbedingter Verfahren, die eingesetzt werden sollen (einschließlich Schmerz, Unannehmlichkeiten, Beschwerden, Verletzung der persönlichen Integrität und Maßnahmen zur Vermeidung und/oder zur Behandlung von unvorhersehbaren/ unerwünschten Ereignissen)

Die Risiken der Intervention (konsequent angeleitetes Krafttrainingsprogramm) sind sehr gering und bleiben nicht zuletzt aufgrund der intensiven Betreuung deutlich hinten denen eines selbständig durchgeführten Muskeltrainings zurück. Selbstverständlich sind in den ersten Wochen der Intervention leichte trainingsbedingte Beschwerden wie bspw. DOMS (Muskelkater) zu erwarten.

Die radiologischen Verfahren Dual Energy X-Ray Absorptiometrie (Gesamtkörper-DXA) bzw. Computertomographie (medialer Anteil Oberschenkel) sind mit niedrigen Strahlendosen (< 10 µSv pro DXA-Messung) bzw. geringen relativen Dosen (<0.8 mSv/CT-Messung[6]) verbunden. Eine Genehmigung dieser Verfahren

[6] Dieser Wert wurde ohne Berücksichtigung entsprechender Schutzmaßnahmen (Abdecken der Gonaden mit Bleischutz errechnet.

wird beim Bundesamt für Strahlenschutz nach Vorliegen der Genehmigung der Ethikkommission selbstverständlich beantragt.

Bei der Blutentnahme und durch periphere Verweilkatheter für die Kontrastmittel-(KM)-Applikation sind Blutergüsse und Infektionen nie komplett ausgeschlossen. In der Kernspintomographie werden nur geringe KM-Mengen eingesetzt. Daher sind die Risiken bei einem Paravasat des KMs in das Weichteilgewebe sowie Nierenbelastungen und mögliche allergische KM-Reaktionen minimiert. Probanden mit relativen oder absoluten Kontraindikationen für eine MRT werden von der Studie ausgeschlossen (s.u.). Insbesondere Probanden mit Nierenfunktionsstörungen oder bekannten KM-Reaktionen. Die KM-Gabe ist notwendig, um relevante kardiale Vorerkrankungen auszuschließen (siehe Ausschlusskriterien), die ein Risiko für die Intervention darstellen können.

bb) Mit welcher Wahrscheinlichkeit ist zu erwarten, daß sich die Risiken realisieren? Wie sicher ist die Wahrscheinlichkeit abschätzbar?

Insgesamt besteht nur eine geringe Wahrscheinlichkeit, dass sich die Risiken realisieren. Die Messungen stellen Standardmessungen der klinischen Routine dar, die von geschultem Fachpersonal sachkundig ausgeführt werden. Die Belastungsvorgaben erfolgen individuell, basierend auf einer Leistungsdiagnostik, sodass eine Überforderung der Teilnehmer kaum zu erwarten ist.

c. Warum ist das mögliche Risiko im Verhältnis zu dem zu erwartenden Nutzen Ihrer Ansicht nach vertretbar?

Es besteht keine wesentlich über das Alltagsrisiko hinausgehende Gefährdung, jedoch ein hoher zu erwartender Nutzen für die Teilnehmer hinsichtlich der Steigerung der körperlichen Fitness, gesundheitsrelevanter Größen, Wohlbefinden, Attraktivität und Selbstwirksamkeit/Kontrollüberzeugung.

d. Werden Zwischenergebnisse ausgewertet, um einen Trend zu erkennen?
ja ☒ nein, kurzer Interventionszeitraum macht Zwischenanalyse inadäquat.

e. Sind Kriterien festgelegt worden, bei deren Eintreten der Versuch geändert oder abgebrochen werden soll? ja, welche? ☒ nein

(allerdings Abbruch des jeweiligen Testverfahrens bei Unwohlsein, oder generell auf Wunsch des Patienten)

11. Bei klinischen Prüfungen nach MPG:

a. Welches Medizinprodukt soll geprüft werden? **entfällt**

b. Wird die klinische Prüfung von einer entsprechend qualifizierten und befugten Person geleitet, die mindestens eine zweijährige Erfahrung in der klinischen Prüfung von Medizinprodukten nachweisen kann? ja nein

c. Wurde (soweit erforderlich) eine dem jeweiligen Stand der wissenschaftlichen Erkenntnisse entsprechende biologische Sicherheitsprüfung oder sonstige für die vorgesehene Zweckbestimmung des Medizinproduktes erforderliche Prüfung durchgeführt? ja nein

d. Wurde (soweit erforderlich) die sicherheitstechnische Unbedenklichkeit für die Anwendung des Medizinproduktes unter Berücksichtigung des Standes der Technik sowie der Arbeitsschutz- und Unfallverhütungsvorschriften nachgewiesen?
ja nein

e. Ist der Leiter der klinischen Prüfung über die Ergebnisse der biologischen Sicherheitsprüfung und die voraussichtlich mit der klinischen Prüfung verbundenen Risiken informiert worden? ja nein

12. a) Ist die Mitarbeit eines Statistikers vorgesehen? ☒ ja nein
b) Welche statistischen Methoden sollen benutzt werden?

Komplettes statistisches Instrumentarium zur Erfassung von Effekten (bspw. Varianzanalysen/nicht parametrische Tests zur Identifikation von Zwischengruppenunterschieden). Zudem regressionsanalytische Modelle zur Aufklärung von Varianzen.

13. a) Handelt es sich um eine multizentrische Studie (d.h. eine nach einem *einzigen* Prüfplan durchgeführte Studie, die in mehr als einer Prüfstelle erfolgt und daher von mehr als einem Prüfer vorgenommen wird)? ja ☒ nein

b) Wurden/Werden an anderer Stelle Studien mit demselben oder einem ähnlichen Ziel durchgeführt? ja, wo? ☒ nein

Es wurden in der Vergangenheit bereits mehrere Untersuchungen mit dem Ziel der Evaluierung eines gesundheitssportlichen Trainings auf muskuläre, physiologische, metabolische und kardiale Parameter (meist isoliert) durchgeführt (s.o.). Diese Studie ist jedoch die erste, die u.a. den Effekt eines intensiven Kraft-/Powertrainings auf muskuläre Parameter, Körperzusammensetzung, metabolische und kardiovaskuläre Größen bei untrainierten Männern in mittlerem Lebensalter auch mittels moderner bildgebender Verfahren und Auswertesoftware evaluiert.

14. Wer hat die Studie initiiert? **Institut für Medizinische Physik**
15. Wer finanziert sie? (*Bitte geben Sie an, ob Drittmittel von nichtöffentlicher Seite beantragt werden. Falls ja, in welcher Höhe?*)

Derzeit sind noch keine Mittel beantragt, es werden jedoch nach positivem Votum der Ethikkommission versucht über unterschiedliche Ebenen Drittmittel zu generieren.

16. Die Aufwandsentschädigung wird übernommen von (*bitte Ansprechpartner benennen*):

Institut für Medizinische Physik (Direktor: Prof. Dr. Dr. W.A. Kalender)

Ansprechpartner: Prof. Dr. Wolfgang Kemmler

III. Angaben zu den Versuchsteilnehmern

1. Anzahl (*bei vergleichenden Studien bitte Aufteilung auf Gruppen angeben*)

80 Personen gesamt:
- Gruppe 1, n = 40: High Intensity Resistance Training (HIT)[7]
- Gruppe 2, n = 40: Kontrollgruppe (Wartegruppe; im Anschluss „Power-Training“)

Bei Nullhypothesen-basierten Studien:
Wurde eine formale Fallzahlschätzung vorgenommen?

☒ ja, Basis CSA-Oberschenkelmuskulatur nein

2. Alter und Geschlecht (*bitte geben Sie das Alter der Versuchsteilnehmer sowie die als Ausschlusskriterien vorgesehenen Ober- und Untergrenzen an*)

---

[7] Training mit relativ hoher Reizintensität (≥70% des Einwiederholungsmaximums: 1RM)

Männer, 30. - 50. Lebensjahr

3. Status: Handelt es sich bei den Versuchsteilnehmern um
   ☒ gesunde Personen
   schwangere oder stillende Frauen
   Kinder oder Jugendliche
   einschlägig Erkrankte (*bitte geben Sie die Krankheit und das Stadium an*)
   Personen, die an anderen Krankheiten leiden? (Insbesondere: psychische Krankheiten, die Zweifel an der Geschäfts- oder Einsichtsfähigkeit begründen)

4. Welche sonstigen **Einschlusskriterien** (z.B. erlaubte Begleitmedikation) sind vorgesehen?
   - initial Untrainierte (während der vergangenen 2 Jahre: ≤1 h/Woche Sport mit positivem Effekt auf die Muskulatur; ≤2 h/Woche Sport gesamt)

5. Welche sonstigen **Ausschlusskriterien** (z.B. fortgeschrittene Nieren- oder Leber-insuffizienz, verbotene Begleitmedikation etc.) sind vorgesehen?
   - Geschichte leistungssportlicher Ausübung von Disziplinen mit erheblicher Relevanz für Körperzusammensetzung und Kraftfähigkeiten
   - pathologische muskuläre, metabolische und kardiale Veränderungen oder Entzündungen; deutlich eingeschränkte Gelenksbeweglichkeit in Knie und Hüfte.
   - Medikamente/Erkrankungen mit relevantem Einfluss auf Körperzusammensetzung und Herz-Kreislauf-System
   - sehr geringe körperliche Leistungsfähigkeit (< 100 Watt auf dem Fahrradergometer)
   - schwere Adipositas (BMI > 35 kg/m^2)
   - Abwesenheit ≥ 2 Wochen während des Interventionszeitraums
   - Geplante Aufnahme einer relevanten parallelen Trainingsmaßnahme
   - Kontraindikationen gegen MRT (Klaustrophobie, Herzschrittmacher, magnetisier-bare intracorporale Fremdkörper); Körpermaße die eine MRT-Messung verhindern
   - Drogenmissbrauch

6. Sollen auch Personen teilnehmen, die auf gerichtliche oder behördliche Anordnung in einer Anstalt verwahrt werden?
   ja ☒ nein

7. Sollen auch Personen teilnehmen, die sich schon für andere Forschungsvorhaben zur Verfügung gestellt haben?
   ja ☒ nein
   wie lange muss die letzte Teilnahme zurückliegen?

8. Bei Studien an Minderjährigen (oder sonst nicht geschäftsfähigen Personen) **entfällt**
   a. Warum kann die Studie nicht an Erwachsenen (voll Geschäftsfähigen) durchgeführt werden?

   b. Sind Aufklärung und Einwilligung der (des) gesetzlichen Vertreter(s) gewährleistet? (*bitte vorformulierte Erklärung beifügen*)
   ja nein, weil

c. Sind zusätzliche Aufklärung und Einwilligung der minderjährigen (nicht voll geschäftsfähigen) Versuchsteilnehmer gewährleistet, die selbst in der Lage sind, Wesen, Bedeutung und Tragweite des Versuchs einzusehen und ihren Willen danach zu bestimmen?

ja nein

9. Probandenversicherung

Wird zugunsten der Versuchsteilnehmer eine Versicherung abgeschlossen?

ja (*bitte Police beifügen, aus der die Versicherungsgesellschaft und die Höhe der Versicherungsleistung hervorgeht)*

☒ nein

10. Schweigepflicht/Datenschutz

Werden die ärztlichen Schweigepflicht- und die Datenschutzbestimmungen beachtet?

ja

11. Entgelt für Probanden

Soll den Versuchsteilnehmern ein Entgelt (Aufwandsentschädigung o.ä.) gezahlt werden?

ja, in Höhe von EUR ☒ nein

12. Wie sollen die Versuchsteilnehmer über Wesen, Bedeutung und Tragweite der Studie **aufgeklärt** werden?

Bitte in deutscher Sprache beifügen:

Dokumentation des Inhalts der Patientenaufklärung durch die/den versuchsdurchführende/n Ärztin/Arzt (Merkblatt), insbesondere mit Hinweisen über:

- **Ziele und Methoden** der Studie;
- **Nutzen und Risiko** der Studie;
- bekannte und möglicherweise zu erwartende **Wirkungen und Nebenwirkungen** von Medikamenten;
- Eingriffe, die nur aus wissenschaftlichen Gründen erfolgen;
- ein angebrachtes Verhalten des Patienten während und nach dem Versuch;
- die **Widerruflichkeit** einer Einwilligung;
- das Bestehen und den Umfang der gesetzlichen **Probandenversicherung** (Name/Anschrift/Telefon/Fax der Versicherungsgesellschaft, Nummer der Versicherungspolice) sowie die danach von der Versuchsperson zu beachtenden Obliegenheiten;
- **Ausschlusskriterien** (z.B. Schwangerschaft/Stillzeit);
- Name und Telefon des **Ansprechpartner**s vor Ort.
- **Besondere Aufklärung** über die Situation
  a. bei der randomisierten Studie
  f. beim Blind- und Doppelblindversuch.

13. Wie sollen die Versuchsteilnehmer ihre **Einwilligung** in die Teilnahme an der Studie erklären? (*bitte formulierte deutschsprachige Erklärung mit datenschutzrechtlicher Einwilligungserklärung beifügen*)

Schriftliche Einverständniserklärung nach umfangreicher mündlicher und schriftlicher Aufklärung über Ziele, Nutzen und Risiken der Untersuchung.

Ich weiß, daß auch bei einer positiven Beurteilung des Vorhabens durch die Ethik-Kommission der Medizinischen Fakultät der FAU Erlangen-Nürnberg die ärztliche und juristische Verantwortung für die Durchführung des Projektes uneingeschränkt bei der Leiterin/dem Leiter verbleibt.

Erlangen/Nürnberg
Datum ....   Unterschrift des/der Antragstellers/in

____
(Name in Druckbuchstaben)

Unterschrift der/des Leiterin/Leiters der Einrichtung, in der das Vorhaben durchgeführt werden soll.
Mit der Durchführung des Forschungsvorhabens einverstanden:

Datum ....   Unterschrift des/der Leiters/Leiterin der Einrichtung

____
(Name in Druckbuchstaben)

EK_May09/mit Unterschriftsblatt

Literatur

1. Benson AC, Torode ME, Fiatarone Singh MA. Effects of resistance training on metabolic fitness in children and adolescents: a systematic review. *Obes Rev*. 2008;9(1):43-66.
2. Kelley GA, Kelley KS. Impact of progressive resistance training on lipids and lipoproteins in adults: a meta-analysis of randomized controlled trials. *Prev Med*. 2009;48(1):9-19.
3. Latham NK, Bennett DA, Stretton CM, Anderson CS. Systematic review of progressive resistance strength training in older adults. *J Gerontol A Biol Sci Med Sci*. 2004;59(1):48-61.
4. Macaluso A, De Vito G. Muscle strength, power and adaptations to resistance training in older people. *Eur J Appl Physiol*. 2004;91:450-472.
5. Snowling NJ, Hopkins WG. Effects of different modes of exercise training on glucose control and risk factors for complications in type 2 diabetic patients: a meta-analysis. *Diabetes Care*. 2006;29(11):2518-27.
6. Weineck J. *Optimales Training* Erlangen: Spitta-Verlag; 2007.
7. Weineck J. *Sportbiologie*. Vol. 10 Balingen: Spitta Verlag; 2009.
8. Wijndaele K, Beunen G, Duvigneaud N, et al. A continuous metabolic syndrome risk score: utility for epidemiological analyses. *Diabetes Care*. 2006;29(10):2329.
9. Wilson PW, D'Agostino RB, Levy D, Belanger AM, Silbershatz H, Kannel WB. Prediction of coronary heart disease using risk factor categories. *Circulation*. 1998;97(18):1837-47.
10. Asikainen TM, Kukkonen-Harjula K, Miilunpalo S. Exercise for health for early postmenopausal women: a systematic review of randomised controlled trials. *Sports Med*. 2004;34(11):753-78.
11. Lagally KM, Cordero J, Good J, Brown DD, McCaw ST. Physiologic and metabolic responses to a continuous functional resistance exercise workout. *J Strength Cond Res*. 2009;23(2):373-9.
12. Kay SJ, Fiatarone Singh MA. The influence of physical activity on abdominal fat: a systematic review of the literature. *Obes Rev*. 2006;7(2):183-200.
13. Kemmler W, von Stengel S, Engelke K, Haberle L, Mayhew JL, Kalender WA. Exercise, body composition, and functional ability: a randomized controlled trial. *Am J Prev Med*. 2010;38(3):279-87.
14. Lamb HJ. Total body fat distribution as part of multiorgan MR imaging: new tool for risk assessment in the metabolic syndrome? *Radiology*. 2010;257(2):307-8.
15. Weiss EP, Racette SB, Villareal DT, et al. Lower extremity muscle size and strength and aerobic capacity decrease with caloric restriction but not with exercise-induced weight loss. *J Appl Physiol*. 2007;102(2):634-40.
16. Valtonen A, Poyhonen T, Sipila S, Heinonen A. Effects of aquatic resistance training on mobility limitation and lower-limb impairments after knee replacement. *Arch Phys Med Rehabil*;91(6):833-9.
17. Petersen SE, Hudsmith LE, Robson MD, et al. Sex-specific characteristics of cardiac function, geometry, and mass in young adult elite athletes. *J Magn Reson Imaging*. 2006;24(2):297-303.
18. Grothues F, Smith GC, Moon JC, et al. Comparison of interstudy reproducibility of cardiovascular magnetic resonance with two-dimensional echocardiography in normal subjects and in patients with heart failure or left ventricular hypertrophy. *Am J Cardiol*. 2002;90(1):29-34.

An die Geschäftsstelle der
Ethik-Kommission
der Medizinischen Fakultät
der FAU Erlangen-Nürnberg
Krankenhausstr. 12
91054 Erlangen

Antrag
an die Ethik-Kommission
der Medizinischen Fakultät

*Bitte **in deutscher Sprache** ausfüllen,*
Zutreffendes bitte ankreuzen.
Für multizentrische Studien mit Vorvotum einer nach Landesrecht gebildeten zuständigen Ethik-Kommission können Sie das verkürzte Antragsformular verwenden, abzurufen unter: http://www.ethik.med.uni-erlangen.de *(Anschlussvotum)*

Antrag auf Beurteilung eines Forschungsprojektes (keine Arzneimittelprüfung)

bitte 9-fach einschließlich Anlagen einreichen sowie 1-mal in elektronischer Fassung

Titel des Projektes:
Einfluss eines 16-wöchigen Kraft-Trainingsprogramms auf leistungsphysiologische und gesundheitsrelevante muskuläre und kardiale Größen bei untrainierten Männern im mittleren Lebensalter. Eine randomisierte kontrollierte Interventionsstudie mit modernen, bildgebenden Verfahren.

I. Projektleitung

1. Name der/des verantwortlichen Projektleiterin/s an der FAU:
Professor Dr. Wolfgang Kemmler[1], Osteoporose-Forschungszentrum, Institut für Medizinische Physik, FAU (Direktor: Professor Dr. Dr. Willi A. Kalender)
App.-Nr. 23999; E-Mail: wolfgang.kemmler@imp.uni-erlangen.de

Angaben über die Qualifikation der/des Versuchsleiterin/s:
Promotion, Habilitation (liegen bereits vor)

2. a) Weitere Teilnehmer/innen vor Ort (alphabetische Reihenfolge):
Prof. Dr. Klaus Engelke, IMP, FAU
PD. Dr. Michael Lell, Radiologisches Institut, FAU
Prof. Dr. Harald Quick, IMP, FAU
PD. Dr. Axel Schmid, Radiologisches Institut, FAU
Dr. Michael Scharf, Radiologisches Institut, FAU
Dr. Simon von Stengel, Osteoporoseforschungszentrum, FAU
Andreas Wittke, IMP, FAU

b) Weitere Prüfzentren (bei multizentrischen Studien): keine

3. Handelt es sich bei diesem Antrag um ein bereits von der Ethik-Kommission der Medizinischen Fakultät der Friedrich-Alexander-Universität Erlangen-Nürnberg begutachtetes Projekt?

ja (bitte lfd. Nr. angeben) ☒ nein

II. Forschungsvorhaben

1. Geplanter Beginn der Studie: September 2012 // voraussichtliches Ende: August 2013
Dauer der Studienteilnahme für den einzelnen Probanden:

Intervention (s.u.): 4 Monate // 8 Monate (Kontrollgruppen-Wartelisten-Design)
Untersuchung (s.u.): ca. 100 min jeweils zu Beginn und Studienende

2. Kurzer Abriss des Projektes *(maximal 1,5 Seiten)*:

Einführung und Fragestellung

Krafttraining gilt auch bei intensiver Durchführung als gesundheitsfördernde Intervention, die sich bei einem zunehmenden Anteil der Bevölkerung immer größerer Beliebtheit erfreut. Neben Körperformung und Attraktivität steht für viele Menschen der präventive Aspekt des Muskeltrainings im Vordergrund. Tatsächlich weisen eine Vielzahl von Untersuchungen (1-5) positive Effekte eines regelmäßig durchgeführten, „überschwelligen" Trainings nach, so auf gesundheitsrelevante muskuläre, physiologische, metabolische und kardiovaskuläre Parameter wie beispielsweise die Körperzusammensetzung/funktionelle Kapazität, Blutfette/Lipoproteine oder Glucoseintoleranz/ Insulinsensitivität. Zudem ist anzunehmen, dass auch relativ rasche funktionelle und morphologische Anpassungserscheinungen des Herzens nachweisbar sind (6, 7). Allerdings liegen keine kernspintomographischen Längsschnittuntersuchungen vor, welche die physiologischen Adaptationserscheinungen des Herzens nach einem mehrmonatigen intensiven Krafttraining bei inaktiven Personen evaluieren. Parallel dazu sind die vorliegenden Methoden, mit denen die Körperzusammensetzung und die muskuläre Massenentwicklung bislang evaluiert wurden, als suboptimal einzuschätzen. Um eine valide und reliable Erfassung der Veränderung muskulärer, physiologischer und kardiologischer Größen als Reaktion auf ein gesundheitsorientiertes Muskeltraining zu gewährleisten, ist der messmethodische Schwerpunkt der vorliegenden Untersuchung auf den Einsatz moderner bildgebender Verfahren (Kernspintomographie (MRT), Computertomographie (CT), Dual Energy X-Ray Absorptiometry (DXA)) und moderner Segmentierungs- und Quantifizierungssoftware gerichtet.
Ziel der Untersuchung ist somit die Evaluierung der Effekte eines intensiven 16-wöchigen Krafttrainingsprogramms auf leistungsphysiologische und gesundheitsrelevante muskuläre und kardiale Größen bei untrainierten Männern im mittleren Lebensalter unter besonderer Berücksichtigung bildgebender Verfahren.

Design:

Randomisierte, kontrollierte, teilverblindete Studie mit Wartegruppe (cross-over)

Endpunkte

Primäre Endpunkte:

- Muskelquerschnitt der Oberschenkelmuskulatur (MRT, CT)
- Intraabdominale Fettmasse (MRT)

Sekundäre Endpunkte:

- Kraftfähigkeiten (u.a. abhängige Variable) [1]
- Gesamtkörperfett und Muskelmasse sowie regionale Verteilung (DXA, MRT)
- Metabolisches Syndrom-Score (8), und 10 Jahres-CHD-Risiko (9)
- Kardiale Masse und enddiastolisches Volumen (MRT)
- Hormonelle Regulation (u.a. freies Testosteron, hGH, Cortisol)
- Schmerzintensität, Quality of Life

[1] Ein Ziel der Studie ist die Identifikation von Variablen, welche die Varianz des Kraft-/Leistungszuwachs am höchsten aufklären.

Stichprobe

Zwei-Gruppen Design, randomisiert, z.T. cross-over (mit Wartegruppe)[2]

- Gruppe 1, n = 40: High Intensity Resistance Training (HIT)[3]
- Gruppe 2, n = 40: Kontrollgruppe (Wartegruppe; im Anschluss „Power-Training[4]“)

Interventionsprogramm

Gruppe 1: HIT mit konventioneller Bewegungsgeschwindigkeit (TUT[5] 2s-1s-2s)

Periodisiertes, progressives Krafttraining über 16 Wochen, basierend auf individuellen Trainingsplänen auf der Basis von 1 RM/x RM-Tests (1, 4, 10 Wochen), zunehmende Intensivierung der Reizhöhe (bis Woche 8), anschließend periodisiertes HIT (70-92,5%, 1RM) mit 2-3 Trainingseinheiten je Woche (1-2 gemeinsame, überwachte Trainingseinheiten; 1-2 Trainingseinheiten in Eigenregie (Vorgaben über Trainingsplan)).

Gruppe 2 (ehemalige Kontrollgruppe): Power-Training (TUT: ϟ-1s-2s)

s.o. aber explosive Ausführung im konzentrischen Bewegungsbereich nach initialer Konditionierung über 6 Wochen. Vergleichbares Trainingsprotokoll, allerdings im Intensitätsbereich von 40-70%, 1RM.

3. Studienbezogene Maßnahmen:

Bitte beschreiben Sie hier alle Maßnahmen, die studienbedingt durchgeführt werden sowie alle erforderlichen Abweichungen von der üblichen Routine-Behandlung:

Interventionsprogramm s.o.

Messungen (jeweils basal und nach 16 Wochen)

Bildgebende Verfahren:

Ganzkörper-MRT und Ganzkörper-DXA zur Erfassung der gesamten und regionalen Körperzusammensetzung.

Lokale MRT und CT am Oberschenkel (über „Muskelbauch“)

Kardiale MRT-Untersuchung mit Kontrastmittel i.v. (Analyse fibrotischer Herzmuskelveränderungen, Strain der Herzmuskulatur, enddiastolisches Volumen (EDV), endsystolisches Volumen (ESV), Schlagvolumen (SV), Ejektionsfraktion (EF) und myokardiale Masse (MM), Herzmuskeldicke).

Blutdruck und Herzfrequenzverhalten in Ruhe

Körperliche Fitness:

Erfassung unterschiedlicher Kraftfähigkeiten (u.a. 1 RM, Schnellkraft) mittels isokinetischem Dynamometer.

Psychosoziale Parameter

Befindlichkeit und Schmerzparameter, QoL (Fragebogen)

Labor

Blutfette/Lipoproteine, Glukose, Insulin, HbAC1, Entzündungsmarker

Testosteron, freies Testosteron, hGH, Cortisol; (evt. noch Parameter des Immunsystems)

Ernährungsanalyse

Analyse über 4 Tage (standardisierte Protokolle)

Anamnese und Risikofaktorenprofil über Fragebogen

---

[2] Die Wartegruppe dient zunächst als parallele Kontrollgruppe zur Interventionsgruppe. Nach Abschluss dieses Untersuchungsabschnittes wird mit dieser Gruppe ebenfalls eine 16-wöchige Intervention durchgeführt, sodass ein eingeschränktes 3-Gruppendesign generiert wird.

[3] Training mit relativ hoher Reizintensität (≥70% des Einwiederholungsmaximums: 1RM)

[4] Training mit explosiver Bewegungsausführung im konzentrischen Bereich (bei vglw. geringer Reizintensität im Bereich 40-60% 1RM)

[5] **T**ime **U**nder **T**ension: Dauer der jeweiligen Bewegungsabschnitte, konzentrisch – isometrisch – exzentrisch in Sec.

4. Wird die Studie gemäß der von der 48. Generalversammlung des Weltärztebundes in Somerset West revidierten Deklaration von Helsinki aus dem Jahre 1996 durchgeführt? Bitte angeben, ob alle anderen Erprobungsmöglichkeiten ausgeschöpft wurden.

Ja, die Intervention wurde in vorhergehenden Studien bereits validiert und optimiert. Die Messtechnologie ist ebenfalls etabliert, wurde aber bislang nur suboptimal zur Validierung der hier genannten Fragestellung eingesetzt.

5. Art des Forschungsvorhabens:
Handelt es sich um
eine diagnostische Prüfung?
eine therapeutische Prüfung?
eine Verträglichkeitsprüfung?
☒ einen ausschließlich wissenschaftlichen Versuch?

6. Gesetzliche Grundlagen

a) Handelt es sich um eine Untersuchung, die dazu bestimmt ist, klinische oder pharmakologische Wirkungen von Arzneimitteln zu erforschen oder nachzuweisen oder Nebenwirkungen festzustellen oder die Resorption, die Verteilung, den Stoffwechsel oder die Ausscheidung zu untersuchen, **mit dem Ziel, sich von der Unbedenklichkeit oder Wirksamkeit des Arzneimittels zu überzeugen** (klinische Prüfung eines Arzneimittels nach §§ 40 Arzneimittelgesetz)?

nein

Bitte begründen. Erläuterungen zum Antrag auf Bewertung einer klinischen Arzneimittelprüfung nach § 40 AMG finden Sie unter http://www.ethik.med.uni-erlangen.de/

b) Handelt es sich um eine klinische Prüfung nach § 20 Medizinproduktegesetz (MPG)?
ja ☒ nein
Bitte begründen. Liegt eine CE-Zertifizierung für das Medizinprodukt vor? Werden zusätzlich invasive oder andere belastende Untersuchungen durchgeführt?
Sämtliche Messverfahren sind CE-zertifiziert und entsprechend MPG geprüft und zugelassen. Invasive Untersuchungen werden nicht durchgeführt, mit Ausnahme von Blutentnahmen und einer venösen Kontrastmittelapplikation.

c) Handelt es sich um ein Vorhaben nach § 8 des Gesetzes zur Regelung des Transfusionswesens (TFG)?
ja ☒ nein

7. Handelt es sich um einen Versuch nach

§ 23 Strahlenschutzverordnung? ☒ ja nein

§ 28 Röntgenverordnung? ☒ ja nein

8. Typ der Studie:

- offen
- ☒ blind
- doppelblind
- ☒ vergleichend
- ☒ randomisiert
- multizentrisch
- ☒ Feldstudie
- Pilotstudie

9. Wissenschaftliche Begründung des Projekts, insbesondere:

a. Erläuterung des Versuchsziels

Das Ziel der Untersuchung ist die Evaluierung eines intensiven körperlichen Krafttrainings auf leistungsphysiologische und gesundheitsrelevante muskuläre und kardiale Größen bei untrainierten Männern im mittleren Lebensalter unter besonderer Berücksichtigung moderner bildgebender Verfahren. Aus radiologischer Sicht ist das Ziele der Studie die Identifikation typischer Kenngrößen der physiologischen Adaption der Körperzusammensetzung und des Herz-Kreislauf-Systems nach unterschiedlichen Typen von Krafttraining (s.o.) sowie der Methodenvergleich kernspintomographischer mit computertomographischen Muskelquerschnitten des Oberschenkels sowie der gesamten und der regionalen Gesamtkörperzusammensetzung mittels MRT vs. dem Goldstandard DXA-Methode.

b. Darstellung des bisherigen Wissensstandes

Eine Vielzahl von wissenschaftlichen Untersuchungen berichten den positiven Effekt eines „Krafttrainings“ auf funktionelle und gesundheitsrelevante physiologische und metabolische Größen (Übersicht in (1, 3, 10, 11)) bei Menschen in mittlerem Lebensalter. Obgleich viele dieser Daten bereits in frühen Studien mit suboptimaler Messmethodik/-technik und schlechter Reproduzierbarkeit evaluiert wurden, gelten sie in der wissenschaftlichen Literatur als absolut verlässlich und werden vielfach zitiert. Moderne bildgebende Verfahren wie die Kernspintomographie (MRT) oder die Computertomographie (CT) im Verbund mit valider Segmentierungstechnologie und quantitativer Analyse haben die in der Vergangenheit eingesetzten Verfahren mit suboptimaler Auflösung und ausschließlich qualitativer Auswerteprozedur bereits in vielen Bereichen ersetzt. So können Risikofaktoren wie die Körperfettverteilung derzeit schon quantifiziert und der Einfluss einer Intervention validiert werden (12-14). Auch die muskuläre Komponente der Körperzusammensetzung und insbesondere der Muskelquerschnitt wurde von einigen neueren Untersuchungen mittels moderner bildgebender Verfahren wie MRT oder CT untersucht (15, 16), die allerdings auf keine validierte Segmentierungs- und Quantifizierungssoftware zurückgreifen konnten.

Dies trifft ebenfalls für die kardiale Volumetrie mittels MRT zu, die derzeit als Goldstandard für die links- und rechtsventrikuläre Volumen- und Massenbestimmung gilt (17). Im Gegensatz zu echokardiographischen Untersuchungen handelt es sich bei der kardialen MRT um ein dreidimensionales Verfahren, welches dadurch eine wesentlich genauere morphologische Darstellung des Herzens ermöglicht (18) und somit u.a.

interventionsbedingte Veränderungen früher zu identifizieren vermag. Darüber hinaus werden sportliche Belastungen immer wieder mit einem plötzlichen Herztod, insbesondere bei männlichen Athleten, assoziiert (23-24). Ob hierfür pathologische morphologische Veränderungen des Myokards durch körperliches Training oder angeborene Organerkrankungen zu Grunde liegen, ist unklar.

10. Angaben zur Nutzen-Risiko-Relation

a. Welcher Nutzen ist von den Ergebnissen der Studie zu erwarten

aa) für die Versuchsteilnehmer?

Steigerung der körperlichen Fitness und physischen Attraktivität durch das Trainingsprogramm. Zudem Verminderung des metabolischen und kardiovaskulären Gesundheitsrisikos und Verbesserung der funktionellen Kapazität. Ggf. Detektion relevanter kardiologischer Befunde (Herzklappendefekte, Kardiomyopathie, Fehlbildungen).

ab) für die Heilkunde?

Beschreibung physiologischer muskulärer, metabolischer, physiologischer und kardialer Adaptation nach intensivem Krafttraining u.a. als Voraussetzung zur Abgrenzung pathologischer Veränderungen und Identifizierung geeigneter Verfahren zur Kontrolle von Trainingseffekten. Sekundäres Ziel ist es, Risikopatienten zu identifizieren, die vor Aufnahme eines spezifischen Trainings ein ausführliches Untersuchungsprogramm durchführen sollten, um Sekundärschäden zur vermeiden.

ac) für die Wissenschaft (z.B. Ergebnisse, die nicht unmittelbar therapeutischen Zwecken dienen)?

Verbesserter Einblick in die Grundlagen trainingsinduzierter muskulärer und kardiovaskulärer Adaptation als Basis optimaler sportwissenschaftlicher Trainings-empfehlungen. Evaluierung und Weiterentwicklung von bildgebenden Untersuchungs-verfahren und computerunterstützter Evaluationssoftware zur Diagnostik und zum Monitoring von Interventionseffekten.

b. Mit welchem Risiko ist die Studie für die Versuchsteilnehmer verbunden?

ba) Welcher Art sind die Risiken? Risikoeinschätzung, vorhersehbare Risiken der Behandlung und sonstiger studienbedingter Verfahren, die eingesetzt werden sollen (einschließlich Schmerz, Unannehmlichkeiten, Beschwerden, Verletzung der persönlichen Integrität und Maßnahmen zur Vermeidung und/oder zur Behandlung von unvorhersehbaren/ unerwünschten Ereignissen)

Die Risiken der Intervention (konsequent angeleitetes Krafttrainingsprogramm) sind sehr gering und bleiben nicht zuletzt aufgrund der intensiven Betreuung deutlich hinten denen eines selbständig durchgeführten Muskeltrainings zurück. Selbstverständlich sind in den ersten Wochen der Intervention leichte trainingsbedingte Beschwerden wie bspw. DOMS (Muskelkater) zu erwarten.

Die radiologischen Verfahren Dual Energy X-Ray Absorptiometrie (Gesamtkörper-DXA) bzw. Computertomographie (medialer Anteil Oberschenkel) sind mit niedrigen Strahlendosen (< 10 µSv pro DXA-Messung) bzw. geringen relativen Dosen (<0.8 mSv/CT-Messung[6]) verbunden. Eine Genehmigung dieser Verfahren

---

[6] Dieser Wert wurde ohne Berücksichtigung entsprechender Schutzmaßnahmen (Abdecken der Gonaden mit Bleischutz errechnet.

wird beim Bundesamt für Strahlenschutz nach Vorliegen der Genehmigung der Ethikkommission selbstverständlich beantragt.

Bei der Blutentnahme und durch periphere Verweilkatheter für die Kontrastmittel-(KM)-Applikation sind Blutergüsse und Infektionen nie komplett ausgeschlossen. In der Kernspintomographie werden nur geringe KM-Mengen eingesetzt. Daher sind die Risiken bei einem Paravasat des KMs in das Weichteilgewebe sowie Nierenbelastungen und mögliche allergische KM-Reaktionen minimiert. Probanden mit relativen oder absoluten Kontraindikationen für eine MRT werden von der Studie ausgeschlossen (s.u.). Insbesondere Probanden mit Nierenfunktionsstörungen oder bekannten KM-Reaktionen. Die KM-Gabe ist notwendig, um relevante kardiale Vorerkrankungen auszuschließen (siehe Ausschlusskriterien), die ein Risiko für die Intervention darstellen können.

bb) Mit welcher Wahrscheinlichkeit ist zu erwarten, daß sich die Risiken realisieren? Wie sicher ist die Wahrscheinlichkeit abschätzbar?

Insgesamt besteht nur eine geringe Wahrscheinlichkeit, dass sich die Risiken realisieren. Die Messungen stellen Standardmessungen der klinischen Routine dar, die von geschultem Fachpersonal sachkundig ausgeführt werden. Die Belastungsvorgaben erfolgen individuell, basierend auf einer Leistungsdiagnostik, sodass eine Überforderung der Teilnehmer kaum zu erwarten ist.

c. Warum ist das mögliche Risiko im Verhältnis zu dem zu erwartenden Nutzen Ihrer Ansicht nach vertretbar?

Es besteht keine wesentlich über das Alltagsrisiko hinausgehende Gefährdung, jedoch ein hoher zu erwartender Nutzen für die Teilnehmer hinsichtlich der Steigerung der körperlichen Fitness, gesundheitsrelevanter Größen, Wohlbefinden, Attraktivität und Selbstwirksamkeit/Kontrollüberzeugung.

d. Werden Zwischenergebnisse ausgewertet, um einen Trend zu erkennen?
ja ☒ nein, kurzer Interventionszeitraum macht Zwischenanalyse inadäquat.

e. Sind Kriterien festgelegt worden, bei deren Eintreten der Versuch geändert oder abgebrochen werden soll? ja, welche? ☒ nein

(allerdings Abbruch des jeweiligen Testverfahrens bei Unwohlsein, oder generell auf Wunsch des Patienten)

11. Bei klinischen Prüfungen nach MPG:

a. Welches Medizinprodukt soll geprüft werden? **entfällt**

b. Wird die klinische Prüfung von einer entsprechend qualifizierten und befugten Person geleitet, die mindestens eine zweijährige Erfahrung in der klinischen Prüfung von Medizinprodukten nachweisen kann? ja nein

c. Wurde (soweit erforderlich) eine dem jeweiligen Stand der wissenschaftlichen Erkenntnisse entsprechende biologische Sicherheitsprüfung oder sonstige für die vorgesehene Zweckbestimmung des Medizinproduktes erforderliche Prüfung durchgeführt? ja nein

d. Wurde (soweit erforderlich) die sicherheitstechnische Unbedenklichkeit für die Anwendung des Medizinproduktes unter Berücksichtigung des Standes der Technik sowie der Arbeitsschutz- und Unfallverhütungsvorschriften nachgewiesen?
ja nein

e. Ist der Leiter der klinischen Prüfung über die Ergebnisse der biologischen Sicherheitsprüfung und die voraussichtlich mit der klinischen Prüfung verbundenen Risiken informiert worden? ja nein

12. a) Ist die Mitarbeit eines Statistikers vorgesehen? ☒ ja nein
b) Welche statistischen Methoden sollen benutzt werden?

Komplettes statistisches Instrumentarium zur Erfassung von Effekten (bspw. Varianzanalysen/nicht parametrische Tests zur Identifikation von Zwischengruppenunterschieden). Zudem regressionsanalytische Modelle zur Aufklärung von Varianzen.

13. a) Handelt es sich um eine multizentrische Studie (d.h. eine nach einem *einzigen* Prüfplan durchgeführte Studie, die in mehr als einer Prüfstelle erfolgt und daher von mehr als einem Prüfer vorgenommen wird)? ja ☒ nein

b) Wurden/Werden an anderer Stelle Studien mit demselben oder einem ähnlichen Ziel durchgeführt? ja, wo? ☒ nein

Es wurden in der Vergangenheit bereits mehrere Untersuchungen mit dem Ziel der Evaluierung eines gesundheitssportlichen Trainings auf muskuläre, physiologische, metabolische und kardiale Parameter (meist isoliert) durchgeführt (s.o.). Diese Studie ist jedoch die erste, die u.a. den Effekt eines intensiven Kraft-/Powertrainings auf muskuläre Parameter, Körperzusammensetzung, metabolische und kardiovaskuläre Größen bei untrainierten Männern in mittlerem Lebensalter auch mittels moderner bildgebender Verfahren und Auswertesoftware evaluiert.

14. Wer hat die Studie initiiert? **Institut für Medizinische Physik**

15. Wer finanziert sie? (*Bitte geben Sie an, ob Drittmittel von nichtöffentlicher Seite beantragt werden. Falls ja, in welcher Höhe?*)

Derzeit sind noch keine Mittel beantragt, es werden jedoch nach positivem Votum der Ethikkommission versucht über unterschiedliche Ebenen Drittmittel zu generieren.

16. Die Aufwandsentschädigung wird übernommen von (*bitte Ansprechpartner benennen*):

Institut für Medizinische Physik (Direktor: Prof. Dr. Dr. W.A. Kalender)

Ansprechpartner: Prof. Dr. Wolfgang Kemmler

III. Angaben zu den Versuchsteilnehmern

1. Anzahl (*bei vergleichenden Studien bitte Aufteilung auf Gruppen angeben*)

80 Personen gesamt:

- Gruppe 1, n = 40: High Intensity Resistance Training (HIT)[7]
- Gruppe 2, n = 40: Kontrollgruppe (Wartegruppe; im Anschluss „Power-Training“)

Bei Nullhypothesen-basierten Studien:
Wurde eine formale Fallzahlschätzung vorgenommen?

☒ ja, Basis CSA-Oberschenkelmuskulatur nein

2. Alter und Geschlecht (*bitte geben Sie das Alter der Versuchsteilnehmer sowie die als Ausschlusskriterien vorgesehenen Ober- und Untergrenzen an*)

---

[7] Training mit relativ hoher Reizintensität (≥70% des Einwiederholungsmaximums: 1RM)

Männer, 30. - 50. Lebensjahr

3. Status: Handelt es sich bei den Versuchsteilnehmern um

☒ gesunde Personen

schwangere oder stillende Frauen

Kinder oder Jugendliche

einschlägig Erkrankte (*bitte geben Sie die Krankheit und das Stadium an*)

Personen, die an anderen Krankheiten leiden? (Insbesondere: psychische Krankheiten, die Zweifel an der Geschäfts- oder Einsichtsfähigkeit begründen)

4. Welche sonstigen **Einschlusskriterien** (z.B. erlaubte Begleitmedikation) sind vorgesehen?

- initial Untrainierte (während der vergangenen 2 Jahre: ≤1 h/Woche Sport mit positivem Effekt auf die Muskulatur; ≤2 h/Woche Sport gesamt)

5. Welche sonstigen **Ausschlusskriterien** (z.B. fortgeschrittene Nieren- oder Leber-insuffizienz, verbotene Begleitmedikation etc.) sind vorgesehen?

- Geschichte leistungssportlicher Ausübung von Disziplinen mit erheblicher Relevanz für Körperzusammensetzung und Kraftfähigkeiten
- pathologische muskuläre, metabolische und kardiale Veränderungen oder Entzündungen; deutlich eingeschränkte Gelenksbeweglichkeit in Knie und Hüfte.
- Medikamente/Erkrankungen mit relevantem Einfluss auf Körperzusammensetzung und Herz-Kreislauf-System
- sehr geringe körperliche Leistungsfähigkeit (< 100 Watt auf dem Fahrradergometer)
- schwere Adipositas (BMI > 35 kg/m^2)
- Abwesenheit ≥ 2 Wochen während des Interventionszeitraums
- Geplante Aufnahme einer relevanten parallelen Trainingsmaßnahme
- Kontraindikationen gegen MRT (Klaustrophobie, Herzschrittmacher, magnetisier-bare intracorporale Fremdkörper); Körpermaße die eine MRT-Messung verhindern
- Drogenmissbrauch

6. Sollen auch Personen teilnehmen, die auf gerichtliche oder behördliche Anordnung in einer Anstalt verwahrt werden?

ja ☒ nein

7. Sollen auch Personen teilnehmen, die sich schon für andere Forschungsvorhaben zur Verfügung gestellt haben?

ja ☒ nein

wie lange muss die letzte Teilnahme zurückliegen?

8. Bei Studien an Minderjährigen (oder sonst nicht geschäftsfähigen Personen) **entfällt**

a. Warum kann die Studie nicht an Erwachsenen (voll Geschäftsfähigen) durchgeführt werden?

b. Sind Aufklärung und Einwilligung der (des) gesetzlichen Vertreter(s) gewährleistet? (*bitte vorformulierte Erklärung beifügen*)

ja nein, weil

c. Sind zusätzliche Aufklärung und Einwilligung der minderjährigen (nicht voll geschäftsfähigen) Versuchsteilnehmer gewährleistet, die selbst in der Lage sind, Wesen, Bedeutung und Tragweite des Versuchs einzusehen und ihren Willen danach zu bestimmen?

ja nein

9. Probandenversicherung

Wird zugunsten der Versuchsteilnehmer eine Versicherung abgeschlossen?

ja (*bitte Police beifügen, aus der die Versicherungsgesellschaft und die Höhe der Versicherungsleistung hervorgeht)*

☒ nein

10. Schweigepflicht/Datenschutz

Werden die ärztlichen Schweigepflicht- und die Datenschutzbestimmungen beachtet?

ja

11. Entgelt für Probanden

Soll den Versuchsteilnehmern ein Entgelt (Aufwandsentschädigung o.ä.) gezahlt werden?

ja, in Höhe von EUR ☒ nein

12. Wie sollen die Versuchsteilnehmer über Wesen, Bedeutung und Tragweite der Studie **aufgeklärt** werden?

Bitte in deutscher Sprache beifügen:

Dokumentation des Inhalts der Patientenaufklärung durch die/den versuchsdurchführende/n Ärztin/Arzt (Merkblatt), insbesondere mit Hinweisen über:

- **Ziele und Methoden** der Studie;
- **Nutzen und Risiko** der Studie;
- bekannte und möglicherweise zu erwartende **Wirkungen und Nebenwirkungen** von Medikamenten;
- Eingriffe, die nur aus wissenschaftlichen Gründen erfolgen;
- ein angebrachtes Verhalten des Patienten während und nach dem Versuch;
- die **Widerruflichkeit** einer Einwilligung;
- das Bestehen und den Umfang der gesetzlichen **Probandenversicherung** (Name/Anschrift/Telefon/Fax der Versicherungsgesellschaft, Nummer der Versicherungspolice) sowie die danach von der Versuchsperson zu beachtenden Obliegenheiten;
- **Ausschlusskriterien** (z.B. Schwangerschaft/Stillzeit);
- Name und Telefon des **Ansprechpartner**s vor Ort.
- **Besondere Aufklärung** über die Situation
  a. bei der randomisierten Studie
  f. beim Blind- und Doppelblindversuch.

13. Wie sollen die Versuchsteilnehmer ihre **Einwilligung** in die Teilnahme an der Studie erklären? (*bitte formulierte deutschsprachige Erklärung mit datenschutzrechtlicher Einwilligungserklärung beifügen*)

Schriftliche Einverständniserklärung nach umfangreicher mündlicher und schriftlicher Aufklärung über Ziele, Nutzen und Risiken der Untersuchung.

Ich weiß, daß auch bei einer positiven Beurteilung des Vorhabens durch die Ethik-Kommission der Medizinischen Fakultät der FAU Erlangen-Nürnberg die ärztliche und juristische Verantwortung für die Durchführung des Projektes uneingeschränkt bei der Leiterin/dem Leiter verbleibt.

Erlangen/Nürnberg
Datum ....

Unterschrift des/der Antragstellers/in

____

(Name in Druckbuchstaben)

Unterschrift der/des Leiterin/Leiters der Einrichtung, in der das Vorhaben durchgeführt werden soll.
Mit der Durchführung des Forschungsvorhabens einverstanden:

Datum ....

Unterschrift des/der Leiters/Leiterin der Einrichtung

____

(Name in Druckbuchstaben)

EK_May09/mit Unterschriftsblatt

Literatur

1. Benson AC, Torode ME, Fiatarone Singh MA. Effects of resistance training on metabolic fitness in children and adolescents: a systematic review. *Obes Rev*. 2008;9(1):43-66.
2. Kelley GA, Kelley KS. Impact of progressive resistance training on lipids and lipoproteins in adults: a meta-analysis of randomized controlled trials. *Prev Med*. 2009;48(1):9-19.
3. Latham NK, Bennett DA, Stretton CM, Anderson CS. Systematic review of progressive resistance strength training in older adults. *J Gerontol A Biol Sci Med Sci*. 2004;59(1):48-61.
4. Macaluso A, De Vito G. Muscle strength, power and adaptations to resistance training in older people. *Eur J Appl Physiol*. 2004;91:450-472.
5. Snowling NJ, Hopkins WG. Effects of different modes of exercise training on glucose control and risk factors for complications in type 2 diabetic patients: a meta-analysis. *Diabetes Care*. 2006;29(11):2518-27.
6. Weineck J. *Optimales Training* Erlangen: Spitta-Verlag; 2007.
7. Weineck J. *Sportbiologie*. Vol. 10 Balingen: Spitta Verlag; 2009.
8. Wijndaele K, Beunen G, Duvigneaud N, et al. A continuous metabolic syndrome risk score: utility for epidemiological analyses. *Diabetes Care*. 2006;29(10):2329.
9. Wilson PW, D'Agostino RB, Levy D, Belanger AM, Silbershatz H, Kannel WB. Prediction of coronary heart disease using risk factor categories. *Circulation*. 1998;97(18):1837-47.
10. Asikainen TM, Kukkonen-Harjula K, Miilunpalo S. Exercise for health for early postmenopausal women: a systematic review of randomised controlled trials. *Sports Med*. 2004;34(11):753-78.
11. Lagally KM, Cordero J, Good J, Brown DD, McCaw ST. Physiologic and metabolic responses to a continuous functional resistance exercise workout. *J Strength Cond Res*. 2009;23(2):373-9.
12. Kay SJ, Fiatarone Singh MA. The influence of physical activity on abdominal fat: a systematic review of the literature. *Obes Rev*. 2006;7(2):183-200.
13. Kemmler W, von Stengel S, Engelke K, Haberle L, Mayhew JL, Kalender WA. Exercise, body composition, and functional ability: a randomized controlled trial. *Am J Prev Med*. 2010;38(3):279-87.
14. Lamb HJ. Total body fat distribution as part of multiorgan MR imaging: new tool for risk assessment in the metabolic syndrome? *Radiology*. 2010;257(2):307-8.
15. Weiss EP, Racette SB, Villareal DT, et al. Lower extremity muscle size and strength and aerobic capacity decrease with caloric restriction but not with exercise-induced weight loss. *J Appl Physiol*. 2007;102(2):634-40.
16. Valtonen A, Poyhonen T, Sipila S, Heinonen A. Effects of aquatic resistance training on mobility limitation and lower-limb impairments after knee replacement. *Arch Phys Med Rehabil*;91(6):833-9.
17. Petersen SE, Hudsmith LE, Robson MD, et al. Sex-specific characteristics of cardiac function, geometry, and mass in young adult elite athletes. *J Magn Reson Imaging*. 2006;24(2):297-303.
18. Grothues F, Smith GC, Moon JC, et al. Comparison of interstudy reproducibility of cardiovascular magnetic resonance with two-dimensional echocardiography in normal subjects and in patients with heart failure or left ventricular hypertrophy. *Am J Cardiol*. 2002;90(1):29-34.

An die Geschäftsstelle der
Ethik-Kommission
der Medizinischen Fakultät
der FAU Erlangen-Nürnberg
Krankenhausstr. 12
91054 Erlangen

Antrag
an die Ethik-Kommission
der Medizinischen Fakultät

*Bitte **in deutscher Sprache** ausfüllen,*
Zutreffendes bitte ankreuzen.
Für multizentrische Studien mit Vorvotum einer nach Landesrecht gebildeten zuständigen Ethik-Kommission können Sie das verkürzte Antragsformular verwenden, abzurufen unter: http://www.ethik.med.uni-erlangen.de *(Anschlussvotum)*

Antrag auf Beurteilung eines Forschungsprojektes (keine Arzneimittelprüfung)

bitte 9-fach einschließlich Anlagen einreichen sowie 1-mal in elektronischer Fassung

Titel des Projektes:
Einfluss eines 16-wöchigen Kraft-Trainingsprogramms auf leistungsphysiologische und gesundheitsrelevante muskuläre und kardiale Größen bei untrainierten Männern im mittleren Lebensalter. Eine randomisierte kontrollierte Interventionsstudie mit modernen, bildgebenden Verfahren.

I. Projektleitung

1. Name der/des verantwortlichen Projektleiterin/s an der FAU:
Professor Dr. Wolfgang Kemmler[1], Osteoporose-Forschungszentrum, Institut für Medizinische Physik, FAU (Direktor: Professor Dr. Dr. Willi A. Kalender)
App.-Nr. 23999; E-Mail: wolfgang.kemmler@imp.uni-erlangen.de

Angaben über die Qualifikation der/des Versuchsleiterin/s:
Promotion, Habilitation (liegen bereits vor)

2. a) Weitere Teilnehmer/innen vor Ort (alphabetische Reihenfolge):
Prof. Dr. Klaus Engelke, IMP, FAU
PD. Dr. Michael Lell, Radiologisches Institut, FAU
Prof. Dr. Harald Quick, IMP, FAU
PD. Dr. Axel Schmid, Radiologisches Institut, FAU
Dr. Michael Scharf, Radiologisches Institut, FAU
Dr. Simon von Stengel, Osteoporoseforschungszentrum, FAU
Andreas Wittke, IMP, FAU

b) Weitere Prüfzentren (bei multizentrischen Studien): keine

3. Handelt es sich bei diesem Antrag um ein bereits von der Ethik-Kommission der Medizinischen Fakultät der Friedrich-Alexander-Universität Erlangen-Nürnberg begutachtetes Projekt?

ja (bitte lfd. Nr. angeben) ☒ nein

II. Forschungsvorhaben

1. Geplanter Beginn der Studie: September 2012 // voraussichtliches Ende: August 2013
Dauer der Studienteilnahme für den einzelnen Probanden:

Intervention (s.u.): 4 Monate // 8 Monate (Kontrollgruppen-Wartelisten-Design)
Untersuchung (s.u.): ca. 100 min jeweils zu Beginn und Studienende

2. Kurzer Abriss des Projektes *(maximal 1,5 Seiten)*:

Einführung und Fragestellung
Krafttraining gilt auch bei intensiver Durchführung als gesundheitsfördernde Intervention, die sich bei einem zunehmenden Anteil der Bevölkerung immer größerer Beliebtheit erfreut. Neben Körperformung und Attraktivität steht für viele Menschen der präventive Aspekt des Muskeltrainings im Vordergrund. Tatsächlich weisen eine Vielzahl von Untersuchungen (1-5) positive Effekte eines regelmäßig durchgeführten, „überschwelligen“ Trainings nach, so auf gesundheitsrelevante muskuläre, physiologische, metabolische und kardiovaskuläre Parameter wie beispielsweise die Körperzusammensetzung/funktionelle Kapazität, Blutfette/Lipoproteine oder Glucoseintoleranz/ Insulinsensitivität. Zudem ist anzunehmen, dass auch relativ rasche funktionelle und morphologische Anpassungserscheinungen des Herzens nachweisbar sind (6, 7). Allerdings liegen keine kernspintomographischen Längsschnittuntersuchungen vor, welche die physiologischen Adaptationserscheinungen des Herzens nach einem mehrmonatigen intensiven Krafttraining bei inaktiven Personen evaluieren. Parallel dazu sind die vorliegenden Methoden, mit denen die Körperzusammensetzung und die muskuläre Massenentwicklung bislang evaluiert wurden, als suboptimal einzuschätzen. Um eine valide und reliable Erfassung der Veränderung muskulärer, physiologischer und kardiologischer Größen als Reaktion auf ein gesundheitsorientiertes Muskeltraining zu gewährleisten, ist der messmethodische Schwerpunkt der vorliegenden Untersuchung auf den Einsatz moderner bildgebender Verfahren (Kernspintomographie (MRT), Computertomographie (CT), Dual Energy X-Ray Absorptiometry (DXA)) und moderner Segmentierungs- und Quantifizierungssoftware gerichtet.
Ziel der Untersuchung ist somit die Evaluierung der Effekte eines intensiven 16-wöchigen Krafttrainingsprogramms auf leistungsphysiologische und gesundheitsrelevante muskuläre und kardiale Größen bei untrainierten Männern im mittleren Lebensalter unter besonderer Berücksichtigung bildgebender Verfahren.

Design:
Randomisierte, kontrollierte, teilverblindete Studie mit Wartegruppe (cross-over)

Endpunkte
Primäre Endpunkte:

- Muskelquerschnitt der Oberschenkelmuskulatur (MRT, CT)
- Intraabdominale Fettmasse (MRT)

Sekundäre Endpunkte:

- Kraftfähigkeiten (u.a. abhängige Variable) [1]
- Gesamtkörperfett und Muskelmasse sowie regionale Verteilung (DXA, MRT)
- Metabolisches Syndrom-Score (8), und 10 Jahres-CHD-Risiko (9)
- Kardiale Masse und enddiastolisches Volumen (MRT)
- Hormonelle Regulation (u.a. freies Testosteron, hGH, Cortisol)
- Schmerzintensität, Quality of Life

[1] Ein Ziel der Studie ist die Identifikation von Variablen, welche die Varianz des Kraft-/Leistungszuwachs am höchsten aufklären.

Stichprobe

Zwei-Gruppen Design, randomisiert, z.T. cross-over (mit Wartegruppe)[2]

- Gruppe 1, n = 40: High Intensity Resistance Training (HIT)[3]
- Gruppe 2, n = 40: Kontrollgruppe (Wartegruppe; im Anschluss „Power-Training[4]“)

Interventionsprogramm

Gruppe 1: HIT mit konventioneller Bewegungsgeschwindigkeit (TUT[5] 2s-1s-2s)

Periodisiertes, progressives Krafttraining über 16 Wochen, basierend auf individuellen Trainingsplänen auf der Basis von 1 RM/x RM-Tests (1, 4, 10 Wochen), zunehmende Intensivierung der Reizhöhe (bis Woche 8), anschließend periodisiertes HIT (70-92,5%, 1RM) mit 2-3 Trainingseinheiten je Woche (1-2 gemeinsame, überwachte Trainingseinheiten; 1-2 Trainingseinheiten in Eigenregie (Vorgaben über Trainingsplan)).

Gruppe 2 (ehemalige Kontrollgruppe): Power-Training (TUT: ϟ-1s-2s)

s.o. aber explosive Ausführung im konzentrischen Bewegungsbereich nach initialer Konditionierung über 6 Wochen. Vergleichbares Trainingsprotokoll, allerdings im Intensitätsbereich von 40-70%, 1RM.

3. Studienbezogene Maßnahmen:

Bitte beschreiben Sie hier alle Maßnahmen, die studienbedingt durchgeführt werden sowie alle erforderlichen Abweichungen von der üblichen Routine-Behandlung:

Interventionsprogramm s.o.

Messungen (jeweils basal und nach 16 Wochen)

Bildgebende Verfahren:

Ganzkörper-MRT und Ganzkörper-DXA zur Erfassung der gesamten und regionalen Körperzusammensetzung.

Lokale MRT und CT am Oberschenkel (über „Muskelbauch“)

Kardiale MRT-Untersuchung mit Kontrastmittel i.v. (Analyse fibrotischer Herzmuskelveränderungen, Strain der Herzmuskulatur, enddiastolisches Volumen (EDV), endsystolisches Volumen (ESV), Schlagvolumen (SV), Ejektionsfraktion (EF) und myokardiale Masse (MM), Herzmuskeldicke).

Blutdruck und Herzfrequenzverhalten in Ruhe

Körperliche Fitness:

Erfassung unterschiedlicher Kraftfähigkeiten (u.a. 1 RM, Schnellkraft) mittels isokinetischem Dynamometer.

Psychosoziale Parameter

Befindlichkeit und Schmerzparameter, QoL (Fragebogen)

Labor

Blutfette/Lipoproteine, Glukose, Insulin, HbAC1, Entzündungsmarker

Testosteron, freies Testosteron, hGH, Cortisol; (evt. noch Parameter des Immunsystems)

Ernährungsanalyse

Analyse über 4 Tage (standardisierte Protokolle)

Anamnese und Risikofaktorenprofil über Fragebogen

---

[2] Die Wartegruppe dient zunächst als parallele Kontrollgruppe zur Interventionsgruppe. Nach Abschluss dieses Untersuchungsabschnittes wird mit dieser Gruppe ebenfalls eine 16-wöchige Intervention durchgeführt, sodass ein eingeschränktes 3-Gruppendesign generiert wird.

[3] Training mit relativ hoher Reizintensität (≥70% des Einwiederholungsmaximums: 1RM)

[4] Training mit explosiver Bewegungsausführung im konzentrischen Bereich (bei vglw. geringer Reizintensität im Bereich 40-60% 1RM)

[5] **T**ime **U**nder **T**ension: Dauer der jeweiligen Bewegungsabschnitte, konzentrisch – isometrisch – exzentrisch in Sec.

4. Wird die Studie gemäß der von der 48. Generalversammlung des Weltärztebundes in Somerset West revidierten Deklaration von Helsinki aus dem Jahre 1996 durchgeführt? Bitte angeben, ob alle anderen Erprobungsmöglichkeiten ausgeschöpft wurden.

Ja, die Intervention wurde in vorhergehenden Studien bereits validiert und optimiert. Die Messtechnologie ist ebenfalls etabliert, wurde aber bislang nur suboptimal zur Validierung der hier genannten Fragestellung eingesetzt.

5. Art des Forschungsvorhabens:
Handelt es sich um
eine diagnostische Prüfung?
eine therapeutische Prüfung?
eine Verträglichkeitsprüfung?
☒ einen ausschließlich wissenschaftlichen Versuch?

6. Gesetzliche Grundlagen

a) Handelt es sich um eine Untersuchung, die dazu bestimmt ist, klinische oder pharmakologische Wirkungen von Arzneimitteln zu erforschen oder nachzuweisen oder Nebenwirkungen festzustellen oder die Resorption, die Verteilung, den Stoffwechsel oder die Ausscheidung zu untersuchen, **mit dem Ziel, sich von der Unbedenklichkeit oder Wirksamkeit des Arzneimittels zu überzeugen** (klinische Prüfung eines Arzneimittels nach §§ 40 Arzneimittelgesetz)?

nein

Bitte begründen. Erläuterungen zum Antrag auf Bewertung einer klinischen Arzneimittelprüfung nach § 40 AMG finden Sie unter http://www.ethik.med.uni-erlangen.de/

b) Handelt es sich um eine klinische Prüfung nach § 20 Medizinproduktegesetz (MPG)?
ja ☒ nein
Bitte begründen. Liegt eine CE-Zertifizierung für das Medizinprodukt vor? Werden zusätzlich invasive oder andere belastende Untersuchungen durchgeführt?
Sämtliche Messverfahren sind CE-zertifiziert und entsprechend MPG geprüft und zugelassen. Invasive Untersuchungen werden nicht durchgeführt, mit Ausnahme von Blutentnahmen und einer venösen Kontrastmittelapplikation.

c) Handelt es sich um ein Vorhaben nach § 8 des Gesetzes zur Regelung des Transfusionswesens (TFG)?
ja ☒ nein

7. Handelt es sich um einen Versuch nach
§ 23 Strahlenschutzverordnung? ☒ ja nein
§ 28 Röntgenverordnung? ☒ ja nein

8. Typ der Studie:
- offen
- ☒ blind
- doppelblind
- ☒ vergleichend
- ☒ randomisiert
- multizentrisch
- ☒ Feldstudie
- Pilotstudie

9. Wissenschaftliche Begründung des Projekts, insbesondere:
a. Erläuterung des Versuchsziels

Das Ziel der Untersuchung ist die Evaluierung eines intensiven körperlichen Krafttrainings auf leistungsphysiologische und gesundheitsrelevante muskuläre und kardiale Größen bei untrainierten Männern im mittleren Lebensalter unter besonderer Berücksichtigung moderner bildgebender Verfahren. Aus radiologischer Sicht ist das Ziele der Studie die Identifikation typischer Kenngrößen der physiologischen Adaption der Körperzusammensetzung und des Herz-Kreislauf-Systems nach unterschiedlichen Typen von Krafttraining (s.o.) sowie der Methodenvergleich kernspintomographischer mit computertomographischen Muskelquerschnitten des Oberschenkels sowie der gesamten und der regionalen Gesamtkörperzusammensetzung mittels MRT vs. dem Goldstandard DXA-Methode.

b. Darstellung des bisherigen Wissensstandes

Eine Vielzahl von wissenschaftlichen Untersuchungen berichten den positiven Effekt eines „Krafttrainings" auf funktionelle und gesundheitsrelevante physiologische und metabolische Größen (Übersicht in (1, 3, 10, 11)) bei Menschen in mittlerem Lebensalter. Obgleich viele dieser Daten bereits in frühen Studien mit suboptimaler Messmethodik/-technik und schlechter Reproduzierbarkeit evaluiert wurden, gelten sie in der wissenschaftlichen Literatur als absolut verlässlich und werden vielfach zitiert. Moderne bildgebende Verfahren wie die Kernspintomographie (MRT) oder die Computertomographie (CT) im Verbund mit valider Segmentierungstechnologie und quantitativer Analyse haben die in der Vergangenheit eingesetzten Verfahren mit suboptimaler Auflösung und ausschließlich qualitativer Auswerteprozedur bereits in vielen Bereichen ersetzt. So können Risikofaktoren wie die Körperfettverteilung derzeit schon quantifiziert und der Einfluss einer Intervention validiert werden (12-14). Auch die muskuläre Komponente der Körperzusammensetzung und insbesondere der Muskelquerschnitt wurde von einigen neueren Untersuchungen mittels moderner bildgebender Verfahren wie MRT oder CT untersucht (15, 16), die allerdings auf keine validierte Segmentierungs- und Quantifizierungssoftware zurückgreifen konnten.

Dies trifft ebenfalls für die kardiale Volumetrie mittels MRT zu, die derzeit als Goldstandard für die links- und rechtsventrikuläre Volumen- und Massenbestimmung gilt (17). Im Gegensatz zu echokardiographischen Untersuchungen handelt es sich bei der kardialen MRT um ein dreidimensionales Verfahren, welches dadurch eine wesentlich genauere morphologische Darstellung des Herzens ermöglicht (18) und somit u.a.

interventionsbedingte Veränderungen früher zu identifizieren vermag. Darüber hinaus werden sportliche Belastungen immer wieder mit einem plötzlichen Herztod, insbesondere bei männlichen Athleten, assoziiert (23-24). Ob hierfür pathologische morphologische Veränderungen des Myokards durch körperliches Training oder angeborene Organerkrankungen zu Grunde liegen, ist unklar.

10. Angaben zur Nutzen-Risiko-Relation

a. Welcher Nutzen ist von den Ergebnissen der Studie zu erwarten

aa) für die Versuchsteilnehmer?

Steigerung der körperlichen Fitness und physischen Attraktivität durch das Trainingsprogramm. Zudem Verminderung des metabolischen und kardiovaskulären Gesundheitsrisikos und Verbesserung der funktionellen Kapazität. Ggf. Detektion relevanter kardiologischer Befunde (Herzklappendefekte, Kardiomyopathie, Fehlbildungen).

ab) für die Heilkunde?

Beschreibung physiologischer muskulärer, metabolischer, physiologischer und kardialer Adaptation nach intensivem Krafttraining u.a. als Voraussetzung zur Abgrenzung pathologischer Veränderungen und Identifizierung geeigneter Verfahren zur Kontrolle von Trainingseffekten. Sekundäres Ziel ist es, Risikopatienten zu identifizieren, die vor Aufnahme eines spezifischen Trainings ein ausführliches Untersuchungsprogramm durchführen sollten, um Sekundärschäden zur vermeiden.

ac) für die Wissenschaft (z.B. Ergebnisse, die nicht unmittelbar therapeutischen Zwecken dienen)?

Verbesserter Einblick in die Grundlagen trainingsinduzierter muskulärer und kardiovaskulärer Adaptation als Basis optimaler sportwissenschaftlicher Trainings-empfehlungen. Evaluierung und Weiterentwicklung von bildgebenden Untersuchungs-verfahren und computerunterstützter Evaluationssoftware zur Diagnostik und zum Monitoring von Interventionseffekten.

b. Mit welchem Risiko ist die Studie für die Versuchsteilnehmer verbunden?

ba) Welcher Art sind die Risiken? Risikoeinschätzung, vorhersehbare Risiken der Behandlung und sonstiger studienbedingter Verfahren, die eingesetzt werden sollen (einschließlich Schmerz, Unannehmlichkeiten, Beschwerden, Verletzung der persönlichen Integrität und Maßnahmen zur Vermeidung und/oder zur Behandlung von unvorhersehbaren/ unerwünschten Ereignissen)

Die Risiken der Intervention (konsequent angeleitetes Krafttrainingsprogramm) sind sehr gering und bleiben nicht zuletzt aufgrund der intensiven Betreuung deutlich hinten denen eines selbständig durchgeführten Muskeltrainings zurück. Selbstverständlich sind in den ersten Wochen der Intervention leichte trainingsbedingte Beschwerden wie bspw. DOMS (Muskelkater) zu erwarten.

Die radiologischen Verfahren Dual Energy X-Ray Absorptiometrie (Gesamtkörper-DXA) bzw. Computertomographie (medialer Anteil Oberschenkel) sind mit niedrigen Strahlendosen (< 10 µSv pro DXA-Messung) bzw. geringen relativen Dosen (<0.8 mSv/CT-Messung[6]) verbunden. Eine Genehmigung dieser Verfahren

[6] Dieser Wert wurde ohne Berücksichtigung entsprechender Schutzmaßnahmen (Abdecken der Gonaden mit Bleischutz errechnet.

wird beim Bundesamt für Strahlenschutz nach Vorliegen der Genehmigung der Ethikkommission selbstverständlich beantragt.

Bei der Blutentnahme und durch periphere Verweilkatheter für die Kontrastmittel-(KM)-Applikation sind Blutergüsse und Infektionen nie komplett ausgeschlossen. In der Kernspintomographie werden nur geringe KM-Mengen eingesetzt. Daher sind die Risiken bei einem Paravasat des KMs in das Weichteilgewebe sowie Nierenbelastungen und mögliche allergische KM-Reaktionen minimiert. Probanden mit relativen oder absoluten Kontraindikationen für eine MRT werden von der Studie ausgeschlossen (s.u.). Insbesondere Probanden mit Nierenfunktionsstörungen oder bekannten KM-Reaktionen. Die KM-Gabe ist notwendig, um relevante kardiale Vorerkrankungen auszuschließen (siehe Ausschlusskriterien), die ein Risiko für die Intervention darstellen können.

bb) Mit welcher Wahrscheinlichkeit ist zu erwarten, daß sich die Risiken realisieren? Wie sicher ist die Wahrscheinlichkeit abschätzbar?

Insgesamt besteht nur eine geringe Wahrscheinlichkeit, dass sich die Risiken realisieren. Die Messungen stellen Standardmessungen der klinischen Routine dar, die von geschultem Fachpersonal sachkundig ausgeführt werden. Die Belastungsvorgaben erfolgen individuell, basierend auf einer Leistungsdiagnostik, sodass eine Überforderung der Teilnehmer kaum zu erwarten ist.

c. Warum ist das mögliche Risiko im Verhältnis zu dem zu erwartenden Nutzen Ihrer Ansicht nach vertretbar?

Es besteht keine wesentlich über das Alltagsrisiko hinausgehende Gefährdung, jedoch ein hoher zu erwartender Nutzen für die Teilnehmer hinsichtlich der Steigerung der körperlichen Fitness, gesundheitsrelevanter Größen, Wohlbefinden, Attraktivität und Selbstwirksamkeit/Kontrollüberzeugung.

d. Werden Zwischenergebnisse ausgewertet, um einen Trend zu erkennen?
ja ☒ nein, kurzer Interventionszeitraum macht Zwischenanalyse inadäquat.

e. Sind Kriterien festgelegt worden, bei deren Eintreten der Versuch geändert oder abgebrochen werden soll? ja, welche? ☒ nein

(allerdings Abbruch des jeweiligen Testverfahrens bei Unwohlsein, oder generell auf Wunsch des Patienten)

11. Bei klinischen Prüfungen nach MPG:

a. Welches Medizinprodukt soll geprüft werden? **entfällt**

b. Wird die klinische Prüfung von einer entsprechend qualifizierten und befugten Person geleitet, die mindestens eine zweijährige Erfahrung in der klinischen Prüfung von Medizinprodukten nachweisen kann? ja nein

c. Wurde (soweit erforderlich) eine dem jeweiligen Stand der wissenschaftlichen Erkenntnisse entsprechende biologische Sicherheitsprüfung oder sonstige für die vorgesehene Zweckbestimmung des Medizinproduktes erforderliche Prüfung durchgeführt? ja nein

d. Wurde (soweit erforderlich) die sicherheitstechnische Unbedenklichkeit für die Anwendung des Medizinproduktes unter Berücksichtigung des Standes der Technik sowie der Arbeitsschutz- und Unfallverhütungsvorschriften nachgewiesen?
ja nein

e. Ist der Leiter der klinischen Prüfung über die Ergebnisse der biologischen Sicherheitsprüfung und die voraussichtlich mit der klinischen Prüfung verbundenen Risiken informiert worden? ja nein

12. a) Ist die Mitarbeit eines Statistikers vorgesehen? ☒ ja nein
b) Welche statistischen Methoden sollen benutzt werden?

Komplettes statistisches Instrumentarium zur Erfassung von Effekten (bspw. Varianzanalysen/nicht parametrische Tests zur Identifikation von Zwischengruppenunterschieden). Zudem regressionsanalytische Modelle zur Aufklärung von Varianzen.

13. a) Handelt es sich um eine multizentrische Studie (d.h. eine nach einem *einzigen* Prüfplan durchgeführte Studie, die in mehr als einer Prüfstelle erfolgt und daher von mehr als einem Prüfer vorgenommen wird)? ja ☒ nein

b) Wurden/Werden an anderer Stelle Studien mit demselben oder einem ähnlichen Ziel durchgeführt? ja, wo? ☒ nein

Es wurden in der Vergangenheit bereits mehrere Untersuchungen mit dem Ziel der Evaluierung eines gesundheitssportlichen Trainings auf muskuläre, physiologische, metabolische und kardiale Parameter (meist isoliert) durchgeführt (s.o.). Diese Studie ist jedoch die erste, die u.a. den Effekt eines intensiven Kraft-/Powertrainings auf muskuläre Parameter, Körperzusammensetzung, metabolische und kardiovaskuläre Größen bei untrainierten Männern in mittlerem Lebensalter auch mittels moderner bildgebender Verfahren und Auswertesoftware evaluiert.

14. Wer hat die Studie initiiert? **Institut für Medizinische Physik**

15. Wer finanziert sie? (*Bitte geben Sie an, ob Drittmittel von nichtöffentlicher Seite beantragt werden. Falls ja, in welcher Höhe?*)

Derzeit sind noch keine Mittel beantragt, es werden jedoch nach positivem Votum der Ethikkommission versucht über unterschiedliche Ebenen Drittmittel zu generieren.

16. Die Aufwandsentschädigung wird übernommen von (*bitte Ansprechpartner benennen*):

Institut für Medizinische Physik (Direktor: Prof. Dr. Dr. W.A. Kalender)

Ansprechpartner: Prof. Dr. Wolfgang Kemmler

III. Angaben zu den Versuchsteilnehmern

1. Anzahl (*bei vergleichenden Studien bitte Aufteilung auf Gruppen angeben*)

80 Personen gesamt:
- Gruppe 1, n = 40: High Intensity Resistance Training (HIT)[7]
- Gruppe 2, n = 40: Kontrollgruppe (Wartegruppe; im Anschluss „Power-Training“)

Bei Nullhypothesen-basierten Studien:
Wurde eine formale Fallzahlschätzung vorgenommen?

☒ ja, Basis CSA-Oberschenkelmuskulatur nein

2. Alter und Geschlecht (*bitte geben Sie das Alter der Versuchsteilnehmer sowie die als Ausschlusskriterien vorgesehenen Ober- und Untergrenzen an*)

[7] Training mit relativ hoher Reizintensität (≥70% des Einwiederholungsmaximums: 1RM)

Männer, 30. - 50. Lebensjahr

3. Status: Handelt es sich bei den Versuchsteilnehmern um
   ☒ gesunde Personen
   schwangere oder stillende Frauen
   Kinder oder Jugendliche
   einschlägig Erkrankte (*bitte geben Sie die Krankheit und das Stadium an*)
   Personen, die an anderen Krankheiten leiden? (Insbesondere: psychische Krankheiten, die Zweifel an der Geschäfts- oder Einsichtsfähigkeit begründen)

4. Welche sonstigen **Einschlusskriterien** (z.B. erlaubte Begleitmedikation) sind vorgesehen?
   - initial Untrainierte (während der vergangenen 2 Jahre: ≤1 h/Woche Sport mit positivem Effekt auf die Muskulatur; ≤2 h/Woche Sport gesamt)

5. Welche sonstigen **Ausschlusskriterien** (z.B. fortgeschrittene Nieren- oder Leber-insuffizienz, verbotene Begleitmedikation etc.) sind vorgesehen?
   - Geschichte leistungssportlicher Ausübung von Disziplinen mit erheblicher Relevanz für Körperzusammensetzung und Kraftfähigkeiten
   - pathologische muskuläre, metabolische und kardiale Veränderungen oder Entzündungen; deutlich eingeschränkte Gelenksbeweglichkeit in Knie und Hüfte.
   - Medikamente/Erkrankungen mit relevantem Einfluss auf Körperzusammensetzung und Herz-Kreislauf-System
   - sehr geringe körperliche Leistungsfähigkeit (< 100 Watt auf dem Fahrradergometer)
   - schwere Adipositas (BMI > 35 kg/m^2)
   - Abwesenheit ≥ 2 Wochen während des Interventionszeitraums
   - Geplante Aufnahme einer relevanten parallelen Trainingsmaßnahme
   - Kontraindikationen gegen MRT (Klaustrophobie, Herzschrittmacher, magnetisier-bare intracorporale Fremdkörper); Körpermaße die eine MRT-Messung verhindern
   - Drogenmissbrauch

6. Sollen auch Personen teilnehmen, die auf gerichtliche oder behördliche Anordnung in einer Anstalt verwahrt werden?
   ja ☒ nein

7. Sollen auch Personen teilnehmen, die sich schon für andere Forschungsvorhaben zur Verfügung gestellt haben?
   ja ☒ nein
   wie lange muss die letzte Teilnahme zurückliegen?

8. Bei Studien an Minderjährigen (oder sonst nicht geschäftsfähigen Personen) **entfällt**
   a. Warum kann die Studie nicht an Erwachsenen (voll Geschäftsfähigen) durchgeführt werden?

   b. Sind Aufklärung und Einwilligung der (des) gesetzlichen Vertreter(s) gewährleistet?
   (*bitte vorformulierte Erklärung beifügen*)
   ja nein, weil

c. Sind zusätzliche Aufklärung und Einwilligung der minderjährigen (nicht voll geschäftsfähigen) Versuchsteilnehmer gewährleistet, die selbst in der Lage sind, Wesen, Bedeutung und Tragweite des Versuchs einzusehen und ihren Willen danach zu bestimmen?

ja nein

9. Probandenversicherung

Wird zugunsten der Versuchsteilnehmer eine Versicherung abgeschlossen?

ja (*bitte Police beifügen, aus der die Versicherungsgesellschaft und die Höhe der Versicherungsleistung hervorgeht)*

☒ nein

10. Schweigepflicht/Datenschutz

Werden die ärztlichen Schweigepflicht- und die Datenschutzbestimmungen beachtet?

ja

11. Entgelt für Probanden

Soll den Versuchsteilnehmern ein Entgelt (Aufwandsentschädigung o.ä.) gezahlt werden?

ja, in Höhe von EUR ☒ nein

12. Wie sollen die Versuchsteilnehmer über Wesen, Bedeutung und Tragweite der Studie **aufgeklärt** werden?

Bitte in deutscher Sprache beifügen:

Dokumentation des Inhalts der Patientenaufklärung durch die/den versuchsdurchführende/n Ärztin/Arzt (Merkblatt), insbesondere mit Hinweisen über:

- **Ziele und Methoden** der Studie;
- **Nutzen und Risiko** der Studie;
- bekannte und möglicherweise zu erwartende **Wirkungen und Nebenwirkungen** von Medikamenten;
- Eingriffe, die nur aus wissenschaftlichen Gründen erfolgen;
- ein angebrachtes Verhalten des Patienten während und nach dem Versuch;
- die **Widerruflichkeit** einer Einwilligung;
- das Bestehen und den Umfang der gesetzlichen **Probandenversicherung** (Name/Anschrift/Telefon/Fax der Versicherungsgesellschaft, Nummer der Versicherungspolice) sowie die danach von der Versuchsperson zu beachtenden Obliegenheiten;
- **Ausschlusskriterien** (z.B. Schwangerschaft/Stillzeit);
- Name und Telefon des **Ansprechpartner**s vor Ort.
- **Besondere Aufklärung** über die Situation
  a. bei der randomisierten Studie
  f. beim Blind- und Doppelblindversuch.

13. Wie sollen die Versuchsteilnehmer ihre **Einwilligung** in die Teilnahme an der Studie erklären? (*bitte formulierte deutschsprachige Erklärung mit datenschutzrechtlicher Einwilligungserklärung beifügen*)

Schriftliche Einverständniserklärung nach umfangreicher mündlicher und schriftlicher Aufklärung über Ziele, Nutzen und Risiken der Untersuchung.

Ich weiß, daß auch bei einer positiven Beurteilung des Vorhabens durch die Ethik-Kommission der Medizinischen Fakultät der FAU Erlangen-Nürnberg die ärztliche und juristische Verantwortung für die Durchführung des Projektes uneingeschränkt bei der Leiterin/dem Leiter verbleibt.

Erlangen/Nürnberg
Datum ....

Unterschrift des/der Antragstellers/in

____

(Name in Druckbuchstaben)

Unterschrift der/des Leiterin/Leiters der Einrichtung, in der das Vorhaben durchgeführt werden soll.
Mit der Durchführung des Forschungsvorhabens einverstanden:

Datum ....

Unterschrift des/der Leiters/Leiterin der Einrichtung

____

(Name in Druckbuchstaben)

EK_May09/mit Unterschriftsblatt

Literatur

1. Benson AC, Torode ME, Fiatarone Singh MA. Effects of resistance training on metabolic fitness in children and adolescents: a systematic review. *Obes Rev*. 2008;9(1):43-66.
2. Kelley GA, Kelley KS. Impact of progressive resistance training on lipids and lipoproteins in adults: a meta-analysis of randomized controlled trials. *Prev Med*. 2009;48(1):9-19.
3. Latham NK, Bennett DA, Stretton CM, Anderson CS. Systematic review of progressive resistance strength training in older adults. *J Gerontol A Biol Sci Med Sci*. 2004;59(1):48-61.
4. Macaluso A, De Vito G. Muscle strength, power and adaptations to resistance training in older people. *Eur J Appl Physiol*. 2004;91:450-472.
5. Snowling NJ, Hopkins WG. Effects of different modes of exercise training on glucose control and risk factors for complications in type 2 diabetic patients: a meta-analysis. *Diabetes Care*. 2006;29(11):2518-27.
6. Weineck J. *Optimales Training* Erlangen: Spitta-Verlag; 2007.
7. Weineck J. *Sportbiologie*. Vol. 10 Balingen: Spitta Verlag; 2009.
8. Wijndaele K, Beunen G, Duvigneaud N, et al. A continuous metabolic syndrome risk score: utility for epidemiological analyses. *Diabetes Care*. 2006;29(10):2329.
9. Wilson PW, D'Agostino RB, Levy D, Belanger AM, Silbershatz H, Kannel WB. Prediction of coronary heart disease using risk factor categories. *Circulation*. 1998;97(18):1837-47.
10. Asikainen TM, Kukkonen-Harjula K, Miilunpalo S. Exercise for health for early postmenopausal women: a systematic review of randomised controlled trials. *Sports Med*. 2004;34(11):753-78.
11. Lagally KM, Cordero J, Good J, Brown DD, McCaw ST. Physiologic and metabolic responses to a continuous functional resistance exercise workout. *J Strength Cond Res*. 2009;23(2):373-9.
12. Kay SJ, Fiatarone Singh MA. The influence of physical activity on abdominal fat: a systematic review of the literature. *Obes Rev*. 2006;7(2):183-200.
13. Kemmler W, von Stengel S, Engelke K, Haberle L, Mayhew JL, Kalender WA. Exercise, body composition, and functional ability: a randomized controlled trial. *Am J Prev Med*. 2010;38(3):279-87.
14. Lamb HJ. Total body fat distribution as part of multiorgan MR imaging: new tool for risk assessment in the metabolic syndrome? *Radiology*. 2010;257(2):307-8.
15. Weiss EP, Racette SB, Villareal DT, et al. Lower extremity muscle size and strength and aerobic capacity decrease with caloric restriction but not with exercise-induced weight loss. *J Appl Physiol*. 2007;102(2):634-40.
16. Valtonen A, Poyhonen T, Sipila S, Heinonen A. Effects of aquatic resistance training on mobility limitation and lower-limb impairments after knee replacement. *Arch Phys Med Rehabil*;91(6):833-9.
17. Petersen SE, Hudsmith LE, Robson MD, et al. Sex-specific characteristics of cardiac function, geometry, and mass in young adult elite athletes. *J Magn Reson Imaging*. 2006;24(2):297-303.
18. Grothues F, Smith GC, Moon JC, et al. Comparison of interstudy reproducibility of cardiovascular magnetic resonance with two-dimensional echocardiography in normal subjects and in patients with heart failure or left ventricular hypertrophy. *Am J Cardiol*. 2002;90(1):29-34.
