# Supplementary material for: Myocardial adaption to HI(R)T in previously untrained men with a randomized, longitudinal cardiac MR imaging study (Physical adaptions in Untrained on Strength and Heart trial, PUSH-trial)
Source: PLoS One. 2017 Dec 7;12(12):e0189204. doi: 10.1371/journal.pone.0189204 (PMC5720775; doi:10.1371/journal.pone.0189204)
Supplement: S4 File — (DOCX) [file pone.0189204.s004.docx]

To the office of the

**Application to the**

**Ethics Committee of the Medical Department**

Ethics Committee

of the Medical Department

of the FAU Erlangen-Nuremberg

Krankenhausstr. 12

91054 Erlangen

**Application for the assessment of a research project**

**(no drug study)**

**Project title:**

Influence of a 16-week resistance training program on exercise physiologic and health related muscular and cardiac parameters in middle-aged untrained men. A randomized controlled intervention using modern imaging modalities.

**I. Project leading:**

1. Name of responsible project leader of the FAU:

Prof. Dr. Wolfgang Kemmler, osteoporosis research center, Institute of Medical Physics, FAU (Director: Dr. Dr. Willi A. Kalender)

Information on qualifications of project leader:

Graduation, state doctorate (already available)

2. a) local cooperators (alphabetical order):

Prof. Dr. Klaus Engelke, IMP, FAU

PD. Dr. Michael Lell, Department of Radiology, FAU

Prof. Dr. Harald Quick, IMP, FAU

PD. Dr. Axel Schmid, Department of Radiology, FAU

Dr. Michael Scharf, Department of Radiology, FAU

Dr. Simon von Stengel, osteoporosis research center, FAU

Andreas Wittke, IMP, FAU

b) further cooperating centers (for multicenter studies): none

3. Does the present ethics application refer to a project under assessment by the ethics committee of the medical faculty of the FAU Erlangen-Nuremberg?

**II. Research project**

1. Scheduled begin of intervention: September 2012 // probable study end: August 2013

Duration of study participation for every single test person:

Intervention: (see below): 4 months // 8 months (control group – waiting list- design)

Examinations (see below): approximately 100 minutes at the beginning and at the end of study period, respectively

2. Abstract of the planned project (maximal 1.5 pages)

**Introduction and study aim**

Resistance training, even if intensely performed, is considered as an intervention with health encouraging effects and is more and more popular in the population. Besides body shaping and physical attractiveness the preventive aspect of resistance training plays a major role. In fact, a multitude of investigations (1-5) proved positive effects of a regularly performed training “above threshold”, e.g. on health related muscular, physiologic, metabolic and cardiovascular parameters such as body composition/functional capacity, blood lipids/lipoproteins or glucose intolerance/insulin sensitivity. In addition, it is to be assumed that cardiac functional and morphologic adaptions can be detected relatively quickly (6, 7). However, there are no longitudinal magnetic resonance imaging studies available, which evaluate physiologic cardiac adaption in previously inactive subjects to an intensive resistance training over several months. Parallel, the available methods according to which body composition and skeletal muscle mass has been evaluated so far, are insufficient. In order to guarantee valid and reliable evaluation of changes in muscular, physiologic, and cardiovascular parameters as a consequence to a health-related resistance training focus of this investigation is the application of modern imaging modalities (magnetic resonance imaging, MRI; computed tomography, CT; dual energy X-ray absorptiometry, DXA) and modern segmentation and quantification software.

Hence, the aim of this investigation is to evaluate the effects of an intensive 16-week resistance training program on exercise physiologic and health related muscular and cardiac parameters in middle-aged untrained men in particular regard of imaging modalities.

**Study design:**

Randomized, controlled, partially blinded study with waiting-group (cross-over)

**Study endpoints:**

*Primary study endpoints:*

- muscle cross-section of thigh muscles (MRI, CT)
- intraabdominal fat mass (MRI)

*Secondary study endpoints:*

- strength skills (amongst others dependent variable)^[[1]](#footnote-1)^
- whole body fat and muscle mass as well as regional distribution (DXA, MRI)
- metabolic syndrome score (8), and 10-years-CHD-risk (9)
- myocardial mass and end-diastolic volume (MRI)
- hormonal regulation (amongst others testosterone, hGH, cortisol)
- pain intensity, quality of life

**Sample:**

Two-groups design, randomized, partially cross-over (with waiting-group)^[[2]](#footnote-2)^

- group 1, n = 40: high intensity resistance training (HIT)^[[3]](#footnote-3)^
- group 2, n = 40: control-group (waiting-group; subsequent “power-training^[[4]](#footnote-4)^”)

**Intervention program**

**Group 1: HIT with conventional movement velocity (TUT^[[5]](#footnote-5)^ 2s-1s-2s)**

Periodized, progressive resistance training for 16 weeks, based on individual training schedules on basis of the 1 RM/x RM-test (1, 4, 10 weeks), increasing training intensity (until week 8), subsequent periodized HIT (70-92.5%, 1 RM) with 2-3 training sessions per week (1-2 collective, supervised training sessions; 1-2 single-handed training sessions (requirements by training schedule)).

**Group 2 (former control-group): power-training (TUT: explosive-1s-2s)**

See above, but explosive movement execution in the concentric movement range after an initial conditioning phase over 6 weeks. Comparable study protocol, but with a training intensity range of 40-70%, 1RM.

**3. Study related procedures:**

**Intervention program (see above)**

**Measurements (each at baseline and after 16 weeks)**

Imaging modalities:

whole body MRI and whole body DXA to assess the whole and regional body composition.

MRI and CT of the thigh (over “muscle belly”)

cardiac MRI examination with intravenous contrast agent application (analysis of fibrotic myocardial changes, myocardial strain, end-diastolic volume (EDV), end-systolic volume (ESV), stroke volume (SV), ejection fraction (EF), myocardial mass (MM), and myocardial wall thickness).

Blood pressure and heart frequency at rest

Body fitness:

assessment of various strength skills (amongst others 1 RM, speed-strength) using isokinetic dynamometry.

Psychosocial parameters

mental state and pain parameters, quality of life (questionnaire)

Laboratory

blood lipids/lipoproteins, blood glucose, insulin, HbA1C, inflammatory markers, testosterone, hGH, cortisol; (perhaps also parameters of the immune system)

Dietary analysis

analysis over 4 days (standardized protocols)

anamnesis and risk factor profile assessed by questionnaires

4. Does the study comply with the Declaration of Helsinki from the year 1996, revised by the general assembly of the world college of physicians in Somerset West? Please indicate whether all other test possibilities have been exploited.

Yes, the intervention has been validated and optimized in previous studies. Measurement technology is also established but has only been insufficiently used to validate the herein mentioned objectives so far.

5. Type of research project

Is it

a diagnostic test?

a therapeutic test?

a compatibility test?

X a solely scientific trial?

6. Legal fundamentals

a) Is this study decided to investigate or prove clinic or pharmacologic effects of drugs or to determine side effects or to examine absorption, distribution, metabolism or elimination of drugs, **with the aim to assure oneself of harmlessness or effectiveness of the medicinal product** (clinical test of drugs according to §§40 Medicines Act)?

No

b) Is this a clinical test pursuant to §20 Medicinal Devices Act?

Yes X No

*Please give reasons. Is there a CE-certification for the medicinal product? Will there be performed any other invasive or wearing investigations?*

*All measuring methods are CE-certified and appropriately tested and approved by the Medicinal Devices Act. No invasive examinations are performed, except taking of blood samples and an intravenous contrast agent application.*

c) Is this a project related to §8 transfusion law?

Yes X No

7. Is this a trial according to

§23 Radiation Protection Ordinance?

§28 X-ray Ordinance?

8. Type of study:

open

X blinded

double blinded

X comparing

X randomized

multicenter

X field study

pilot study

9. Scientific rationale for the project, especially:

a. explanation of study aims

The aim of this study is to investigate the effects of an intensive physical training on exercise physiologic and health related muscular and cardiac parameters in middle-aged untrained men with particular focus on modern imaging modalities. From the radiologist point of view the aim of this study is to identify typical parameters of physiologic adaptions in body composition and of the cardiovascular system to different types of resistance training (see above). Another aim is the comparison of magnetic resonance imaging and computed tomography derived muscle cross-section of the thigh as well as a comparison of whole and regional body composition with MRI versus DXA as the reference method.

b. summary of the present state of knowledge

A multitude of scientific investigations reported a positive effect of resistance training on functional and health related physiologic and metabolic parameters (overview in (1,3,10,11)) in middle-aged subjects. Although many of the present data has been evaluated in previous studies with bad reproducibility and using insufficient measurement techniques, they are considered as absolutely reliable in scientific literature and are often cited. Modern imaging modalities like magnetic resonance imaging (MRI) or computed tomography (CT) combined with segmentation technologies and quantitative analysis have replaced previously used methods with worse resolution and exclusively qualitative evaluation procedure in many fields. For example, it is currently possible to quantify risk factors like body fat distribution and to validate the effects of interventions (12-24). Besides, the muscular components of body composition, especially muscle cross-section, have been investigated by some newer trials using modern imaging modalities like MRI or CT (15, 16) without access to validated segmentation and quantification software.

This applies also to cardiac volumetric analysis assessed by MRI, which is currently considered as the gold standard for left and right ventricular volume and mass assessment (17). Compared to echocardiographic examinations cardiac MRI is a three-dimensional method, therefore enabling more accurate morphologic analysis of the heart (18) so that, amongst others, training induced changes can be identified much earlier. Moreover, physical stress is consistently thought to be associated with sudden cardiac death, especially in male athletes (23-24). It is not known whether this is based on pathologic morphologic myocardial changes induced by physical training or hereditary organic diseases.

**10. Specifications about risk-benefit analysis**

**a) What benefit is to be expected from the study?**

aa) for participants?

Training program induced increase in physical fitness and physical attractiveness. In addition, reduction of metabolic and cardiovascular health risk and improvement of functional capacity. Possibly detection of relevant cardiac diseases (valvular heart disease, cardiomyopathy, malformations).

ab) for medical science?

Description of physiologic muscular, metabolic, and cardiac adaption to intensive resistance training, amongst others as a precondition for the differentiation to pathologic changes and to identify appropriate methods to reassess training effects. Secondary aim is to identify patients at risk, who should undergo an extensive examination program before starting with a specific training, to avoid side effects.

ac) for science (e.g. results that do not primarily serve therapeutic purposes)?

Better insight into the principles of training induced muscular and cardiovascular adaption as a basis for ideal sport scientific training recommendations. Evaluation and enhancement of imaging modalities and computer assisted evaluation software for diagnosis and monitoring of training induced effects.

**b) What id the study related risk for participants?**

ba) What kind of risks are there? Risk assessment, predictable risks of intervention and other study related procedures, that are to be conducted (including pain, inconveniences, discomfort, violation of personal integrity and arrangements to avoid and/or to handle unpredictable/undesirable events)

Risks resulting from the intervention (consistently supervised resistance training) are marginal and are much lower than single-handedly performed resistance training, not least because of the intense supervision. Of course, slight training induced complaints like e.g. aching muscles (DOMS, delayed onset muscle soreness) are to be expected during the first weeks of the intervention.

The radiologic methods (dual energy x-ray absorptiometry (whole body DXA) and computed tomography (medial part of the thigh), respectively, are associated with low radiation levels (< 10 µSv per DXA-measurement) or low relative radiation levels (< 0.8 mSv/CT measurement^[[6]](#footnote-6)^), respectively. Approval of these treatments will of course be applied for after the permission of the ethics committee at the Federal Office for Radiation Protection.

Due to taking blood samples and indwelling catheters hematomas and infections cannot always be excluded. Only minimal amounts of contrast agent are applied in MRI. Therefore, risks due to extravasat injuries from contrast agent, stress on kidneys, and possible allergic reactions are minimized. Test persons with relative or absolute contraindications to MRI are excluded from the study (see below). Especially subjects suffering from renal impairment or known allergic reactions will be excluded. Application of intravenous contrast agent is necessary to exclude relevant pre-existing cardiac diseases (see exclusion criteria) for the intervention.

bb) How high is the likelihood of risk realization? How reliably is the likelihood assessable?

On the whole, there is only a low likelihood that risks will put into effect. Measurements are standardized procedures of clinical routine and will be performed by qualified personnel. Training intensity prescriptions are individualized, based on medical assessment of athletic performance, so that overstraining of subjects is not to be expected.

**c) Why is the possible risk justifiable in relation to the expected benefit according to your opinion?**

There is no relevant hazard above everyday risk but a high expected benefit for participants in respect to physical fitness, health related parameters, well-being, attractiveness, and self-efficacy/self-esteem.

d. Will intermediary results be evaluated to assess a tendency?

Yes X no, due to the short intervention period intermediary results are inadequate

e) Have any criteria been fixed according to which the intervention will be modified or interrupted? Yes, which? X No

(but termination of particular test procedures in the case of malaise or in general at the request of study subjects)

11. For clinical investigations according to the Medicinal devices Act?

Not applicable

12. a) Is the participation of a statistician planned? X Yes? No

b) Which statistical method will be used?

Complete statistical possibilities to evaluate study effects (e.g. analysis of variance/non-parametric tests to identify between group differences). In addition, regression analytical models to determine variances.

13. a) Is this a multicenter study (i.e. a study performed on basis of a *single* test plan, conducted in several testing centers and therefore conducted by more than one investigator)? Yes? X No

b) Have there been/are there being conducted studies with the same or comparable aim at other centers?

In the past, several studies have been performed with the aim to evaluate the effects of a health-related training on muscular, physiologic, metabolic, and cardiac parameters (mostly segregated) (see above), This is the first study to evaluate, amongst others, the effect of an intensive resistance-/power training on muscular parameters, body composition, metabolic, and cardiovascular parameters in untrained middle-aged men also with modern imaging modalities and evaluation software.

14. Who initiated the study? The Institute of Medical Physics.

15. Who finances the study? *(Please indicate whether third-party funds from private bodies have been requested. If so how much?).*

At present, no funds have been applied for. But we will try to receive third-party funds from different fields after a positive vote of the ethics committee.

16. Financial recompensation will be provided by *(please name contact person)*:

Institute of Medical Physics (Director: Prof. Dr. Dr. W. A. Kalender)

Contact person: Prof. Dr. Wolfgang Kemmler

**III. Details about study participants**

1. Number *(for comparing studies please indicate subdivision into groups)*

80 subjects total

- group 1, n = 40: high intensity resistance training (HIT)^[[7]](#footnote-7)^
- group 2, n = 40: control-group (waiting- group; subsequent “power-training”)

For null hypothesis based studies:

Has a formal testing of number of cases been done?

X Yes? On basis of CSA thigh muscles and myocardial mass No

2. Age and sex *(please indicate the age of study subjects and the upper and lower limits designated for study exclusion)*

men, 30-50 years of age

3. Status: are the study participants

healthy subjects

pregnant or breast-feeding women

children or adolescents

relevant ill persons (please indicate disease and stage)

persons suffering from other diseases? (in particular: mental illnesses, implying doubts about legal competence or capacity of discernment)

4. Which other **inclusion criteria** (e.g. concurrent medication) are provided?

- initially untrained subjects (during the past 2 years: ≤1 h/week exercise with positive effect on musculature; ≤2 h/week sports in total)

5. Which other **exclusion criteria** (advanced kidney or liver insufficiency, prohibited concurrent medication) are provided?

- history of participation in competitive disciplines with considerable relevance to body composition and strength skills
- pathologic muscular, metabolic, and cardiac diseases or inflammations; considerably restricted flexibility of hip and knee joints.
- drugs/diseases with relevant influence on body composition and cardiovascular system
- very low physical performance (< 100 watts on bicycle ergometer)
- severe obesity (BMI > 35 kg/m^2^)
- absence ≥ 2 weeks during the intervention period
- predictable beginning of a relevant parallel training procedure
- contraindications to MRI (claustrophobia, pace maker, magnetizeable intracorporeal foreign bodies); body dimensions preventing MRI measurements
- drug abuse.

6. Will subjects participate that are kept in a mental institution due to juridical or governmental decree?

Yes? X No

7. Will subjects participate that have provided themselves to other research projects before?

Yes? X No

How long ago must the last participation have been?

8. For studies in underage persons (or other persons without legal competency)

**Not applicable**

9. Clinical trial insurance

Will a clinical trial insurance be concluded for study subjects?

Yes? *(please add insurance policy, detailing insurance company and amount of indemnification payments)*

X No

10. Professional discretion/privacy protection

Will medical confidentiality and data protection specifications be respected?

Yes

11. Fee for participants

Will the participants be remunerated for their efforts? Yes, how much? X No

12. How will study subjects be **informed** about nature, meaning and importance of the study?

*Please add in German language:*

Documentation of the patient information content by the operating medical doctor (information leaflet), especially information about:

- aims and methods of the study;
- benefits and risks of the study;
- well-known and possibly to be expected effects and side-effects of drugs;
- interventions that happen only for scientific reasons;
- appropriate behavior of study subjects during and after the intervention;
- revocability of consent;
- existance and coverage of clinical trial insurance (name/address/telephone number/fax number of insurance company, number of insurance policy) as well as duties that have to be respected by the study subjects;
- exclusion criteria (e.g., pregnancy/lactation);
- name and telephone number of local contact person;
- specific informations about the situation

1. in a randomized study
2. in a double-blinded investigation

13. How do study subjects have to give their consent for study participation? *(please add a verbalized declaration in German with informed consent regarding data protection)*

Written informed consent after detailed oral and written information about aims, benefits and risks of the intervention/examination.

**Literature**

1. Benson AC, Torode ME, Fiatarone Singh MA. Effects of resistance training on metabolic fitness in children and adolescents: a systematic review. Obes Rev. 2008;9(1):43-66.

2. Kelley GA, Kelley KS. Impact of progressive resistance training on lipids and lipoproteins in adults: a meta-analysis of randomized controlled trials. Prev Med. 2009;48(1):9-19.

3. Latham NK, Bennett DA, Stretton CM, Anderson CS. Systematic review of progressive resistance strength training in older adults. J Gerontol A Biol Sci Med Sci. 2004;59(1):48-61.

4. Macaluso A, De Vito G. Muscle strength, power and adaptations to resistance training in older people. Eur J Appl Physiol. 2004;91:450-472.

5. Snowling NJ, Hopkins WG. Effects of different modes of exercise training on glucose control and risk factors for complications in type 2 diabetic patients: a meta-analysis. Diabetes Care. 2006;29(11):2518-27.

6. Weineck J. Optimales Training Erlangen: Spitta-Verlag; 2007.

7. Weineck J. Sportbiologie. Vol. 10 Balingen: Spitta Verlag; 2009.

8. Wijndaele K, Beunen G, Duvigneaud N, et al. A continuous metabolic syndrome risk score: utility for epidemiological analyses. Diabetes Care. 2006;29(10):2329.

9. Wilson PW, D'Agostino RB, Levy D, Belanger AM, Silbershatz H, Kannel WB. Prediction of coronary heart disease using risk factor categories. Circulation. 1998;97(18):1837-47.

10. Asikainen TM, Kukkonen-Harjula K, Miilunpalo S. Exercise for health for early postmenopausal women: a systematic review of randomised controlled trials. Sports Med. 2004;34(11):753-78.

11. Lagally KM, Cordero J, Good J, Brown DD, McCaw ST. Physiologic and metabolic responses to a continuous functional resistance exercise workout. J Strength Cond Res. 2009;23(2):373-9.

12. Kay SJ, Fiatarone Singh MA. The influence of physical activity on abdominal fat: a systematic review of the literature. Obes Rev. 2006;7(2):183-200.

13. Kemmler W, von Stengel S, Engelke K, Haberle L, Mayhew JL, Kalender WA. Exercise, body composition, and functional ability: a randomized controlled trial. Am J Prev Med. 2010;38(3):279-87.

14. Lamb HJ. Total body fat distribution as part of multiorgan MR imaging: new tool for risk assessment in the metabolic syndrome? Radiology. 2010;257(2):307-8.

15. Weiss EP, Racette SB, Villareal DT, et al. Lower extremity muscle size and strength and aerobic capacity decrease with caloric restriction but not with exercise-induced weight loss. J Appl Physiol. 2007;102(2):634-40.

16. Valtonen A, Poyhonen T, Sipila S, Heinonen A. Effects of aquatic resistance training on mobility limitation and lower-limb impairments after knee replacement. Arch Phys Med Rehabil;91(6):833-9.

17. Petersen SE, Hudsmith LE, Robson MD, et al. Sex-specific characteristics of cardiac function, geometry, and mass in young adult elite athletes. J Magn Reson Imaging. 2006;24(2):297-303.

18. Grothues F, Smith GC, Moon JC, et al. Comparison of interstudy reproducibility of cardiovascular magnetic resonance with two-dimensional echocardiography in normal subjects and in patients with heart failure or left ventricular hypertrophy. Am J Cardiol. 2002;90(1):29-34.

1. One aim of the study is the identification of variables, which explain variance of gains in strength/fitness the most. [↑](#footnote-ref-1)
2. Primarily, waiting-group serves as a parallel control-group to the intervention-group. After the end of this project-phase the former waiting-group receives a 16-week intervention as well, so that a limited 3-group-design is generated. [↑](#footnote-ref-2)
3. Training with relatively high intensity (≥70% of the one-repetition maximum: 1RM) [↑](#footnote-ref-3)
4. Training with explosive movement execution in the concentric range (with comparably low intensity in the range of 40-60% 1RM) [↑](#footnote-ref-4)
5. **T**ime **U**nder **T**ension: duration of the several movement phases, concentric – isometric – eccentric in seconds. [↑](#footnote-ref-5)
6. This value was calculated in consideration of appropriate preventive measures (covering testicles with lead protection). [↑](#footnote-ref-6)
7. Training with relatively high intensity (≥ 70% of one repetition maximum: 1RM) [↑](#footnote-ref-7)
