# Supplementary figures and images for: Myocardial adaption to HI(R)T in previously untrained men with a randomized, longitudinal cardiac MR imaging study (Physical adaptions in Untrained on Strength and Heart trial, PUSH-trial)
Source: PLoS One. 2017 Dec 7;12(12):e0189204. doi: 10.1371/journal.pone.0189204 (PMC5720775; doi:10.1371/journal.pone.0189204)

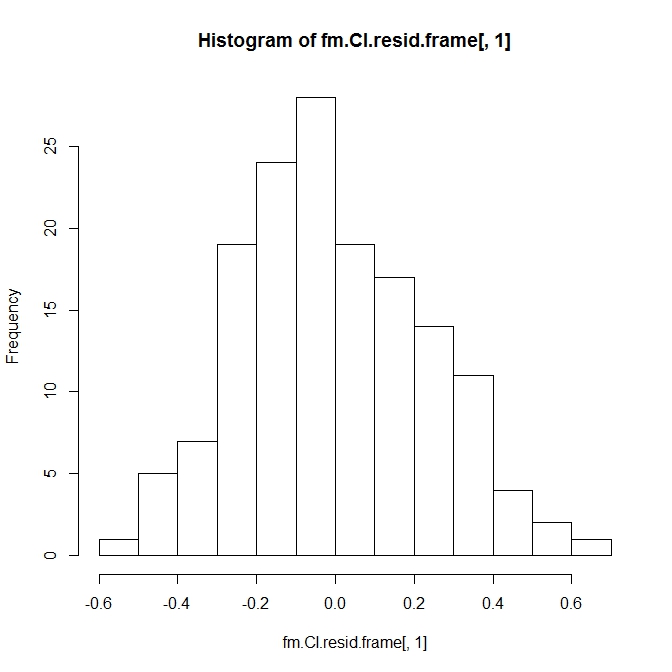


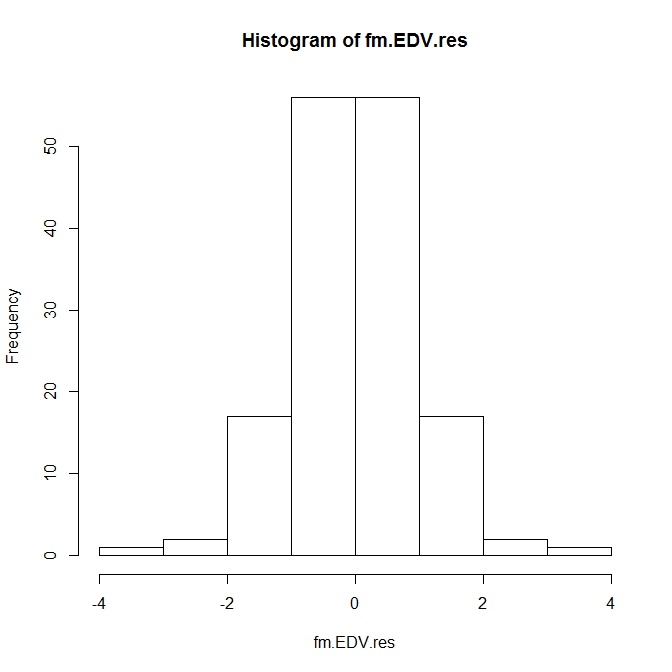


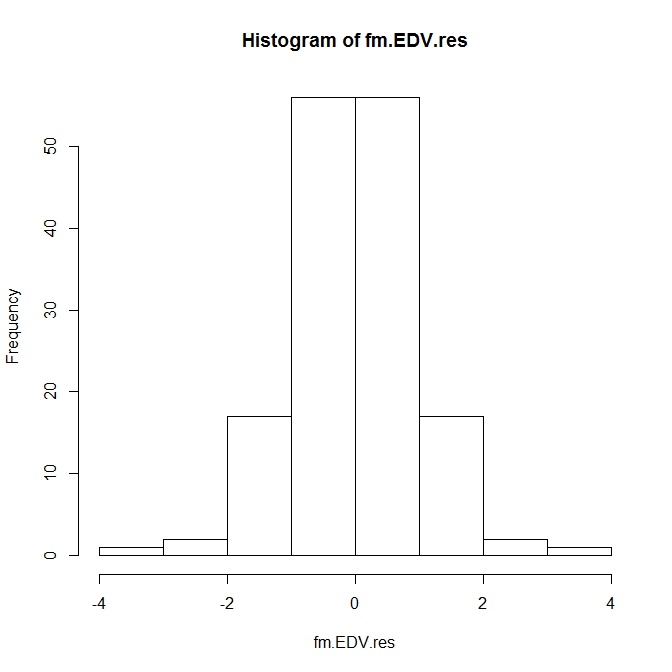


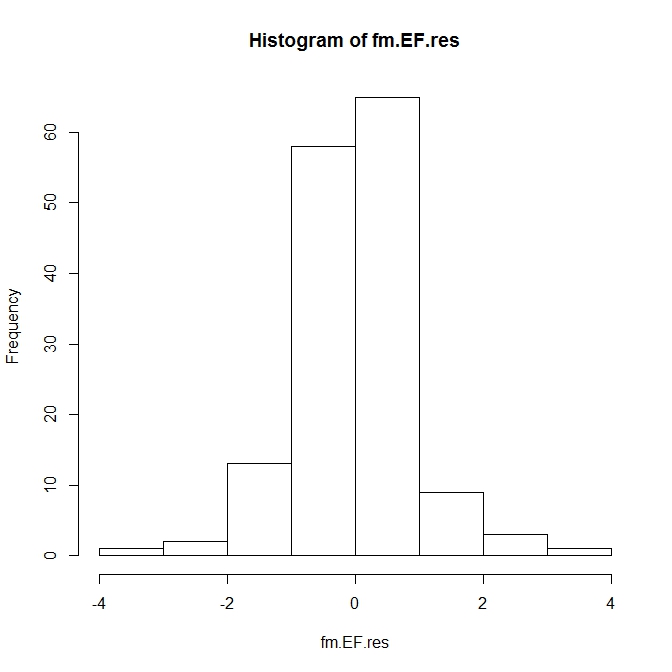


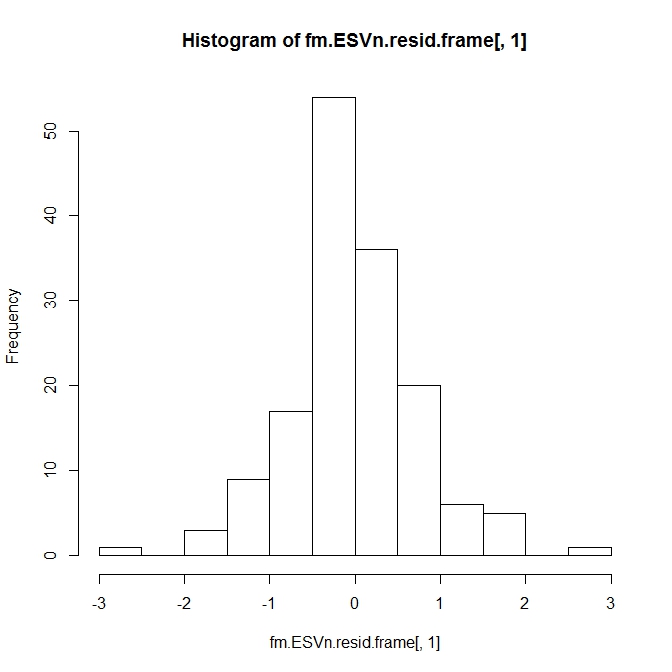


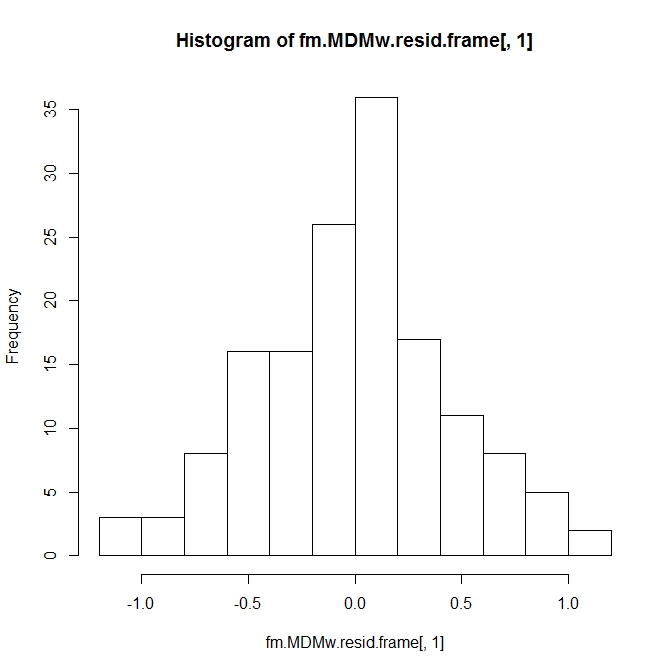


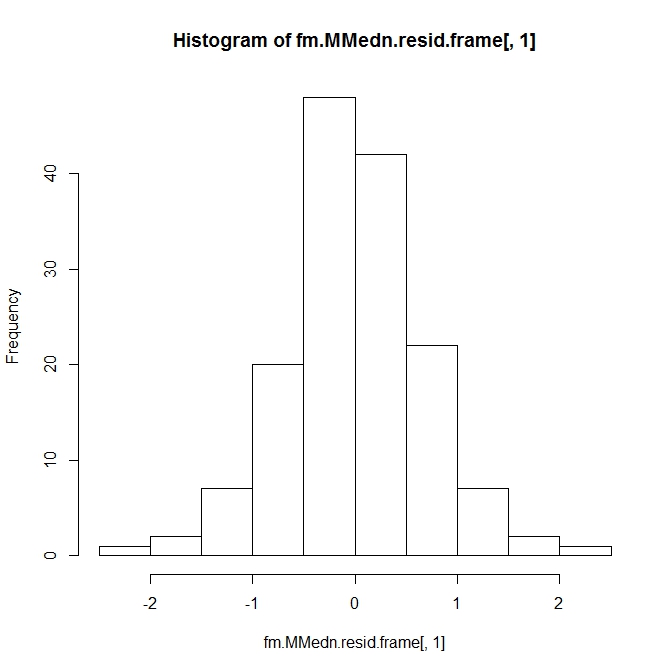


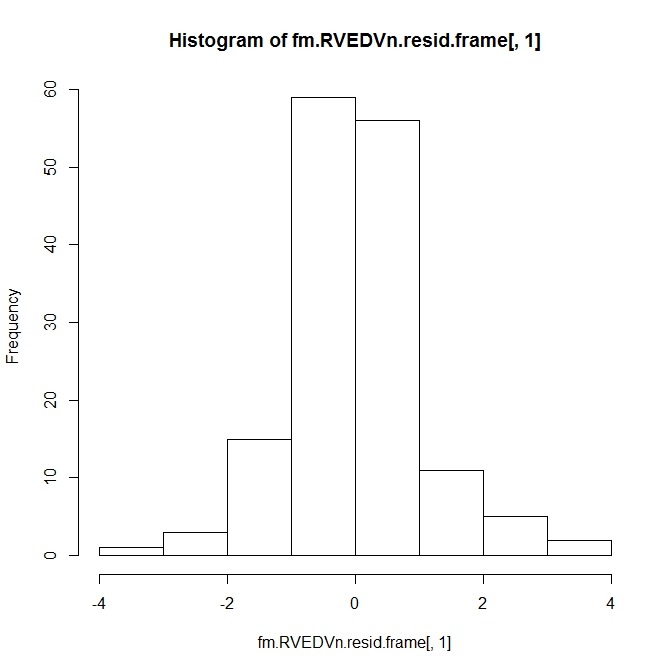


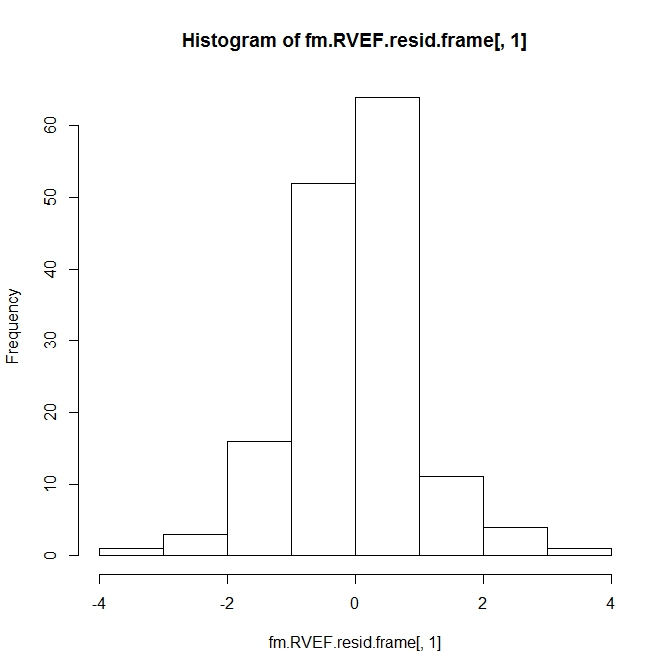

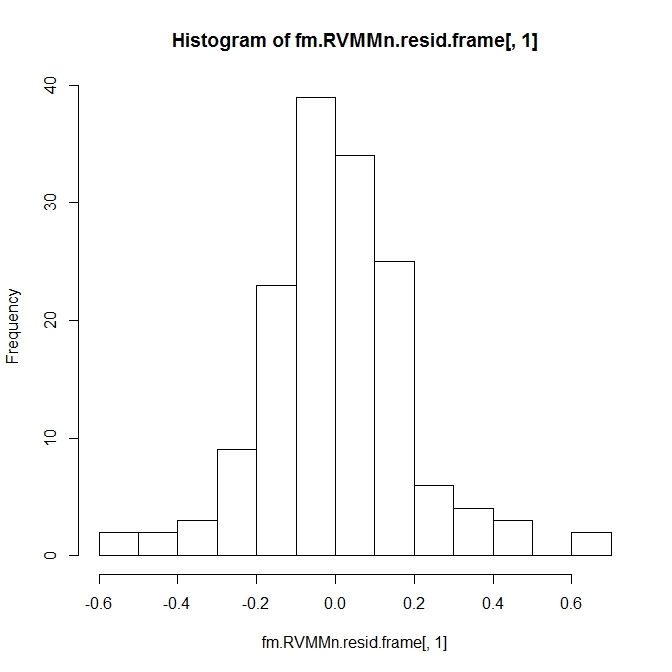

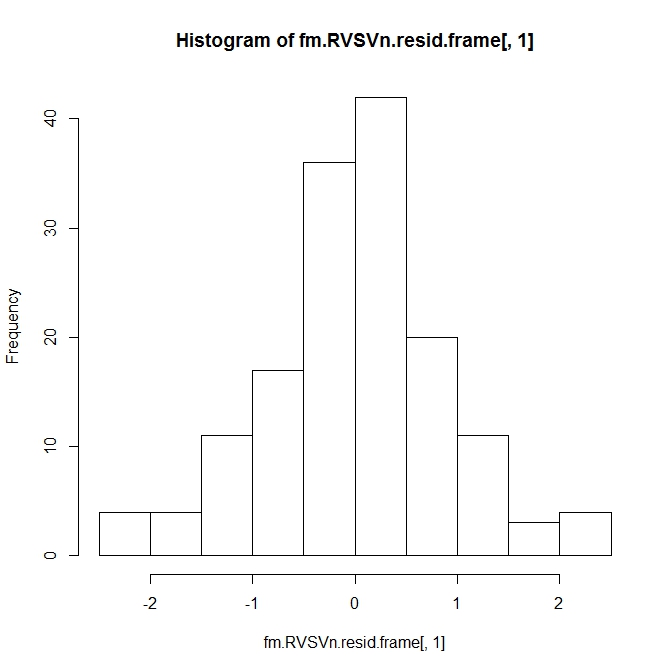

Supplement: S5 File — (DOCX) [file pone.0189204.s005.docx]
